# Skeletal rearrangement of 6,8-dioxabicyclo[3.2.1]octan-4-ols promoted by thionyl chloride or Appel conditions

**DOI:** 10.3762/bjoc.20.74

**Published:** 2024-04-16

**Authors:** Martyn Jevric, Julian Klepp, Johannes Puschnig, Oscar Lamb, Christopher J Sumby, Ben W Greatrex

**Affiliations:** 1 Faculty of Medicine and Health, University of New England, Armidale, 2351, Australia; 2 Department of Chemistry and the Centre for Advanced Nanomaterials, The University of Adelaide, Adelaide, 5005, Australia

**Keywords:** bicyclic ring, cyrene, levoglucosenone, rearrangement, thionyl chloride

## Abstract

A skeletal rearrangement of a series of 6,8-dioxabicyclo[3.2.1]octan-4-ols has been developed using SOCl_2_ in the presence of pyridine. An oxygen migration from C5 to C4 was observed when the C4 alcohols were treated with SOCl_2_/pyridine, giving a 2-chloro-3,8-dioxabicyclo[3.2.1]octane ring-system via the chlorosulfite intermediate. Analogous allylic alcohols with endocyclic and exocyclic unsaturations underwent chlorination without rearrangement due to formation of allylic cations. The rearrangement was also demonstrated using Appel conditions, which gave similar results via the alkoxytriphenylphosphonium intermediate. Several reactions of the products were investigated to show the utility of the rearrangement.

## Introduction

The 6,8-dioxabicyclo[3.2.1]octane derivative levoglucosenone (**1**) is produced selectively when cellulose-containing materials, including lignocellulosic biomass, are acidified and pyrolysed [1–2]. Lab scale synthesis of this chiral material can be accomplished in a single step without special glassware [3], while large scale production of the reduction product cyrene (**2**) allows for its use as a chiral solvent [4]. This product is emerging as a promising platform chemical for the construction of chiral small molecules for pharmaceuticals [5–8], as a building block for catalysts and auxiliaries [9–11], and in materials applications [12–14]. New reactions will increase the number of accessible materials that can be made from this biorenewable starting material, particularly if novel approaches for modifying the connectivity of the bicyclic ring system can be developed.

**Figure 1:**
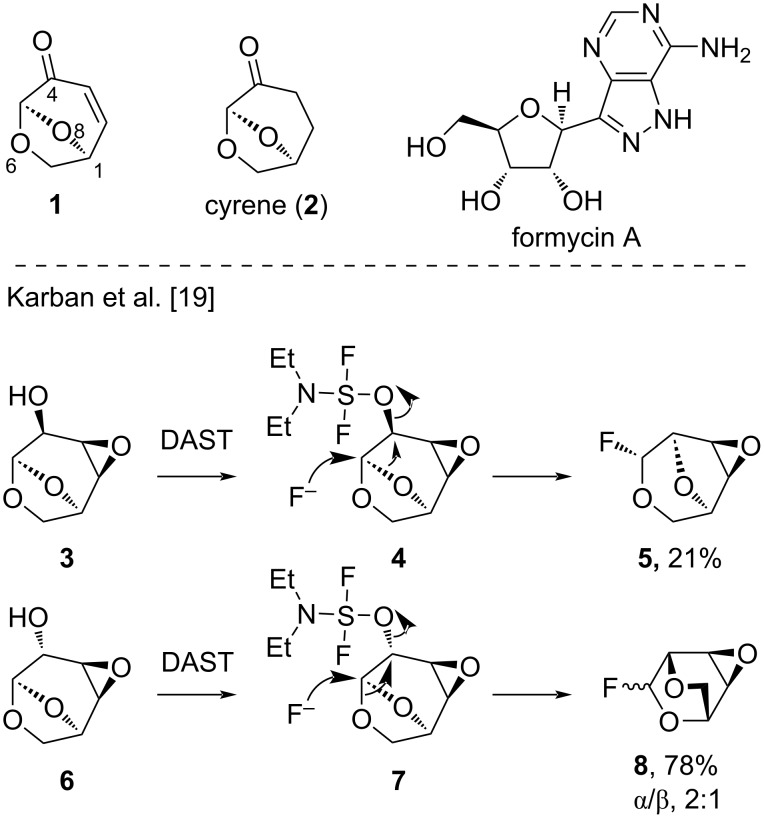
Previous work on migration reactions in 6,8-dioxabicyclooctan-4-ols [18].

The 6,8-dioxabicyclo[3.2.1]octane system is known to undergo a number of bond-cleavage reactions and rearrangements when modified at the 4-position [15–17]. Baillargeon and Reddy first reported rearrangements of 6,8-dioxabicyclo[3.2.1]octane derivatives promoted by diethylaminosulfur trifluoride (DAST) [18], and later Karban and co-workers reported a migration of oxygen from the acetal in **3** and **6** to the neighbouring C4-position (Figure 1) [19–20]. A variety of products were reported resulting from fluorination as well as the skeletal rearrangement, with the reaction outcome highly substrate-dependent. A key finding in this work was that the configuration of the alcohol at C4 determined the resultant ring system, as the σ* orbital is not accessible to external nucleophiles due to steric hindrance and the rigid conformation of the bicyclic ring system. When the C4–OH was equatorial, O8 migrated as it was aligned with the σ* orbital giving a 3,8-dioxabicyclo[3.2.1]octane, while O6 migrated when the C4–OH was axial leading to 2,4-dioxabicyclo[2.2.2]octanes. The formation of both anomers from the non-selective addition of fluoride suggested intermediates with oxocarbenium character. This work has recently been extended by Banwell and co-workers to include a set of Diels–Alder adducts of **1**, and similar results on the effect of configuration were observed [21].

During some recent attempts at the chlorination of the π-stacking chiral auxiliary **10a** using SOCl_2_ [9], we observed the migration of O8 resulting in the formation of anomeric chlorides analogous to the reports of Karban et al. (Scheme 2) [19]. We envisaged that these hexose-derived building blocks with a 2,5-anhydro bond such as **5** could be useful materials for the construction of *C*-nucleosides such as formycin A (Figure 1) [22–24]. The preparation of *C*-glycosides usually involves the creation of the glycosidic bond using an organometallic purine or pyrimidine derivative and an electrophilic furanose derivative [23,25]. This process can result in anomeric mixtures, so **5** has potential applications in targeted synthesis, as the configuration of the pseudo-anomeric centre matches the common biological ribosides. This prompted an investigation of the scope of the SOCl_2_-mediated rearrangement, with the aim of producing useful chiral materials for synthesis.

## Results and Discussion

**Scheme 1:**
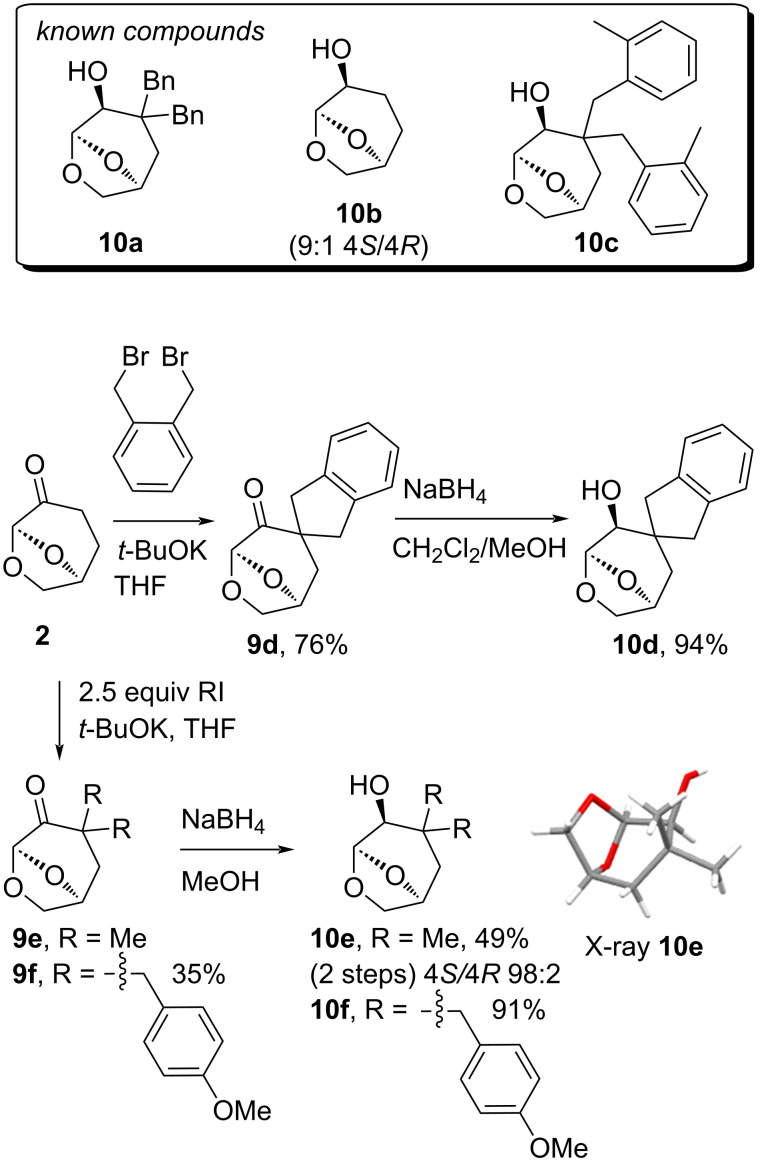
Structures for **10a**–**c**, preparation of **10d**–**f**, and X-ray structure of **10e**.

**Figure 2:**
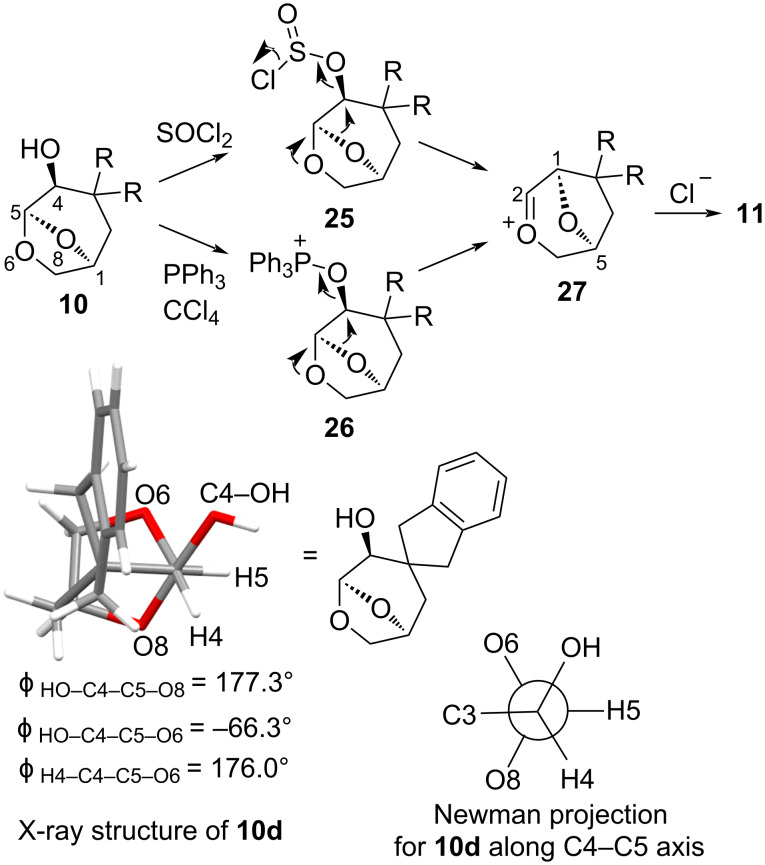
Mechanism for the rearrangement of **10**, and Newman projection and the X-ray structure of **10d** projected along the C4–C5 axis.

The set of bicyclic systems **10a**–**f** with a C4 alcohol were prepared starting with cyrene (**2**) by alkylation and then reduction using NaBH_4_ as per our previously published approach (Scheme 1) [9]. When α-alkylations are performed using **2**, the second alkylation step is faster than the first, meaning that only the dialkylated products are formed [16], and the reduction is highly selective with approach of the reductant from the *exo*-face. This process was used to prepare the known compounds **10a**–**c**, and the novel materials **10d**–**f** [9]. Thus, the reaction of *o*-dibromoxylene with cyrene gave the alcohol **10d** in 71% yield over two-steps through the spirocyclic ketone **9d**. Alkylation of **2** with methyl iodide gave an inseparable mixture of ketone **9e** and the *O*-alkylated enol ether by-product, which was then reduced using NaBH_4_ to give alcohol **10e** with 98:2 selectivity. Similarly, the reaction of 4-methoxybenzyl bromide and **2** gave ketone **9f** in 35% yield without chromatography, and when reduced resulted in only a single alcohol stereoisomer **10f** in 91% yield. The selectivity of the NaBH_4_ reduction was confirmed for both **10d** (see discussion in Supporting Information File 1) and **10e** by X-ray crystallography (Figure 2 and Scheme 1, respectively).

The oxygen-migration reaction giving **11a** was initially observed using the readily available chiral auxiliary **10a**, and following a survey of conditions and isolation protocols, a 90% yield was obtained when **10a** was heated in the presence of 2 equivalents of SOCl_2_ and 5 equivalents of pyridine in DCE. Flash chromatography of the chloroalkyl ether **11a** resulted in significant loss due to hydrolysis, although **11a** was sufficiently stable for filtration through a pad of silica. Applying these conditions and isolation protocols to all 3,3-disubstituted alcohols **10c**–**f** gave moderate to excellent yields of the rearrangement products **11c**–**f** as single stereoisomers. The reactions of alcohols **10b**,**d**,**e** also gave some sulfites **13b**,**d**,**e**, attributed to the reduced steric hindrance in the chlorosulfite intermediate allowing for the second alcohol to approach prior to rearrangement. The isolation of these materials suggested that dialkyl sulfite formation could compete with the rearrangement if the neighbouring groups were small, and attempts to prevent the formation of **13d** by slow addition of alcohol **10d** to a solution of SOCl_2_/pyridine in DCE reduced the yield of **11d** to 14%. Heating sulfite **13d** with tetrabutylammonium chloride led only to hydrolysis back to **10d** without rearrangement, indicating that these sulfites were not intermediates in the reaction manifold. Furthermore, the isolation of some starting alcohols in the reactions of **10b** and **10d** following chromatography was attributed to hydrolysis of the corresponding dialkyl sulfite **13b** and **13d** on silica, a process that can be acid or base-catalysed [26]. The unsubstituted derivative **11b** was difficult to isolate in good yields as multiple products were formed giving complex reaction mixtures. The product **11b** was consistently contaminated with a second inseparable product tentatively assigned as **14**, which is the expected product of chlorination without skeletal rearrangement (vide infra). Inclusion of the soft-nucleophile allyltrimethylsilane in the reaction of **10b** to trap potential oxocarbenium ion intermediates also resulted in a complex mixture.

**Scheme 2:**
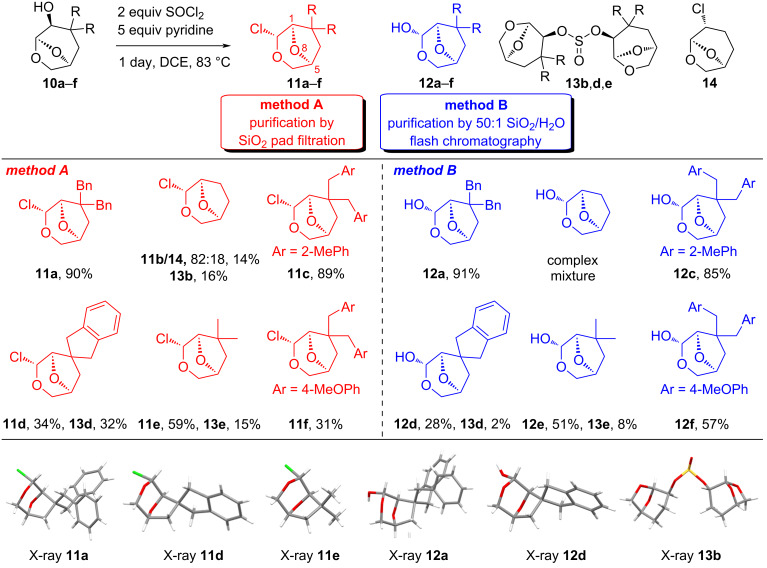
Rearrangement reactions for **10a**–**f** promoted by SOCl_2_.

During the isolation of the chloroalkyl ether products **11a**–**f**, it was apparent that hydrolysis occurred during chromatography, and so an alternate method was developed to generate a single product by promoting the formation of the hemiacetal series **12a**–**f**. Following the rearrangement reaction, chromatography of the chlorides using silica with 2% water added led to the isolation of **12a**,**c**–**f** in good yield, with the *exo*-hemiacetals favoured due to steric interactions between the substituents and alcohol, while the attempted preparation of **12b** led only to complex mixtures (Scheme 2).

The products of the reactions were characterised by 1D and 2D NMR, and X-ray crystallography of members from each class was used to confirm assignments. In the ^1^H NMR spectra of the chlorides **11a**–**f**, a downfield shift for the anomeric methine was observed to δ ≈6 ppm from δ ≈5.4 ppm in the starting materials **10a**–**f**. There was also a characteristic change in the appearance of the oxymethylene bridge spin system, with the products exhibiting much larger differences in the chemical shifts for the geminal protons relative to those observed in the starting material. For example, in the ^1^H NMR spectrum of **11e**, the H4/H4′ resonances have a difference of 0.94 ppm, while in **10e**, the progenitor H7/H7′ methylene resonances are separated by 0.21 ppm. In the ^13^C NMR spectrum of **11a**–**f**, a ≈10 ppm upfield shift for the chloroacetal was seen to δ ≈92 ppm from the C5 acetal present in the starting materials. Only a single diastereomer was formed for all 3,3-disubstituted rearrangement products due to the hindrance on the *endo*-face, with the X-ray crystal structures for **11a** and **11b** allowing for the unambiguous assignment of configuration. The chlorinated product **14** contaminating **11b** exhibited a ^1^H NMR spectrum similar to the starting material **10b**, except that the resonance for the H4 methine was shifted from δ 3.60 in **10b** to δ 3.90 ppm in **14**. The methine had a correlation to a resonance at δ_C_ 55.1 ppm in the 2D HSQC spectra consistent with an attached chloride. In the ^1^H NMR spectra for the hemiacetals **12a**,**c**–**f**, an upfield shift for the anomeric centre of ≈1.1 ppm was observed relative to the chlorides. There was also evidence of open chain aldehydes present in solution (≈5%) with a doublet at δ 9.6 ppm, with the configuration of the hemiacetal centre confirmed in the solid state by X-ray crystallography on **12a** and **12d**. The ^1^H and ^13^C NMR spectra for **13b**,**d**,**e** were similar to the starting materials, except that the resonances were doubled due to the diastereotopic ring systems. The different environments were mainly evident in the chemical shifts for H4/H4′ (Δδ 0.06 ppm) and H5/H5′ (Δδ 0.03 ppm), with other resonances only showing broadening due to their remote relationship with the sulfite group. The structures of the dialkyl sulfites were confirmed using X-ray crystallography for **13b**, which clearly demonstrated the lack of symmetry across the molecule.

**Scheme 3:**
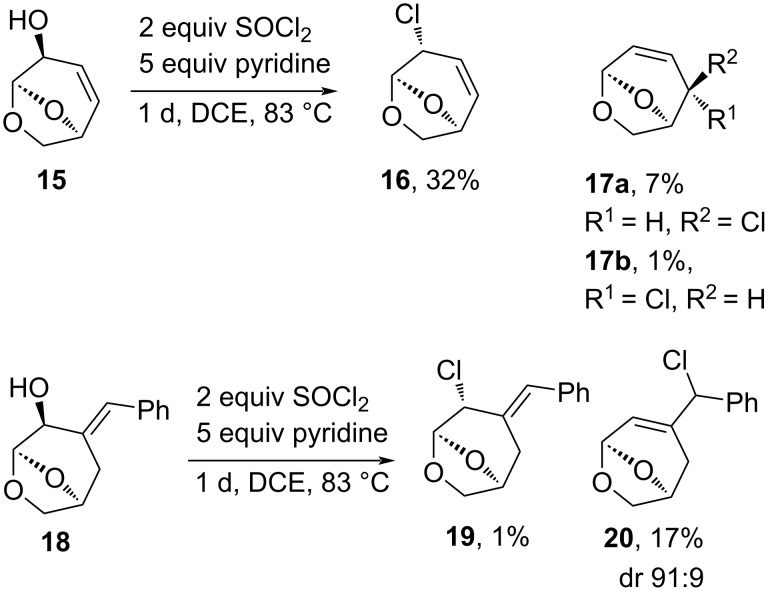
Reactions of allylic alcohols **15** and **18** with SOCl_2_.

To further examine the scope of the reaction, allylic alcohols **15** and **18** were subjected to the optimised reaction conditions [9,27]. The reaction of **15** with an endocyclic olefin led to a series of separable allylic chlorides **16** and **17a**,**b**, from direct displacement or transposition of the allylic system (Scheme 3). This substrate has previously been examined in the reaction with SOCl_2_ by Matsumoto et al. in THF and CH_2_Cl_2_ and similar results were obtained [28]. When **18** containing an exocyclic alkene was subjected to the reaction conditions, a mixture of benzylic chlorides (**20**) was formed in low yields, and trace amounts of the allylic chloride **19** was also isolated, the materials differentiated on the basis of the coupling of the acetal H5 with the respective vicinal proton. These results suggested that the formation of the allylic cation occurred readily from alcohols **15** and **18**; however, the transition states leading to the rearrangement products were inaccessible and so only chloride addition occurred.

**Scheme 4:**
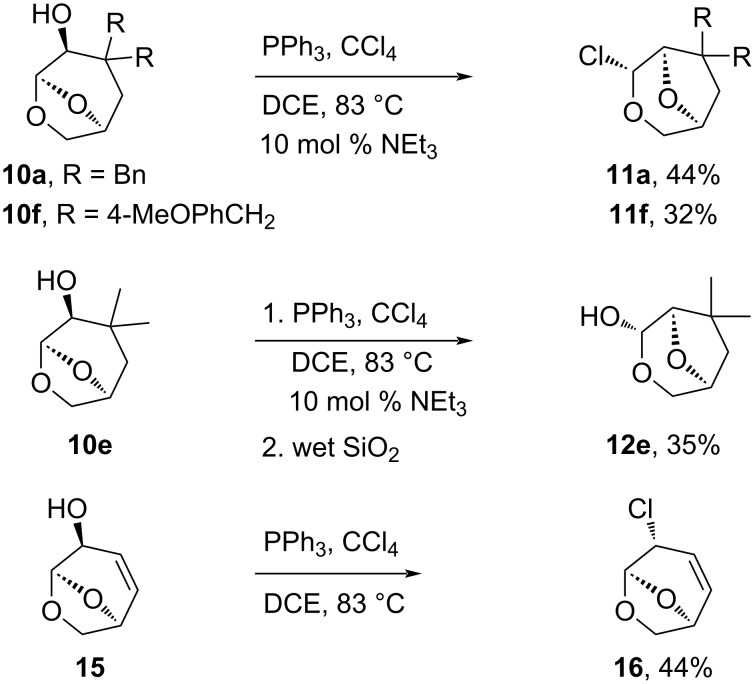
Appel reactions of dioxabicyclo[3.2.1]octan-4-ols **10a**,**e**,**f** and **15**.

The generation of the rearrangement products from the reaction with SOCl_2_, and the previous work with DAST, suggested that good leaving groups were required to drive the migration reaction. A variety of processes are known for deoxyhalogenation, and it was thought that alternatives to SOCl_2_ could also be used to promote the oxygen migration. When Appel conditions (PPh_3_, CCl_4_) for the deoxychlorination were examined [29], the rearrangement products were observed as the major components in the reaction mixture (Scheme 4). For the reaction of **10a**, the starting material was consumed within 1 hour (NMR) to give an intermediate assigned as the alkoxytriphenylphosphonium chloride (**26**, R = Bn), which then slowly rearranged over 24 hours at 83 °C in DCE, eliminating triphenylphosphine oxide (Figure 2). A single ion was observed in the ESI mass spectrum for the intermediate at *m/z* 571.1 corresponding to the [M + PPh_3_ − H]^+^, and in the ^1^H NMR, the H4 adjacent to the oxyphosphonium group was observed at δ 4.44 ppm, shifted downfield relative to the starting alcohol **10a** along with resonances for the phenyl groups. The isolation of the products using the Appel conditions was more challenging than for the reactions with SOCl_2_ due to the difficulties separating the products from the byproduct triphenylphosphine oxide, necessitating chromatography which resulted in some hydrolysis. There are a number of catalytic activation strategies for Appel or Mitsunobu reactions such as those described by the Denton group [30], and Rutjes and co-workers [31], and while these may prove useful in future studies, they were not examined in this work.

**Scheme 5:**
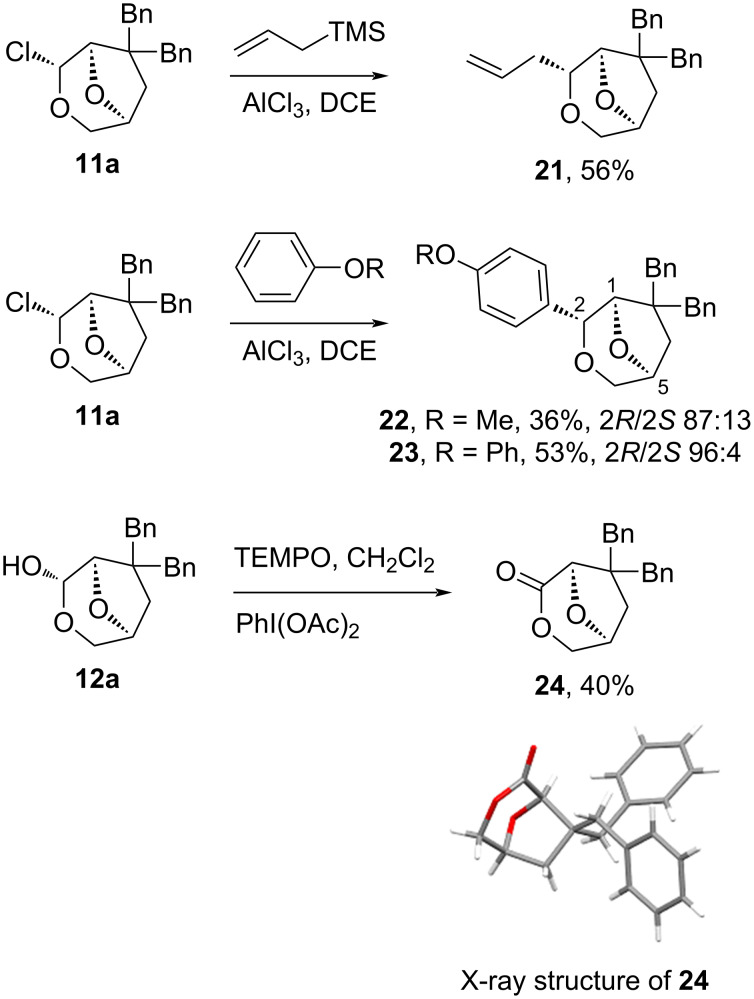
Some transformations for the skeletal rearrangement products **11a** and **12a** and X-ray structure for **24**.

To investigate the potential uses for the rearrangement products, a series of reactions on the chloroalkyl ethers **11a** and **12a** was performed (Scheme 5). The reaction of **11a** with allyltrimethylsilane catalysed by aluminium chloride resulted in the displacement of the chloro substituent with the allyl group, affording **21** in good yield. Electrophilic aromatic substitution reactions at the chloroalkyl ether site were possible when promoted by aluminium chloride, with anisole and diphenyl ether giving addition products **22** and **23** containing small amounts of the C2 epimers. Oxidation of the hemiacetal **12a** gave a moderate and unoptimised yield of 40% for lactone **24**.

The probable mechanism for the transformation with SOCl_2_ and under Appel conditions is shown in Figure 2. The reaction of alcohol **10** with the electrophiles gives the chlorosulfite **25** or the alkoxytriphenylphosphonium chloride **26**, respectively. With heating, SO_2_ or triphenylphosphine oxide is extruded with a concerted migration of the neighbouring O8 leading to an oxocarbenium ion **27**, which is then trapped with chloride giving the observed products. The crystal structure for the precursor alcohol **10d** is shown projected along the C4–C5 axis, which demonstrates a 177.3° dihedral angle for the HO–C4–C5–O8 group, aligning O8 antiperiplanar and positioned to migrate during the reaction. The relationship between H4 and O6 is similarly antiperiplanar, with a H4–C4–C5–O6 dihedral angle of 176°, explaining the preference for the different skeletal rearrangements in the two possible configurations at C4 in these rigid ring systems [19,21]. The involvement of the ring-oxygen in nucleophilic displacement reactions in 1,6-anhydroglucose derivatives has also been invoked to explain the observed retention of configuration, showing that substantial interactions between the oxygens of the ring and centres on the larger bridge are possible [32]. The specificity of the rearrangement also eliminates the possibility of an intermediate secondary C4 carbocation, and requires a concerted bond migration. This is a mechanistic difference to the related 1,2-oxygen migration reactions of spiroacetals that involve alkoxy intermediates reported by Suarez and co-workers [33–34]. The presence of oxocarbenium ion **27** is inferred due to the formation of two diastereomers in Karban’s previous work, which suggests a stepwise migration of oxygen from C5 to C4, followed by addition of the halogen nucleophile. Furthermore, if the halogen was involved in the transition state via the σ* orbital (avoiding intermediate **27**), the opposite configuration would result at C2 in the products. The single diastereomers isolated in the current work are attributed to the differences in sterics on the faces of the oxocarbenium ion **27**, caused by the substitution on the bicyclic ring system.

## Conclusion

The formation of anomeric chlorides due to bond migrations in the dioxabicyclo[3.2.1]octanol ring system has been described for the first time. The work builds upon the findings of the groups of Karban and Banwell, who described this type of ring transformation using DAST, with two new reagents for promoting the rearrangement reaction. This work adds to the growing set of transformations that are known for levoglucosenone, cyrene and their derivatives, generating a unique set of bicyclic building blocks.

## Supporting Information

Supporting information includes detailed experimental details and characterisation data, X-ray crystallography, and copies of ^1^H and ^13^C spectra for new compounds.

File 1 Experimental details, X-ray crystallography and spectra.

File 2 ^1^H and ^13^C NMR FIDs, HRMS spectra for all new compounds.

## Data Availability

All data that supports the findings of this study is available in the published article and/or the supporting information to this article.
