# Supplementary material for: Skeletal rearrangement of 6,8-dioxabicyclo[3.2.1]octan-4-ols promoted by thionyl chloride or Appel conditions
Source: Beilstein J Org Chem. 2024 Apr 16;20:823–9. doi: 10.3762/bjoc.20.74 (PMC11035982; doi:10.3762/bjoc.20.74)
Supplement: File 1 — Experimental details, X-ray crystallography and spectra. [file Beilstein_J_Org_Chem-20-823-s001.pdf]

BEILSTEIN JOURNAL OF ORGANIC CHEMISTRY

Supporting Information

for

Skeletal rearrangement of 6,8-dioxabicyclo[3.2.1]octan-4-ols promoted by thionyl chloride or Appel conditions

Martyn Jevric, Julian Klepp, Johannes Puschnig, Oscar Lamb, Christopher J. Sumby and Ben W. Greatrex

Beilstein J. Org. Chem. **2024,** *20,* 823–829. doi:10.3762/bjoc.20.74

Experimental details, X-ray crystallography and spectra

License and Terms: This is a supporting information file under the terms of the Creative Commons Attribution License (https://creativecommons.org/licenses/by/4.0). Please note that the reuse, redistribution and reproduction in particular requires that the author(s) and source are credited and that individual graphics may be subject to special legal provisions.
The license is subject to the *Beilstein Journal of Organic Chemistry* terms and conditions: (https://www.beilstein-journals.org/bjoc/terms)

Table of contents

Table of contents S1

General experimental S2

Preparation of alcohols **10d**–**f** S2

Representative procedure for the rearrangement reaction to give **11a**–**f** S5

Representative procedure for the formation of alcohols **12a**,**c**–**f**. S8

Reactions of alcohol **18** with $SOCl_2$ S11

Representative procedure for the rearrangement using Appel conditions. S11

Single crystal X-ray crystallography S15

Copies of ^1H and $^{13}C\{^1H\}$ NMR spectra S24

References S82

General experimental

Unless otherwise stated, common chemicals and solvents (HPLC-grade or reagent-grade quality) were purchased from commercial sources and used without further purification. $SOCl_2$ was distilled prior to use. A hot plate magnetic stirrer and PEG bath was used as the heating source in all reactions requiring heat, and all reactions were performed under an atmosphere of N_2. Aluminum plates coated with a 0.2 mm-thick layer of silica gel 60 F254 were used for thin-layer chromatography (TLC) analysis, and flash column chromatography purification was carried out using silica gel 60 (230–400 mesh). Water treated silica was prepared by adding the specified volume of water to the silica gel with stirring until the mixture was thoroughly combined. A slurry was prepared with the eluent, and a glass column packed with the wet silica, and manual flash chromatography techniques used. Proton (^{1}H) spectra were recorded at 25 °C in a 500 MHz spectrometer and proton-decoupled carbon ($^{13}C\{^{1}H\}$) NMR spectra were recorded at 125 MHz using the deuterated solvent as an internal deuterium lock. ^{1}H NMR spectra were referenced to $CDCl_3$ (δ 7.26 ppm) and $^{13}C\{^{1}H\}$ NMR spectra recorded in $CDCl_3$ were referenced to $CDCl_3$ (δ 77.0 ppm). High-resolution mass spectra were recorded using either a Waters Xevo time of flight or a Thermo Fisher Fusion Orbitrap mass spectrometer equipped with either an electrospray ionisation source or an atmospheric pressure chemical ionisation source, in positive or negative ionisation mode to match preferred compound ionisation properties. All chemical formula were identified with matches <5ppm. Alcohols **10a** [1], **10b** [2], and **10c** [1] were prepared according to previously published procedures.

Preparation of alcohols 10d–f

(1*S*,5*R*)-1',3'-Dihydro-6,8-dioxaspiro[bicyclo[3.2.1]octane-3,2'-inden]-4-one (9d). To a stirred solution of cyrene (**2**, 11.2 g, 87 mmol), dibromoxylene (25.0 g, 105 mmol) and tetrabutylammonium iodide (3.2 g, 8.7 mmol) in dry THF (200 mL) cooled to 0 °C was added *t*-BuOK (24.3 g, 218 mmol). After stirring for 4 h at 0 °C, the reaction was quenched by adding 1.0 M HCl (250 mL). The mixture was extracted with EtOAc (4 × 50 mL), the combined organic layers were dried over $MgSO_4$ and concentrated under reduced pressure, and then the residue was purified by flash column chromatography on silica (200 g) with toluene (750 mL) to give **9d** (15.2 g, 76%) as an orange wax; $[\alpha]_D^{25}$ −174 (*c* 1.0, CH_2Cl_2); IR: ν_{max} 2914, 1725, 1485, 1118, 1098, 747 cm^{-1}; ^{1}H NMR (500 MHz, $CDCl_3$) δ 7.21–7.10 (m, 4H), 5.25 (s, 1H), 4.75 (app. t, *J* = 4.8 Hz, 1H), 4.18 (d, *J* = 7.0 Hz, 1H), 3.91 (ddd, *J* = 7.0, 4.8, 1.6 Hz, 1H), 3.74 (d, *J* = 15.9 Hz, 1H), 3.50 (d, *J* = 16.2 Hz, 1H), 3.18 (d, *J* = 15.9 Hz, 1H), 2.70 (d, *J* = 16.2 Hz, 1H), 2.37 (ddd, *J* = 14.6, 4.8, 1.6 Hz, 1H), 2.18 (d, *J* = 14.6 Hz, 1H); $^{13}C\{^{1}H\}$ NMR (125 MHz, $CDCl_3$) δ 203.1, 141.0, 139.9, 127.2, 126.9, 124.5, 124.4, 101.5, 73.9, 68.0, 51.7, 46.7, 45.1, 42.8; HRMS (ESI-TOF) *m/z*: $[M+Na]^+$ calcd for $C_{14}H_{14}O_3Na$: 253.0835; found: 253.0832.

(1*S*,5*R*)-3,3-Bis(4-methoxybenzyl)-6,8-dioxabicyclo[3.2.1]octan-4-one (9f). To a solution of cyrene (**2**, 3.35 g, 26.1 mmol), 1-(bromomethyl)-4-methoxybenzene (9.00 g, 57.5 mmol) and potassium iodide (8.67 g, 52.3 mmol) in THF (100 mL) cooled to 0 °C was added *t*-BuOK (6.45 g, 57.5 mmol) in portions over 15 min. The mixture was allowed to warm to ambient temperature, stirred overnight and then quenched with 1 M HCl (200 mL). The resulting mixture was extracted with Et_2O (3 × 100 mL), then the combined organic extracts were washed with satd. $NaHCO_3$ (150 mL), dried over Na_2SO_4 and the volatiles removed under reduced pressure. The residue was dissolved in boiling MeOH (150 mL), then cooled to ambient temperature and the precipitated solid collected and washed with cold MeOH (50 mL). The mother liquor was then concentrated under reduced pressure, and the residue recrystallised from boiling MeOH (100 mL). The crystals were collected and washed with cold MeOH (50 mL) to furnish **9f** as a colourless solid (3.34 g, combined yield 35%); mp 134–135 °C; $[\alpha]_D^{21}$ −17 (*c* 1.0, CH_2Cl_2); IR: ν_{max} 2959, 1723, 1610, 1510, 1245, 1177 cm^{-1}; ^{1}H NMR (500 MHz, $CDCl_3$) δ 7.04–6.97 (m, 4H), 6.84–6.78 (m, 4H), 5.03 (s, 1H), 4.55 (dd, *J* = 6.4, 5.2 Hz, 1H), 3.79 (s, 3H), 3.78 (s, 3H), 3.56 (ddd, *J* = 7.2, 5.2, 1.1 Hz, 1H), 3.22–3.13 (m, 3H), 2.67 (d, *J* = 13.5 Hz, 1H), 2.50 (d, *J* = 13.5 Hz, 1H), 2.37 (ddd, *J* = 14.6, 6.4, 1.1 Hz, 1H), 1.71 (d, *J* = 14.6 Hz, 1H); $^{13}C\{^1H\}$ NMR (125 MHz, $CDCl_3$) δ 204.6, 158.7, 158.5, 132.2, 132.1, 129.0, 128.9, 113.8, 113.7, 100.3, 72.9, 68.2, 55.4, 55.3, 50.0, 45.7, 45.0, 32.2; HRMS (ESI-TOF) *m/z*: $[M+Na]^+$ calcd for $C_{22}H_{24}O_5Na$: 391.1516; found: 391.1515.

cm^{-1}

(1*S*,4*S*,5*R*)-1',3'-Dihydro-6,8-dioxaspiro[bicyclo[3.2.1]octane-3,2'-inden]-4-ol (10d). A solution of ketone **9d** (15.2 g, 66 mmol) in CH_2Cl_2/MeOH (1:2, 300 mL) was cooled to −15 °C and $NaBH_4$ (1.7 g, 46.2 mmol) was added in portions over 15 minutes. The mixture was kept at −15 °C for 3 h and concentrated under reduced pressure and CH_2Cl_2 (250 mL) and 1.0 M HCl (250 mL) were added. Following separation, the aqueous layer was extracted with CH_2Cl_2 (2 × 100 mL) and the combined organic layers were dried over $MgSO_4$ and concentrated. The residue was recrystallized with EtOAc/petroleum spirit to give the **10d** (14.4 g, 94%) as pale-yellow crystals suitable for X-ray crystallography; mp 92–94 °C; $[\alpha]_D^{25}$ −164 (*c* 1.0, CH_2Cl_2); IR: ν_{max} 2924, 1711, 1363, 1220, 1078, 924 cm^{-1}; ^{1}H NMR (500 MHz, $CDCl_3$) δ 7.20–7.09 (m, 4H), 5.38 (d, *J* = 1.6 Hz, 1H), 4.53 (ddd, *J* = 4.8, 4.3, 1.4 Hz, 1H), 4.10 (d, *J* = 7.3 Hz, 1H), 3.78 (ddd, *J* = 7.3, 4.8, 1.4 Hz, 1H), 3.69 (dd, *J* = 7.6, 1.6 Hz, 1H), 3.64 (d, *J* = 16.3 Hz, 1H), 3.18 (d, *J* = 16.3 Hz, 1H), 2.85 (d, *J* = 16.3 Hz, 1H), 2.84 (d, *J* = 16.3 Hz, 1H), 2.05 (ddd, *J* = 14.6, 4.3, 1.4 Hz, 1H), 1.92 (dd, *J* = 14.6, 1.4 Hz, 1H), 1.67 (d, *J* = 7.6 Hz, OH); $^{13}C\{^1H\}$ NMR (125 MHz, $CDCl_3$) δ 143.1, 140.9, 126.7, 126.6, 124.4, 124.2, 102.5, 76.5, 74.0, 67.9, 48.7, 46.8, 40.7, 39.8; HRMS (ESI-TOF) *m/z*: $[M+H]^+$ calcd for $C_{14}H_{17}O_3$: 233.1172; found: 233.1169.

(1*S*,4*S*,5*R*)-3,3-Dimethyl-6,8-dioxabicyclo[3.2.1]octan-4-ol (10e). To a magnetically stirred solution of cyrene (**2**, 16.27 g, 127 mmol) and methyl iodide (25 mL, 400 mmol) in THF (200 mL) cooled to 0 °C using was added *t*-BuOK (39.79 g, 355 mmol). The resulting solution was stirred vigorously overnight, and allowed to come to ambient temperature. The reaction mixture was poured into a magnetically stirred beaker containing saturated aqueous $NaHCO_3$ (200 mL), and the mixture concentrated to a volume of 200 mL under reduced pressure in a ventilated fumehood to remove any residual methyl iodide. The aqueous mixture was extracted with Et_2O (3 × 200 mL), the combined organic phases were dried (Na_2SO_4), filtered and volatiles were removed under reduced pressure to give **9e** (11.27 g, 57%) as a colourless oil, which was used directly in the following step without further processing; ^{1}H NMR (500 MHz, $CDCl_3$) δ 5.15 (s, 1H), 4.71 (dddd, *J* = 5.5, 5.2, 1.5, 0.5 Hz, 1H), 4.02 (dd, *J* = 7.4 0.8 Hz, 1H), 3.84 (ddd, *J* = 7.4, 5.2, 0.5 Hz, 1H), 2.21 (ddd, *J* = 14.4, 5.5, 1.7 Hz, 1H), 1.86 (br d, *J* = 14.4 Hz, 1H), 1.32 (s, 3H), 1.16 (s, 3H). To a stirred solution of **9e** (4.62 g, 29.6 mmol) in methanol (50 mL) cooled using an ice/water bath was added $NaBH_4$ (0.57 g, mmol) in portions over 30 min. The resulting solution was stirred overnight at room temperature, then saturated aqueous $NaHCO_3$ (100 mL) was added and the mixture was extracted with EtOAc (3 × 100 mL). The combined organic extracts were dried over Na_2SO_4, filtered, and the volatiles were removed under reduced pressure. The residue was purified by dry flash column chromatography (gradient elution of EtOAc/ petroleum spirit 1:9 to 2:3) to give **10e** (4.02 g, 86%) as a white solid (mix of diastereomers, 98:2). Small amounts of isomerically pure **10e** and crystals suitable for X-ray crystallography were obtained by vacuum sublimation; R_f 0.4 (EtOAc/petroleum spirit, 1:3); mp 43–45 °C; $[\alpha]_D^{25}$ −138 (*c* 0.50, CH_2Cl_2); IR: ν_{max} 3453, 3001, 2953, 2912, 2897, 2873 cm^{-1}; ^{1}H NMR (500 MHz, $CDCl_3$) δ 5.32 (d, *J* = 1.9 Hz, 1H), 4.46 (ddd, *J* = 4.7, 4.7, 1.6 Hz, 1H), 3.93 (d, *J* = 7.4 Hz, 1H), 3.72 (ddd, *J* = 7.4, 5.0, 1.5 Hz, 1H), 3.34 (dd, *J* = 11.1, 1.9 Hz, 1H), 1.88 (ddd, *J* = 14.5, 4.1, 1.5 Hz, 1H), 1.75 (d, *J* = 11.1 Hz, 1H), 1.62 (dd, *J* = 14.5, 1.5 Hz, 1H), 1.15 (s, 3H), 1.05 (s, 3H); ^{13}C{^{1}H} NMR (125 MHz, $CDCl_3$) δ 102.4, 76.3, 73.8, 67.7, 42.4, 34.1, 32.6, 23.7; HRMS (ESI-TOF) *m/z*: [M+Na]$^+$ calcd for $C_8H_{14}NaO_3$: 181.0835; found: 181.0842.

(1*S*,4*S*,5*R*)-3,3-Bis(4-methoxybenzyl)-6,8-dioxabicyclo[3.2.1]octan-4-ol (10f). To a stirred solution of **9f** (2.00 g, 5.43 mmol) in CH_2Cl_2/MeOH (1:1, 20 mL) cooled to 0 °C was added $NaBH_4$ (205 mg, 5.43 mmol). After 1 h, the ice bath was removed and then the mixture was allowed to stir at ambient temperature overnight. The mixture was concentrated under reduced pressure, the residue suspended in 1 M HCl (50 mL), and then extracted with CH_2Cl_2 (4 × 50 mL). The combined organic layers were washed with brine (50 mL), dried over Na_2SO_4 and the solvent removed under reduced pressure. The residue was purified via flash column chromatography (petroleum spirit/EtOAc, 2:1) to yield **10f** as a colourless solid (1.83 g, 91%); R_f 0.3 (petroleum spirit/EtOAc, 2:1); mp 54–55 °C; $[\alpha]_D^{21}$ −51.3 (*c* 1.0, CH_2Cl_2); IR: ν_{max} 3461, 2958, 1610, 1510, 1250 cm^{-1}; ^{1}H NMR (500 MHz, $CDCl_3$) δ 7.19–7.15 (m,

2H), 7.01–6.96 (m, 2H), 6.90–6.85 (m, 2H), 6.83–6.78 (m, 2H), 5.33 (d, *J* = 2.0 Hz, 1H), 4.42–4.39 (m, 1H), 4.24 (d, *J* = 7.5 Hz, 1H), 3.82 (s, 3H), 3.82–3.78 (m, 1H), 3.78 (s, 3H), 3.68 (d, *J* = 2.0 Hz, 1H), 3.09 (d, *J* = 14.1 Hz, 1H), 2.93 (d, *J* = 14.1 Hz, 1H), 2.78 (d, *J* = 13.6 Hz, 1H), 2.31 (d, *J* = 13.6 Hz, 1H), 1.77 (br s, 1H), 171–1.67 (m, 2H); $^{13}C\{^1H\}$ NMR (125 MHz, $CDCl_3$) δ 158.38, 158.32, 132.7, 132.4, 129.9, 129.6, 113.8, 113.7, 102.9, 73.6, 71.6, 67.9, 55.23, 55.19, 43.4, 41.3, 38.3, 30.9; HRMS (ESI-TOF) *m/z*: $[M+Na]^+$ calcd for $C_{22}H_{26}O_5Na$: 393.1672; found: 393.1690.

Representative procedure for the rearrangement reaction to give 11a–f

(1*R*,2*R*,5*S*)-7,7-Dibenzyl-2-chloro-3,8-dioxabicyclo[3.2.1]octane (11a). To a stirred solution of **10a** (0.94 g, 3.0 mmol) in DCE (50 mL) was added $SOCl_2$ (0.45 mL, 6.2 mmol) and pyridine (1.2 mL, 15 mmol) and the resulting solution was heated to boiling under reflux for 24 h. The solution was allowed to cool, then chilled in an ice bath and poured onto ice cold 2 M HCl (50 mL). The layers were separated and the organic phase was washed with saturated aqueous $NaHCO_3$ (50 mL), dried using Na_2SO_4, and then the solution was passed rapidly through a SiO_2 pad, washing with CH_2Cl_2 and the solvent removed to afford **11a** (885 mg, 90%) as a white solid. Crystals suitable for X-ray crystallography were prepared by slow evaporation from an *i*-Pr_2O solution; R_f 0.6 (EtOAc/petroleum spirit 1:9, partial hydrolysis); mp 136–138 °C; $[\alpha]_D^{25}$ −134 (*c* 1.0, CH_2Cl_2); IR: ν_{max} 3085, 3060, 3028, 2964, 2927, 2874, 2855, 1602, 1583, 1495 cm^{-1}; ^1H NMR (500 MHz, $CDCl_3$) δ 7.40–7.35 (m, 2H), 7.32–7.19 (m, 6H), 7.03–6.99 (m, 2H), 6.05 (s, 1H), 4.42 (d, *J* = 7.6 Hz, 1H), 4.34 (dd, *J* = 11.3, 1.2 Hz, 1H), 4.03 (s, 1H), 3.42 (d, *J* = 11.3 Hz, 1H), 3.15 (d, *J* = 15.0 Hz, 1H), 2.92 (d, *J* = 15.0 Hz, 1H), 2.86 (d, *J* = 13.5 Hz, 1H), 2.79 (d, *J* = 13.5 Hz, 1H), 2.24 (dd, *J* =12.4, 7.6 Hz, 1H), 2.14 (br d, *J* = 12.4 Hz, 1H); $^{13}C\{^1H\}$ NMR (125 MHz, $CDCl_3$) δ 138.6, 138.1, 130.7, 129.84, 129.82, 128.3, 126.62, 126.58, 91.8, 83.9, 75.3, 67.3, 50.5, 43.1, 39.7, 37.9; HRMS (ESI-TOF) *m/z*: $[M+Na]^+$ calcd for $C_{20}H_{21}O_2ClNa$: 351.1122; found: 351.1132.

(1*R*,2*R*,5*S*)-2-Chloro-3,8-dioxabicyclo[3.2.1]octane (11b). The reaction of **10b** (0.39 g, 3.0 mmol) in DCE (30 mL) with $SOCl_2$ (0.45 mL, 6.2 mmol) and pyridine (1.2 mL, 15 mmol) as for **11a** gave **11b** (64 mg, 14%) as a yellowish oil contaminated with **14** (**11b**/**14** 82:18). The SiO_2 pad was then washed with a further portion of EtOAc, and the solvent was removed under reduced pressure. The residue was purified by flash column chromatography (EtOAc/petroleum spirit, 1:1) to afford **13b** (75 mg, 16%) as an off-white wax; R_f 0.4 (EtOAc/petroleum spirit, 1:9); ^1H NMR (500 MHz, $CDCl_3$) δ 5.68 (ddd, *J* = 1.0, 1.0, 1.0 Hz, 1H), 4.33 (br s, 1H), 4.29 (br d, *J* = 11.3 Hz, 1H), 4.26–4.24 (br m, 1H), 3.40 (ddd, *J* = 11.2, 1.0, 1.0 Hz, 1H), 2.05–1.98 (m, 4H); $^{13}C\{^1H\}$ NMR (125 MHz, $CDCl_3$) δ 93.3, 78.9, 74.8, 67.2, 28.1, 26.0; ESI-MS *m/z* 171.0 $[M+Na]^+$; No molecular ions were observed when analysed by MS, and only species corresponding to hydrolysed material were seen. **(1*S*,4*R*,5*R*)-4-chloro-6,8-dioxabicyclo[3.2.1]octane (14)**. ^1H NMR (500 MHz, $CDCl_3$) δ 5.39 (br s, 1H), 4.57–4.53 (m, 1H), 3.94

(dd, J = 7.2, 0.7 Hz, 1H), 3.88–3.86 (m, 1H), 3.83 (ddd, J = 7.2, 5.1, 1.2 Hz, 1H), 2.36–2.22 (m, 2H), 1.89–1.85 (m, 1H), 1.45–1.41 (m, 1H); $^{13}C\{^{1}H\}$ NMR (125 MHz, $CDCl_3$) δ 101.6, 73.4, 67.3, 54.8, 24.4, 24.3.

Di((1*S*,4*S*,5*R*)-6,8-dioxabicyclo[3.2.1]octan-4-yl) sulfite (13b). Crystals suitable for X-ray crystallography were prepared by slow evaporation from CH_2Cl_2/*n*-heptane solution; R_f 0.4 (EtOAc/petroleum spirit 1:1); mp 111–114 °C (CH_2Cl_2/*n*-heptane); $[\alpha]_D^{16}$ −141 (*c* 0.50, CH_2Cl_2); IR: ν_{max} 2957, 2896, 2858 cm^{-1}; ^{1}H NMR (500 MHz, $CDCl_3$) δ 5.44 (br s, 1H), 5.39 (br s, 1H), 4.55–4.47 (m, 4H), 3.92–3.89 (m, 2H), 3.85–3.80 (m, 2H), 2.04–1.82 (m, 6H), 1.69–1.60 (m, 2H); $^{13}C\{^{1}H\}$ NMR (125 MHz, $CDCl_3$) δ 101.2, 100.9, 72.93, 72.90, 71.1, 70.8, 68.50, 68.47, 28.13, 28.11, 23.7, 23.62; HRMS (ESI-TOF) *m/z*: $[M+Na]^+$ calcd for $C_{12}H_{18}NaO_7S$: 329.0665; found: 329.0666.

(1*R*,2*R*,5*S*)-2-Chloro-7,7-bis(2-methylbenzyl)-3,8-dioxabicyclo[3.2.1]octane (11c). The reaction of **10c** (0.69 g, 2.0 mmol) in DCE (30 mL) with $SOCl_2$ (0.30 mL, 4.1 mmol) and pyridine (0.80 mL, 10 mmol) as for **11a** gave **11c** (630 mg, 89%) as a white solid; R_f 0.6 (EtOAc/petroleum spirit 1:9, partial hydrolysis); mp 144–149 °C; $[\alpha]_D^{25}$ −101 (*c* 1.0, CH_2Cl_2); IR: ν_{max} 3103, 3062, 3020, 2966, 2874, 1603, 1490 cm^{-1}; ^{1}H NMR (500 MHz, $CDCl_3$) δ 7.32 (d, J = 7.7 Hz, 1H), 7.29–7.17 (m, 3H), 7.14 (d, J = 7.5 Hz, 1H), 7.09 (t, J = 7.5 Hz, 1H), 6.98 (t, J = 7.5 Hz, 1H), 6.59 (d, J = 7.5 Hz, 1H), 6.01 (s, 1H), 4.50 (d, J = 7.8 Hz, 1H), 4.36 (d, J = 11.2 Hz, 1H), 4.00 (s, 1H), 3.40 (d, J = 11.2 Hz, 1H), 3.08–3.00 (m, 3H), 2.93 (d, J = 16.9 Hz, 1H), 2.37 (dd, J = 12.5, 7.8 Hz, 1H), 2.27 (s, 3H), 2.26 (s, 3H), 2.04 (d, J = 12.5 Hz, 1H); $^{13}C\{^{1}H\}$ NMR (125 MHz, $CDCl_3$) δ 137.6, 137.3, 136.8, 136.6, 131.0, 130.7, 130.4, 127.4, 126.5, 126.2, 126.1, 125.5, 91.6, 84.4, 75.2, 67.1, 50.3, 40.1, 37.5, 33.8, 20.5, 19.9; No molecular ions were observed when analysed by MS, and only species corresponding to hydrolysed material were seen.

(1*S*,4*R*,5*R*)-4-Chloro-1',3'-dihydro-3,8-dioxaspiro[bicyclo[3.2.1]octane-6,2'-indene] (11d). The reaction of **10d** (0.67 g, 2.9 mmol) in DCE (50 mL) with $SOCl_2$ (0.45 mL, 6.2 mmol) and pyridine (1.2 mL, 15 mmol) as for **11a** gave **11d** (248 mg, 34%) as a white solid. The SiO_2 pad used to purify **11d** was washed with EtOAc, and the volatiles removed under reduced pressure. The residue was subjected to flash column chromatography (EtOAc/petroleum spirit 3:7) to give **13d** (233 mg, 32%) as an amorphous white solid in addition to starting material **10d** (74 mg, 11%). Crystals of **11d** suitable for X-ray crystallography were prepared by slow evaporation from CH_2Cl_2/*n*-heptane solution; R_f 0.5 (EtOAc/petroleum spirit 1:9, partial hydrolysis); mp 178–182 °C (CH_2Cl_2/*n*-heptane); $[\alpha]_D^{25}$ –93 (*c* 1.0, CH_2Cl_2); IR: ν_{max} 2988, 2962, 2946, 2928, 2872, 2839 cm^{-1}; ^{1}H NMR (500 MHz, $CDCl_3$) δ 7.28–7.14

(m, 4H), 5.97 (ddd, J = 0.8, 0.8, 0.8 Hz, 1H), 4.43 (br d, J = 7.7 Hz, 1H), 4.36 (br d, J = 11.2 Hz, 1H), 3.87 (s, 1H), 3.41 (ddd, J = 11.2, 0.8, 0.8 Hz, 1H), 3.16 (d, J = 15.4 Hz, 1H), 3.09 (d, J = 15.4 Hz, 1H), 3.08 (d, J = 15.3 Hz, 1H), 2.95 (d, J = 15.3 Hz, 1H), 2.21 (dd, J = 12.6, 7.7 Hz, 1H), 2.04 (br d, J = 12.6 Hz, 1H); $^{13}C\{^1H\}$ NMR (125 MHz, $CDCl_3$) δ 141.8, 141.6, 126.69, 126.66, 124.7, 124.2, 91.8, 85.5, 75.3, 67.1, 54.7, 48.8, 41.4, 39.5; No molecular ions were observed when analysed by MS, and only species corresponding to hydrolysed material were seen.

Bis((1*S*,4*S*,5*R*)-1',3'-dihydro-6,8-dioxaspiro[bicyclo[3.2.1]octane-3,2'-inden]-4-yl) sulfite (13d). R_f 0.6 (EtOAc/petroleum spirit 1:2); mp 200 °C (dec); $[\alpha]_D^{18}$ −102 (*c* 0.54, CH_2Cl_2); IR: ν_{max} 2987, 2970, 2901 cm^{-1}; ^1H NMR (500 MHz, $CDCl_3$) δ 7.17–6.98 (m, 6H), 6.97–6.93 (m, 2H), 5.47 (d, J = 1.7 Hz, 1H), 5.44 (d, J = 1.7 Hz, 1H), 4.51–4.48 (m, 2H), 4.33 (d, J = 1.7 Hz, 1H), 4.27 (d, J = 1.7 Hz, 1H), 4.14 (app. d, J = 7.8 Hz, 2H), 3.81–3.76 (m, 2H), 3.60 (d, J = 16.2 Hz, 1H), 3.57 (d, J = 16.2 Hz, 1H), 3.14 (d, J = 16.2 Hz, 1H), 2.98 (d, J = 16.2 Hz, 1H), 2.87 (d, J = 16.2 Hz, 1H), 2.86 (d, J = 16.2 Hz, 1H), 2.73 (d, J = 16.2 Hz, 1H), 2.72 (d, J = 16.2 Hz, 1H), 2.06–2.01 (m, 2H), 1.99–1.93 (m, 2H); $^{13}C\{^1H\}$ NMR (125 MHz, $CDCl_3$) δ 142.8, 142.6, 140.04, 140.00, 126.5, 126.4, 126.21, 126.16, 124.3, 124.01, 123.94, 123.88, 101.6, 100.9, 78.2, 76.2, 73.7, 73.6, 67.8, 67.7, 48.0, 47.7, 45.6, 45.5, 41.4, 41.3, 40.5, 40.0; HRMS (ESI-TOF) *m/z*: $[M+Na]^+$ calcd for $C_{28}H_{30}NaO_7S$: 533.1604; found: 533.1605.

(1*R*,2*R*,5*S*)-2-Chloro-7,7-dimethyl-3,8-dioxabicyclo[3.2.1]octane (11e). The reaction of **10e** (0.47 g, 3.0 mmol) in DCE (40 mL) with $SOCl_2$ (0.45 mL, 6.2 mmol) and pyridine (1.2 mL, 15 mmol) as for the synthesis of **11a** gave **11e** (312 mg, 59%) as a white solid. The SiO_2 pad was washed with EtOAc, and the volatiles removed under reduced pressure. The residue was subjected to flash column chromatography (EtOAc/petroleum spirit, 1:2) to give **13e** (80 mg, 15%) as a colourless oil. Crystals suitable for X-ray crystallography of **11e** were obtained by vacuum sublimation; R_f 0.7 (EtOAc/petroleum spirit 1:9, partial hydrolysis); mp 63–67 °C; $[\alpha]_D^{23}$ +218 (*c* 0.78, CH_2Cl_2); IR: ν_{max} 2961, 2881, 1459, 1226, 1147, 1102cm^{-1}; ^1H NMR (500 MHz, $CDCl_3$) δ 5.95 (br s, 1H), 4.35 (br d, J = 7.9 Hz, 1H), 4.29 (br d, J = 11.1 Hz, 1H), 3.55 (br s, 1H), 3.35 (br d, J = 11.1 Hz, 1H), 1.92 (dd, J = 12.3, 7.9 Hz, 1H), 1.80 (br d, J = 12.3 Hz, 1H), 1.27 (s, 3H), 1.16 (s, 3H); $^{13}C\{^1H\}$ NMR (125 MHz, $CDCl_3$) δ 91.9, 86.9, 75.7, 67.0, 42.1, 41.7, 31.1, 22.4; No molecular ions were observed when analysed by MS, and only species corresponding to hydrolysed material were seen.

Di((1*S*,4*S*,5*R*)-3,3-dimethyl-6,8-dioxabicyclo[3.2.1]octan-4-yl) sulfite (13e). R_f 0.6 (EtOAc/petroleum spirit; 1:2); $[\alpha]_D^{25}$ −63 (*c* 0.98, CH_2Cl_2); IR: ν_{max} 3683, 2973, 2899, 1394, 1203, 1156, 1056 cm^{-1}; ^1H NMR (500 MHz, $CDCl_3$) δ 5.52 (d, J = 1.8 Hz, 1H), 5.47 (d, J = 1.8 Hz, 1H), 4.47–4.44

(m, 2H), 4.31 (d, *J* = 1.8 Hz, 1H), 4.21 (d, *J* = 1.8 Hz, 1H), 3.98 (app. br d, *J* = 7.3 Hz, 2H), 3.75–3.71 (m, 2H), 1.98–1.92 (m, 2H), 1.70–1.64 (m, 2H), 1.21 (s, 3H), 1.19 (s, 3H), 1.05 (s, 3H), 0.99 (s, 3H); $^{13}C\{^1H\}$ NMR (125 MHz, $CDCl_3$) δ 101.5, 100.8, 79.1, 76.9, 73.54, 73.51, 67.9 (2C), 43.2 (2C), 33.9, 33.8, 32.23, 32.18, 24.50, 24.45; HRMS (ESI-TOF) *m/z*: $[M+Na]^+$ calcd for $C_{16}H_{26}NaO_7S$: 385.1291; found: 385.1310

(1*R*,2*R*,5*S*)-2-Chloro-7,7-bis(4-methoxybenzyl)-3,8-dioxabicyclo[3.2.1]octane (11f). The reaction of **10f** (0.200 g, 0.54 mmol) in DCE (3 mL) with $SOCl_2$ (78 µL, 1.08 mmol) and pyridine (0.217 mL, 1.65 mmol) as for **11a** gave **11f** (65 mg, 31%) as a colourless solid; R_f 0.6 (petroleum spirit/EtOAc, 2:1); mp 121–123 °C; $[\alpha]_D^{20}$ +61.3 (*c* .75, CH_2Cl_2); IR: ν_{max} 2986, 2898, 1610, 1511, 1246 cm^{-1}; ^1H NMR (500 MHz, $CDCl_3$) δ 7.23–7.18 (m, 2H), 6.95–6.89 (m, 4H), 6.80–6.75 (m, 2H), 6.03 (s, 1H), 4.41 (br d, *J* = 7.7 Hz, 1H), 4.33 (dd, *J* = 11.2, 1.1 Hz, 1H), 3.99 (s, *J* = 5.2 Hz, 1H), 3.84 (s, 3H), 3.78 (s, 3H), 3.41 (d, *J* = 11.2 Hz, 1H), 3.06 (d, *J* = 15.0 Hz, 1H), 2.83 (d, *J* = 15.0 Hz, 1H), 2.78 (d, *J* = 13.8 Hz, 1H), 2.71 (d, *J* = 13.8 Hz, 1H), 2.18 (dd, *J* = 12.3, 7.7 Hz, 1H), 2.10 (dd, *J* = 12.3, 1.1 Hz, 1H); $^{13}C\{^1H\}$ NMR (125 MHz, $CDCl_3$) δ 158.2, 158.1, 131.5, 130.6, 130.4, 130.0, 114.0, 113.5, 91.8, 83.7, 75.2, 67.2, 55.3, 55.2, 50.5, 42.0, 39.4, 36.7; No molecular ions were observed when analysed by MS, and only species corresponding to hydrolysed material were seen.

Representative procedure for the formation of alcohols 12a,c–f.

(1*R*,2*S*,5*S*)-7,7-Dibenzyl-3,8-dioxabicyclo[3.2.1]octan-2-ol (12a). To a stirred solution of **10a** (0.62 g, 2.0 mmol) in DCE (30 mL) was added $SOCl_2$ (0.30 mL, 4.1 mmol) and pyridine (0.80 mL, 10 mmol) and the resulting solution was heated to reflux for 24 hours. The mixture was diluted with CH_2Cl_2 (20 mL) and poured into ice cooled 2 M HCl (15 mL) and the phases were separated. The organic phase was washed with satd. aqueous $NaHCO_3$ (15 mL) and dried over Na_2SO_4, filtered and the volatiles removed under reduced pressure. The residue was purified via flash column chromatography (EtOAc/petroleum spirit, 1:1) using water treated silica (0.6 mL H_2O in 30 mL SiO_2) to afford **12a** (565 mg 91%) as a white solid. Crystals suitable for X-ray crystallography were prepared from a solution of CH_2Cl_2/*n*-heptane; R_f 0.3 (EtOAc/petroleum spirit, 1:2); mp 110–115 °C; $[\alpha]_D^{25}$ −15 (*c* 1.2, CH_2Cl_2); IR: ν_{max} 3411, 3085, 3059, 3028, 2955, 1727, 1602, 1582, 1496 cm^{-1}; ^1H NMR (500 MHz, $CDCl_3$) δ 7.38–7.19 (m, 9H), 7.04–7.02 (m, 1H), 4.95 (br d, *J* = 10.4 Hz, 1H), 4.28 (br dd, *J* = 4.5, 4.5 Hz, 1H), 4.16 (dd, *J* = 11.4, 1.3 Hz, 1H), 3.95 (d, *J* = 10.4 Hz, 1H), 3.91 (s, 1H), 3.30 (d, *J* = 11.4 Hz, 1H), 3.25 (d, *J* = 14.9 Hz, 1H), 2.95 (d, *J* = 14.9 Hz, 1H), 2.85 (d, *J* = 13.5 Hz, 1H), 2.75 (d, *J* = 13.5 Hz, 1H), 2.21–2.19 (m, 2H); $^{13}C\{^1H\}$ NMR (125 MHz, $CDCl_3$) δ 138.9, 138.5, 130.4, 129.8, 128.5, 128.0, 126.3,

126.3, 89.9, 82.4, 76.1, 65.6, 48.2, 43.5, 38.9, 38.0; HRMS (ESI-TOF) *m/z*: $[M+Na]^+$ calcd for $C_{20}H_{22}NaO_3$: 333.1461; found: 333.1471.

(1*R*,2*S*,5*S*)-7,7-Bis(2-methylbenzyl)-3,8-dioxabicyclo[3.2.1]octan-2-ol (12c). The reaction of **10c** (0.35 g, 1.0 mmol) in DCE (30 mL) with $SOCl_2$ (0.20 mL, 2.8 mmol) and pyridine (0.40 mL, 5 mmol) as for **12a** gave **12c** (287 mg, 85%) as a crystalline white solid; R_f 0.4 (EtOAc/petroleum spirit, 1:2); mp 62–67 °C; $[\alpha]_D^{20}$ +34 (*c* 0.50, CH_2Cl_2); IR: ν_{max} 3419, 3062, 3020, 2956. 2871, 1726, 1603, 1491 cm^{-1}; ^{1}H NMR (500 MHz, $CDCl_3$) δ 7.36 (d, *J* = 7.5 Hz, 1H), 7.27–7.16 (m, 3H), 7.13 (br d, *J* = 7.5 Hz, 1H), 7.08 (ddd, *J* = 7.5, 7.5, 1.4 Hz, 1H), 6.98 (ddd, *J* = 7.5, 7.5, 1.4 Hz, 1H), 6.63 (dd, *J* = 7.5, 1.4 Hz, 1H), 4.89 (d, *J* = 9.7 Hz, 1H), 4.39 (br d, *J* = 7.8 Hz, 1H), 4.19 (dd, *J* = 11.5, 1.7 Hz, 1H), 3.85 (s, 1H), 3.82 (br d, *J* = 9.7 Hz, 1H), 3.28 (br d, *J* = 11.5 Hz, 1H), 3.14 (d, *J* = 16.9 Hz, 1H), 3.06 (d, *J* = 13.9 Hz, 1H), 2.98 (d, *J* = 13.9 Hz, 1H), 2.97 (d, *J* = 16.9 Hz, 1H), 2.32 (dd, *J* = 12.4, 7.8 Hz, 1H), 2.27 (s, 3H), 2.24 (s, 3H), 2.12 (br d, *J* = 12.4 Hz, 1H); $^{13}C\{^1H\}$ NMR (125 MHz, $CDCl_3$) δ 137.5, 137.3 (2C), 137.0, 130.8, 130.6, 130.3, 127.7, 126.4, 126.0, 125.9, 125.5, 89.8, 82.9, 76.1, 65.6, 48.3, 39.9, 37.7, 33.5, 20.5, 19.9; HRMS (ESI-TOF) *m/z*: $[M+Na]^+$ calcd for $C_{22}H_{26}NaO_3$: 361.1774; found: 361.1788.

(1*S*,4*S*,5*R*)-1',3'-Dihydro-3,8-dioxaspiro[bicyclo[3.2.1]octane-6,2'-inden]-4-ol (12d). The reaction of **10d** (0.44 g, 1.9 mmol) in DCE (30 mL) with $SOCl_2$ (0.30 mL, 4.1 mmol) and pyridine (0.80 mL, 10 mmol) as for **12a** gave **12d** (125 mg, 28%) and **13d** (11 mg, 2%), and recovered starting material **10d** (175 mg, 40%). Crystals suitable for X-ray crystallography of **12d** were prepared by slow evaporation from EtOAc solution; R_f 0.3 (EtOAc/petroleum spirit, 1:1); mp 169–171 °C (EtOAc); $[\alpha]_D^{25}$ +58 (*c* 0.52, CH_2Cl_2); IR: ν_{max} 3458, 3433, 3021, 2967, 2953, 2918, 2871, 2844, 1727, 1602 cm^{-1}; ^{1}H NMR (500 MHz, $CDCl_3$) δ 7.24–7.14 (m, 4H), 4.87 (d, *J* = 10.2 Hz, 1H), 4.34 (br d, *J* = 7.6 Hz, 1H), 4.21 (dd, *J* = 11.5, 1.5 Hz, 1H), 3.78 (br d, *J* = 9.7 Hz, 1H), 3.73 (s, 1H), 3.30 (d, *J* = 11.5 Hz, 1H), 3.20 (d, *J* = 15.6 Hz, 1H), 3.14 (d, *J* = 15.6 Hz, 1H), 3.07 (d, *J* = 15.2 Hz, 1H), 2.95 (d, *J* = 15.2 Hz, 1H), 2.17 (dd, *J* = 12.4, 7.6 Hz, 1H), 2.08 (dd, *J* = 12.4, 1.5 Hz, 1H); $^{13}C\{^1H\}$ NMR (125 MHz, $CDCl_3$) δ 142.3, 141.9, 126.6, 126.5, 124.6, 124.3, 90.4, 84.4, 76.4, 65.6, 52.8, 48.9, 41.4, 39.4; HRMS (ESI-TOF) *m/z*: $[M+H]^+$ calcd for $C_{14}H_{17}O_3$: 233.1172 found: 233.1187.

(1*R*,2*S*,5*S*)-7,7-Dimethyl-3,8-dioxabicyclo[3.2.1]octan-2-ol (12e). The reaction of **10e** (0.48 g, 3.0 mmol) in DCE (50 mL) with $SOCl_2$ (0.45 mL, 6.2 mmol) and pyridine (1.2 mL, 15 mmol) as for **12a** gave **12e** (244 mg 51%) as a crystalline white solid, sulphite **13e** (43 mg, 8%) and recovered starting material **10e** (46 mg, 10%); R_f 0.3 (EtOAc/petroleum spirit, 1:2); mp 54–62 °C (EtOAc/*n*-heptane);

$[\alpha]_D^{25}$ +24 (*c* 0.81, CH_2Cl_2); IR: ν_{max} 3424, 2970, 2953, 2936, 2870, 1729 cm^{-1}; ^1H NMR (500 MHz, $CDCl_3$) δ 4.81 (br d, *J* = 0.4 Hz, 1H), 4.27 (br d, *J* = 7.0 Hz, 1H), 4.13 (dd, *J* = 1.4, 1.5 Hz, 1H), 3.89 (d, *J* = 10.4 Hz, 1H), 3.39 (br s, 1H), 3.23 (br d, *J* = 0.4 Hz, 1H), 1.87, (dd, *J* = 12.2, 7.4 Hz, 1H), 1.82 (dd, *J* = 12.2, 1.5 Hz, 1H), 1.28 (s, 3H), 1.15 (s, 3H); $^{13}C\{^1H\}$ NMR (125 MHz, $CDCl_3$) δ 90.0, 85.7, 76.7, 65.5, 41.6, 39.8, 31.2, 22.0; HRMS (ESI-TOF) *m/z*: $[M+Na]^+$ calcd for $C_8H_{14}NaO_3$: 181.0835; found: 181.0849.

(1*R*,2*S*,5*S*)-7,7-Bis(4-methoxybenzyl)-3,8-dioxabicyclo[3.2.1]octan-2-ol (12f). The reaction of **10f** (0.122 g, 0.33 mmol) in DCE (2 mL) with $SOCl_2$ (48 µL, 0.66 mmol) and pyridine (0.133 mL, 1.65 mmol) as for **12a** gave **12f** as a colourless solid (70 mg, 57%); R_f 0.4 (petroleum spirit/EtOAc, 1:1); mp 48–50 °C; $[\alpha]_D^{20}$ −24.3 (*c* 1.0, CH_2Cl_2); IR: ν_{max} 3421, 2937, 2834, 1610, 1510, 1244 cm^{-1}; ^1H NMR (500 MHz, $CDCl_3$) δ 7.23–7.20 (m, 2H), 6.96–6.92 (m, 2H), 6.92–6.88 (m, 2H), 6.79–6.76 (m, 2H), 4.92 (s, 1H), 4.29–4.26 (m, 1H), 4.15 (dd, *J* = 11.4, 1.4 Hz, 1H), 3.87–3.85 (m, 1H), 3.83 (s, 3H), 3.77 (s, 3H), 3.29 (d, *J* = 11.4 Hz, 1H), 3.16 (d, *J* = 14.8 Hz, 1H), 2.86 (d, *J* = 14.8 Hz, 1H), 2.77 (d, *J* = 13.8 Hz, 1H), 2.67 (d, *J* = 13.8 Hz, 1H), 2.16–2.12 (m, 2H), 1.26 (br s, 1H); $^{13}C\{^1H\}$ NMR (125 MHz, $CDCl_3$) δ 158.1, 158.0, 131.4, 130.79, 130.85, 130.5, 113.9, 113.4, 89.9, 82.4, 76.1, 65.7, 55.3, 55.2, 48.4, 42.4, 38.9, 36.8; HRMS (ESI-TOF) *m/z*: $[M+Na]^+$ calcd for $C_{22}H_{26}NaO_5$: 393.1672; found: 393.1685.

(1*S*,4*S*,5*R*)-4-Chloro-6,8-dioxabicyclo[3.2.1]oct-2-ene (16).[3] The reaction of **15** (0.39 g, 3.0 mmol) in DCE (30 mL) with $SOCl_2$ (0.45 mL, 6.2 mmol) and pyridine (1.2 mL, 15 mmol) was performed as for **11a** with purification by flash column chromatography (EtOAc/petroleum spirit, 1:9) to affording order of elution **17a** (32 mg, 7%), **16** (142 mg, 32%), then **17b** (5 mg, 1%) as colourless oils; R_f 0.4

(EtOAc/petroleum spirit, 1:9); $[\alpha]_D^{25}$ −310 (*c* 1.0, CH_2Cl_2); IR: ν_{max} 2966, 2893 cm^{-1}; ^1H NMR (500 MHz, $CDCl_3$) δ 6.18 (ddd, *J* = 9.7, 4.8, 1.5 Hz, 1H), 5.82 (ddd, *J* = 9.7, 3.8, 1.5 Hz, 1H), 5.63 (br dd, *J* = 1.5, 1.5 Hz, 1H), 4.76 (ddd, *J* = 4.8, 3.2, 1.5 Hz, 1H), 4.07 (ddd, *J* = 3.8, 1.5, 1.5 Hz, 1H), 3.78–3.75 (m, 2H); $^{13}C\{^1H\}$ NMR (125 MHz, $CDCl_3$) δ 130.1, 125.1, 102.1, 70.5, 70.4, 52.5.

(1*R*,2*R*,5*R*)-2-Chloro-6,8-dioxabicyclo[3.2.1]oct-3-ene (17a).[1] R_f 0.7 (EtOAc/petroleum spirit, 1:9); $[\alpha]_D^{25}$ −13 (*c* 1.0, CH_2Cl_2); IR: ν_{max} 2974, 2904, 1633 cm^{-1}; ^1H NMR (500 MHz, $CDCl_3$) δ 5.93 (ddd, *J* = 9.6, 3.1, 1.9 Hz, 1H), 5.72 (ddd, *J* = 9.6, 1.9, 1.9 Hz, 1H), 5.52 (d, *J* = 3.1 Hz, 1H), 5.00 (dddd, *J* = 3.9, 1.9, 1.9, 1.9 Hz, 1H), 4.62 (dddd, *J* = 6.2, 3.9, 1.9, 1.9 Hz, 1H), 4.29 (dd, *J* = 8.4, 1.9 Hz, 1H), 3.96 (ddd, *J* = 8.4, 6.2, 1.9 Hz, 1H); $^{13}C\{^1H\}$ NMR (125 MHz, $CDCl_3$) δ 129.4, 127.3, 95.6, 75.1, 63.6, 55.7.

(1*R*,2*S*,5*R*)-2-Chloro-6,8-dioxabicyclo[3.2.1]oct-3-ene (17b).[1] R_f 0.3 (EtOAc/petroleum spirit, 1:9); $[\alpha]_D^{25}$ +149 (*c* 1.0, CH_2Cl_2); IR: ν_{max} 2961, 2923, 2852, 1634 cm^{-1}; ^{1}H NMR (500 MHz, $CDCl_3$) δ 6.05 (ddd, *J* = 9.5, 3.5, 1.0 Hz, 1H), 5.85 (dddd, *J* = 9.5, 4.4, 1.9, 1.0 Hz, 1H), 5.60 (dd, *J* = 3.5, 1.0 Hz, 1H), 4.78 (dddd, *J* = 6.5, 1.9, 1.9, 1.0 Hz, 1H), 4.17 (ddd, *J* = 4.4, 1.0, 1.0 Hz, 1H), 4.01 (dd, *J* = 8.2, 6.5 Hz, 1H), 3.56 (dd, *J* = 8.2, 1.9 Hz, 1H); $^{13}C\{^1H\}$ NMR (125 MHz, $CDCl_3$) δ 129.7, 125.0, 95.6, 76.7, 64.8, 54.2.

Reactions of alcohol 18 with $SOCl_2$.

The reaction of **18** (0.63 g, 2.9 mmol) in DCE (50 mL) with $SOCl_2$ (0.45 mL, 6.2 mmol) and pyridine (1.2 mL, 15 mmol) was performed as for **11a** with purification by flash column chromatography (EtOAc/petroleum spirit 2:23). Eluting first was **20** (114 mg, 17%, dr 91:9) as a colourless oil followed by **19** (10 mg, 1%) as a white crystalline solid. **(1*S*,5*R*)-3-(Chloro(phenyl)methyl)-6,8-dioxabicyclo[3.2.1]oct-3-ene** (**20**) major isomer: ^{1}H NMR (500 MHz, $CDCl_3$) δ 7.39–7.35 (m, 2H), 7.32–7.23 (m, 3H), 6.77 (d, *J* = 2.2 Hz, 1H), 5.57 (d, *J* = 2.2 Hz, 1H), 4.65–4.62 (m, 1H), 4.39 (s, 1H), 3.76 (ddd, *J* = 7.0, 5.2 Hz, 1H), 3.71 (br d, *J* = 7.0 Hz, 1H), 3.07–3.01 (m, 1H), 2.64 (br d, *J* = 15.2 Hz, 1H); $^{13}C\{^1H\}$ NMR (125 MHz, $CDCl_3$) δ 135.4, 134.6, 131.2, 128.9, 128.4, 127.6, 102.1, 72.9, 68.0, 61.2, 31.3; minor isomer: ^{1}H NMR (500 MHz, $CDCl_3$, partial) δ 7.10 (br s, 1H), 4.67 (br s, 1H), 2.86 (br d, *J* = 14.8 Hz, 1H), 2.80–2.75 (m, 1H); $^{13}C\{^1H\}$ NMR (125 MHz, $CDCl_3$) δ 136.2, 130.7, 130.4, 128.9, 128.3, 127.1, 103.1, 73.5, 68.8, 62.2, 35.5.

(1*S*,5*R*)-3-((E)-Benzylidene)-4-chloro-6,8-dioxabicyclo[3.2.1]octane (**19**). mp 108–113 °C; ^{1}H NMR (500 MHz, $CDCl_3$) δ 7.41–7.35 (m, 4H), 7.30–7.26 (m, 1H), 6.56 (d, *J* = 2.0 Hz, 1H), 5.45 (d, *J* = 1.7 Hz, 1H), 4.73–4.70 (m, 1H), 4.57 (br s, 1H), 3,89 (d, *J* = 6.8 Hz, 1H), 3.78 (ddd, *J* = 6.8, 5.1, 1.5 Hz, 1H), 3.20 (br d, *J* = 14.9 Hz, 1H), 2.20 (br d, *J* = 14.9 Hz, 1H); $^{13}C\{^1H\}$ NMR (125 MHz, $CDCl_3$) δ 135.3, 133.6, 130.9, 128.6, 128.5, 127.6, 101.9, 73.9, 67.7, 55.1, 36.5; HRMS (ESI-TOF) *m/z*: $[M+H]^+$ calcd for $C_{13}H_{14}ClO_2$: 237.0677; found: 237.0674.

Representative procedure for the rearrangement using Appel conditions.

Preparation of 11a using Appel conditions. To a stirred solution of **10a** (311 mg, 1.0 mmol) in DCE (5 mL) was added PPh_3 (1.05 g, 4.0 mmol), CCl_4 (0.24 mL, 2.5 mmol) and Et_3N (14 µL, 0.10 mmol) and the resulting solution was heated to reflux for 24 hours. The mixture was concentrated under reduced pressure, and the residue was purified via a short column of silica (10 cm) (EtOAc/petroleum spirit, 2:1) to afford **11a** (146 mg, 44 %) as a colourless solid. The data was identical to that obtained previously.

Preparation of 11f using Appel conditions. The reaction of **10f** (0.250 g, 0.67 mmol), PPh_3 (0.700 g, 2.68 mmol), CCl_4 (0.129 mL, 1.34 mmol) and Et_3N (10 µL, 0.071 mmol) in DCE (7 mL) as per the reaction of **10a** under Appel conditions afforded **11f** (83 mg, 32%) as a colourless solid. The data was identical to that obtained previously.

Preparation of 12e using Appel conditions. The reaction of **10e** (108 mg, 0.68 mmol), PPh_3 (716 mg, 2.73 mmol), CCl_4 (0.140 mL, 1.36 mmol) and Et_3N (11 µL, 0.078 mmol) in DCE (3.5 mL) as per the reaction of **10a** under Appel conditions, with purification by column chromatography using water treated silica (0.6 mL H_2O in 30 mL SiO_2) afforded **12e** (38 mg, 35%) as a colourless oil. The data was identical to that obtained previously.

Preparation of 16 using Appel conditions. The reaction of alcohol **15** (128 mg, 1.00 mmol), PPh_3 (1.042 g, 3.97 mmol), CCl_4 (237 µL, 2.44 mmol) in DCE (5 mL) as per the reaction of **10a** under Appel conditions, with purification by flash chromatography (gradient elution of EtOAc/petroleum spirit, 4:21 to 1:4) afforded **16** (64 mg, 44%) as a colourless oil. The data was identical to that obtained previously.

(1*R*,2*R*,5*S*)-2-Allyl-7,7-dibenzyl-3,8-dioxabicyclo[3.2.1]octane (21). To a stirred solution of **11a** (251 mg, 0.763 mmol) and allyltrimethylsilane (260 mg, 2.28 mmol) in CH_2Cl_2 (5 mL) was added $AlCl_3$ (118 mg, 0.885 mmol). After 30 minutes, the reaction was quenched by the addition of saturated aqueous $NaHCO_3$ (5 mL) and the mixture extracted with CH_2Cl_2 (3 × 5 mL). The combined organic layers were passed through a cotton pad and the volatiles removed under reduced pressure. Purification of the residue by flash column chromatography (EtOAc/petroleum spirit 1:9) afforded **21** (144 mg, 56%) as a colourless oil; R_f 0.5 (EtOAc/petroleum spirit, 1:9); $[\alpha]_D^{29}$ +10 (*c* 0.62, CH_2Cl_2); IR: ν_{max} 3084, 3061, 3001, 3026, 2949, 2861, 1640, 1602, 1581, 1495 cm^{-1}; ^1H NMR (500 MHz, $CDCl_3$) δ 7.41–7.16 (m, 8H), 7.05–6.99 (m, 2H), 5.82 (dddd, *J* = 17.0, 10.1, 7.6, 6.8 Hz, 1H), 5.18–5.08 (m, 2H), 4.28–4.22 (m, 1H), 4.01 (dd, *J* = 11.2, 1.3 Hz, 1H), 3.84 (s, 1H), 3.79 (dd, *J* = 8.6, 5.9 Hz, 1H), 3.30–3.22 (m, 2H), 2.94 (d, *J* = 15.1 Hz, 1H), 2.87–2.75 (m, 3H), 2.49–2.42 (m, 1H), 2.20–2.12 (m, 2H); $^{13}C\{^1H\}$ NMR (125 MHz, $CDCl_3$) δ 139.6, 139.0, 134.8, 130.5, 129.9, 128.4, 127.9, 126.1, 126.0, 117.3, 81.2, 75.8, 72.0, 66.1, 48.7, 43.4, 39.9, 38.5, 33.9; HRMS (ESI-TOF) *m/z*: $[M+H]^+$ calcd for $C_{23}H_{27}O_2$: 335.2006; found: 335.1997

(1*R*,2*R*,5*S*)-7,7-Dibenzyl-2-(4-methoxyphenyl)-3,8-dioxabicyclo[3.2.1]octane (22). To a stirred solution of **11a** (445 mg, 1.35 mmol) and anisole (499 mg, 4.61 mmol) in DCE (7 mL) was added $AlCl_3$ (263 mg, 1.97 mmol) and the resulting solution was stirred for 30 min. The reaction was quenched by the addition of saturated aqueous $NaHCO_3$ (20 mL) and extracted with CH_2Cl_2 (2 × 50 mL). The combined organics were dried over Na_2SO_4, filtered and the volatiles removed under reduced pressure. Purification of the residue by flash column chromatography (EtOAc/petroleum spirit 1:2) afforded **22** (195 mg, 36%) as a colourless viscous oil; R_f 0.4 (EtOAc/petroleum spirit; 1:2); $[\alpha]_D^{29}$ +7 (*c* 0.67, CH_2Cl_2); IR: ν_{max} 3062, 3026, 2999, 2913, 2871, 2851, 2838, 1611, 1604, 1582. 1512 cm^{-1}; ^{1}H NMR (500 MHz, $CDCl_3$) δ 7.33–7.10 (m, 12H), 6.93–6.88 (m, 2H), 4.27 (d, *J* = 6.5 Hz, 1H), 4.02 (d, *J* = 6.5 Hz, 1H), 3.95–3.89 (m, 1H), 3.82 (s, 3H), 3.66 (dd, *J* = 11.8, 2.9 Hz, 1H), 3.36 (dd, *J* = 11.8, 4.4 Hz, 1H), 2.86 (d, *J* = 14.6 Hz, 1H), 2.66 (d, *J* = 13.4 Hz, 1H), 2.52 (*J* = 14.6 Hz, 1H), 2.50 (d, *J* = 13.4 Hz, 1H), 2.10 (dd, *J* = 12.7, 5.4 Hz, 1H), 1.54 (dd, *J* = 12.7, 11.1 Hz, 1H); ^{13}C{^{1}H} NMR (125 MHz, $CDCl_3$) δ 158.3, 139.0, 138.0, 137.5, 131.7, 130.7, 129.8, 128.3, 128.2, 128.1, 126.7, 126.7, 126.5, 114.1, 89.9, 77.8, 63.6, 55.3, 51.0, 48.3, 45.5, 39.5, 38.3.; HRMS (ESI-TOF) *m/z*: $[M+Na]^+$ calcd for $C_{27}H_{28}NaO_3$: 423.1931; found: 423.1948.

(1*R*,2*R*,5*S*)-7,7-Dibenzyl-2-(4-phenoxyphenyl)-3,8-dioxabicyclo[3.2.1]octane (23). To a stirred solution of **11a** (315 mg, 0.958 mmol) and diphenyl ether (885 mg, 5.20 mmol) in DCE (10 mL) was added $AlCl_3$ (175 mg, 1.31 mmol) and the resulting solution was stirred for 5 min. The reaction was quenched by the addition of saturated aqueous $NaHCO_3$ (20 mL) then extracted with CH_2Cl_2 (2 × 30 mL). Purification of the residue by flash column chromatography (EtOAc/petroleum spirit 3:7) afforded **23** (236 mg, 53%) as a glassy solid; R_f 0.4 (EtOAc/petroleum spirit, 3:7); $[\alpha]_D^{25}$ −5 (*c* 0.62, CH_2Cl_2); IR: ν_{max} 3062, 3025, 2918, 2871, 1589, 1504, 1488 cm^{-1}; ^{1}H NMR (500 MHz, $CDCl_3$) δ 7.39–7.17 (m, 9H), 7.16–7.09 (m, 5H), 7.08–7.03 (m, 2H), 7.02–6.98 (m, 2H), 6.95–6.90 (m, 1H), 4.31 (d, *J* = 6.2 Hz, 1H), 4.07 (d, *J* = 6.2 Hz, 1H), 3.97–3.91 (m, 1H), 3.68 (dd, *J* = 11.8, 2.8 Hz, 1H), 3.38 (dd, *J* = 11.8, 4.4 Hz, 1H), 2.87 (d, *J* = 14.6 Hz, 1H), 2.68 (d, *J* = 13.4 Hz, 1H), 2.53 (d, *J* = 14.6 Hz, 1H), 2.53 (d, *J* = 13.4 Hz, 1H), 2.11 (dd, *J* = 12.7, 5.3 Hz, 1H), 1.56 (dd, *J* = 12.7, 10.4 Hz, 1H); ^{13}C{^{1}H} NMR (125 MHz, $CDCl_3$) δ 157.1, 155.9, 138.6, 137.9, 137.5, 134.5, 130.7, 130.1, 129.7, 128.4, 128.2, 128.1, 126.8, 126.7, 126.5, 123.3, 119.0, 118.9, 89.7, 77.9, 63.7, 51.1, 48.3, 45.5, 39.4, 38.3; HRMS (ESI-TOF) *m/z*: $[M+Na]^+$ calcd for $C_{32}H_{30}NaO_3$: 485.2087; found: 485.2096.

(1*R*,5*S*)-7,7-Dibenzyl-3,8-dioxabicyclo[3.2.1]octan-2-one (24). To a stirred solution of **12a** (618 mg, 1.99 mmol) in DCE (25 mL) was added iodobenzene diacetate (658 mg, 2.04 mmol) and TEMPO (37 mg, 0.24 mmol) and the resulting mixture stirred overnight at ambient temperature. The volatiles were removed under reduced pressure and the residue subjected to flash column chromatography

(EtOAc/petroleum spirit 1:3), affording **24** (247 mg, 40%) as a white solid. Crystals suitable for X-ray crystallography were prepared by slow evaporation from an *i*-Pr_2O solution; R_f 0.3 (EtOAc/petroleum spirit, 1:3); mp 125–128 °C; $[\alpha]_D^{23}$ −39 (*c* 0.40, CH_2Cl_2); IR: ν_{max} 3085, 3059, 3028, 2958, 2924, 2898, 2856, 1745, 1603, 1583, 1496 cm^{-1}; ^1H NMR (500 MHz, $CDCl_3$) δ 7.38–7.30 (m, 4H), 7.29–7.20 (m, 4H), 7.09–7.05 (m, 2H), 4.60–4.52 (m, 2H), 4.46 (s, 1H), 4.13 (d, *J* = 10.5 Hz, 1H), 2.95 (d, *J* = 15.0 Hz, 1H), 2.87 (d, *J* = 13.8 Hz, 1H), 2.72 (d, *J* = 13.8 Hz, 1H), 2.70 (d, *J* = 15.0 Hz, 1H), 2.28 (dd, *J* = 13.0, 8.0 Hz, 1H), 2.14 (dd, *J* = 13.0, 2.0 Hz, 1H); $^{13}C\{^1H\}$ NMR (125 MHz, $CDCl_3$) δ 167.4, 137.9, 137.5, 130.3, 130.0, 128.5, 128.3, 126.7, 126.6, 81.8, 74.7, 72.2, 51.9, 42.0, 40.0, 38.8; HRMS (ESI-TOF) *m/z*: $[M+Na]^+$ calcd for $C_{20}H_{20}NaO_3$: 331.1305; found: 331.1318.

Single crystal X-ray crystallography

Single crystal of were mounted in Paratone-N oil on a MiTeGen micromount. X-ray diffraction data were collected at temperatures between 100(2) K – 119(2) K (as specified) on a Rigaku-Oxford Diffraction Synergy single crystal diffractometer using Cu Kα radiation or at 150(2) K on a Oxford Diffraction Xcalibur single crystal diffractometer using Mo Kα radiation.[4] The data sets were corrected for absorption using a multi-scan method, and the structures solved by intrinsic phasing (SHELXT)[5] and refined by full-matrix least squares on F2 by SHELXL,[6] interfaced through the programs X-Seed (version 4)[7] and Olex2.3.[8] All non-hydrogen atoms were refined with anisotropic displacement parameters and hydrogen atoms were included as invariants at geometrically estimated positions.

For all compounds, the absolute structure was determined by anomalous dispersion effects and reference to the chiral starting material, where chirality at C1 (starting material) should be retained in all products. For compounds **10d** and **12d** the Flack and Hooft y parameters poorly defined, but examination of the Bijvoet pairs using the Hooft method suggests that the compounds are correctly assigned, although the analysis is not conclusive for **10d**. For **10d** P2(true) = 0.989, P3(true) = 0.114 and P3(rac-twin) = 0.885; **12d** P2(true) = 1.000, P3(true) = 1.000 and P3(rac-twin) = 0.000. This was true for multiple crystals of **10d**.

Tables S1–S3 list the X-ray experimental data and refinement parameters for the crystal structures. Perspective views of the asymmetric units of the structures and other salient features are shown in Figures S1–S9.

Full details of the structure determination have been deposited with the Cambridge Crystallographic Data Centre as CSD 2327007-2327015 (compounds **10d –** 2327007; **10e** – 2327012; **11a** – 2327010; **11d** – 2327015; **11e** – 2327008; **12a** – 2327013; **12d** – 2327009; **13b** – 2327011 and **24** – 2327014). Copies of this information may be obtained free of charge from The Director, CCDC, 12 Union Street, Cambridge CB2 1EZ, U.K. (fax, +44-1223-336-033; e-mail, deposit@ccdc.cam.ac.uk).

Table S1. X-ray experimental data and refinement parameters for **10d**, **10e** and **11a**.

Compound	**10d**	**10e**	**11a**
CCDC number	2327007	2327012	2327010
Identification code	cyroxOH2_auto	exp_211_auto	JKAux04Cl01
Empirical formula	$C_{14}H_{16}O_3$	$C_8H_{14}O_3$	$C_{20}H_{21}ClO_2$
Formula weight	232.27	158.19	328.82
Temperature/K	100(2)	104(2)	150(2)
Crystal system	monoclinic	trigonal	monoclinic
Space group	$P2_1$	$P3_2$	$P2_1$
a/Å	8.8024(3)	10.9631(2)	6.7910(3)
b/Å	5.9126(2)	10.9631(2)	9.0122(3)
c/Å	11.0209(3)	5.92714(15)	27.4291(11)
α/°	90	90	90
β/°	89.984(3)	90	89.864(4)
γ/°	90	120	90
Volume/$Å^3$	573.58(3)	616.93(3)	1678.71(11)
Z	2	3	4
$\rho_{calc}g/cm^3$	1.345	1.277	1.301
μ/mm^{-1}	0.761	0.798	0.235
F(000)	248.0	258.0	696.0
Crystal size/mm^3	0.325 × 0.176 × 0.047	0.293 × 0.043 × 0.019	0.5 × 0.38 × 0.2
Radiation	Cu Kα (λ = 1.54184)	Cu Kα (λ = 1.54184)	Mo Kα (λ = 0.71073)
2Θ range for data collection/°	8.022 to 159.448	9.314 to 156.058	7.428 to 58.286
Index ranges	$-11 \le h \le 11$, $-7 \le k \le 7$, $-13 \le l \le 13$	$-13 \le h \le 13$, $-13 \le k \le 13$, $-7 \le l \le 7$	$-8 \le h \le 9$, $-10 \le k \le 12$, $-35 \le l \le 37$
Reflections collected	9494	12359	11095
Independent reflections	2400 [R_{int} = 0.0830, R_{sigma} = 0.0620]	1734 [R_{int} = 0.0551, R_{sigma} = 0.0318]	6123 [R_{int} = 0.0330, R_{sigma} = 0.0612]
Data/restraints/parameters	2400/1/155	1734/1/104	6123/1/415
Goodness-of-fit on F^2	1.072	1.127	1.044
Final R indexes [I>=2σ (I)]	R_1 = 0.0462, wR_2 = 0.1160	R_1 = 0.0298, wR_2 = 0.0816	R_1 = 0.0488, wR_2 = 0.0910
Final R indexes [all data]	R_1 = 0.0513, wR_2 = 0.1267	R_1 = 0.0303, wR_2 = 0.0817	R_1 = 0.0681, wR_2 = 0.0974
Largest diff. peak/hole / e $Å^{-3}$	0.31/-0.23	0.16/-0.15	0.35/-0.33
Flack parameter	0.4(2)	-0.05(15)	0.03(4)

Table S2. X-ray experimental data and refinement parameters for **11d**, **11e** and **12a**.

Compound	**11d**	**11e**	**12a**
CCDC number	2327015	2327008	2327013
Identification code	cyroxRCl_auto	cyrM2RCl_auto	exp_227_auto
Empirical formula	$C_{14}H_{15}ClO_2$	$C_8H_{13}ClO_2$	$C_{40}H_{44}O_6$
Formula weight	250.71	176.63	620.75
Temperature/K	107(2)	104(2)	110(2)
Crystal system	orthorhombic	monoclinic	triclinic
Space group	$P2_12_12_1$	$P2_1$	P1
a/Å	6.01671(12)	5.85210(10)	6.31790(10)
b/Å	8.00444(16)	9.16200(10)	9.1992(2)
c/Å	24.6468(4)	7.97400(10)	13.8173(3)
α/°	90	90	82.676(2)
β/°	90	95.7800(10)	86.756(2)
γ/°	90	90	89.6190(10)
Volume/$Å^3$	1187.00(4)	425.368(10)	795.23(3)
Z	4	2	1
$\rho_{calc}g/cm^3$	1.403	1.379	1.296
μ/mm^{-1}	2.735	3.562	0.685
F(000)	528.0	188.0	332.0
Crystal size/mm^3	0.166 × 0.124 × 0.038	0.347 × 0.174 × 0.084	0.132 × 0.072 × 0.041
Radiation	Cu Kα (λ = 1.54184)	Cu Kα (λ = 1.54184)	Cu Kα (λ = 1.54184)
2Θ range for data collection/°	7.174 to 159.98	11.152 to 158.894	6.46 to 159.102
Index ranges	$-7 \le h \le 6$, $-9 \le k \le 10$, $-24 \le l \le 31$	$-7 \le h \le 7$, $-11 \le k \le 11$, $-8 \le l \le 10$	$-8 \le h \le 8$, $-11 \le k \le 11$, $-16 \le l \le 17$
Reflections collected	12493	14354	48726
Independent reflections	2539 [R_{int} = 0.0409, R_{sigma} = 0.0275]	1815 [R_{int} = 0.0456, R_{sigma} = 0.0198]	6120 [R_{int} = 0.0461, R_{sigma} = 0.0237]
Data/restraints/parameters	2539/0/155	1815/1/103	6120/3/417
Goodness-of-fit on F^2	1.055	1.116	1.043
Final R indexes [I>=2σ (I)]	R_1 = 0.0295, wR_2 = 0.0731	R_1 = 0.0293, wR_2 = 0.0767	R_1 = 0.0406, wR_2 = 0.1142
Final R indexes [all data]	R_1 = 0.0310, wR_2 = 0.0740	R_1 = 0.0296, wR_2 = 0.0770	R_1 = 0.0421, wR_2 = 0.1159
Largest diff. peak/hole / e $Å^{-3}$	0.24/-0.22	0.20/-0.22	0.40/-0.17
Flack parameter	-0.026(8)	-0.019(15)	0.02(7)

Table S3. X-ray experimental data and refinement parameters for **12d**, **13b** and **24**.

Compound	**12d**	**13b**	**24**
CCDC number	2327009	2327011	2327014
Identification code	cyroxROH_auto	exp_226_auto	cyBn2Rlactone_auto
Empirical formula	$C_{14}H_{16}O_3$	$C_{12}H_{18}O_7S$	$C_{40}H_{40}O_6$
Formula weight	232.27	306.32	616.72
Temperature/K	105(2)	119(2)	100(2)
Crystal system	orthorhombic	monoclinic	triclinic
Space group	$P2_12_12_1$	$P2_1$	P1
a/Å	5.8349(3)	5.60610(10)	6.4634(2)
b/Å	10.8824(6)	10.52160(10)	9.4715(3)
c/Å	17.3475(10)	11.6074(2)	13.2656(4)
α/°	90	90	83.742(2)
β/°	90	103.364(2)	82.764(2)
γ/°	90	90	89.338(2)
Volume/Å^3	1101.52(11)	666.124(18)	800.82(4)
Z	4	2	1
ρ_{calc}g/cm^3	1.401	1.527	1.279
μ/mm^{-1}	0.792	2.459	0.680
F(000)	496.0	324.0	328.0
Crystal size/mm^3	0.345 × 0.128 × 0.072	0.155 × 0.092 × 0.086	0.228 × 0.096 × 0.075
Radiation	Cu Kα (λ = 1.54184)	Cu Kα (λ = 1.54184)	Cu Kα (λ = 1.54184)
2Θ range for data collection/°	9.594 to 159.192	7.828 to 158.416	6.756 to 158.644
Index ranges	-6 ≤ h ≤ 7, -13 ≤ k ≤ 13, -21 ≤ l ≤ 22	-7 ≤ h ≤ 6, -13 ≤ k ≤ 13, -14 ≤ l ≤ 14	-8 ≤ h ≤ 7, -12 ≤ k ≤ 11, -16 ≤ l ≤ 16
Reflections collected	12424	22263	28803
Independent reflections	2345 [R_{int} = 0.0615, R_{sigma} = 0.0323]	2831 [R_{int} = 0.0415, R_{sigma} = 0.0201]	6044 [R_{int} = 0.0413, R_{sigma} = 0.0285]
Data/restraints/parameters	2345/0/156	2831/1/181	6044/3/415
Goodness-of-fit on F^2	1.050	1.043	1.055
Final R indexes [I>=2σ (I)]	R_1 = 0.0518, wR_2 = 0.1286	R_1 = 0.0612, wR_2 = 0.1690	R_1 = 0.0339, wR_2 = 0.0916
Final R indexes [all data]	R_1 = 0.0534, wR_2 = 0.1297	R_1 = 0.0621, wR_2 = 0.1706	R_1 = 0.0358, wR_2 = 0.0936
Largest diff. peak/hole / e Å^{-3}	0.29/-0.35	1.46/-0.49	0.19/-0.19
Flack parameter	-0.18(18)	0.000(7)	0.06(8)

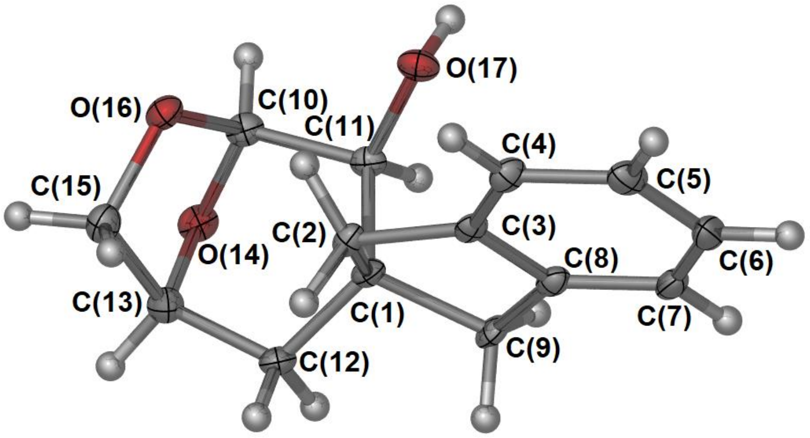

Figure S1. Perspective view of the labelled asymmetric unit showing the structure of **10d**, with the ellipsoids shown at 50% probability level. Carbon – grey, hydrogen – white and oxygen – red.

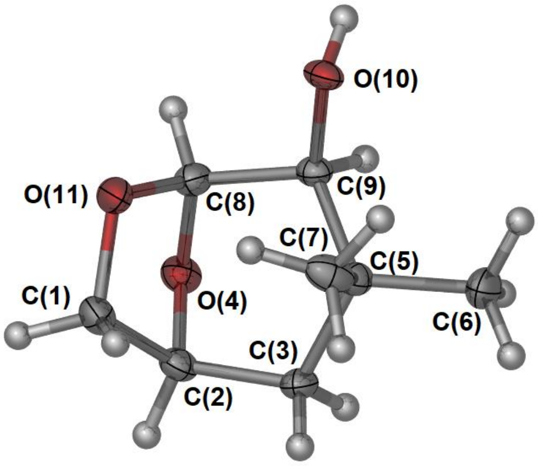

Figure S2. Perspective view of the labelled asymmetric unit showing the structure of **10e**, with the ellipsoids shown at 50% probability level. Carbon – grey, hydrogen – white and oxygen – red.

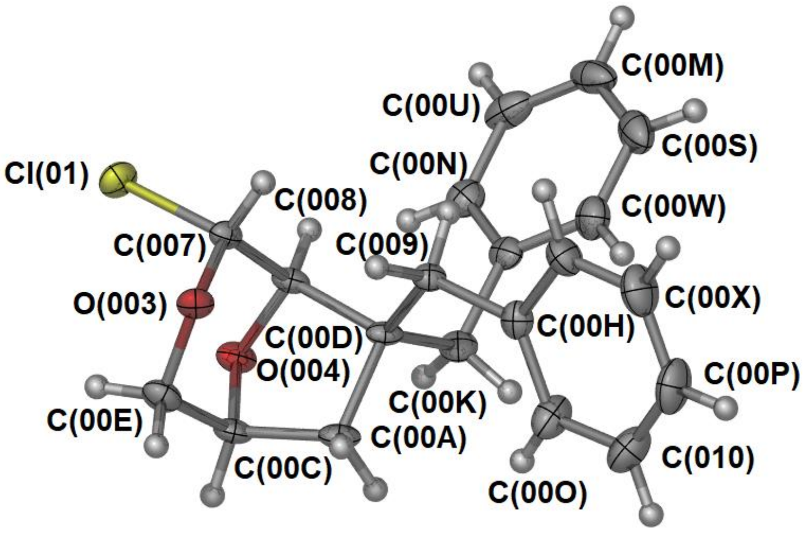

Figure S3. Perspective view of the labelled asymmetric unit of **11a** (showing one of the two molecules) with the ellipsoids shown at 50% probability level. Carbon – grey, hydrogen – white, oxygen – red, and chlorine – yellow.

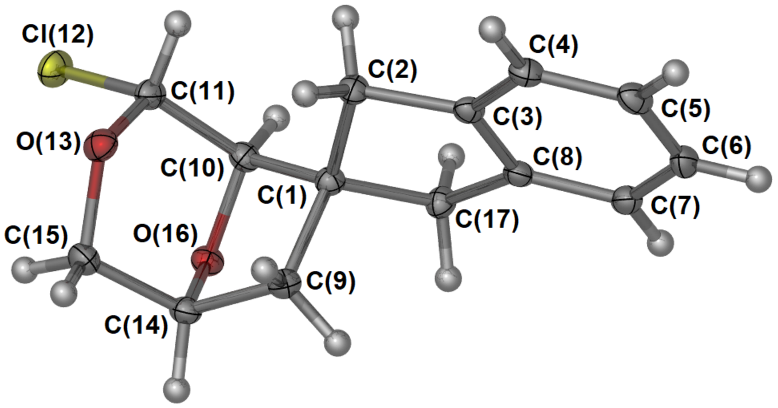

Figure S4. Perspective view of the labelled asymmetric unit showing the structure of **11d**, with the ellipsoids shown at 50% probability level. Carbon – grey, hydrogen – white, oxygen – red and chloride – yellow.

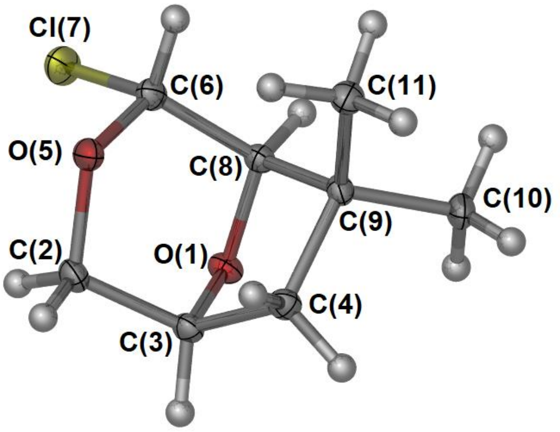

Figure S5. Perspective view of the labelled asymmetric unit showing the structure of **11e**, with the ellipsoids shown at 50% probability level. Carbon – grey, hydrogen – white, oxygen – red and chloride – yellow.

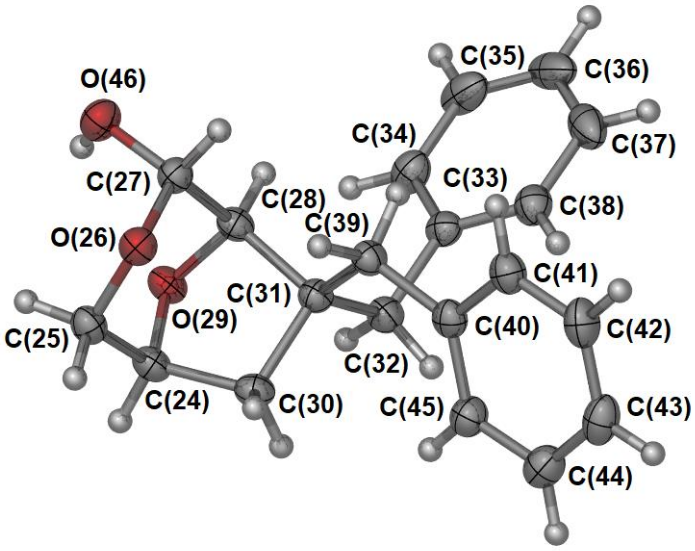

Figure S6. Perspective view of the labelled asymmetric unit of **12a** (showing one of the two molecules) with the ellipsoids shown at 50% probability level. Carbon – grey, hydrogen – white and oxygen – red.

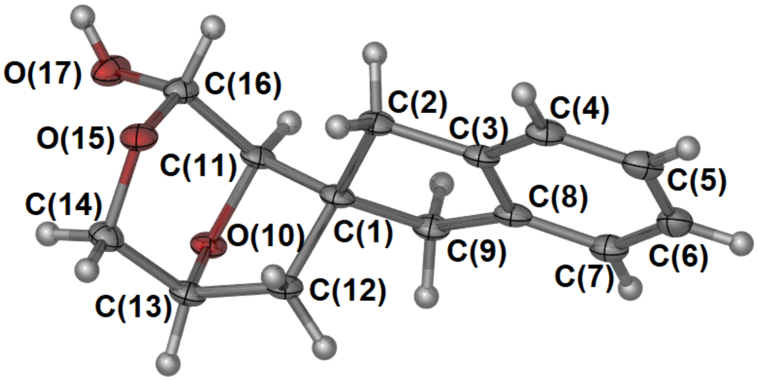

Figure S7. Perspective view of the labelled asymmetric unit showing the structure of **12d**, with the ellipsoids shown at 50% probability level. Carbon – grey, hydrogen – white and oxygen – red.

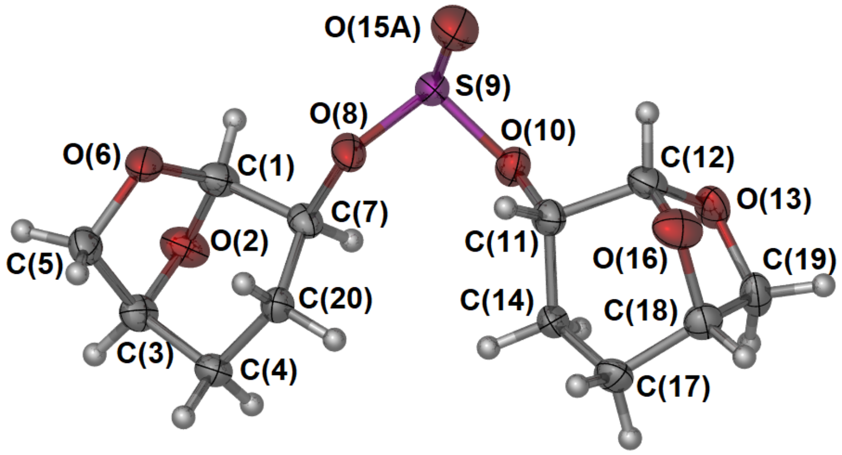

Figure S8. Perspective view of the labelled asymmetric unit showing the structure of **13b**, with the ellipsoids shown at 50% probability level. Carbon – grey, hydrogen – white, oxygen – red and sulfur – pink.

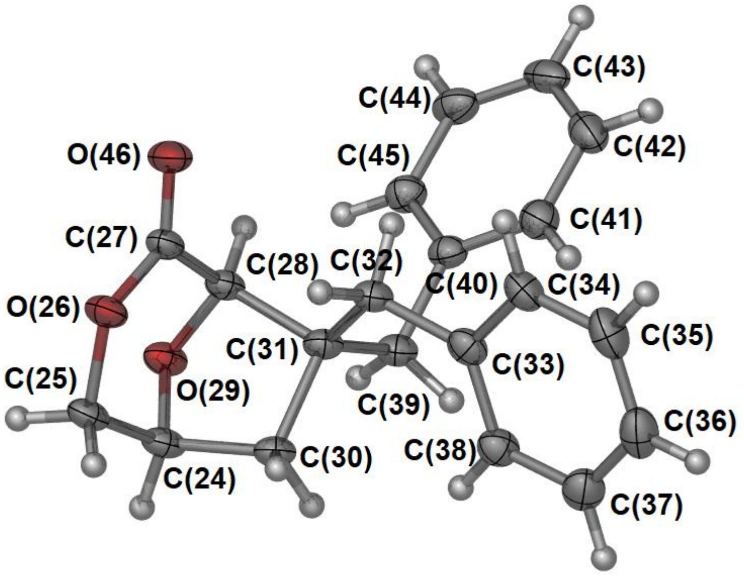

Figure S9. Perspective view of the labelled asymmetric unit of **24** (showing one of the two molecules), with the ellipsoids shown at 50% probability level. Carbon – grey, hydrogen – white and oxygen – red.

Copies of ^{1}H and ^{13}C{^{1}H} NMR spectra

^{1}H NMR of (1*S*,5*R*)-1',3'-Dihydro-6,8-dioxaspiro[bicyclo[3.2.1]octane-3,2'-inden]-4-one (9d)

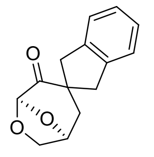

9d

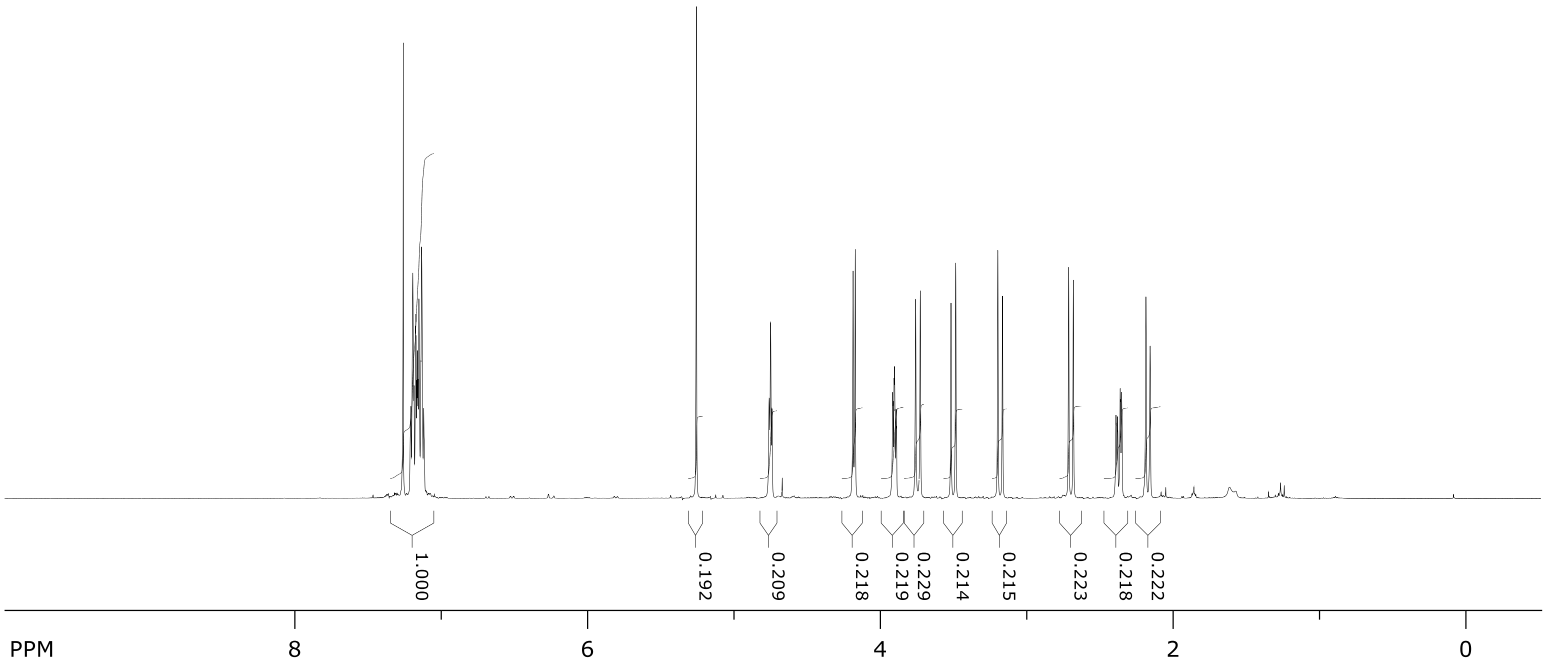

$^{13}C\{^{1}H\}$ NMR of (1*S*,5*R*)-1',3'-Dihydro-6,8-dioxaspiro[bicyclo[3.2.1]octane-3,2'-inden]-4-one (9d)

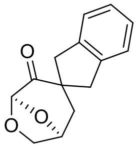

9d

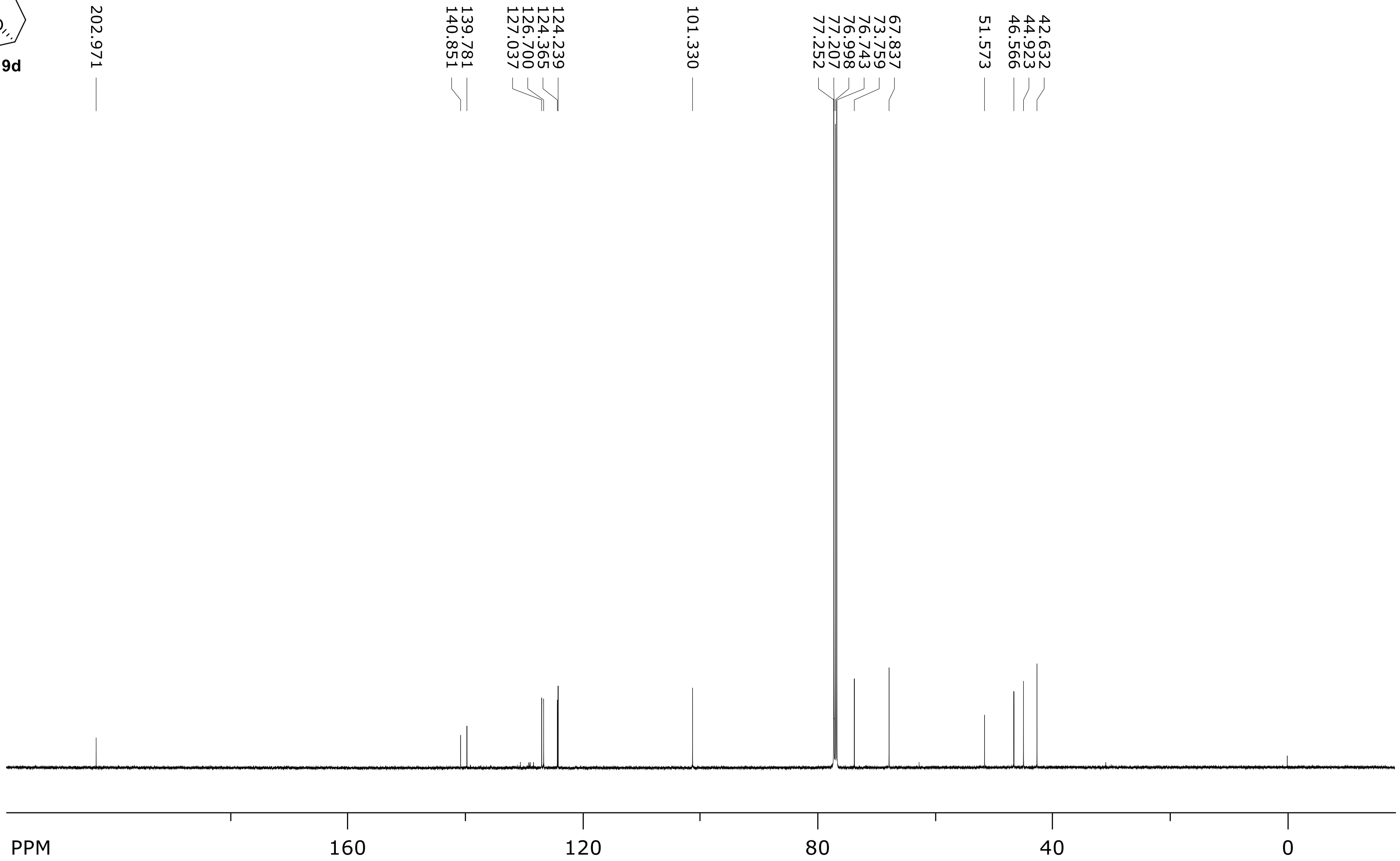

^{1}H NMR of (1*S*,5*R*)-3,3-Dimethyl-6,8-dioxabicyclo[3.2.1]octan-4-one (9e)

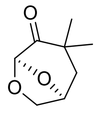

9e

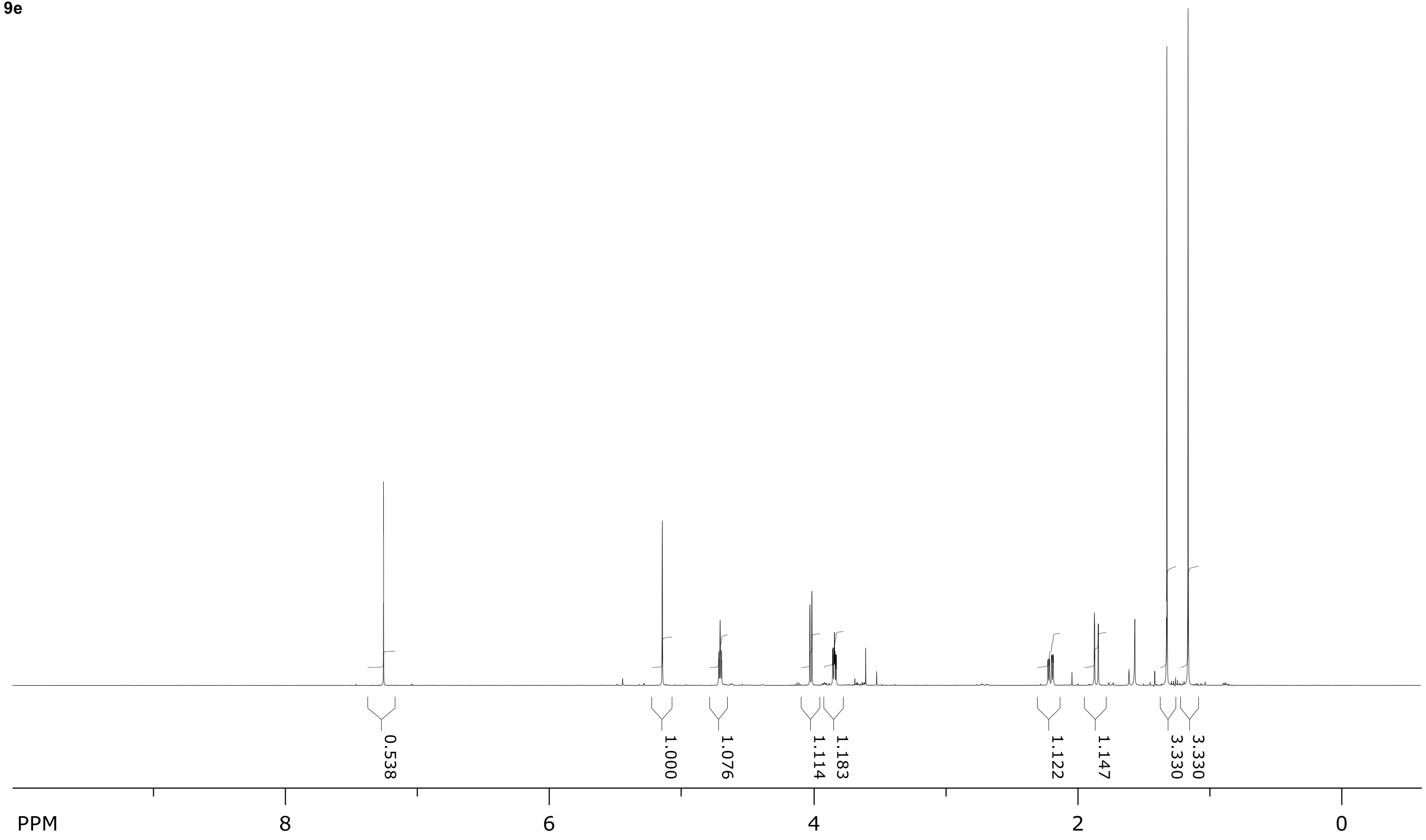

^{13}C{^{1}H} NMR of (1*S*,5*R*)-3,3-Dimethyl-6,8-dioxabicyclo[3.2.1]octan-4-one (9e)

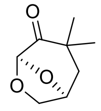

9e

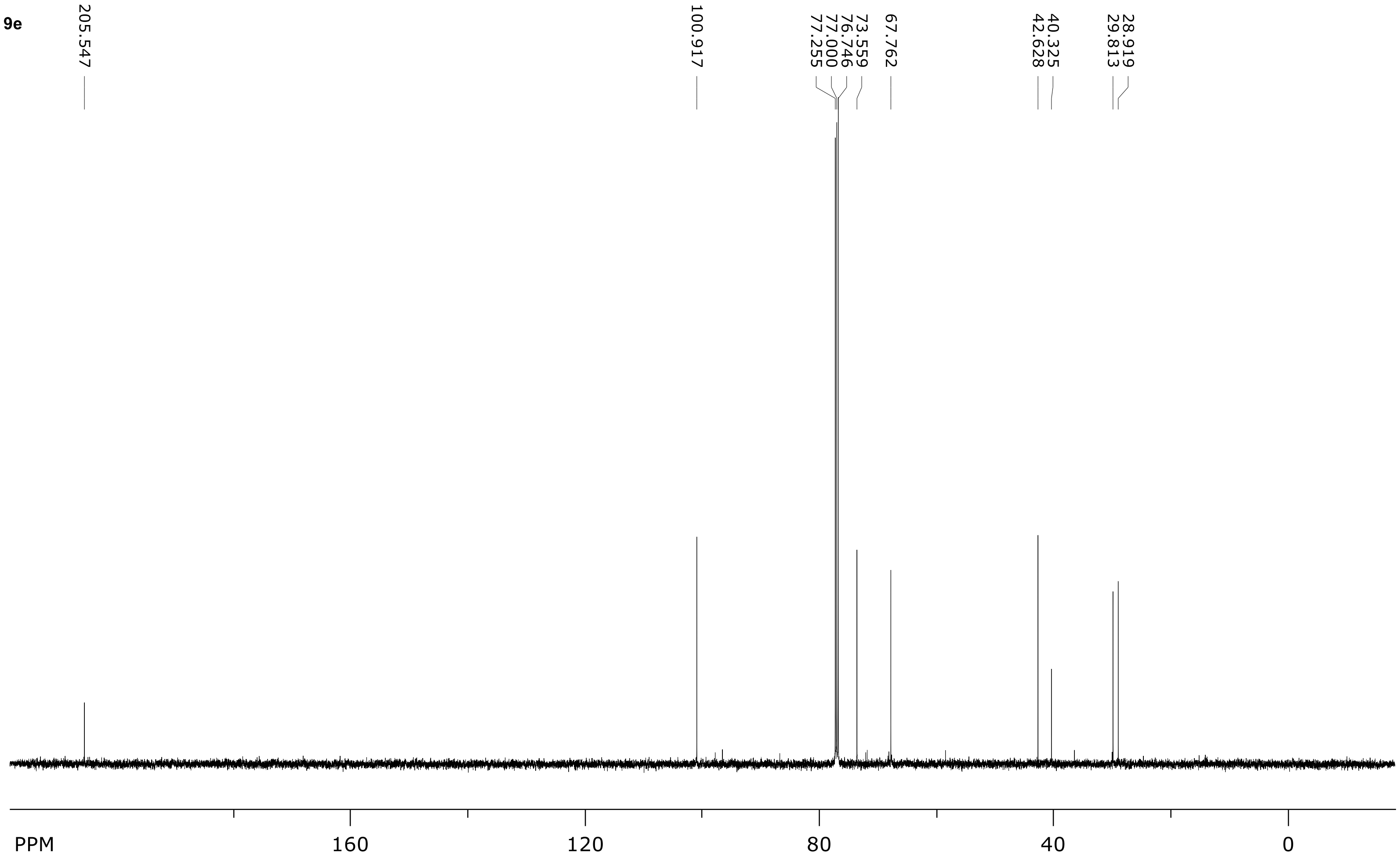

^{1}H NMR of (1*S*,5*R*)-3,3-Bis(4-methoxybenzyl)-6,8-dioxabicyclo[3.2.1]octan-4-one (9f)

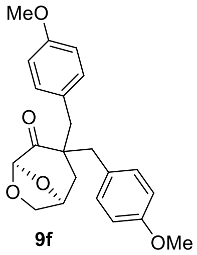

9f

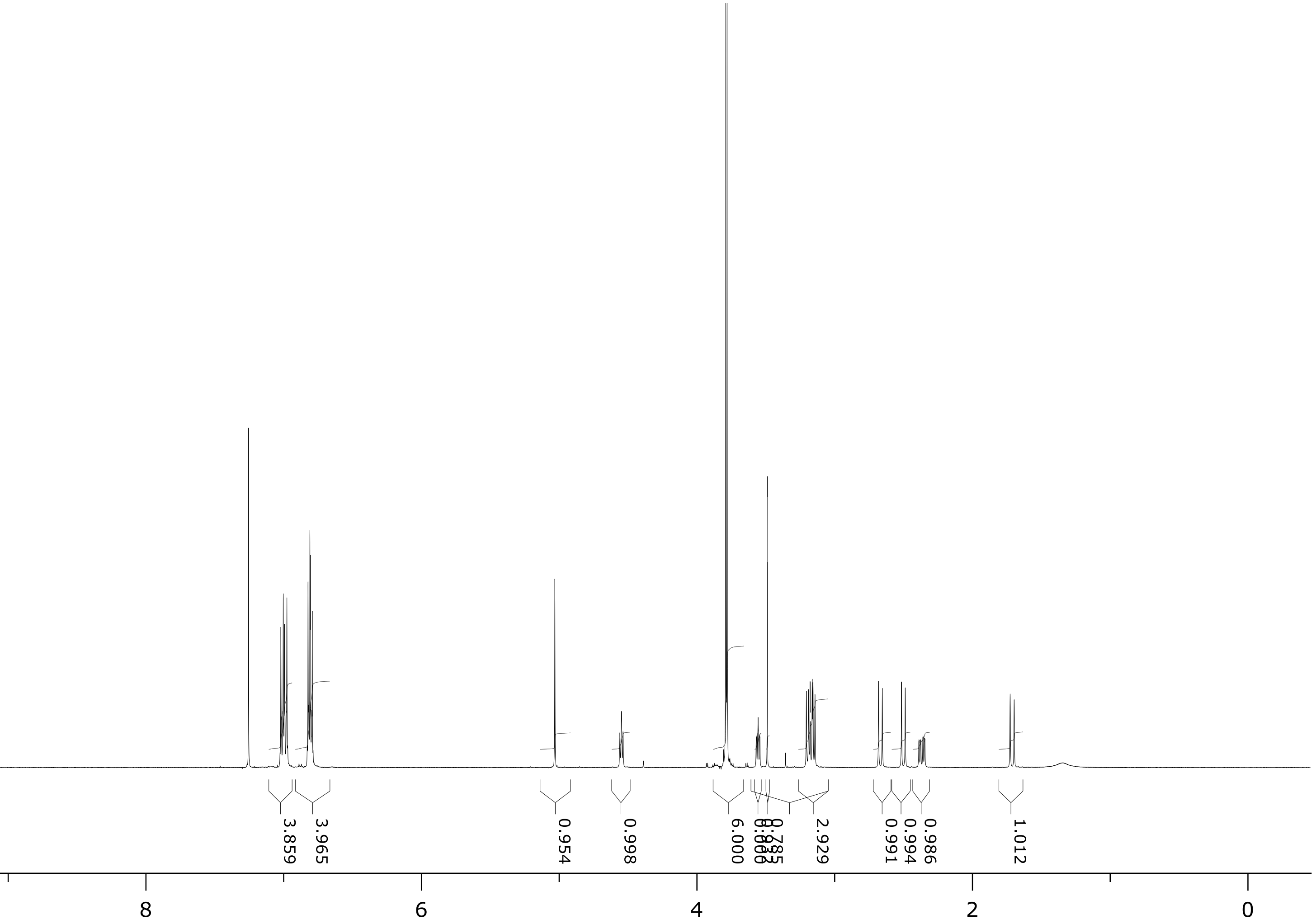

$^{13}C\{^{1}H\}$ NMR of (1*S*,5*R*)-3,3-Bis(4-methoxybenzyl)-6,8-dioxabicyclo[3.2.1]octan-4-one (9f)

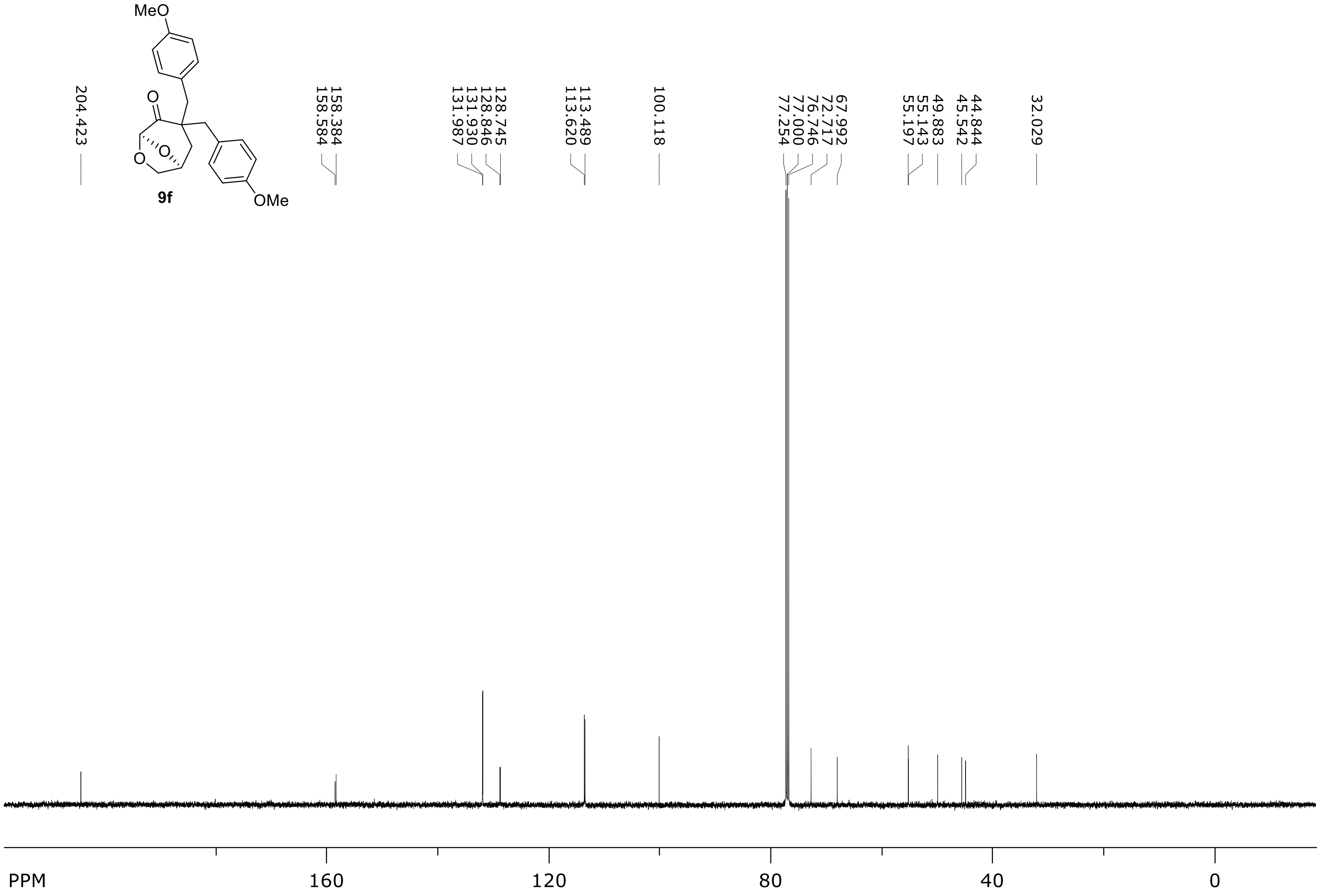

^{1}H NMR of (1*S*,4*S*,5*R*)-1',3'-Dihydro-6,8-dioxaspiro[bicyclo[3.2.1]octane-3,2'-inden]-4-ol (10d)

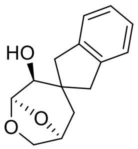

10d

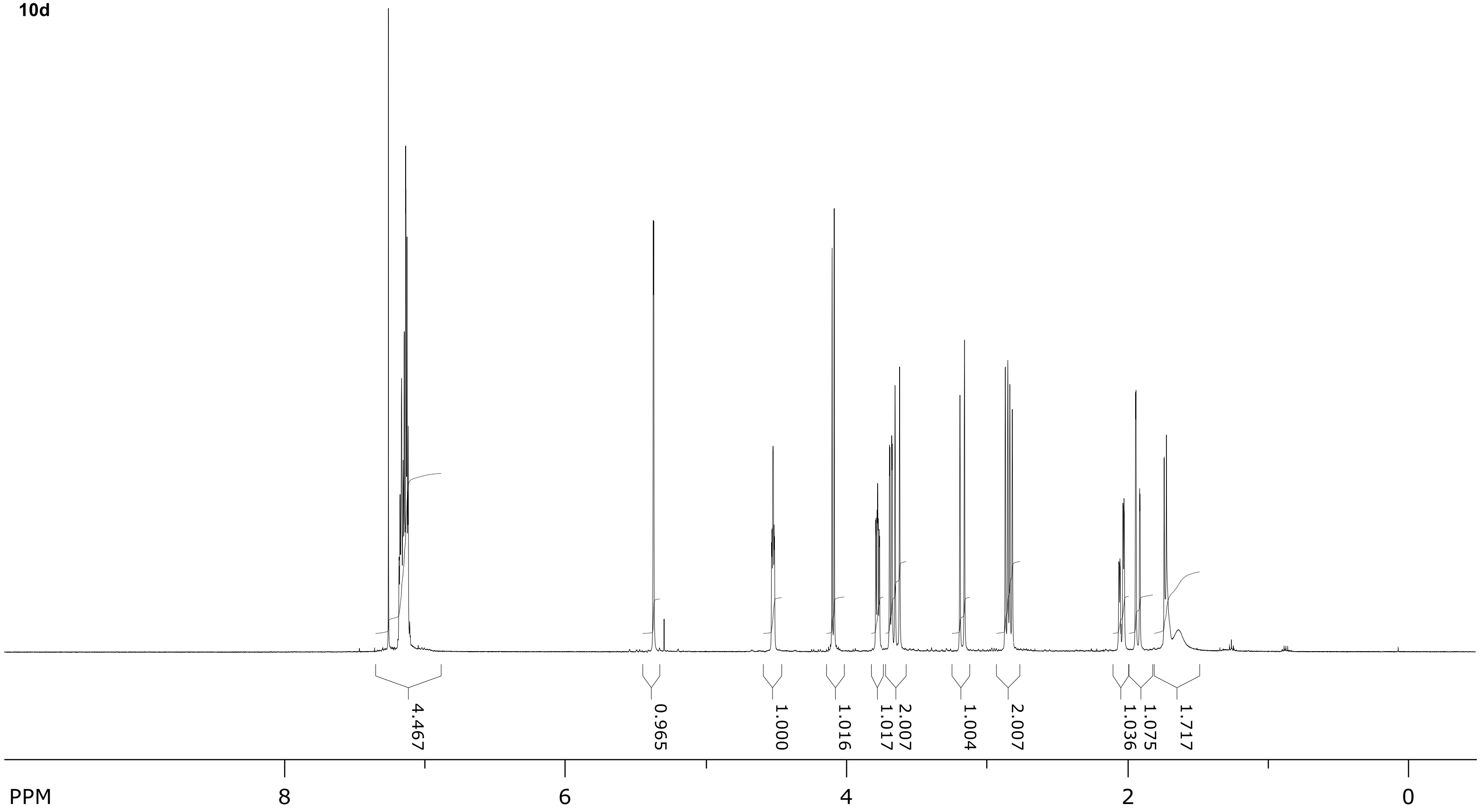

$^{13}C\{^1H\}$ NMR of (1*S*,4*S*,5*R*)-1',3'-Dihydro-6,8-dioxaspiro[bicyclo[3.2.1]octane-3,2'-inden]-4-ol (10d)

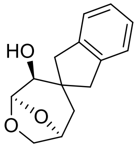

10d

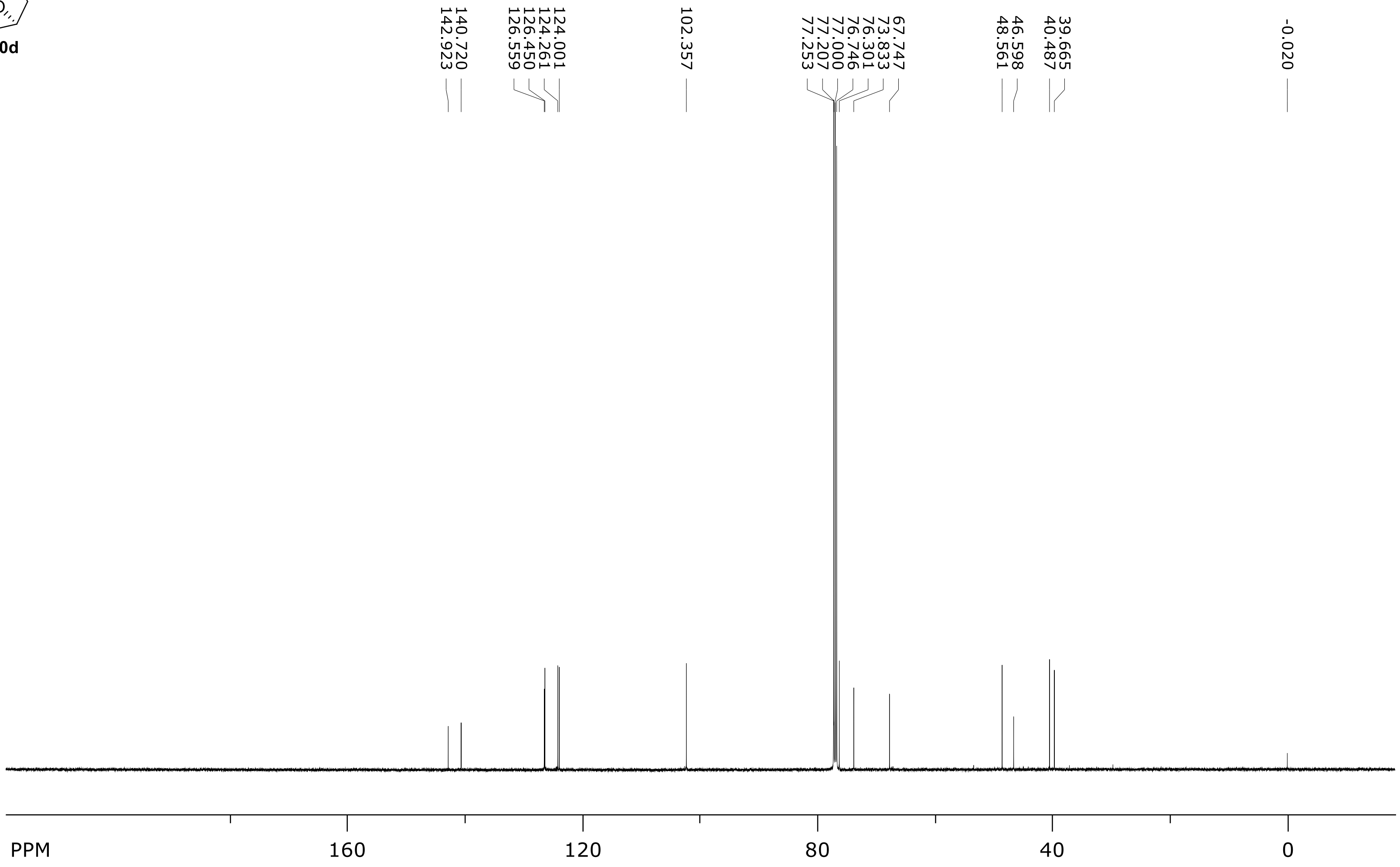

^{1}H NMR of (1*S*,4*S*,5*R*)-3,3-Dimethyl-6,8-dioxabicyclo[3.2.1]octan-4-ol (10e)

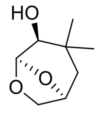

10e

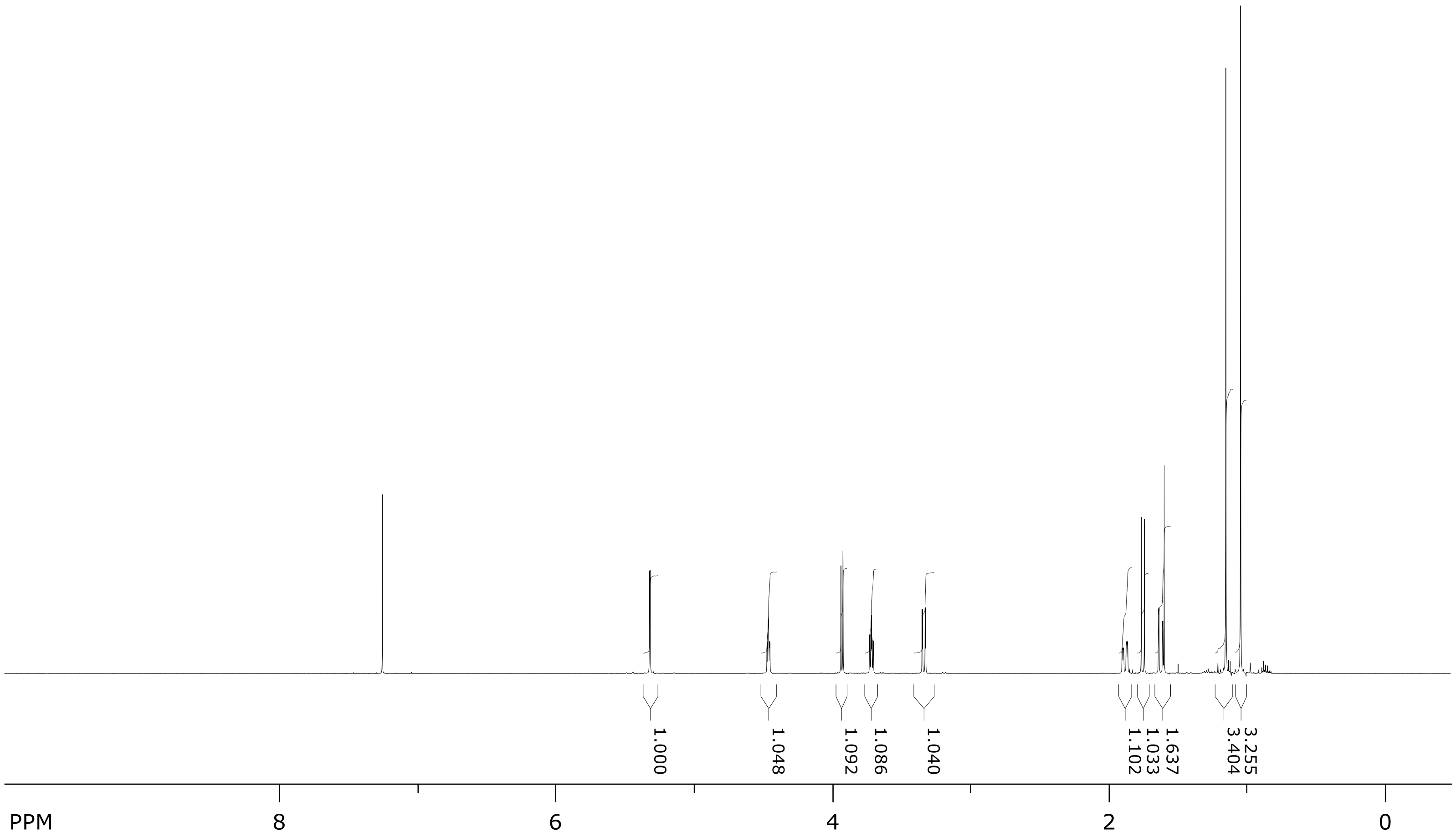

$^{13}C\{^1H\}$ **NMR of (1*S*,4*S*,5*R*)-3,3-Dimethyl-6,8-dioxabicyclo[3.2.1]octan-4-ol (10e).**

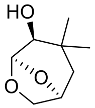

10e

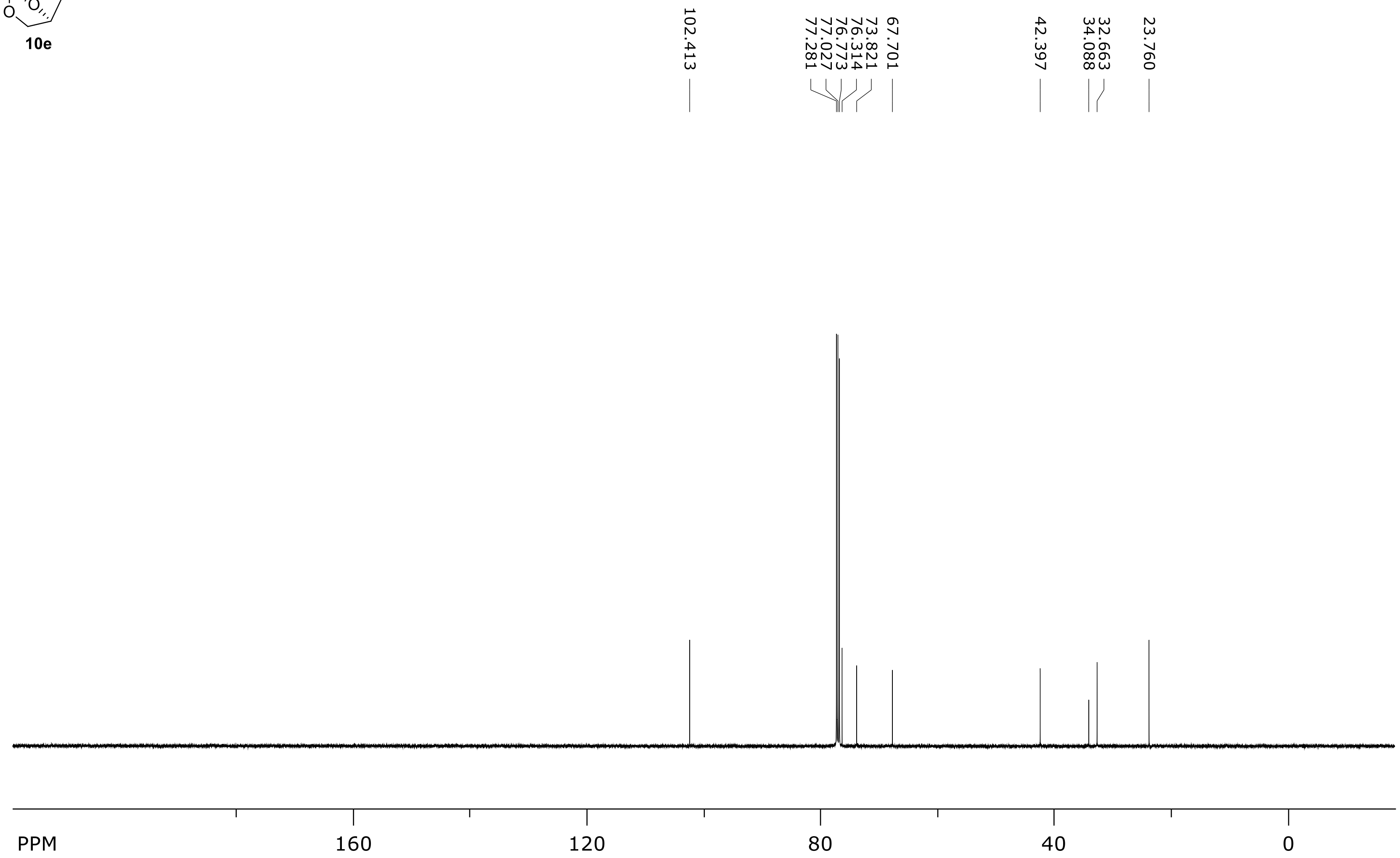

^{1}H NMR of (1*S*,4*S*,5*R*)-3,3-Bis(4-methoxybenzyl)-6,8-dioxabicyclo[3.2.1]octan-4-ol (10f)

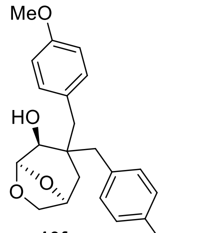

10f

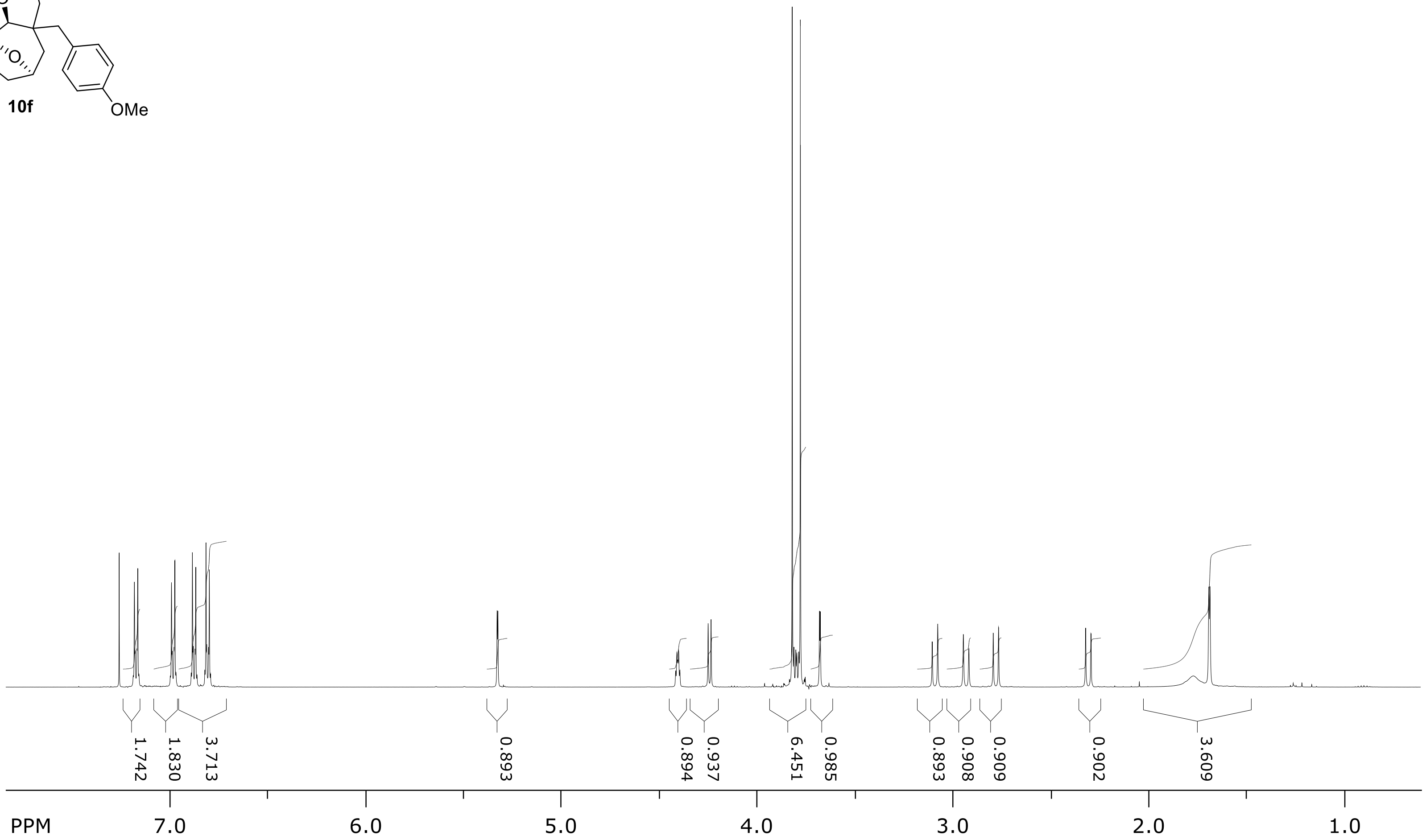

$^{13}C\{^{1}H\}$ NMR of (1*S*,4*S*,5*R*)-3,3-Bis(4-methoxybenzyl)-6,8-dioxabicyclo[3.2.1]octan-4-ol (10f)

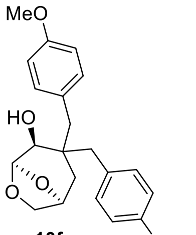

10f

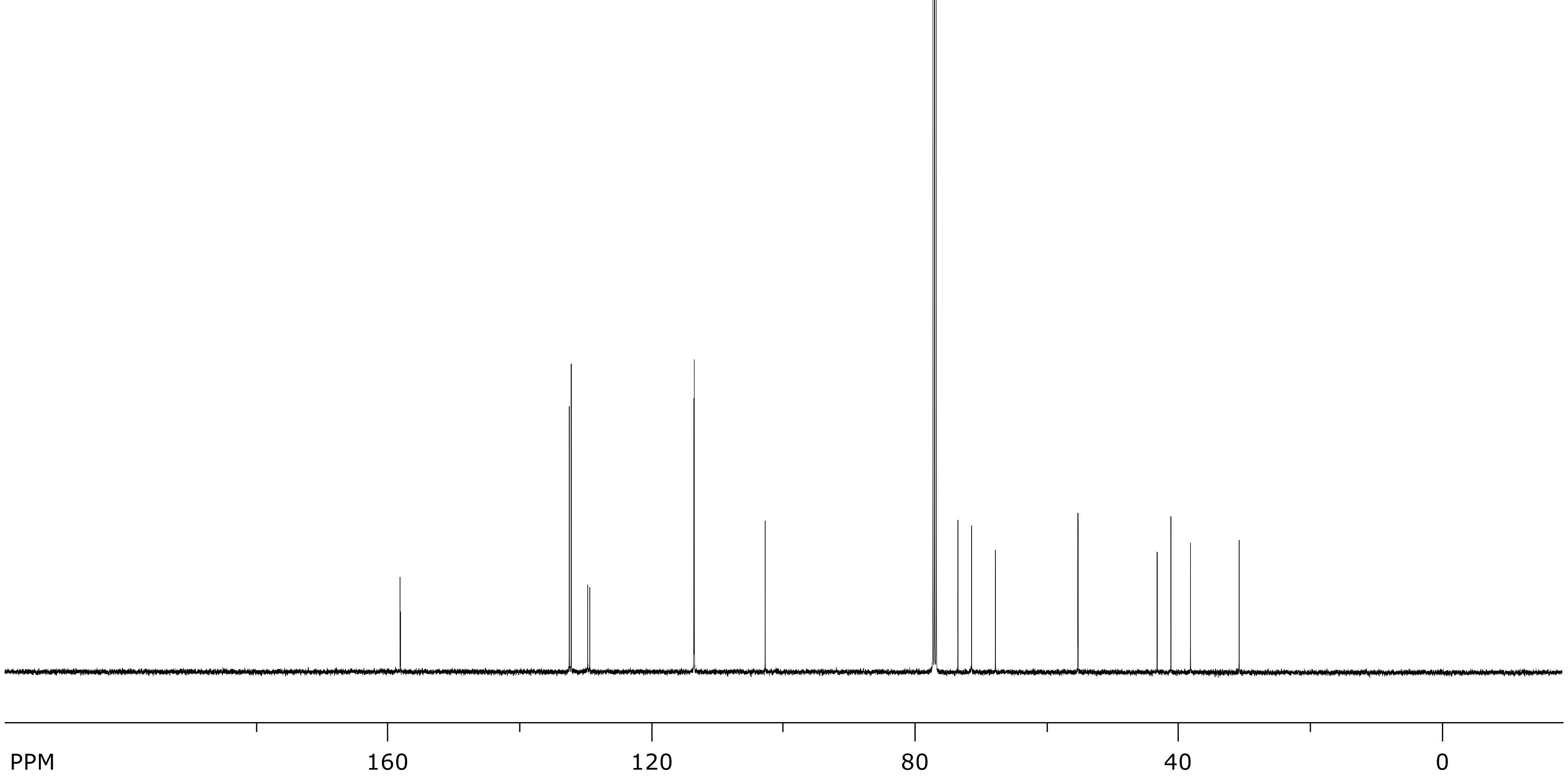

^{1}H NMR of (1*R*,2*R*,5*S*)-7,7-dibenzyl-2-chloro-3,8-dioxabicyclo[3.2.1]octane (11a).

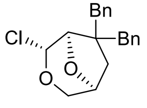

11a

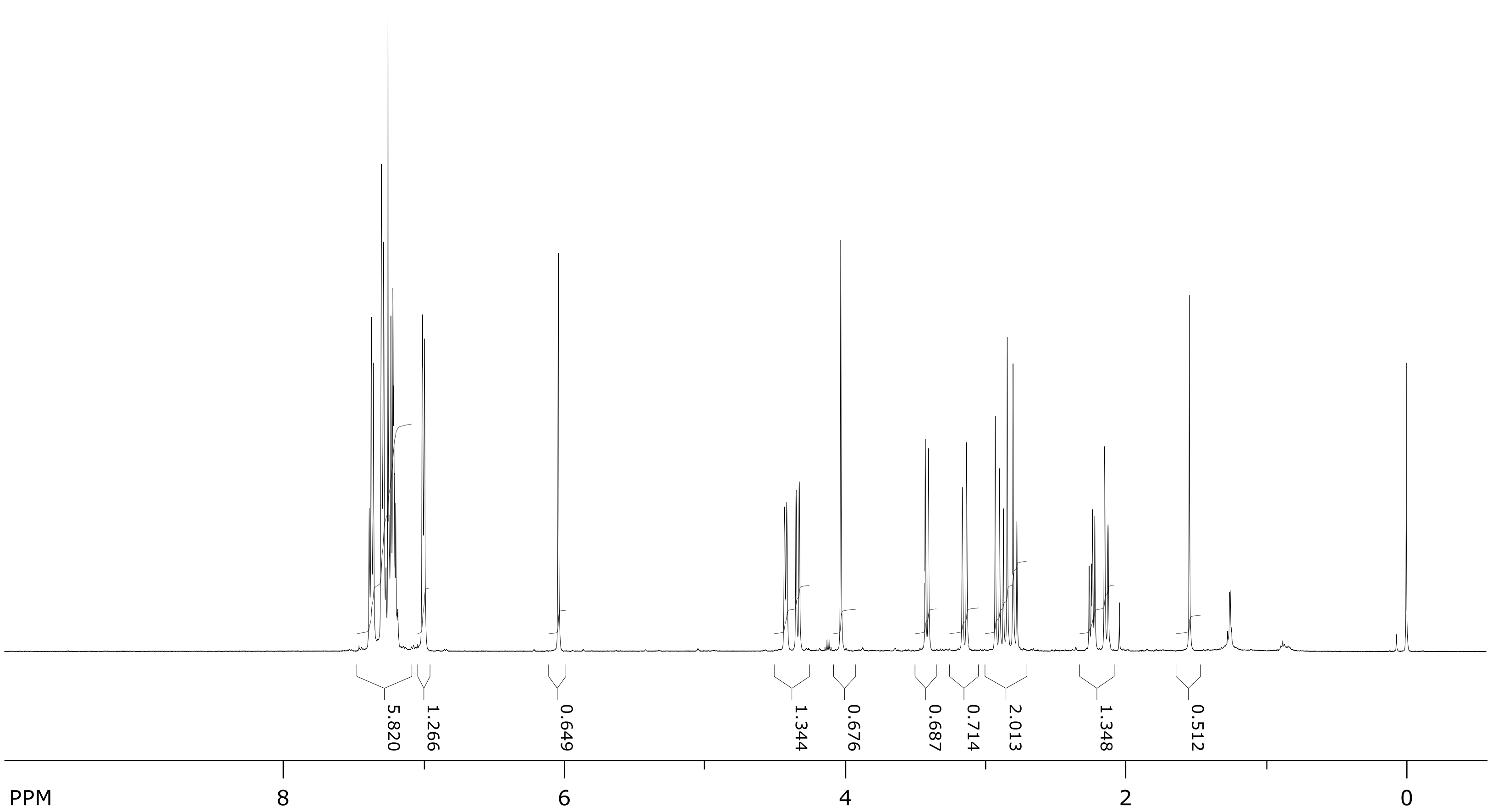

$^{13}C\{^{1}H\}$ NMR of (1*R*,2*R*,5*S*)-7,7-dibenzyl-2-chloro-3,8-dioxabicyclo[3.2.1]octane (11a).

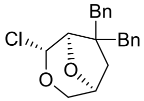

11a

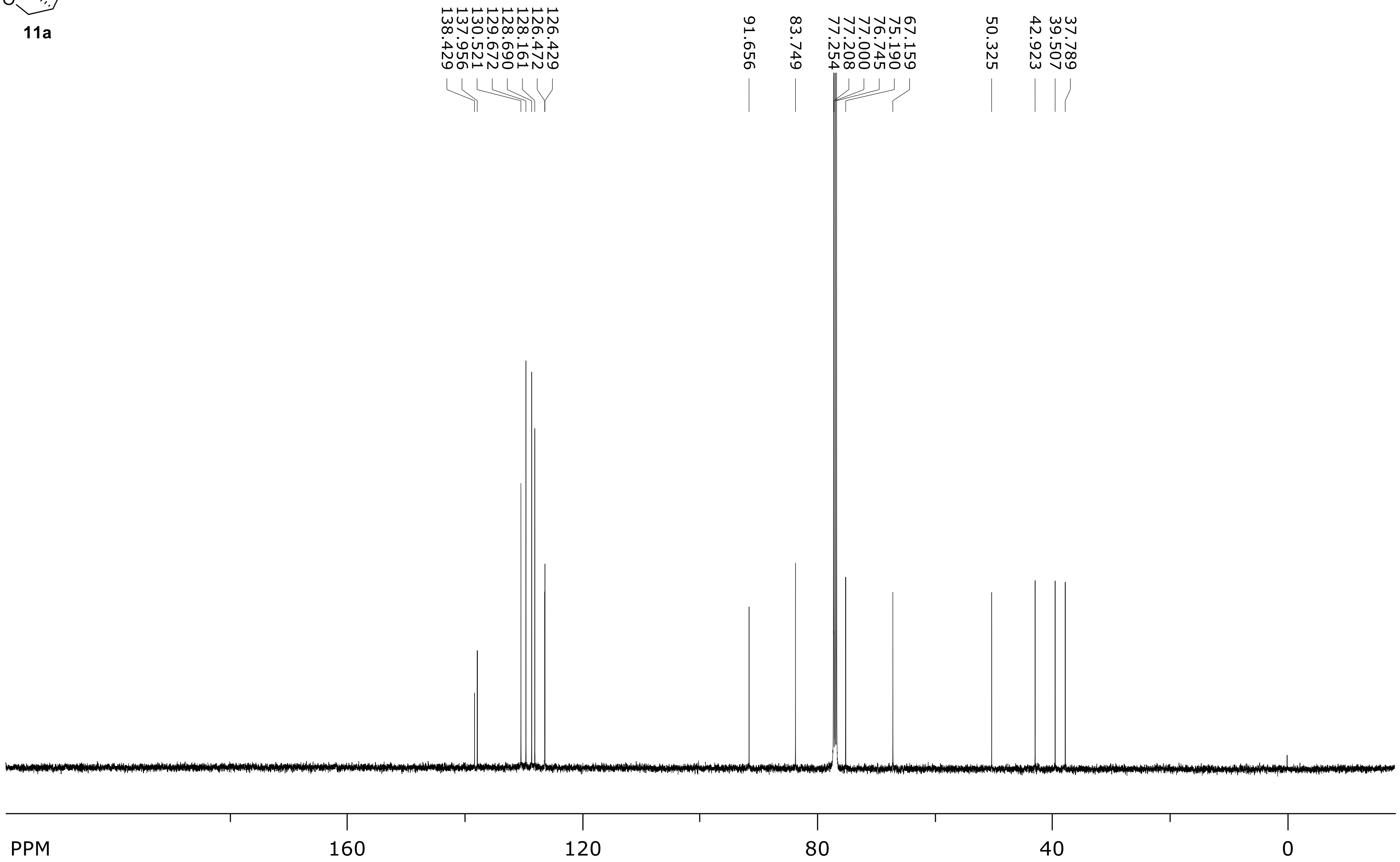

^{1}H NMR of (1*R*,2*R*,5*S*)-2-Chloro-3,8-dioxabicyclo[3.2.1]octane (11b) contaminated with 14

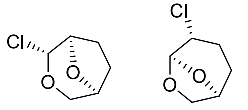

11b **14**

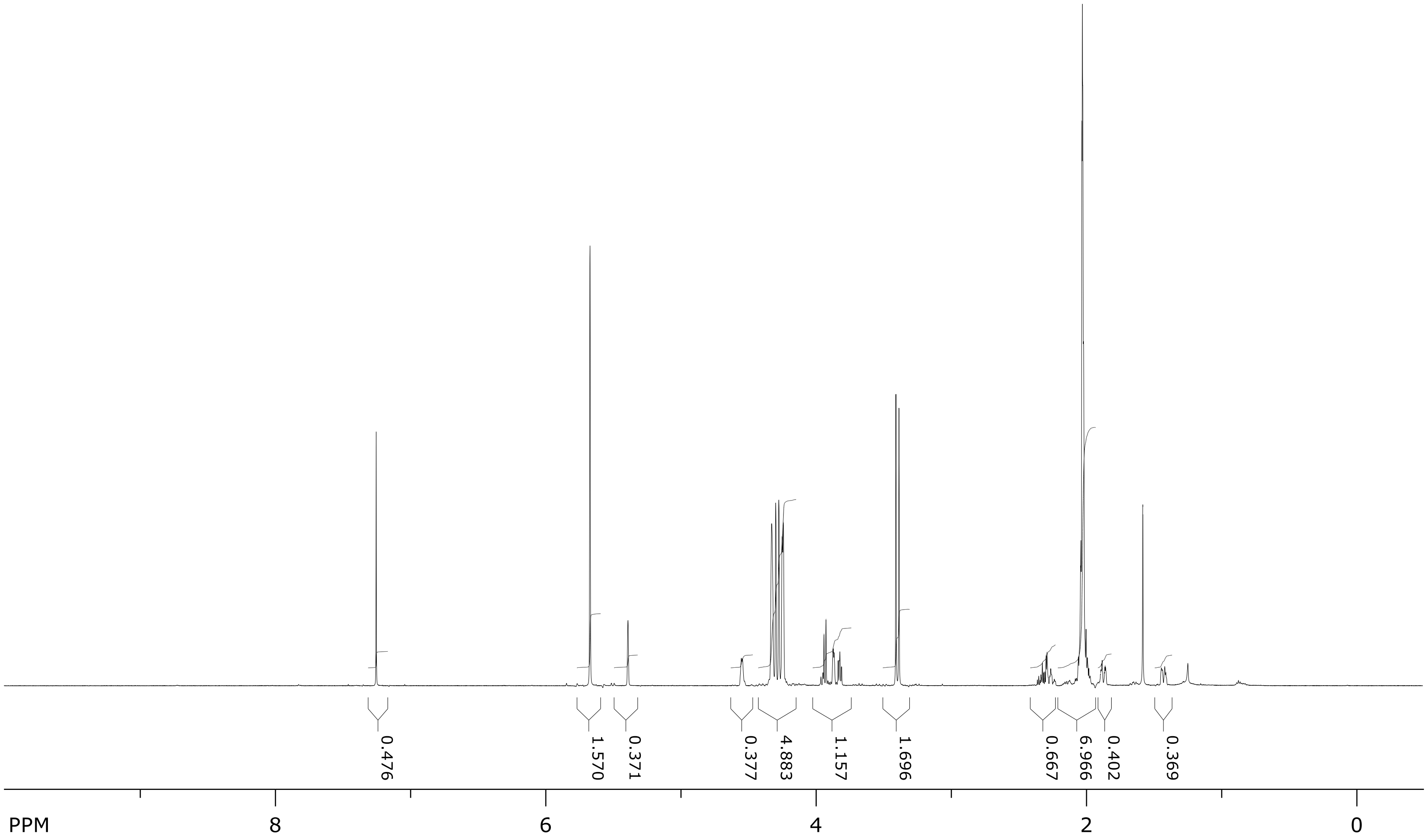

^{13}C{^{1}H} NMR of (1*R*,2*R*,5*S*)-2-Chloro-3,8-dioxabicyclo[3.2.1]octane (11b) contaminated with 14

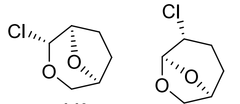

11b **14**

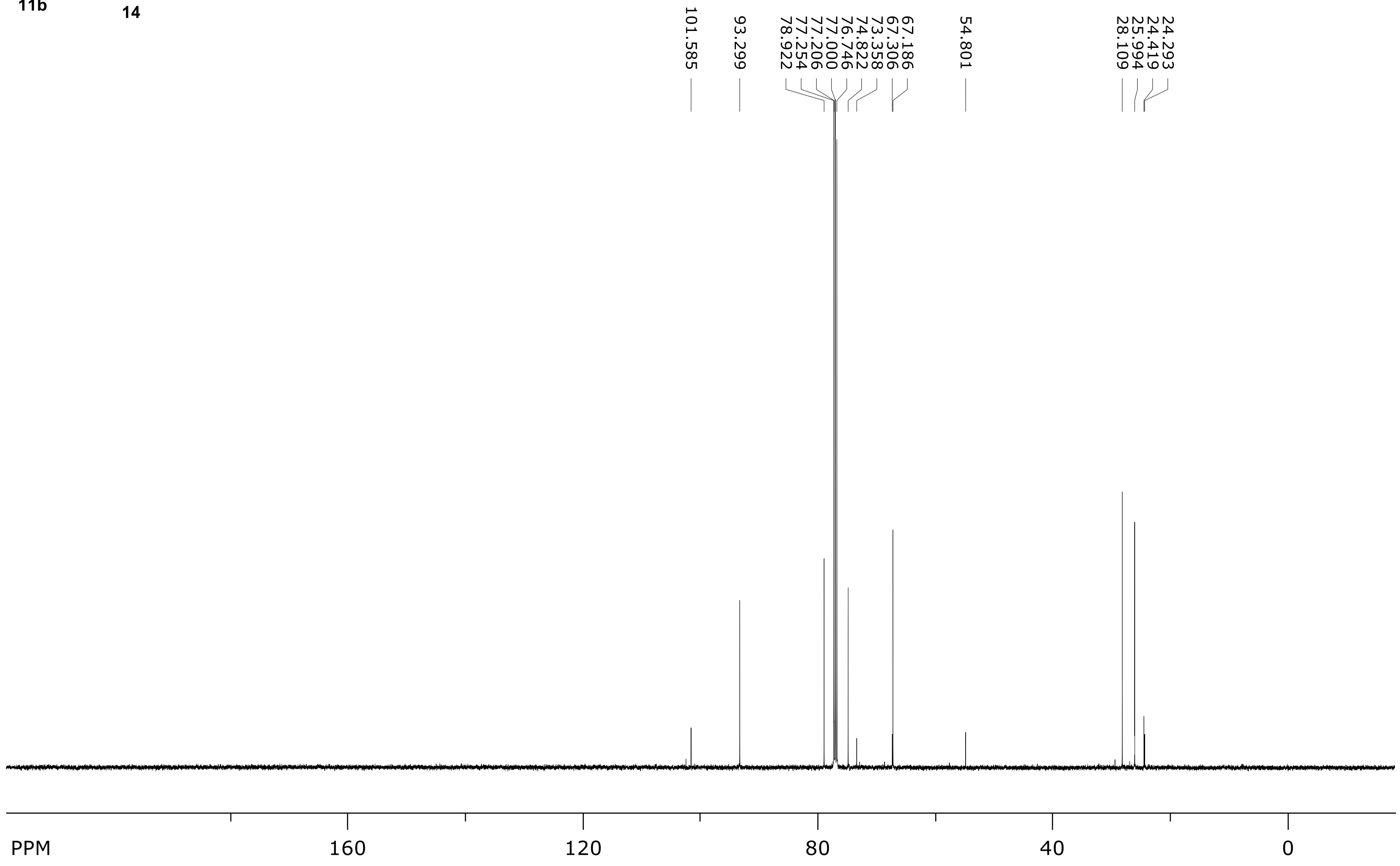

^{1}H NMR of (1*R*,2*R*,5*S*)-2-Chloro-7,7-bis(2-methylbenzyl)-3,8-dioxabicyclo[3.2.1]octane (11c)

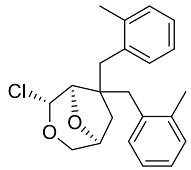

11c

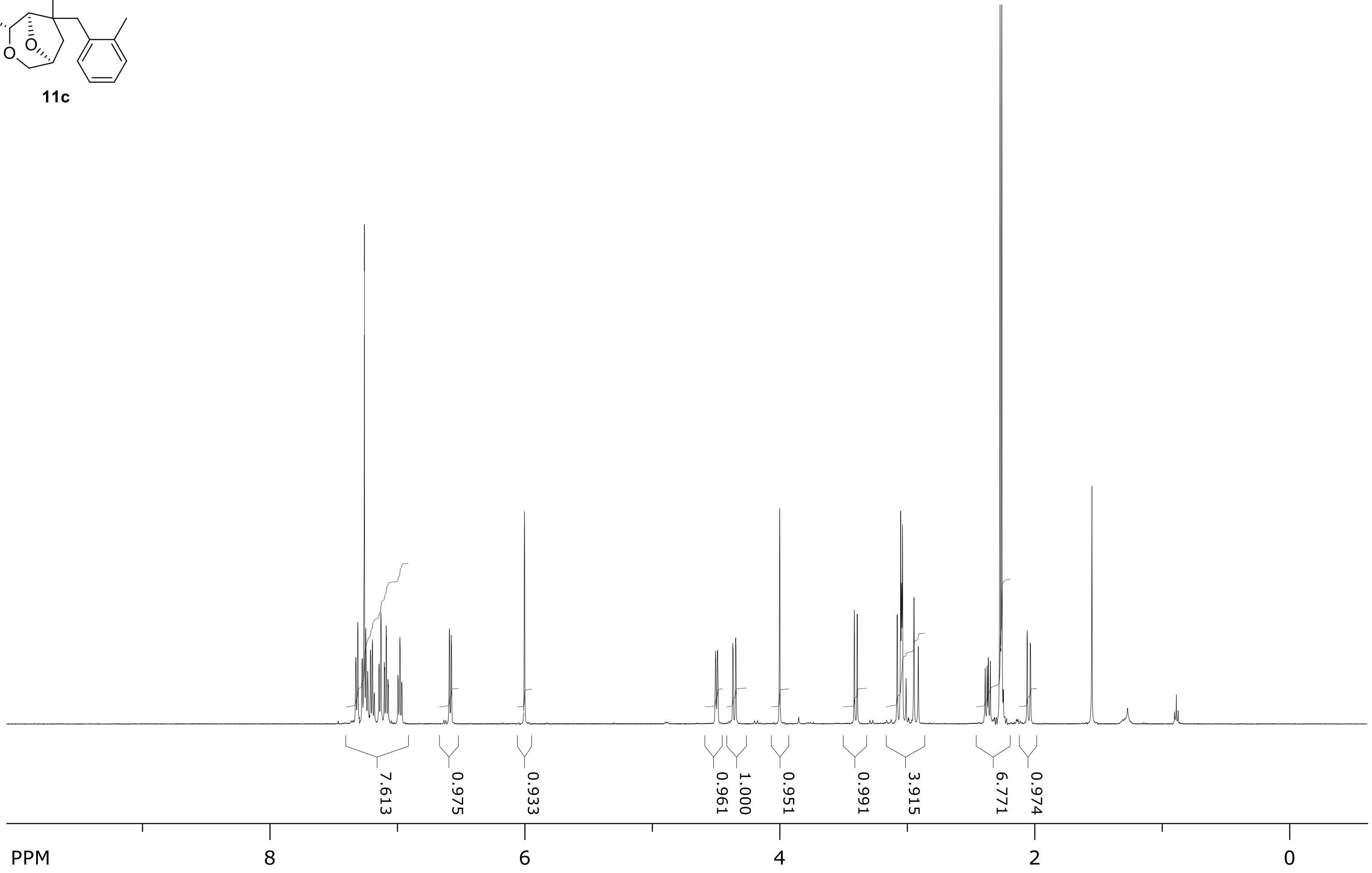

$^{13}C\{^1H\}$ NMR of (1*R*,2*R*,5*S*)-2-Chloro-7,7-bis(2-methylbenzyl)-3,8-dioxabicyclo[3.2.1]octane (11c)

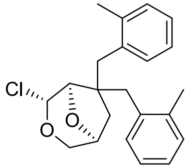

11c

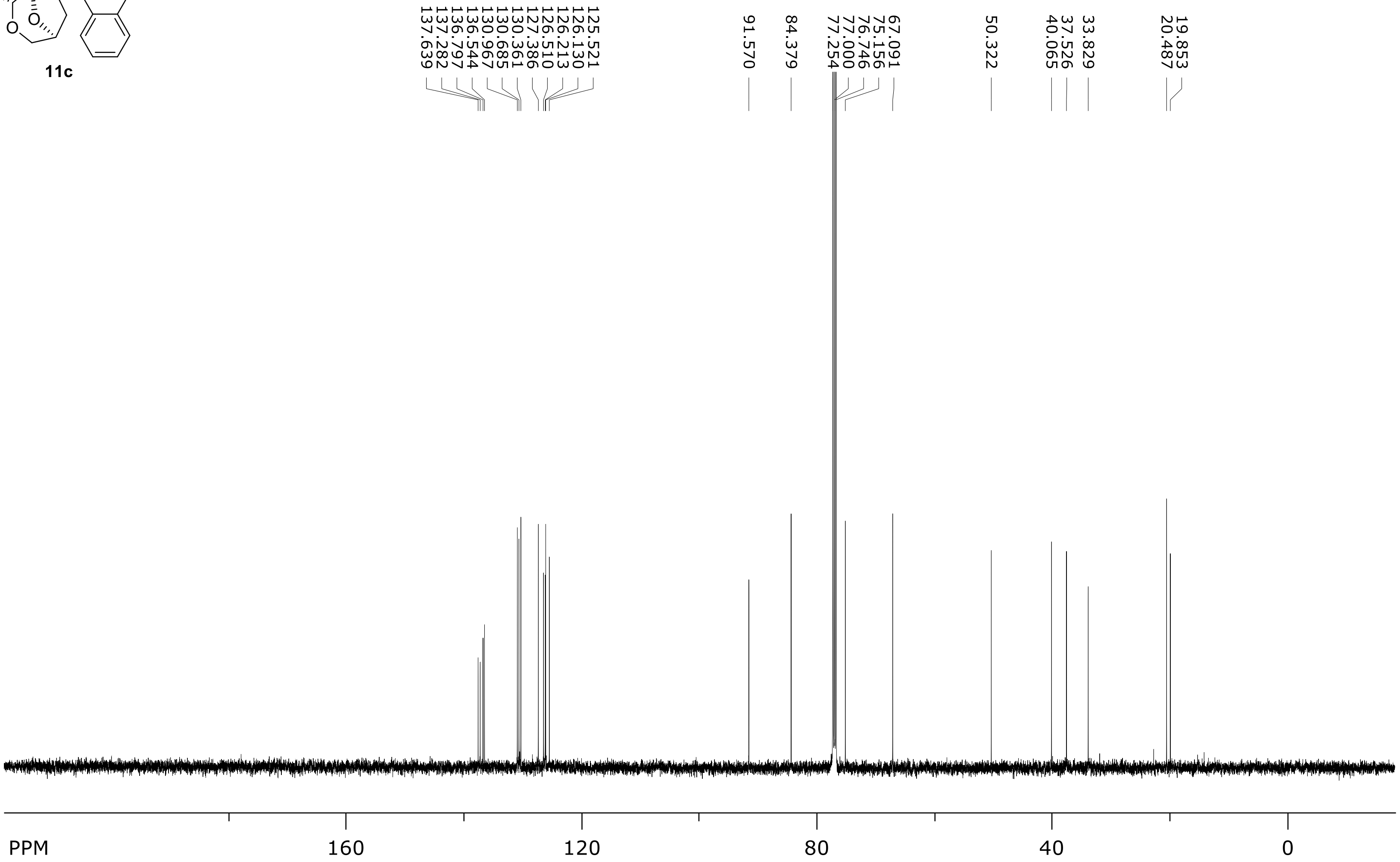

^{1}H NMR of (1*S*,4*R*,5*R*)-4-Chloro-1',3'-dihydro-3,8-dioxaspiro[bicyclo[3.2.1]octane-6,2'-indene] (11d)

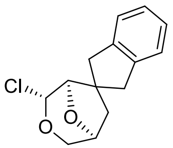

11d

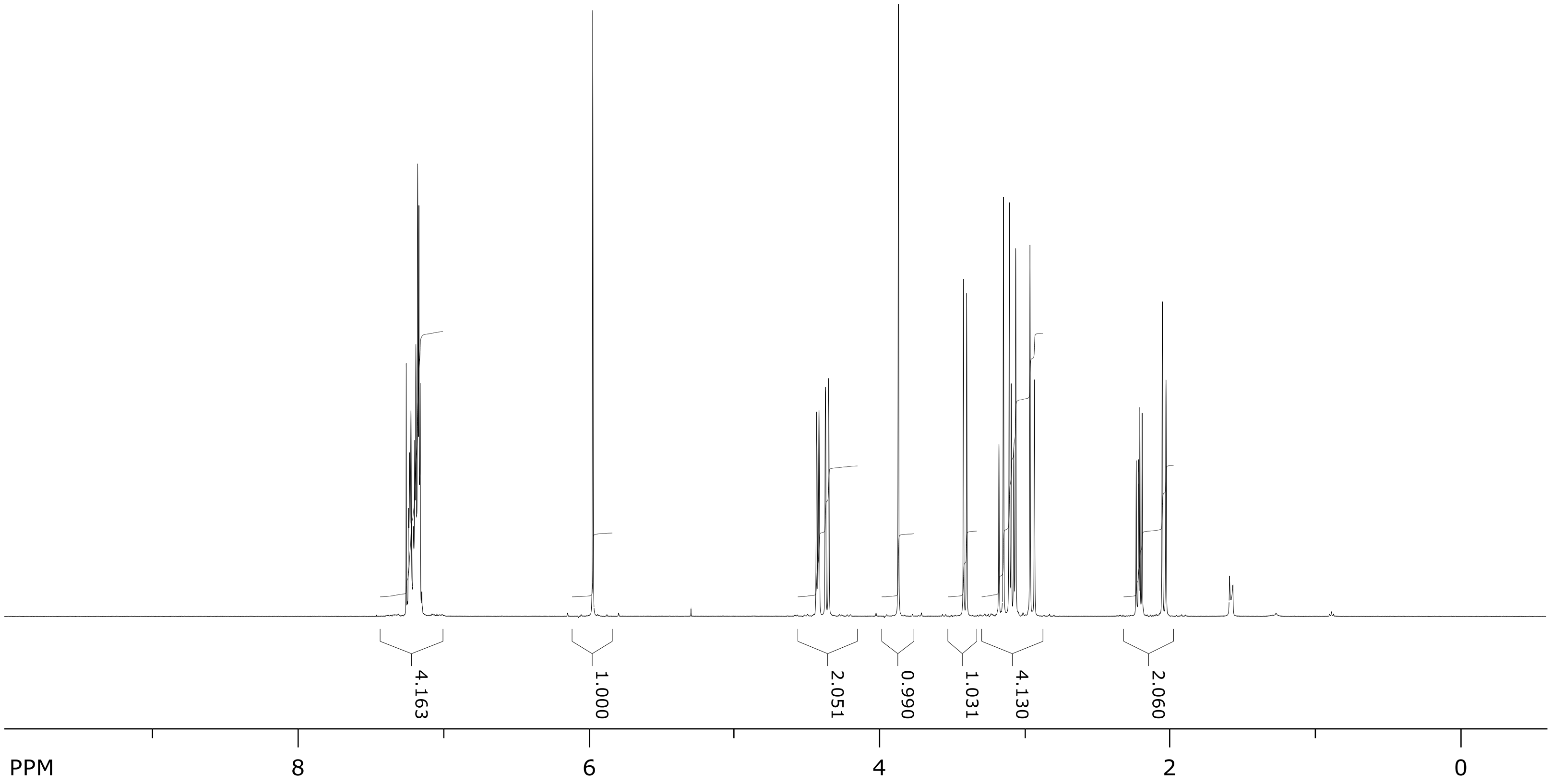

$^{13}C\{^1H\}$ NMR of (1*S*,4*R*,5*R*)-4-Chloro-1',3'-dihydro-3,8-dioxaspiro[bicyclo[3.2.1]octane-6,2'-indene] (11d)

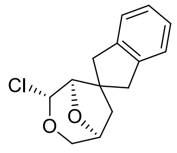

11d

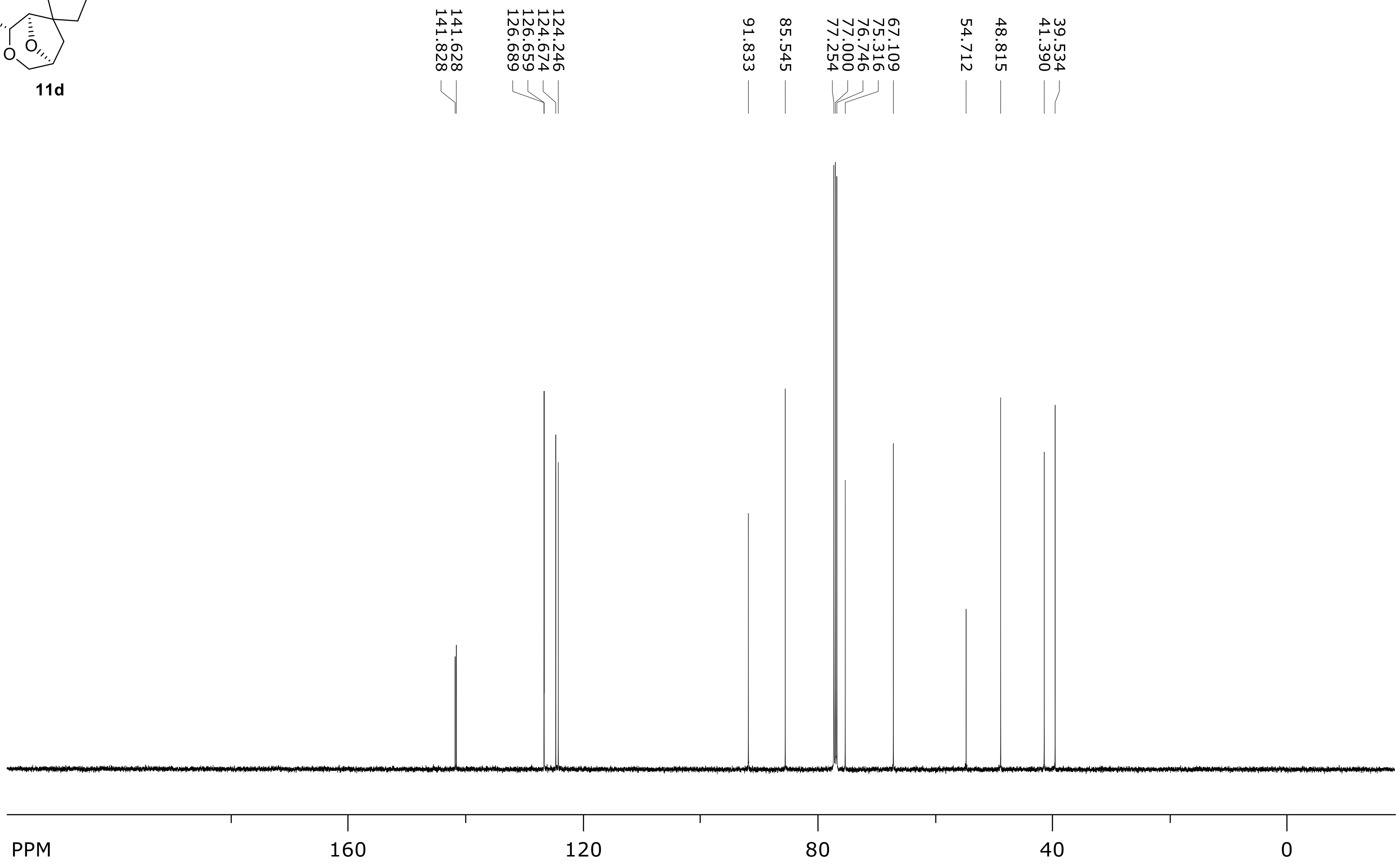

^{1}H NMR of (1R,2R,5S)-2-Chloro-7,7-dimethyl-3,8-dioxabicyclo[3.2.1]octane (11e)

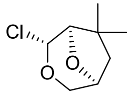
11e

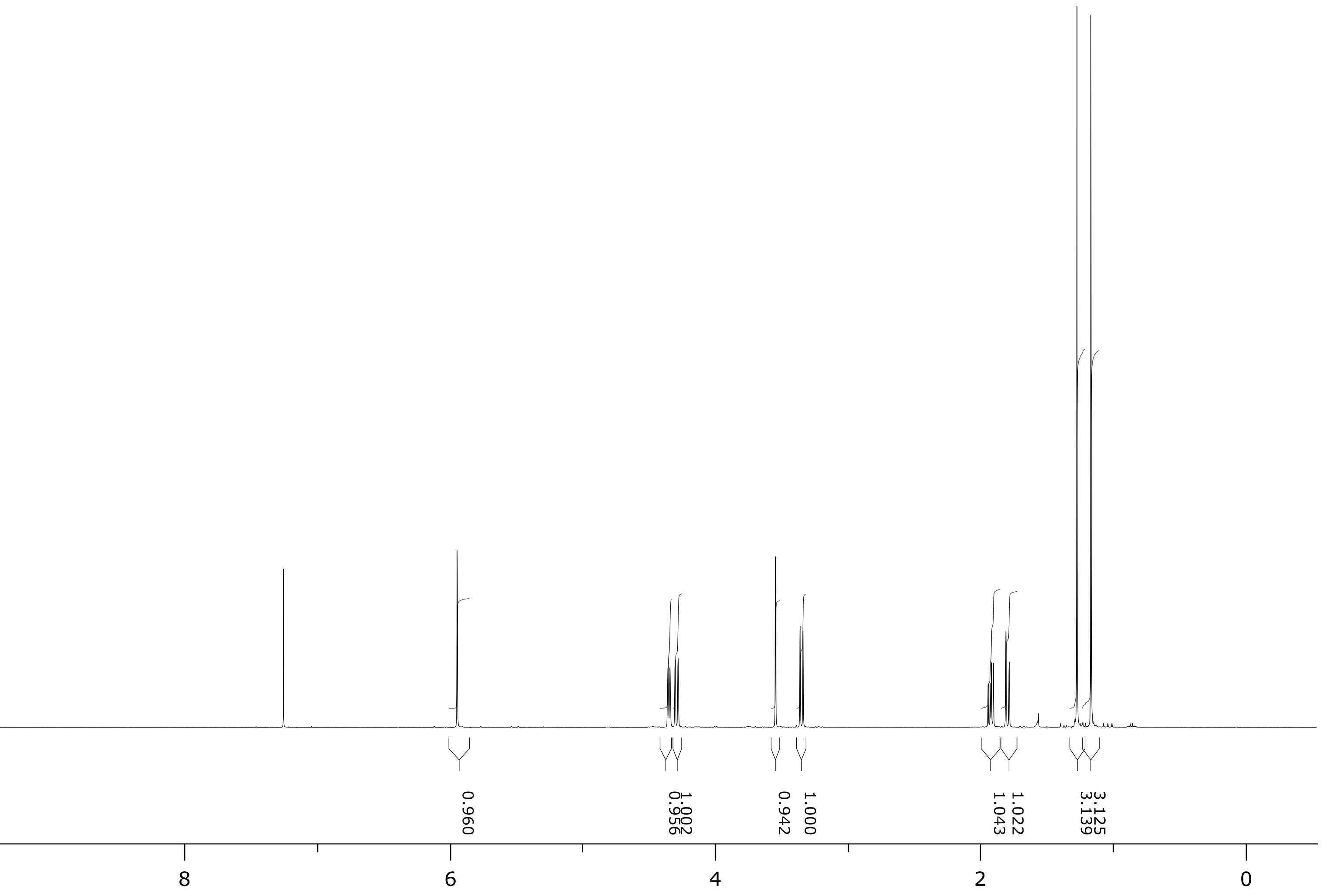

$^{13}C\{^{1}H\}$ **NMR of (1R,2R,5S)-2-Chloro-7,7-dimethyl-3,8-dioxabicyclo[3.2.1]octane (11e)**

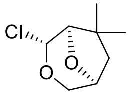

11e

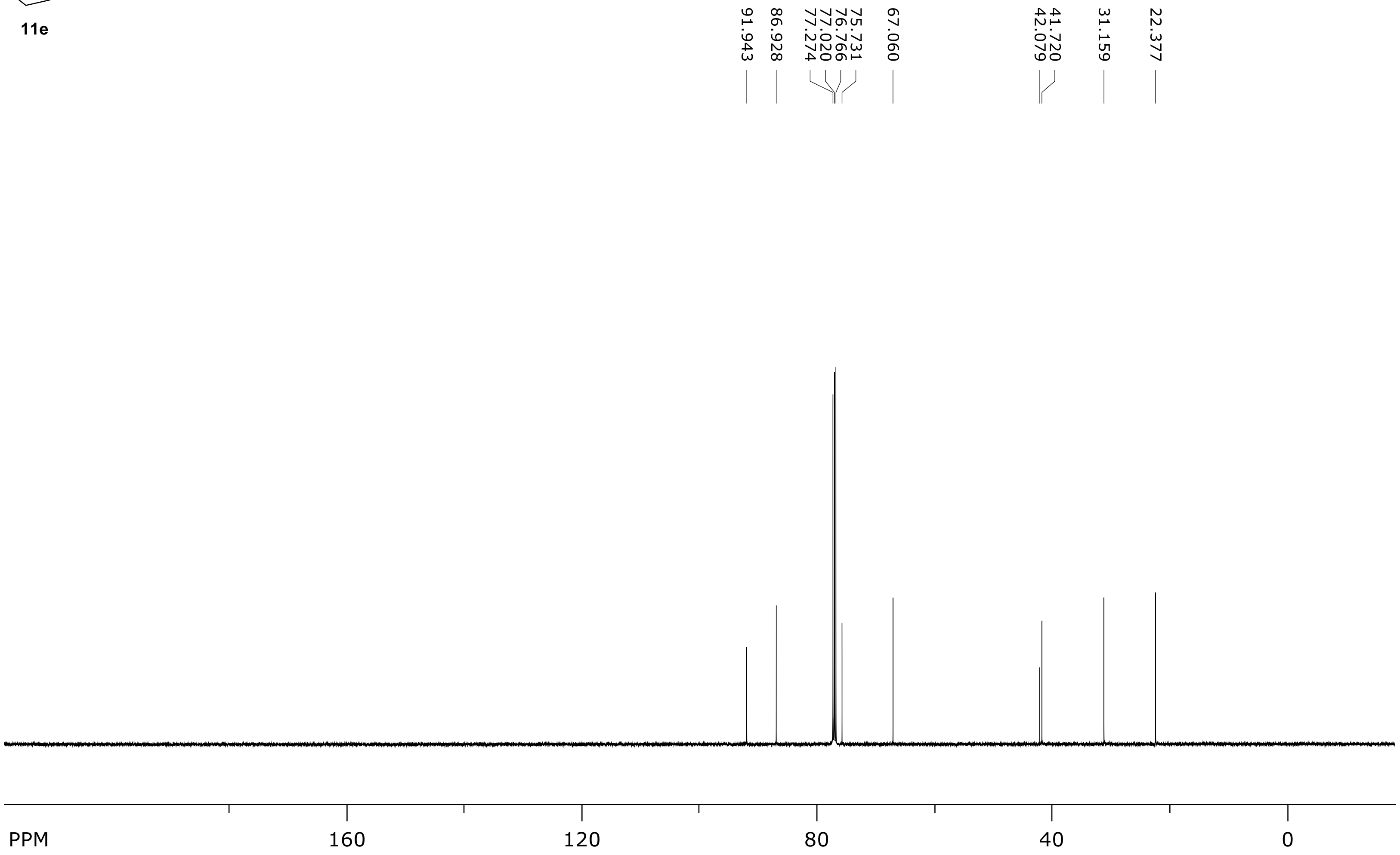

^{1}H NMR of (1*R*,2*R*,5*S*)-2-Chloro-7,7-bis(4-methoxybenzyl)-3,8-dioxabicyclo[3.2.1]octane (11f)

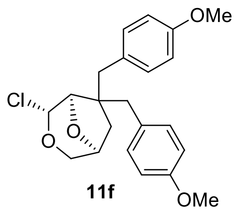

11f

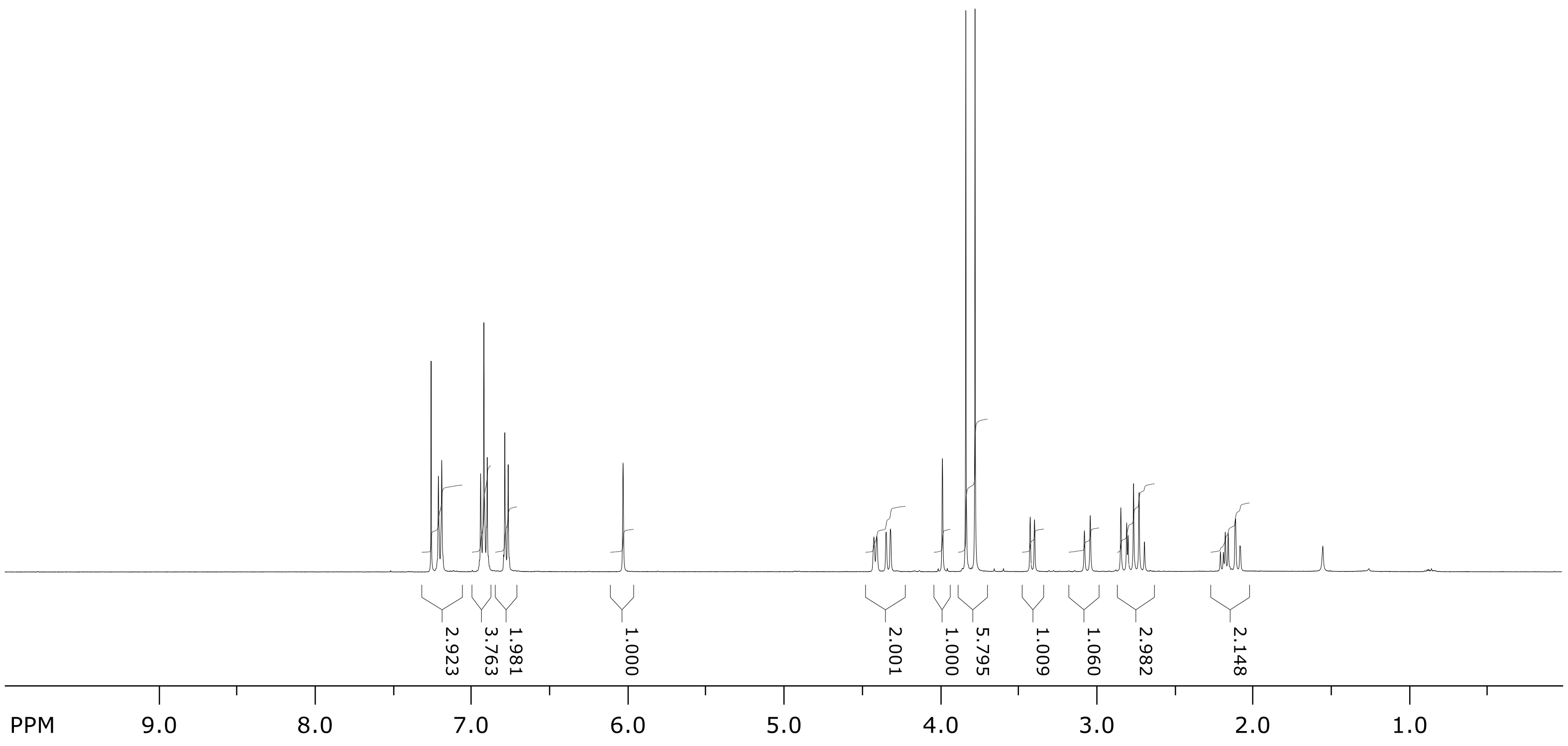

^{13}C{^{1}H} NMR of (1*R*,2*R*,5*S*)-2-Chloro-7,7-bis(4-methoxybenzyl)-3,8-dioxabicyclo[3.2.1]octane (11f).

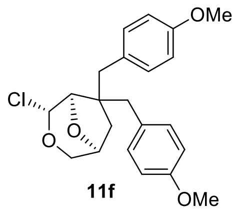

11f

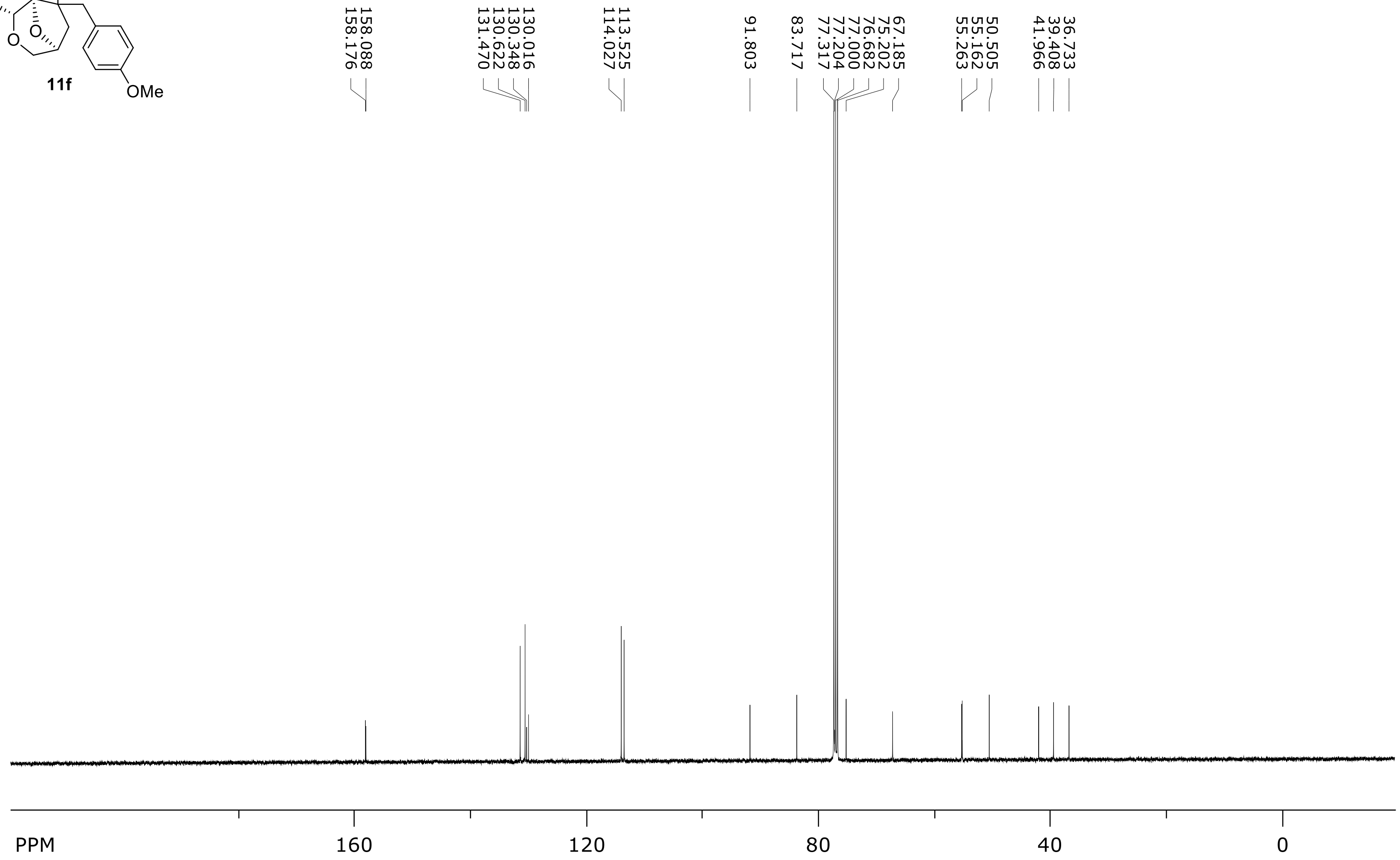

^{1}H NMR of (1*R*,2*S*,5*S*)-7,7-Dibenzyl-3,8-dioxabicyclo[3.2.1]octan-2-ol (12a)

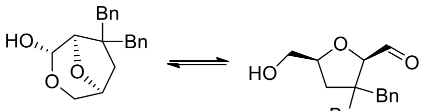

12a

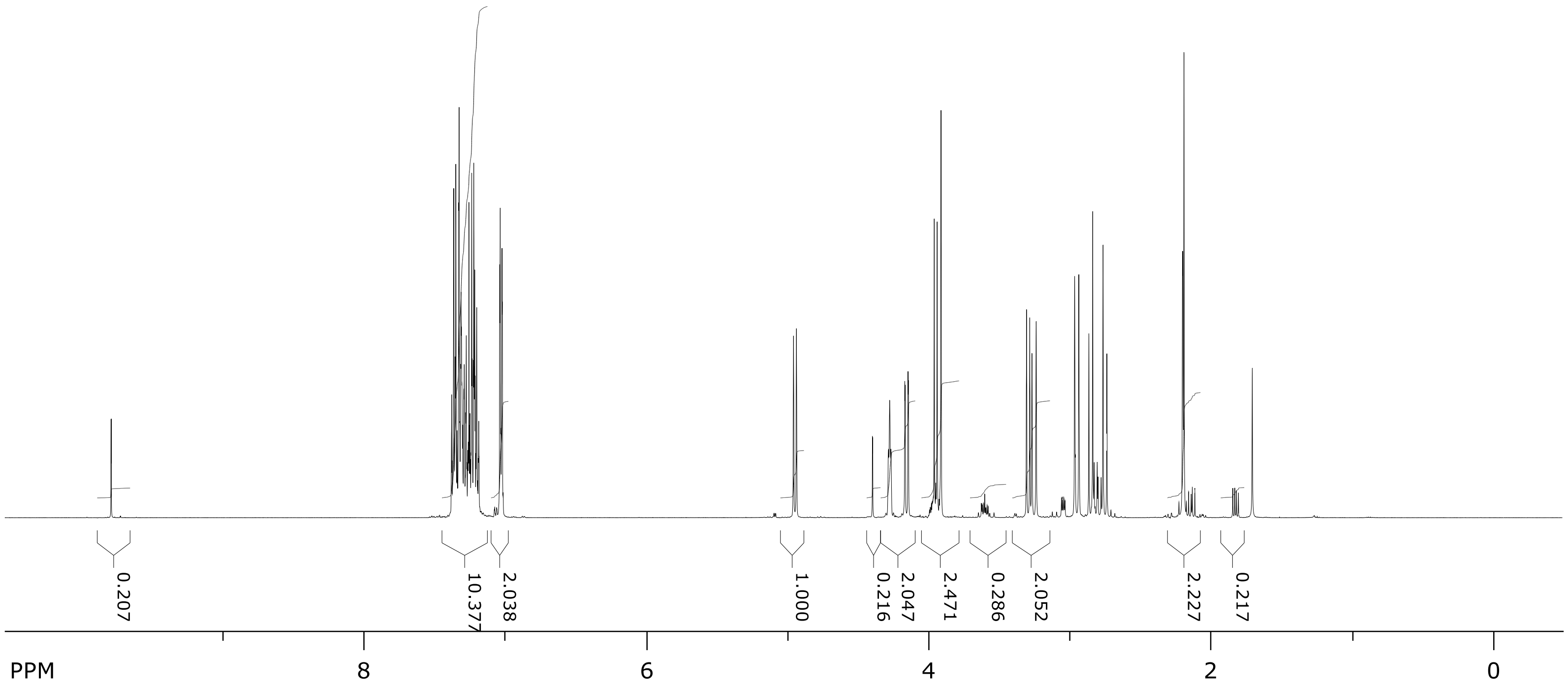

$^{13}C\{^{1}H\}$ NMR of (1*R*,2*S*,5*S*)-7,7-Dibenzyl-3,8-dioxabicyclo[3.2.1]octan-2-ol (12a)

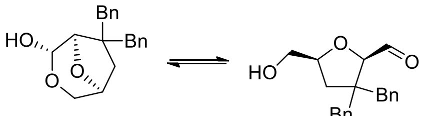

12a

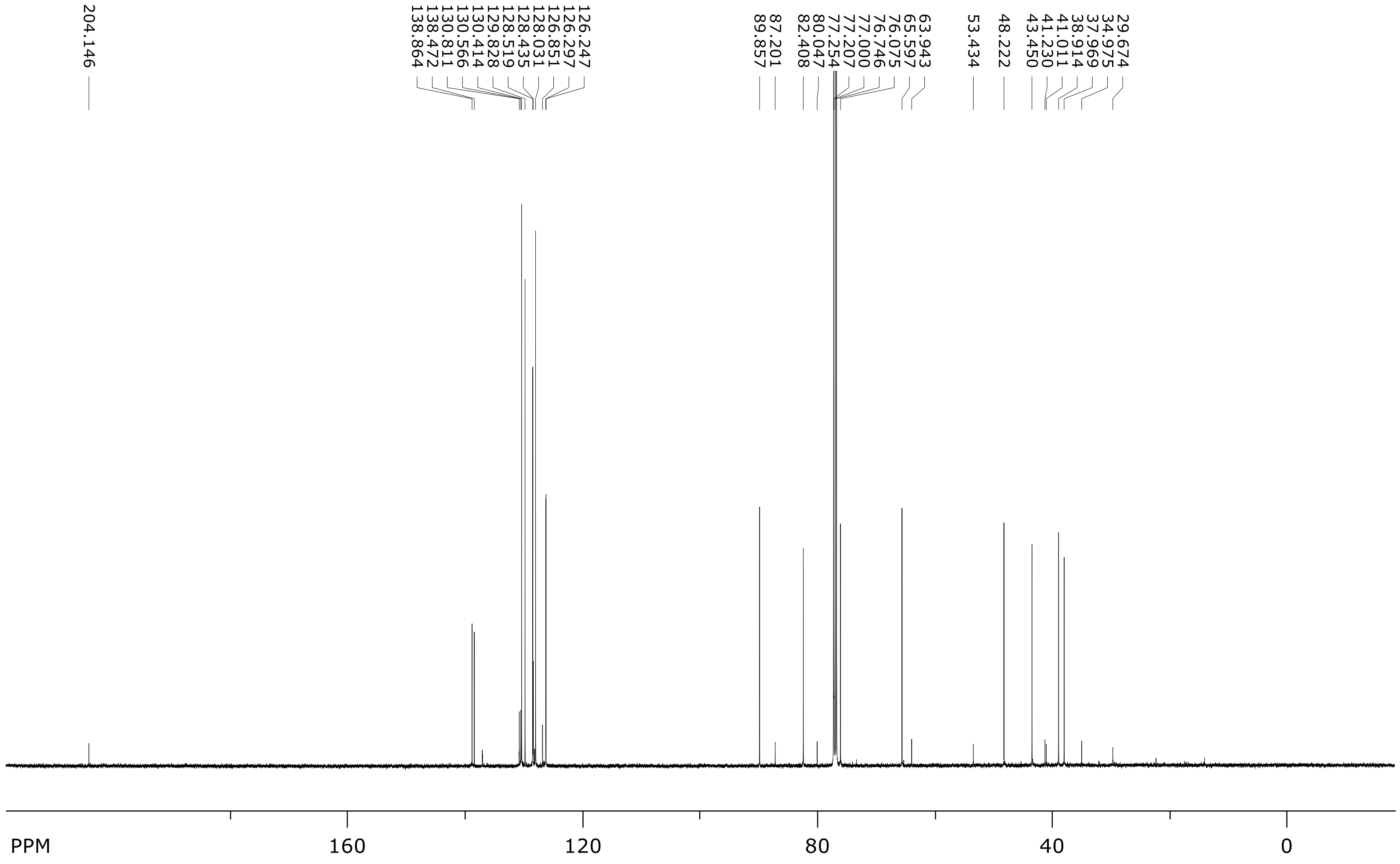

^{1}H NMR of (1*R*,2*S*,5*S*)-7,7-Bis(2-methylbenzyl)-3,8-dioxabicyclo[3.2.1]octan-2-ol (12c)

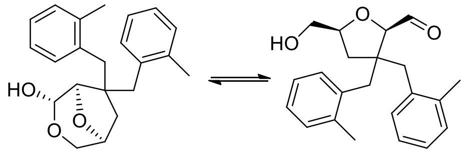

12c

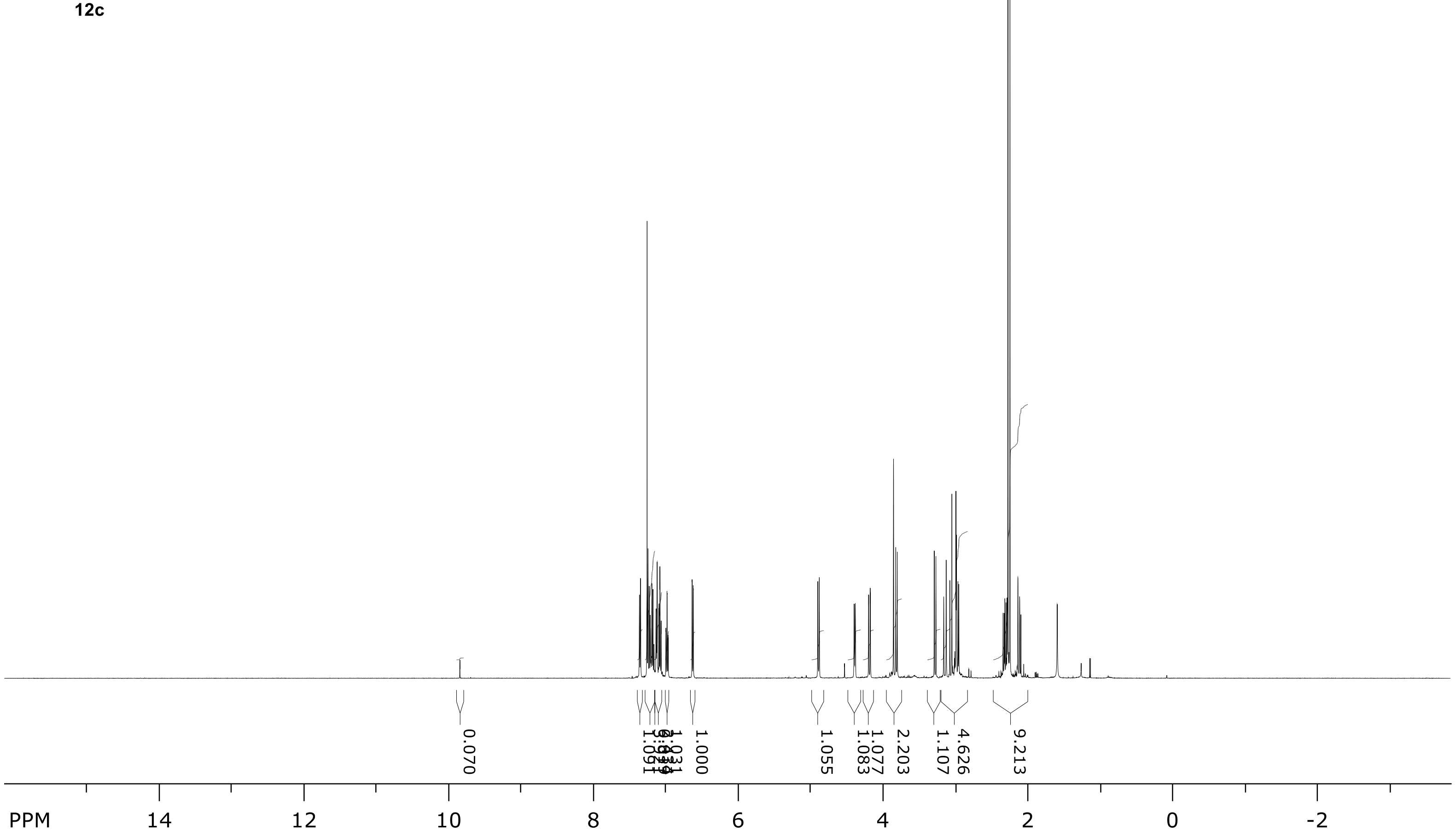

$^{13}C\{^{1}H\}$ NMR of (1*R*,2*S*,5*S*)-7,7-Bis(2-methylbenzyl)-3,8-dioxabicyclo[3.2.1]octan-2-ol (12c).

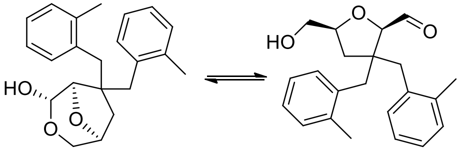

12c

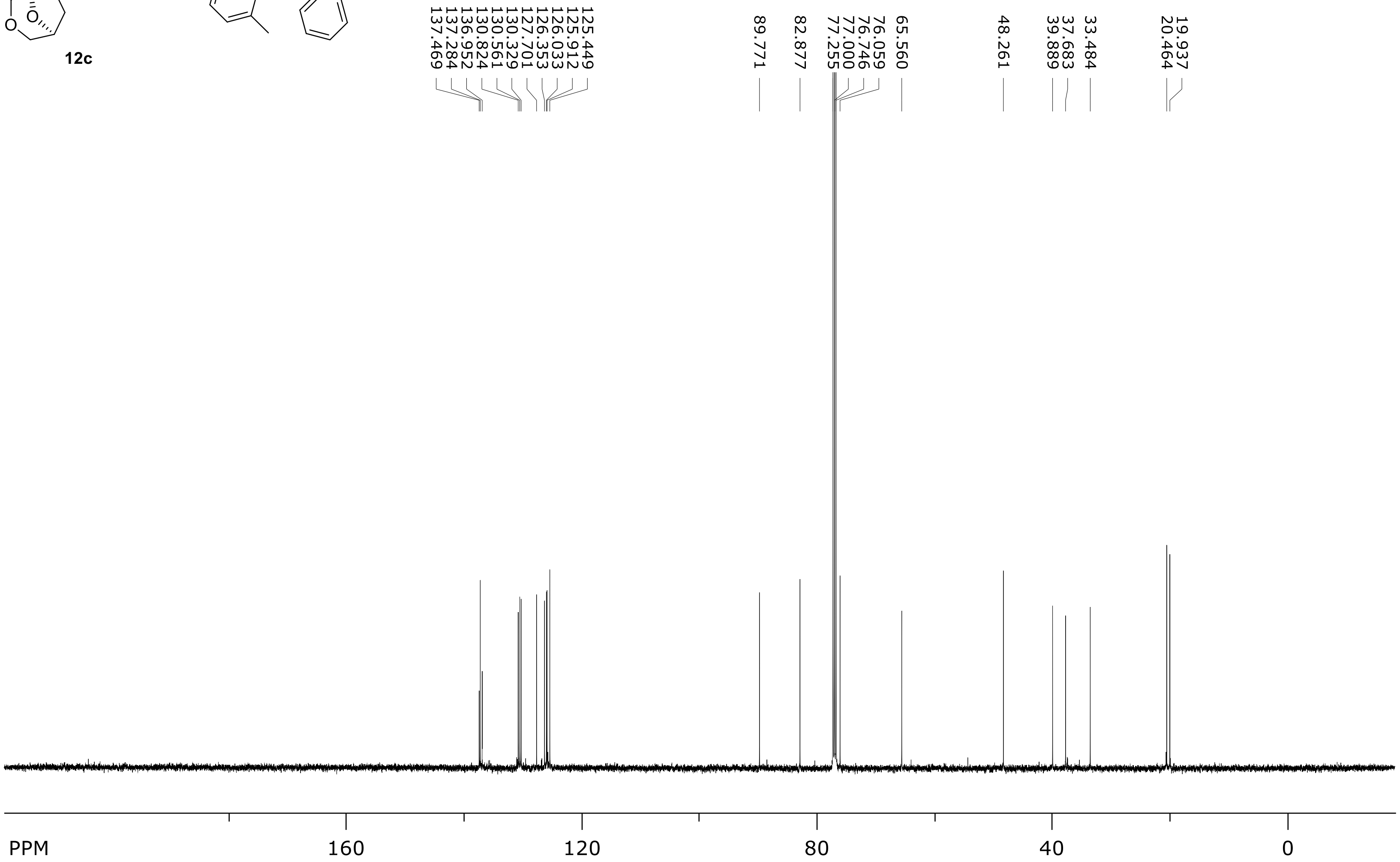

^{1}H NMR of (1*S*,4*S*,5*R*)-1',3'-Dihydro-3,8-dioxaspiro[bicyclo[3.2.1]octane-6,2'-inden]-4-ol (12d)

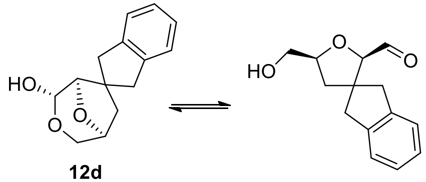

12d

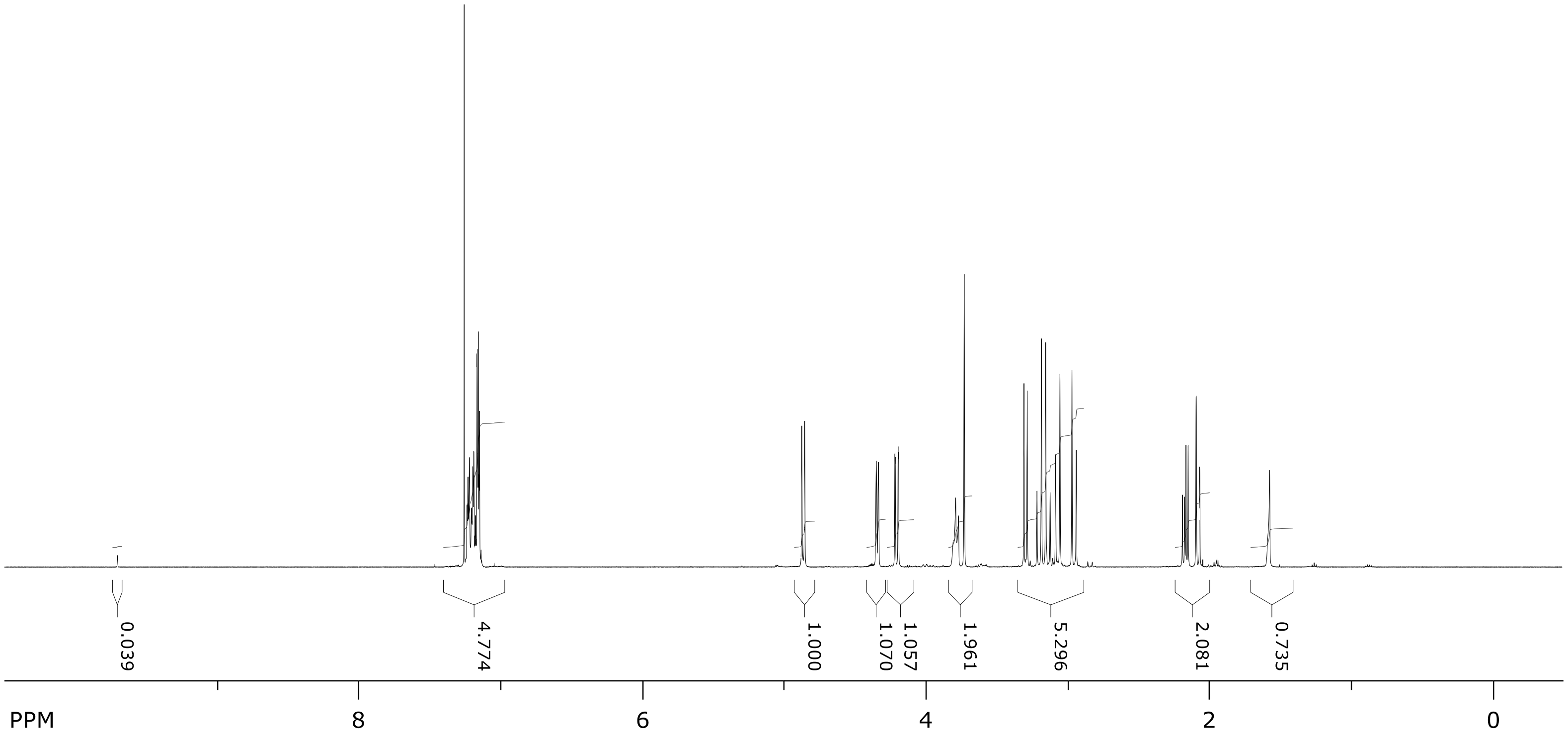

$^{13}C\{^{1}H\}$ NMR of (1*S*,4*S*,5*R*)-1',3'-Dihydro-3,8-dioxaspiro[bicyclo[3.2.1]octane-6,2'-inden]-4-ol (12d).

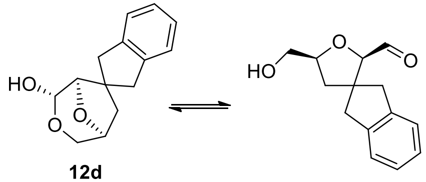

12d

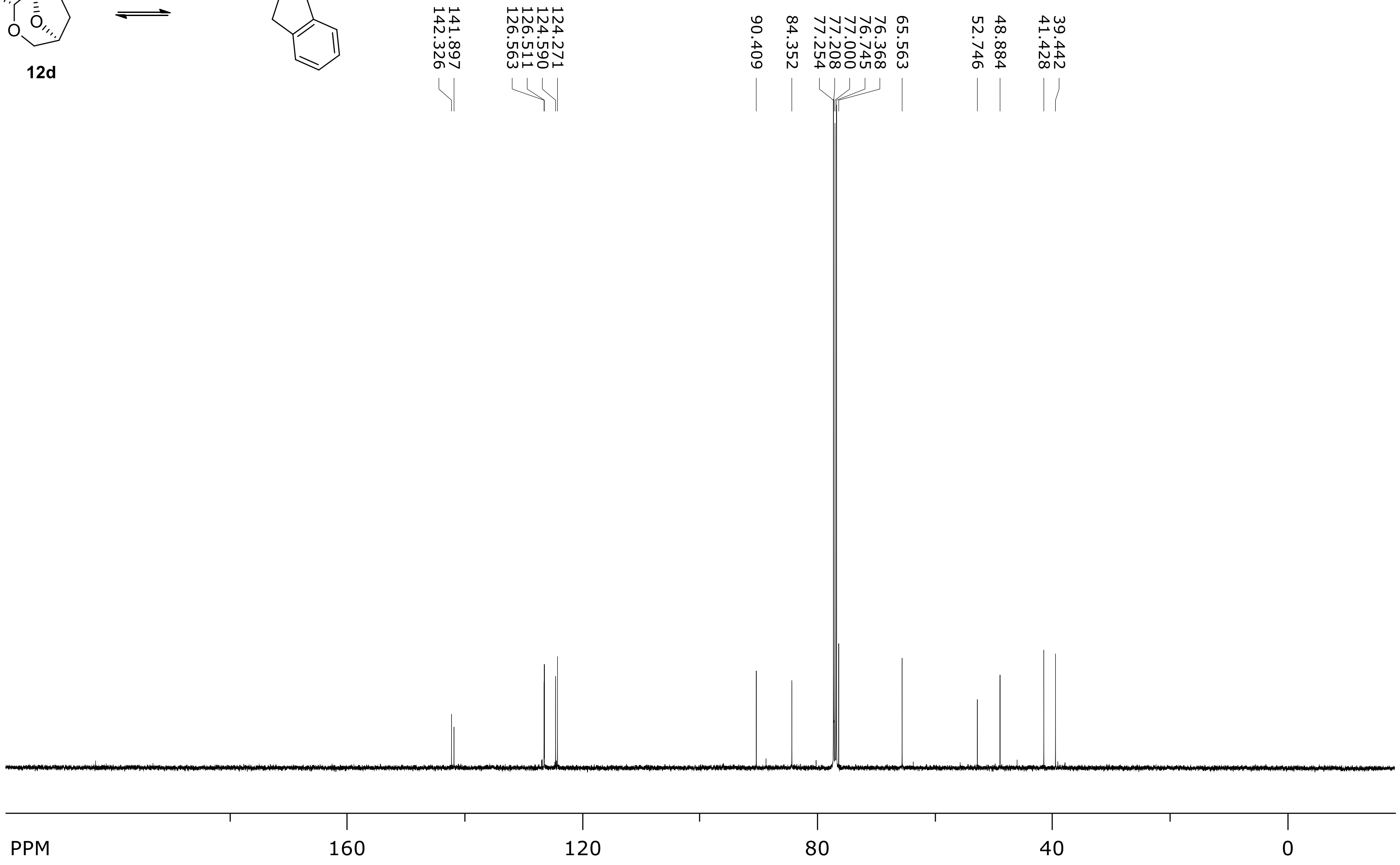

^{1}H NMR of (1*R*,2*S*,5*S*)-7,7-Dimethyl-3,8-dioxabicyclo[3.2.1]octan-2-ol (12e)

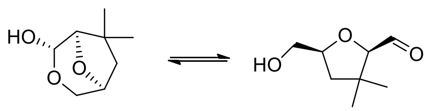

12e

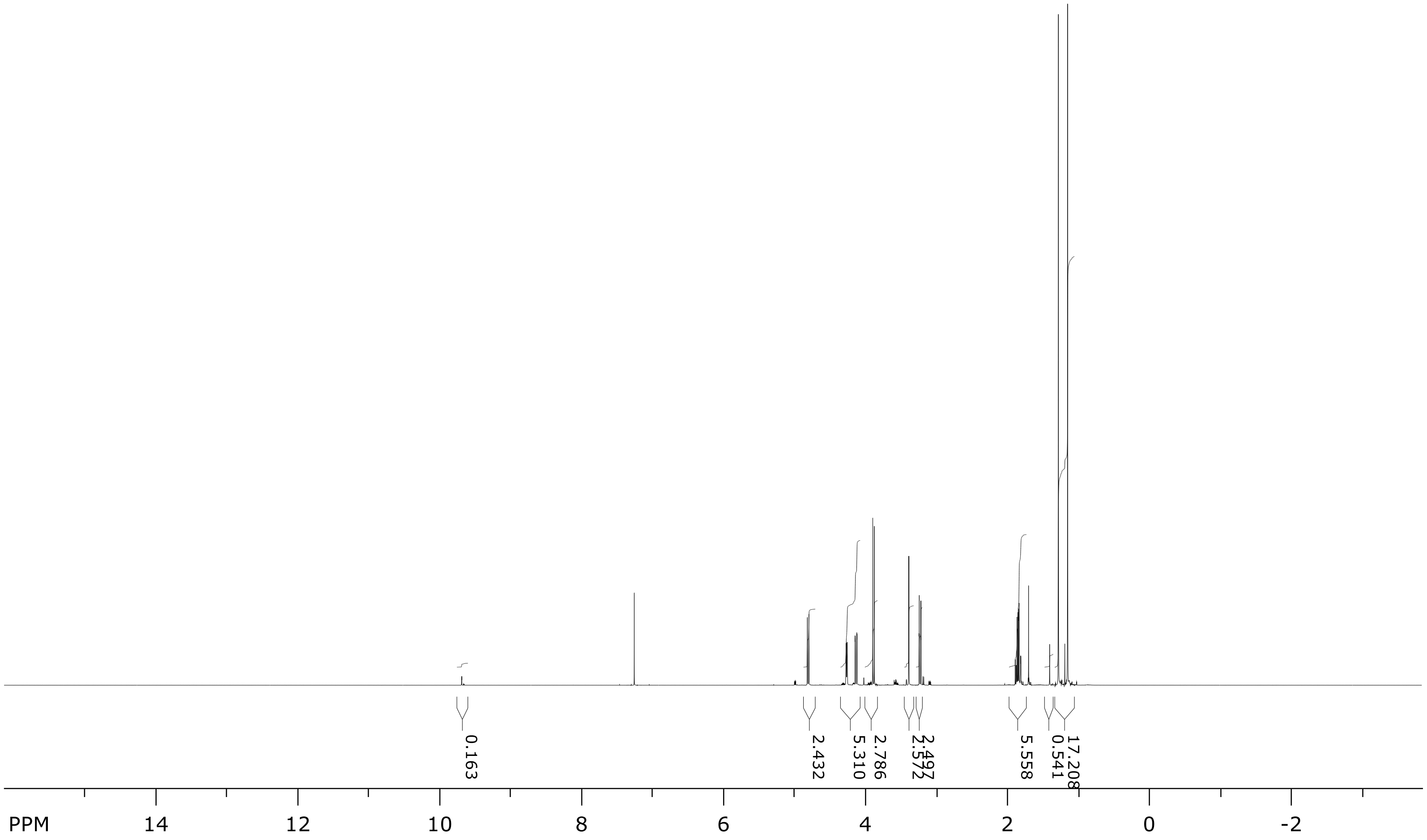

$^{13}C\{^{1}H\}$ NMR of (1*R*,2*S*,5*S*)-7,7-Dimethyl-3,8-dioxabicyclo[3.2.1]octan-2-ol (12e)

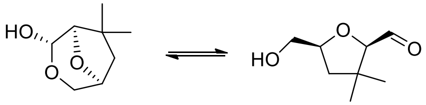

12e

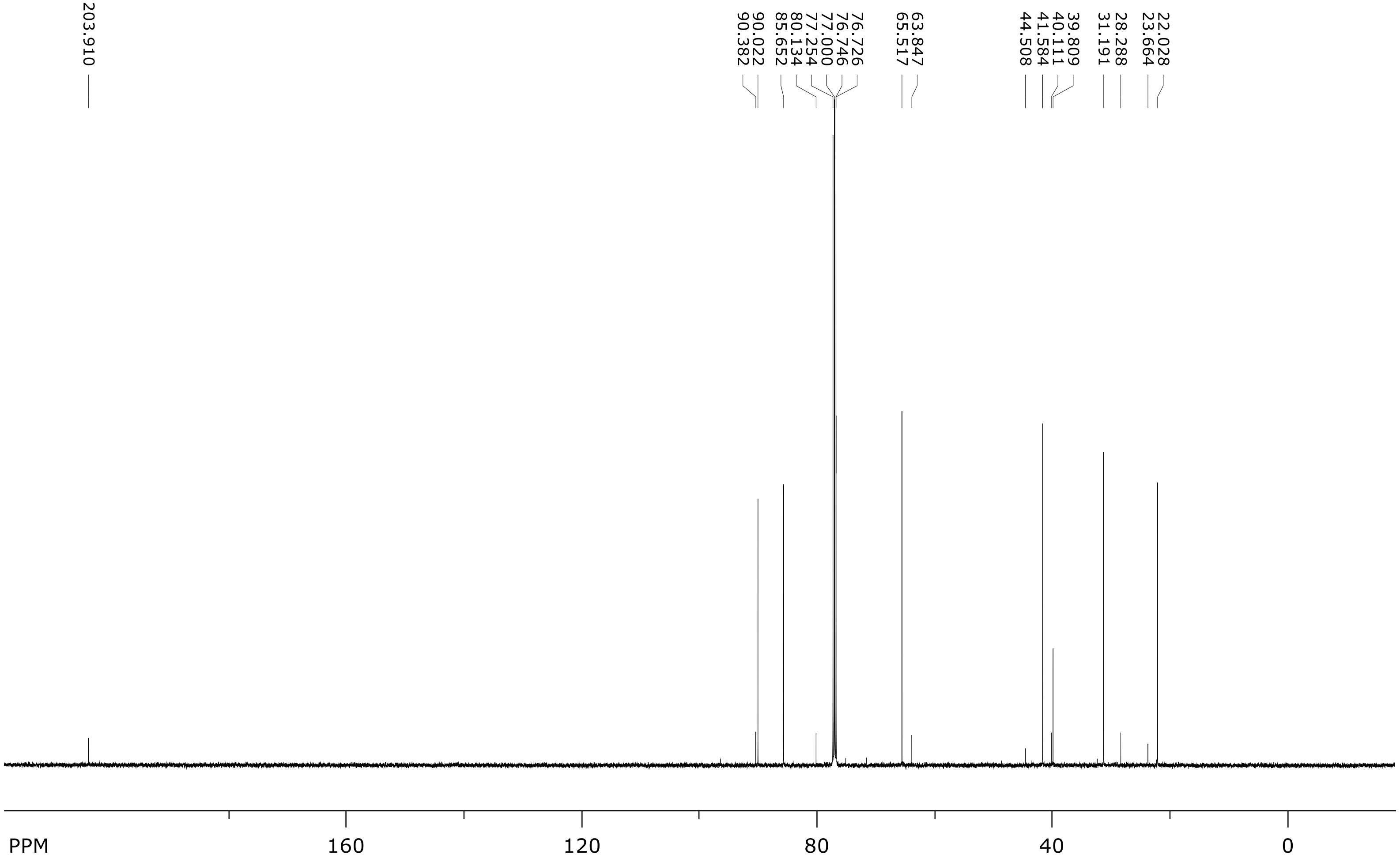

^{1}H NMR of (1*R*,2*S*,5*S*)-7,7-Bis(4-methoxybenzyl)-3,8-dioxabicyclo[3.2.1]octan-2-ol (12f)

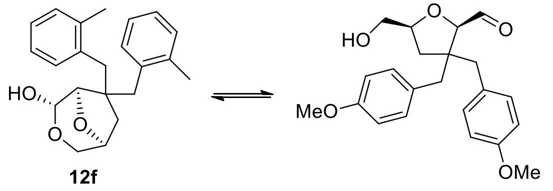

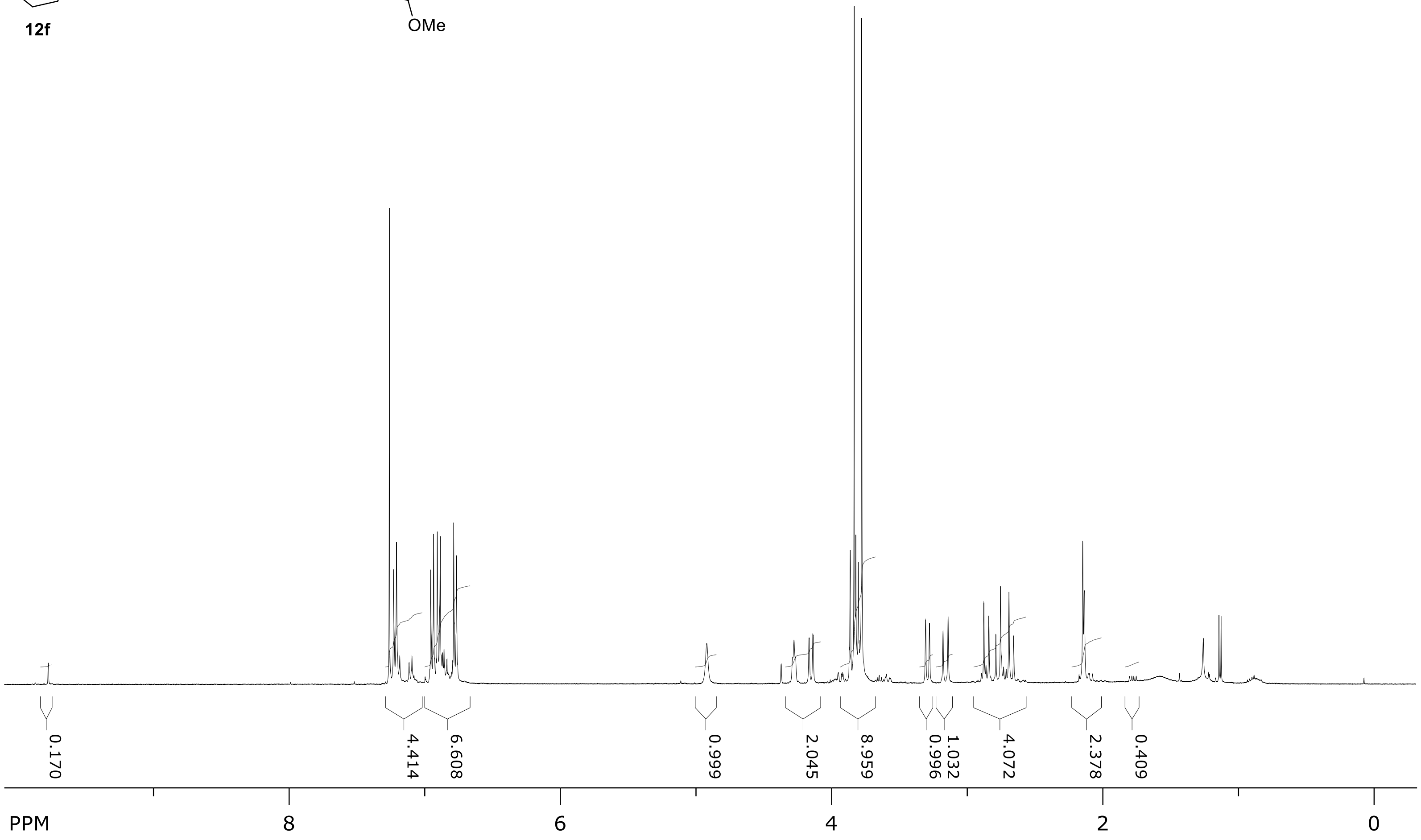

$^{13}C\{^{1}H\}$ NMR of (1*R*,2*S*,5*S*)-7,7-Bis(4-methoxybenzyl)-3,8-dioxabicyclo[3.2.1]octan-2-ol (12f).

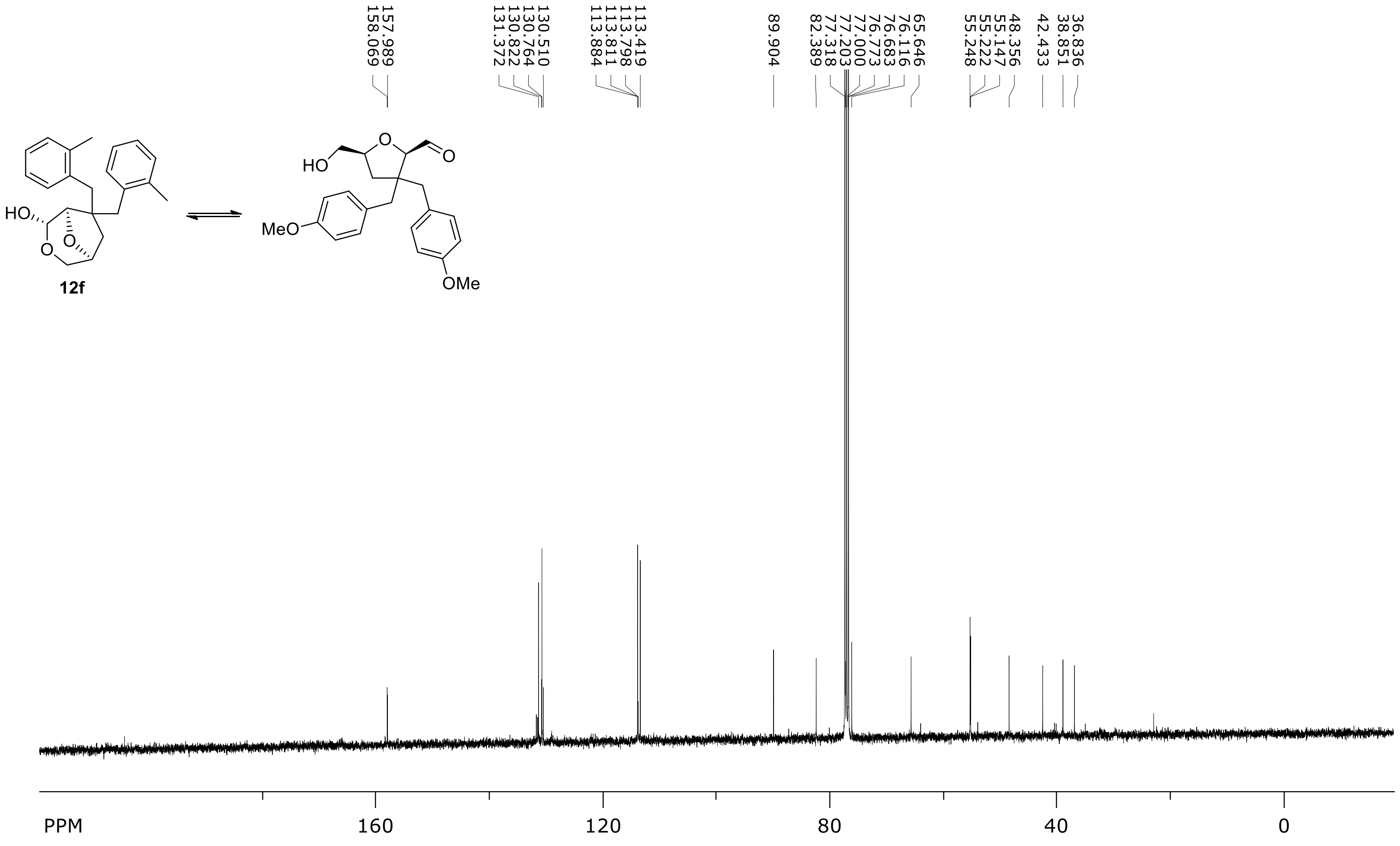

^{1}H NMR of Di((1*S*,4*S*,5*R*)-6,8-dioxabicyclo[3.2.1]octan-4-yl)sulfite (13b)

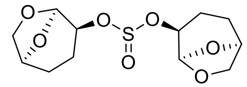

13b

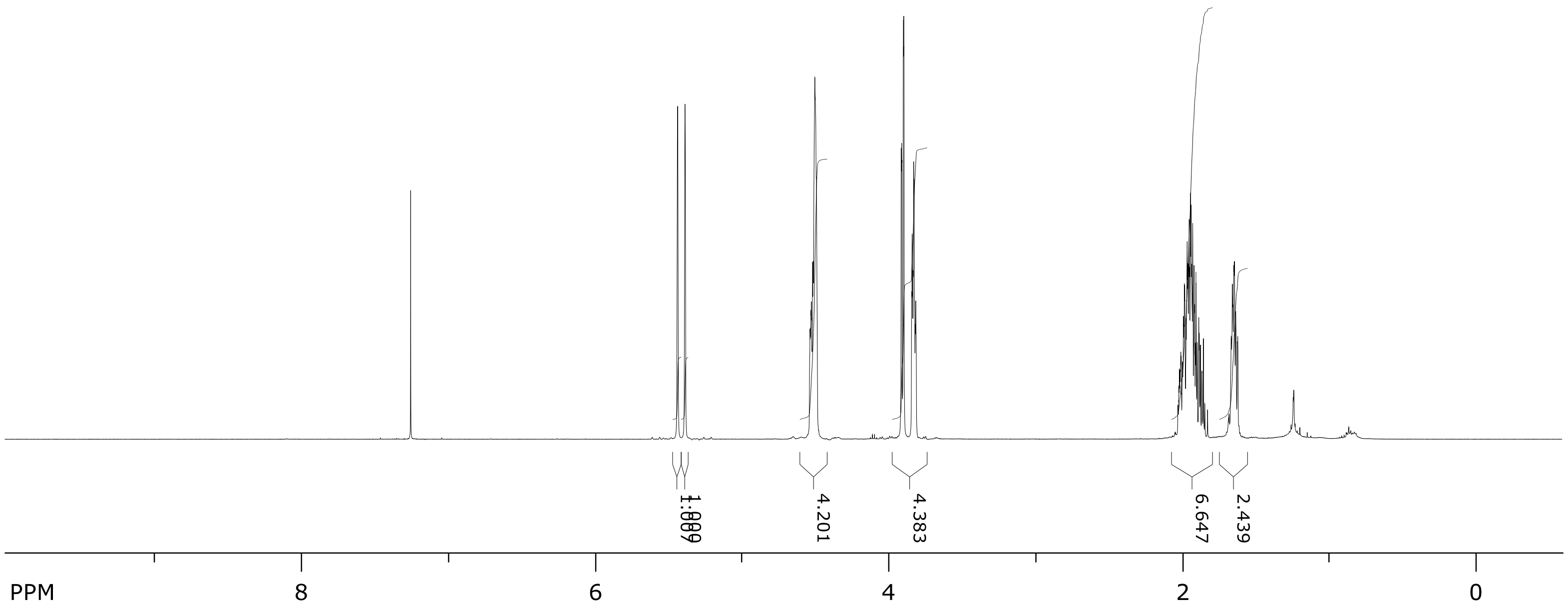

^{13}C{^{1}H} NMR of Di((1*S*,4*S*,5*R*)-6,8-dioxabicyclo[3.2.1]octan-4-yl)sulfite (13b)

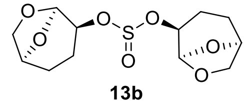

13b

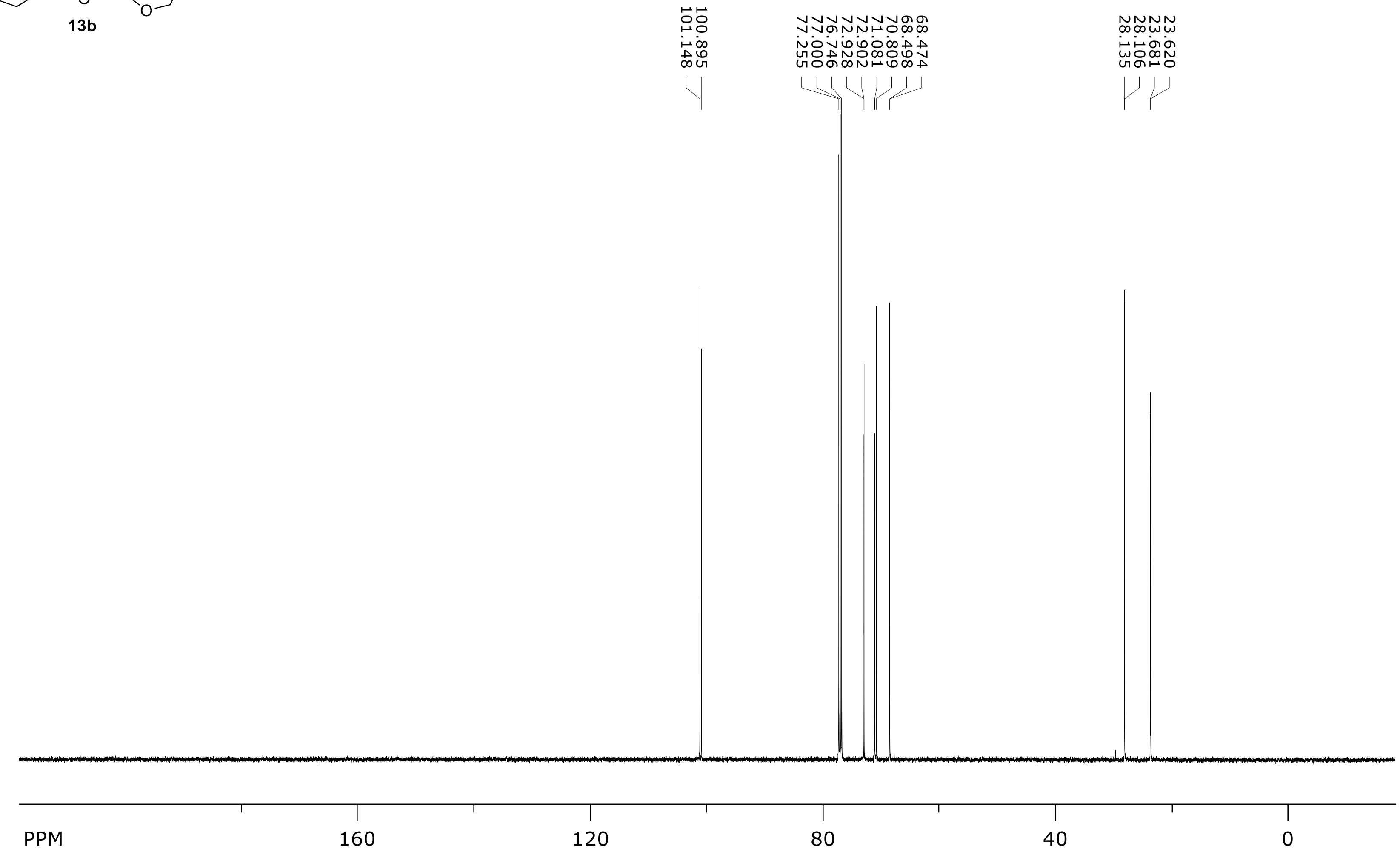

^{1}H NMR of Bis((1*S*,4*S*,5*R*)-1',3'-dihydro-6,8-dioxaspiro[bicyclo[3.2.1]octane-3,2'-inden]-4-yl)sulphite (13d)

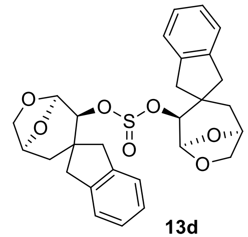

13d

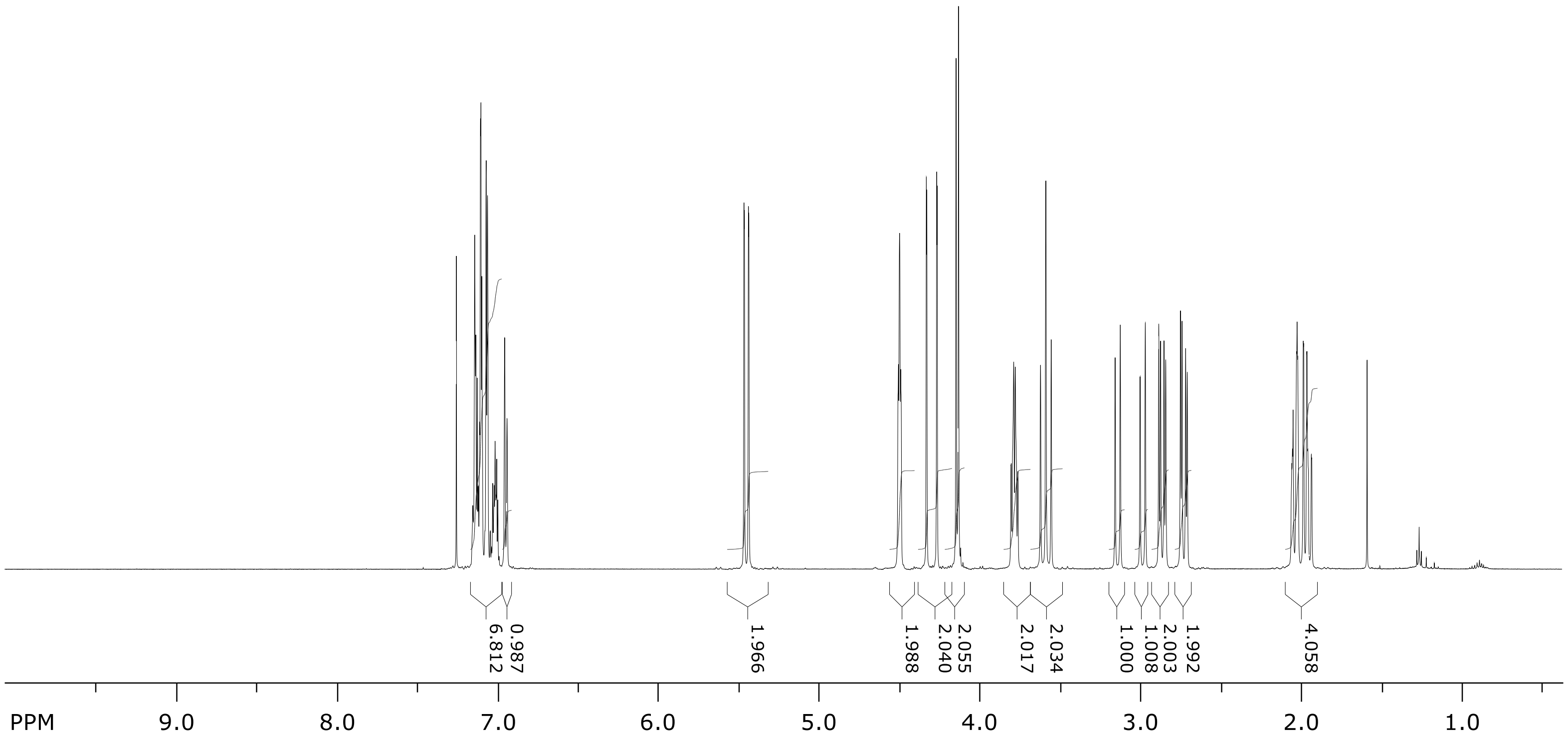

$^{13}C\{^1H\}$ NMR of Bis((1*S*,4*S*,5*R*)-1',3'-dihydro-6,8-dioxaspiro[bicyclo[3.2.1]octane-3,2'-inden]-4-yl)sulphite (13d)

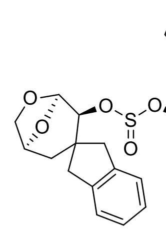

13d

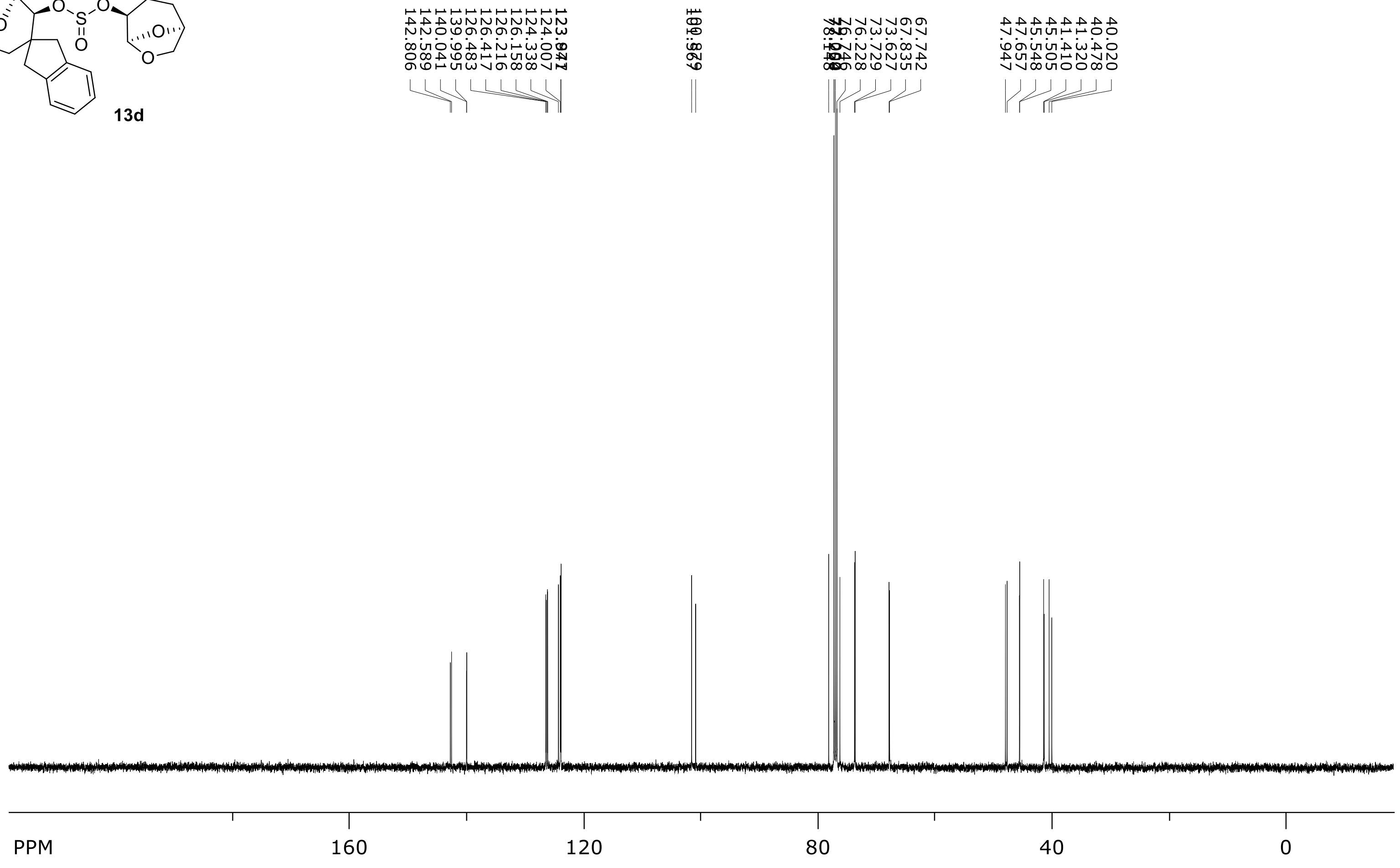

^{1}H NMR of Di((1*S*,4*S*,5*R*)-3,3-dimethyl-6,8-dioxabicyclo[3.2.1]octan-4-yl) sulfite (13e)

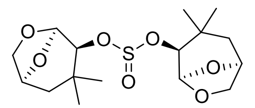

13e

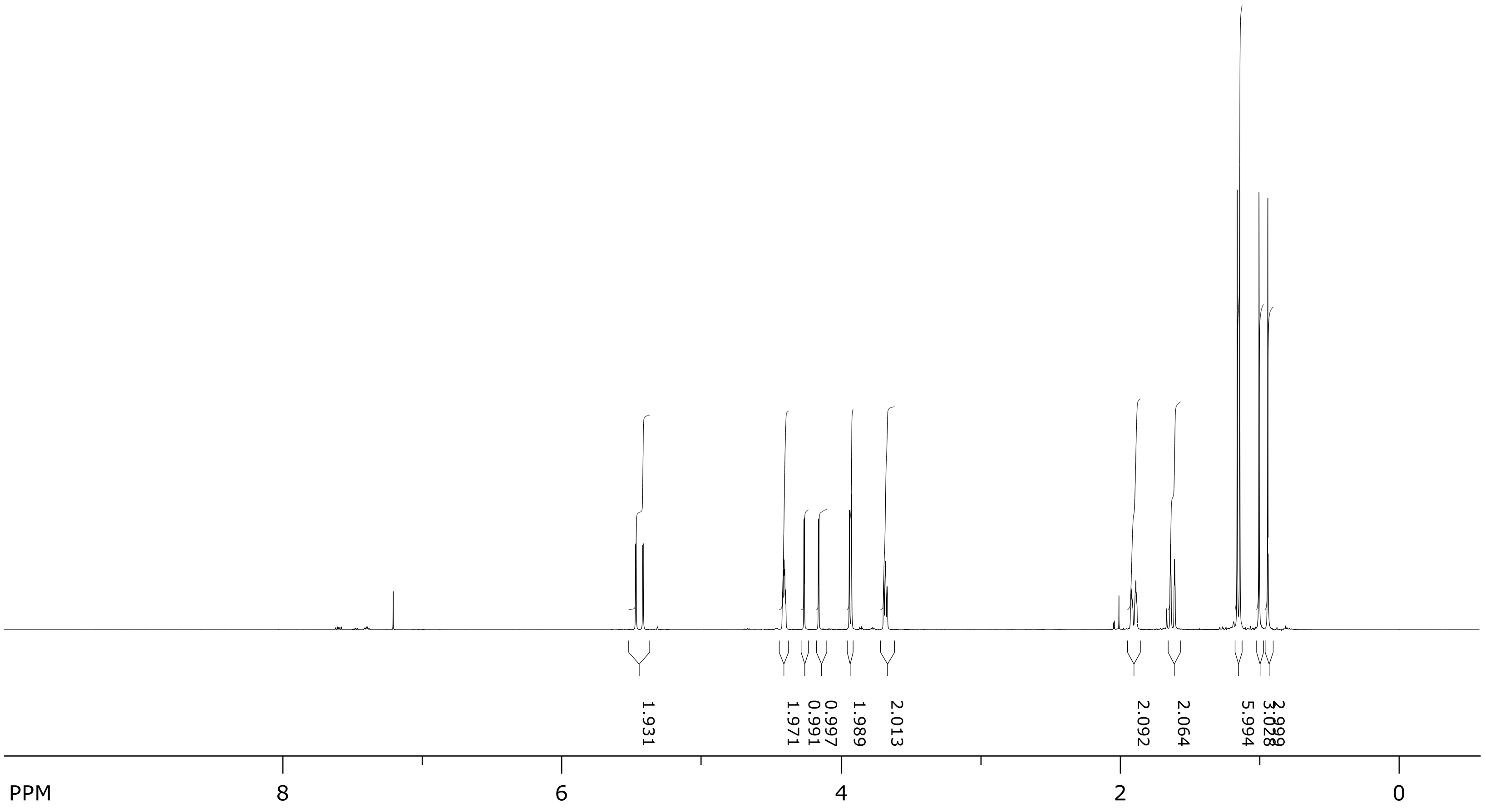

$^{13}C\{^{1}H\}$ NMR of Di((1*S*,4*S*,5*R*)-3,3-dimethyl-6,8-dioxabicyclo[3.2.1]octan-4-yl) sulfite (13e)

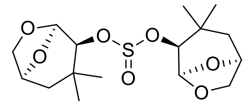

13e

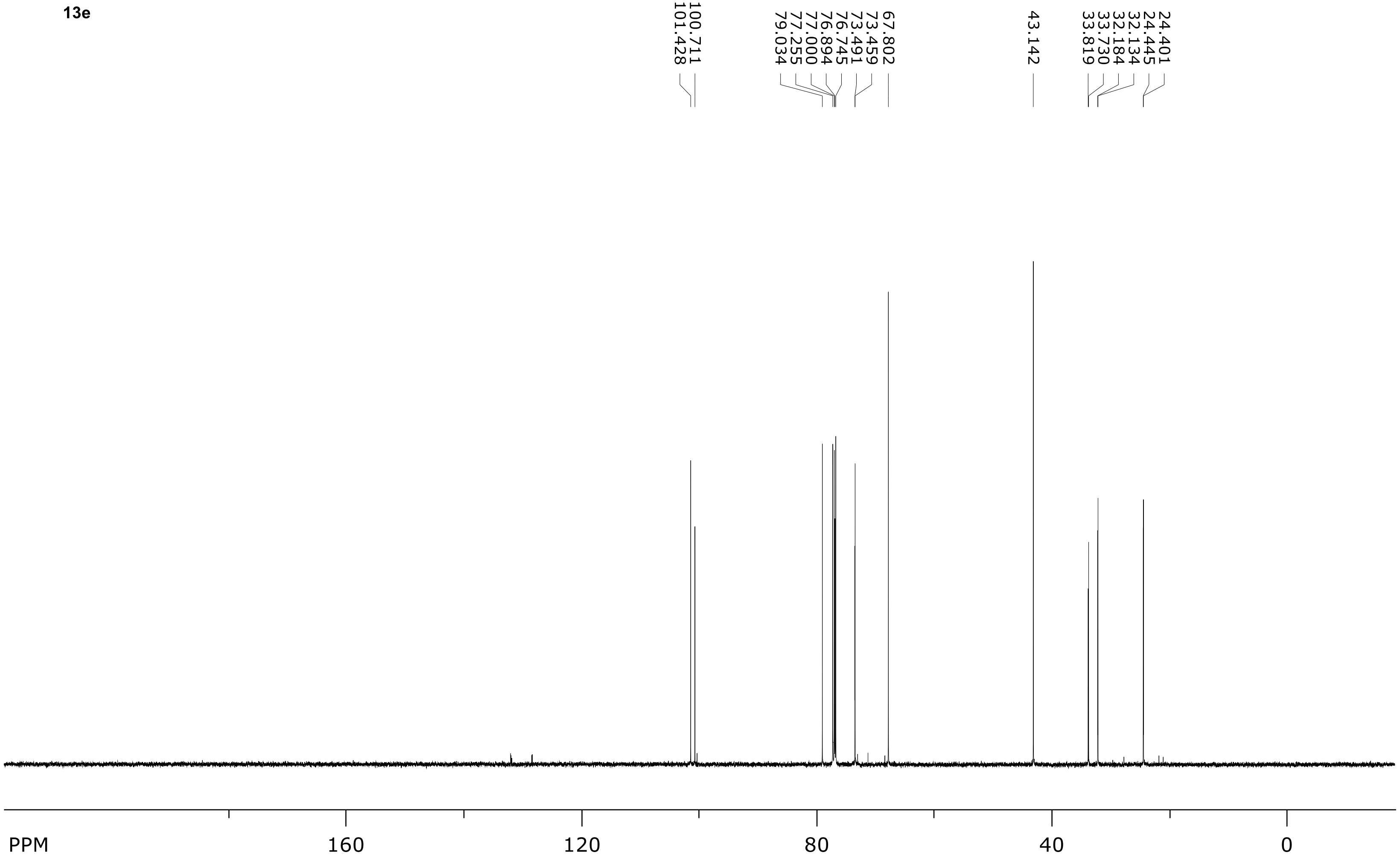

^{1}H NMR of (1*R*,4*S*,5*R*)-4-Chloro-6,8-dioxabicyclo[3.2.1]oct-2-ene (16)

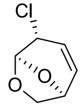

16

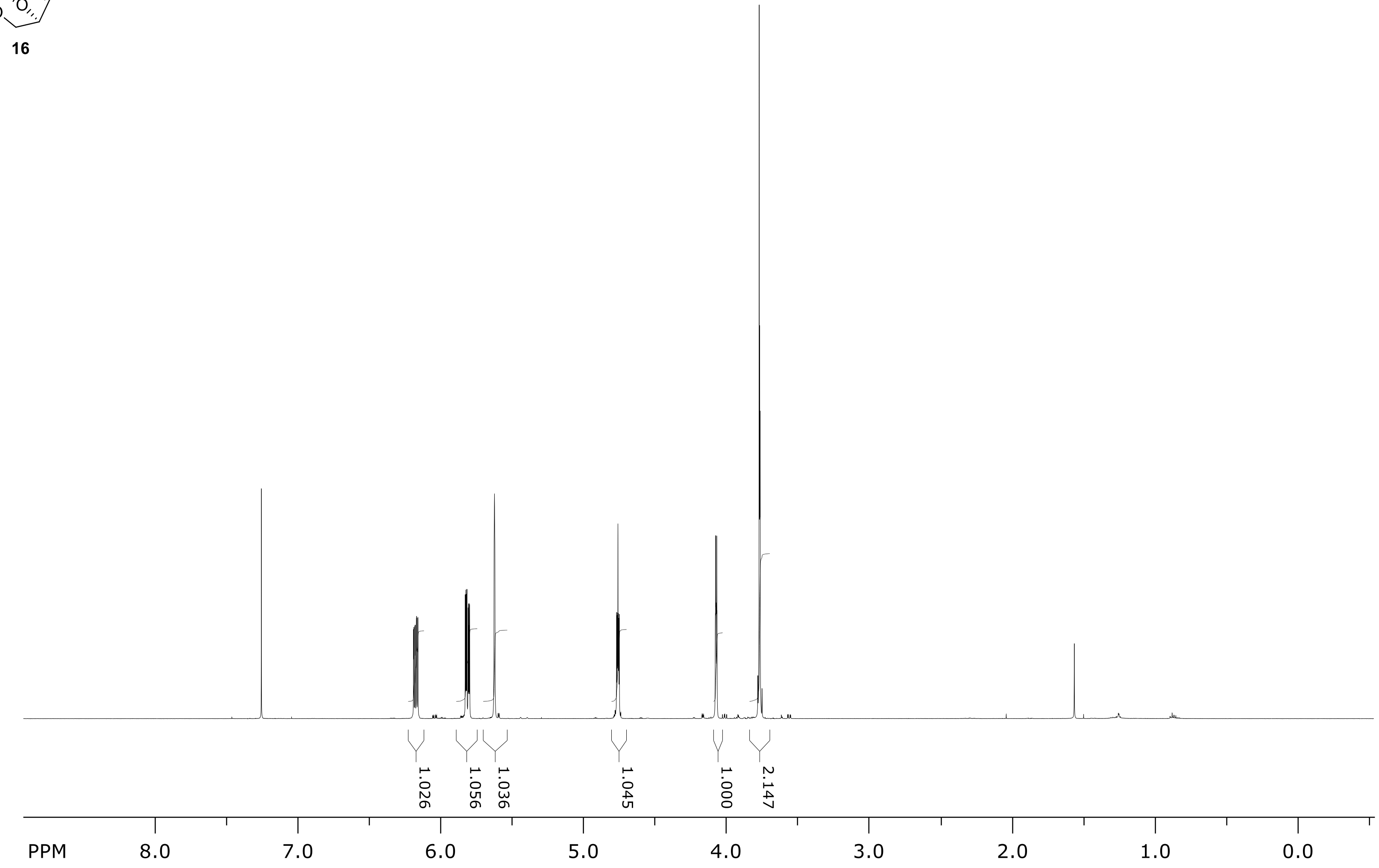

$^{13}C\{^1H\}$ NMR of (1*R*,4*S*,5*R*)-4-Chloro-6,8-dioxabicyclo[3.2.1]oct-2-ene (16)

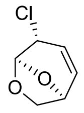

16

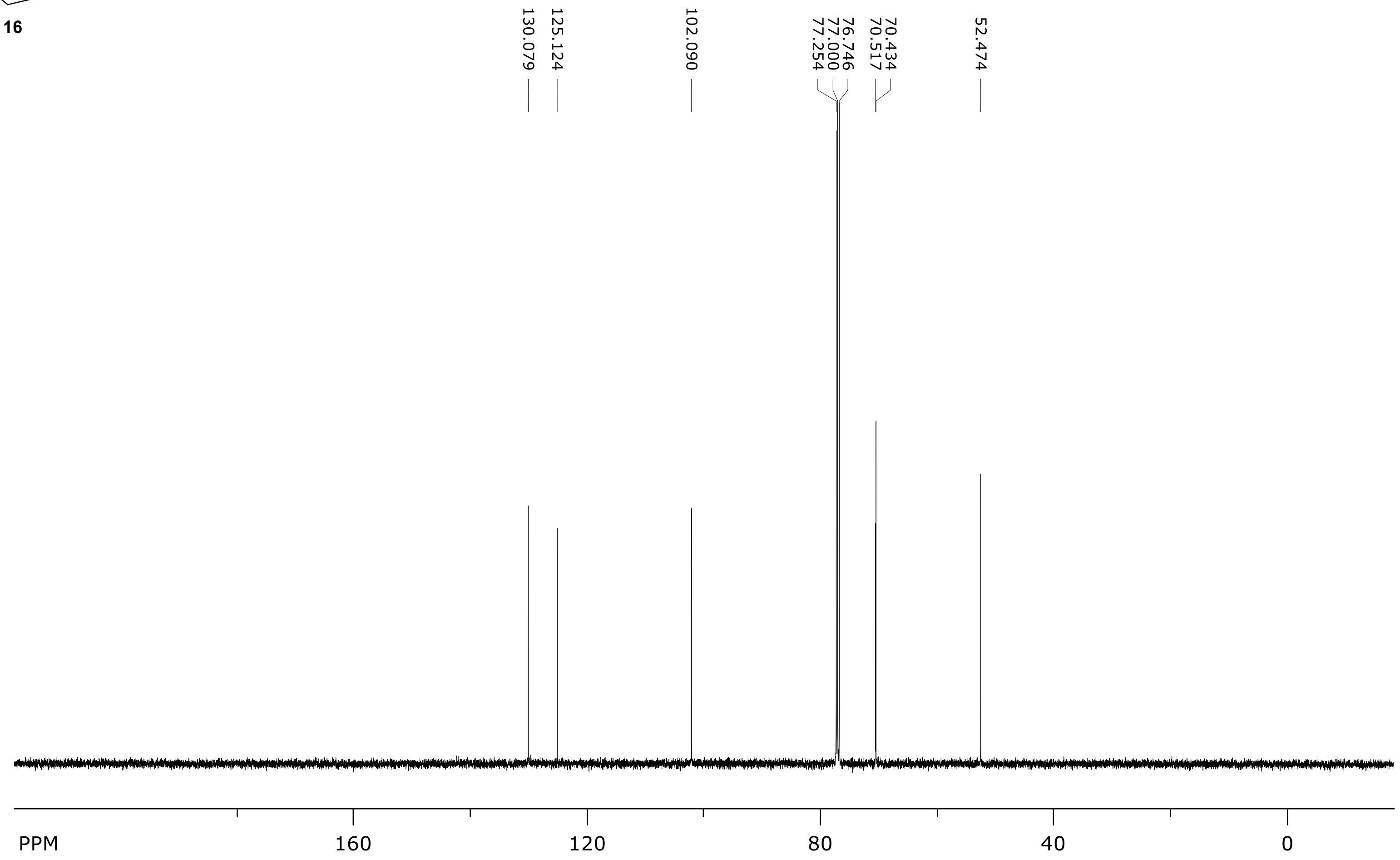

^{1}H NMR of (1*R*,2*R*,5*R*)-2-Chloro-6,8-dioxabicyclo[3.2.1]oct-3-ene (17a)

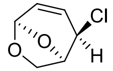

17a

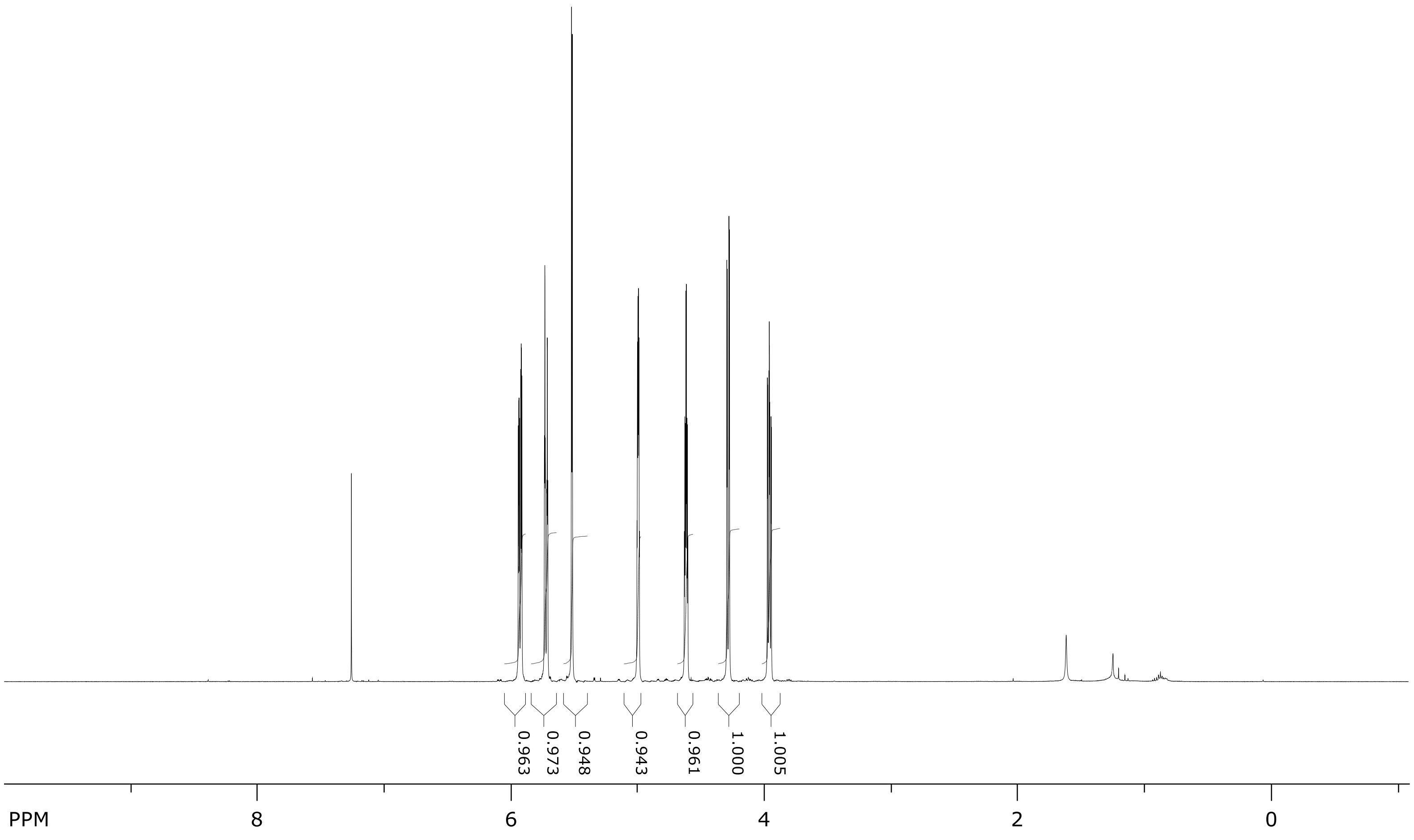

$^{13}C\{^{1}H\}$ NMR of (1*R*,2*R*,5*R*)-2-Chloro-6,8-dioxabicyclo[3.2.1]oct-3-ene (17a)

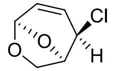

17a

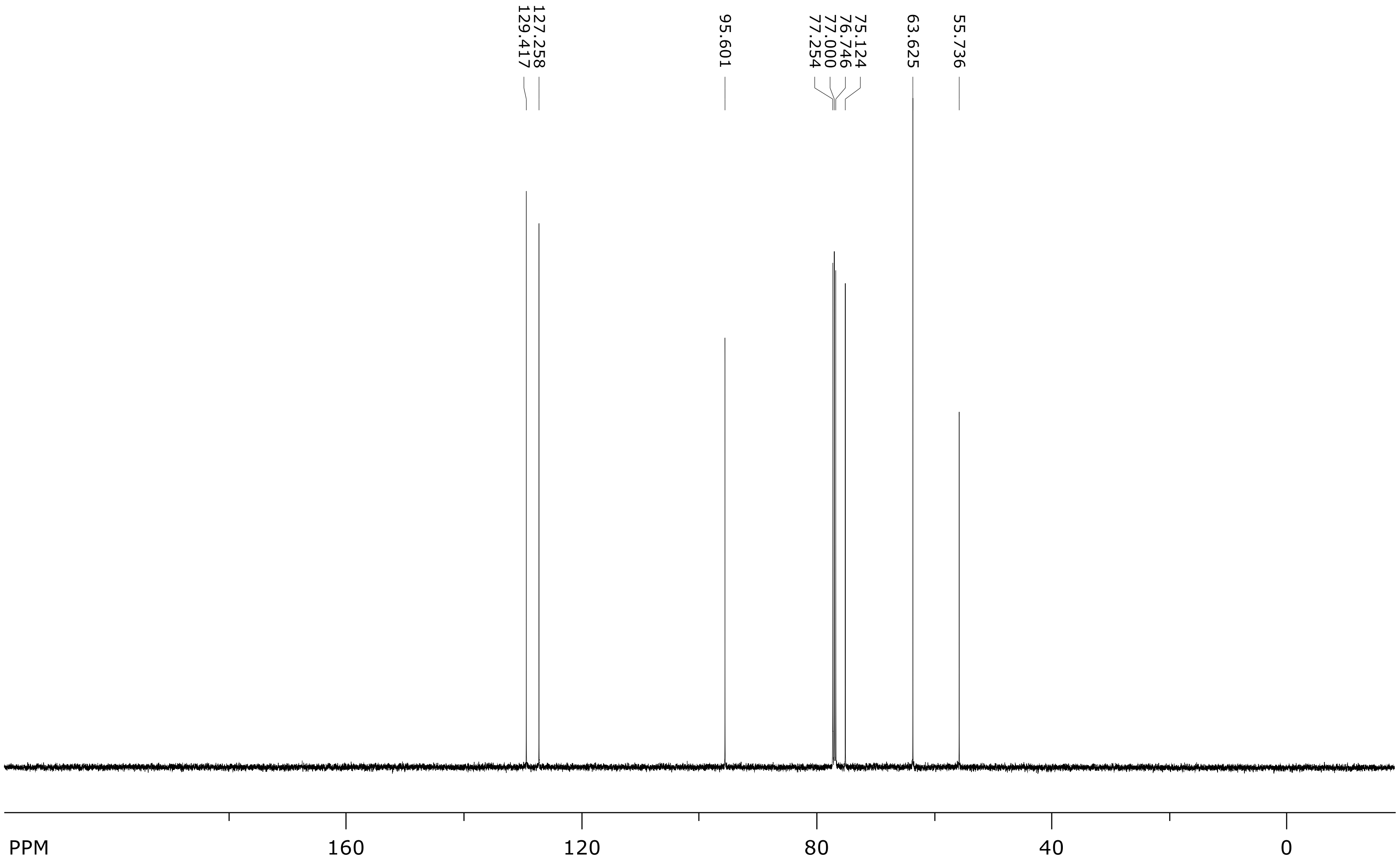

^{1}H NMR of (1*R*,2*S*,5*R*)-2-Chloro-6,8-dioxabicyclo[3.2.1]oct-3-ene (17b)

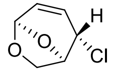

17b

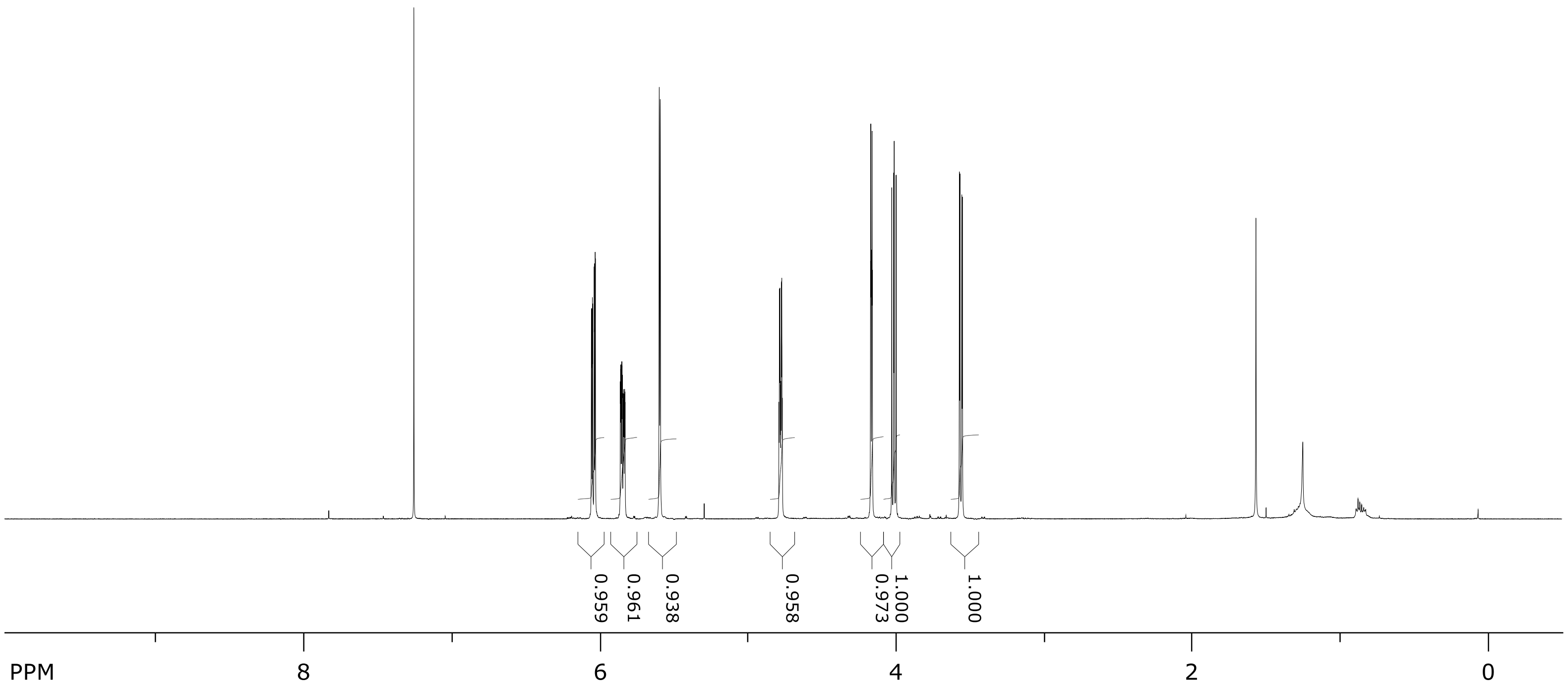

$^{13}C\{^1H\}$ NMR of (1*R*,2*S*,5*R*)-2-Chloro-6,8-dioxabicyclo[3.2.1]oct-3-ene (17b)

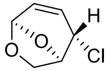

17b

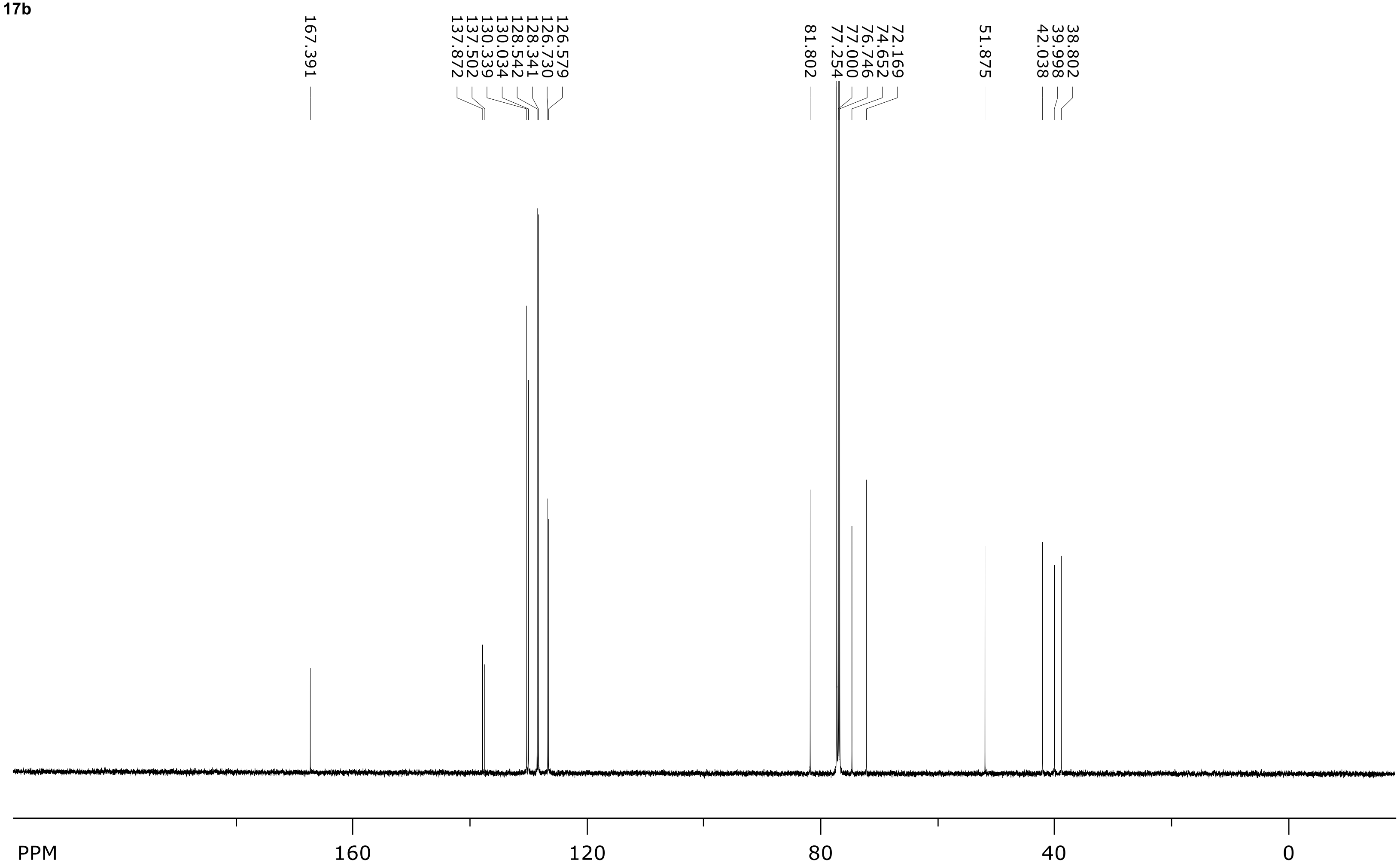

^{1}H NMR of (1*S*,5*R*)-3-((E)-Benzylidene)-4-chloro-6,8-dioxabicyclo[3.2.1]octane (19)

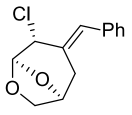

19

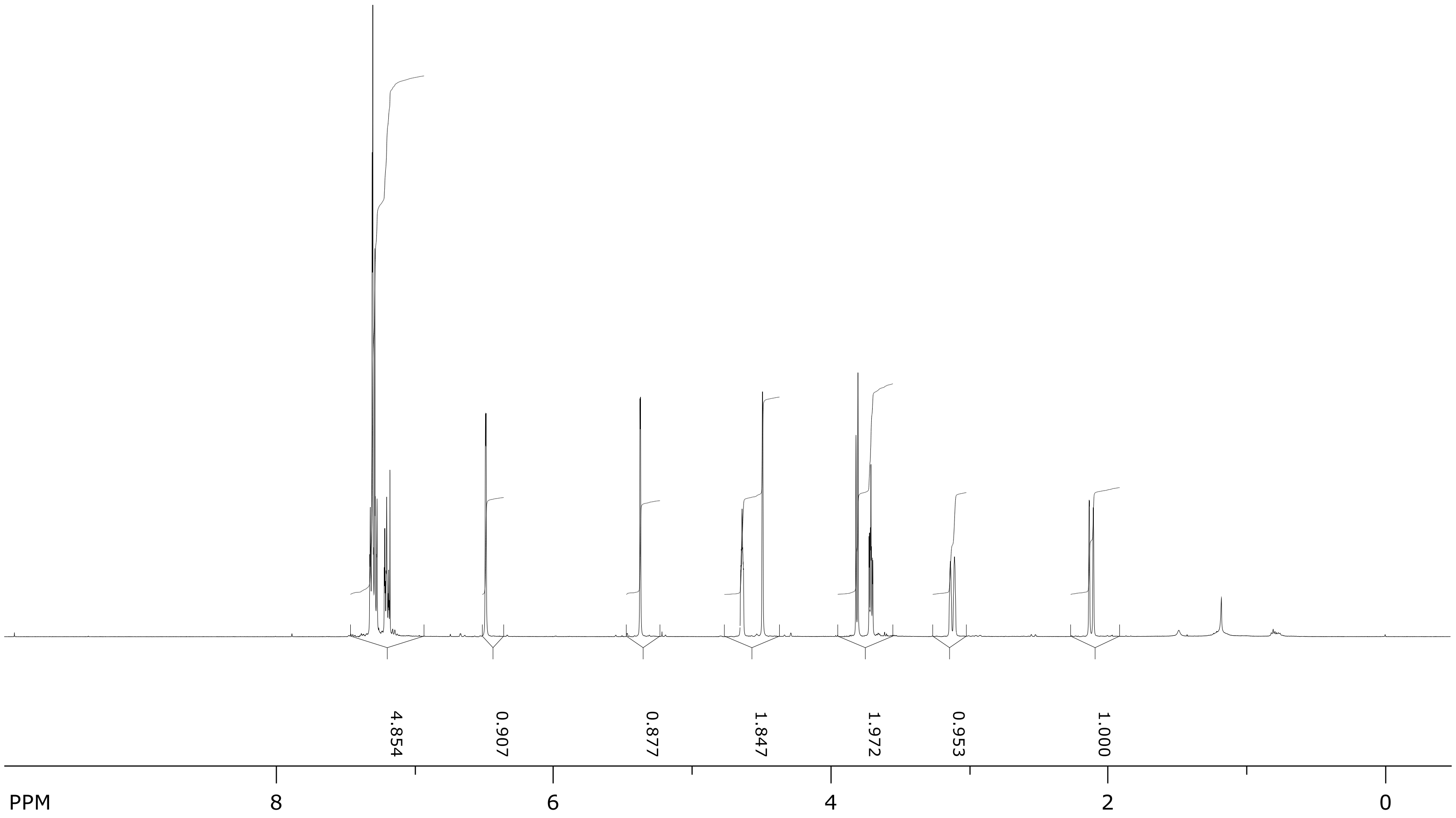

$^{13}C\{^{1}H\}$ **NMR of (1*S*,5*R*)-3-((E)-Benzylidene)-4-chloro-6,8-dioxabicyclo[3.2.1]octane (19)**

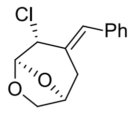

19

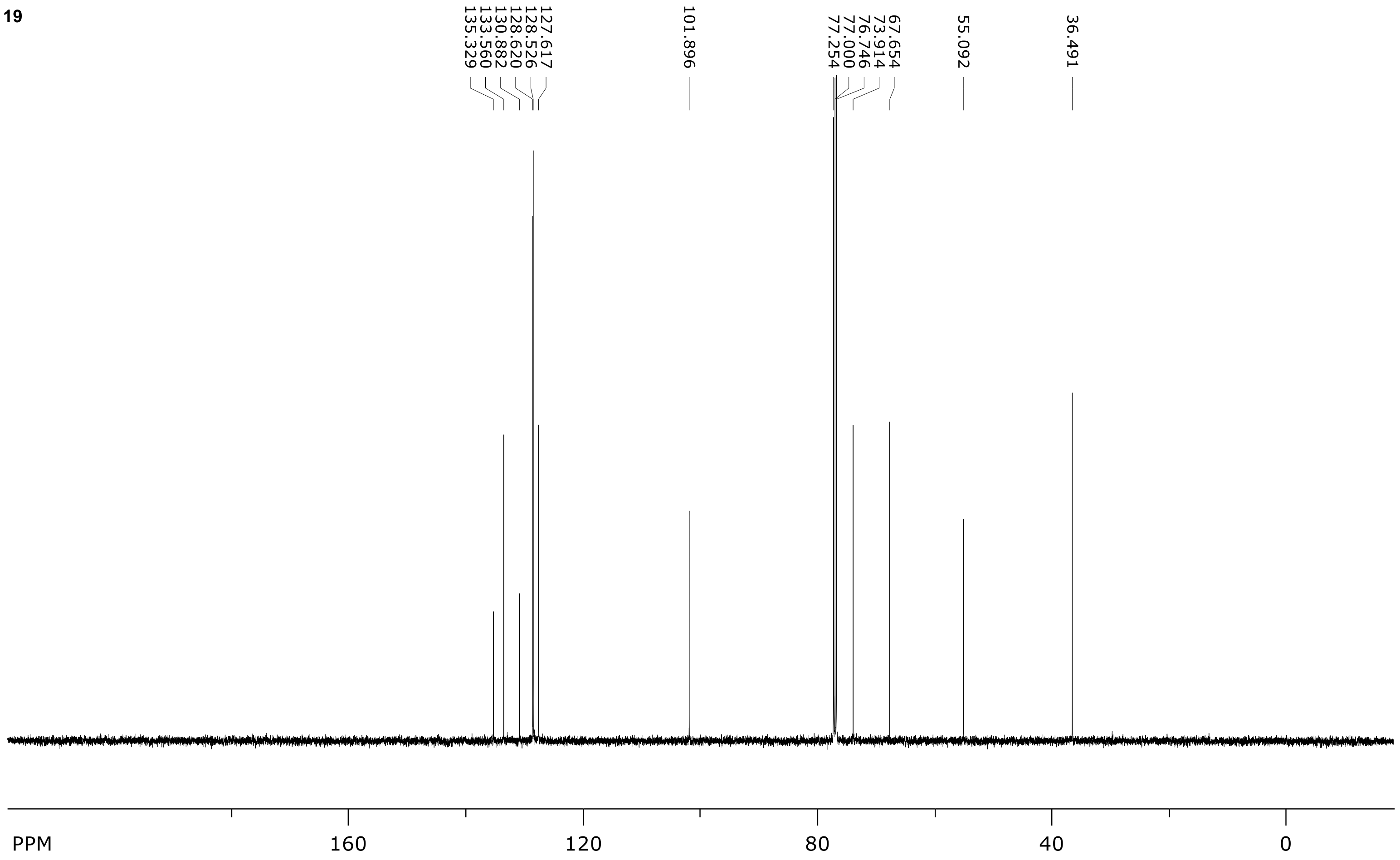

^{1}H NMR of (1*S*,5*R*)-3-(Chloro(phenyl)methyl)-6,8-dioxabicyclo[3.2.1]oct-3-ene (20)

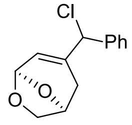

20 dr 91:9

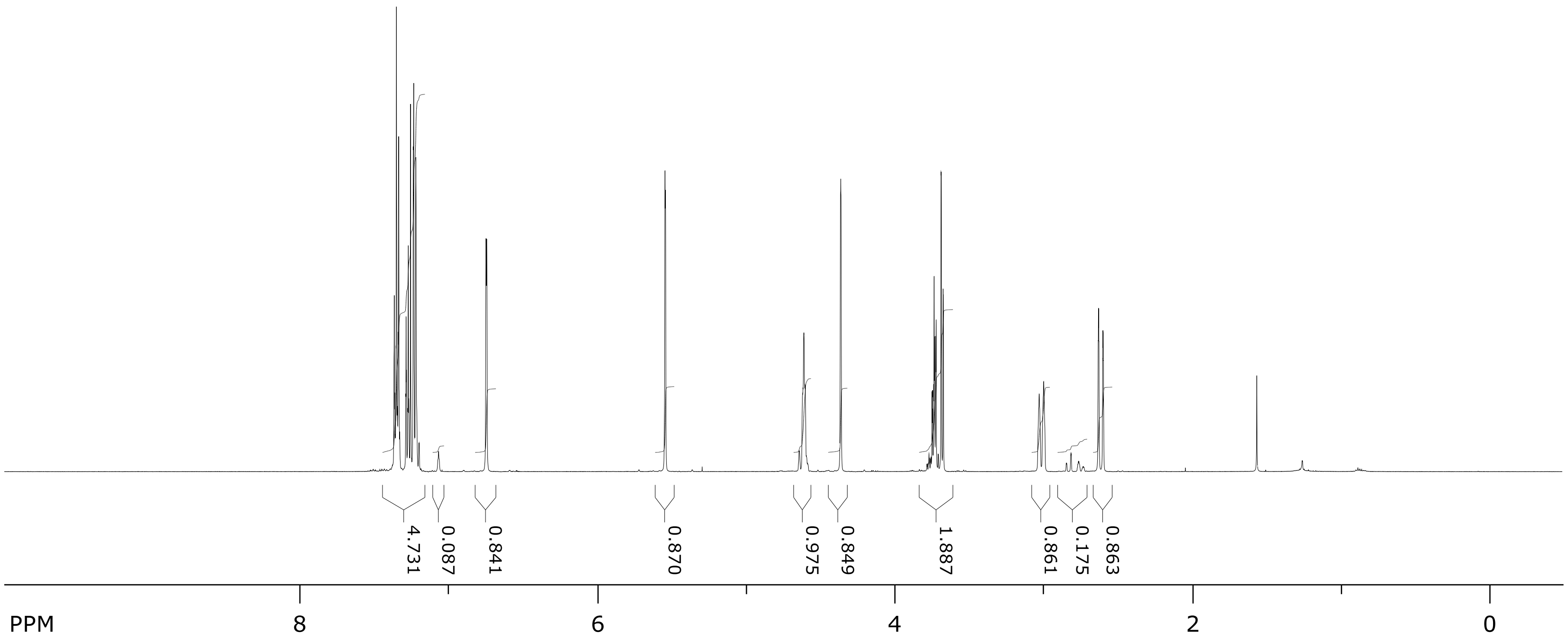

$^{13}C\{^1H\}$ NMR of (1*S*,5*R*)-3-(Chloro(phenyl)methyl)-6,8-dioxabicyclo[3.2.1]oct-3-ene (20)

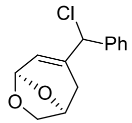

20 dr 91:9

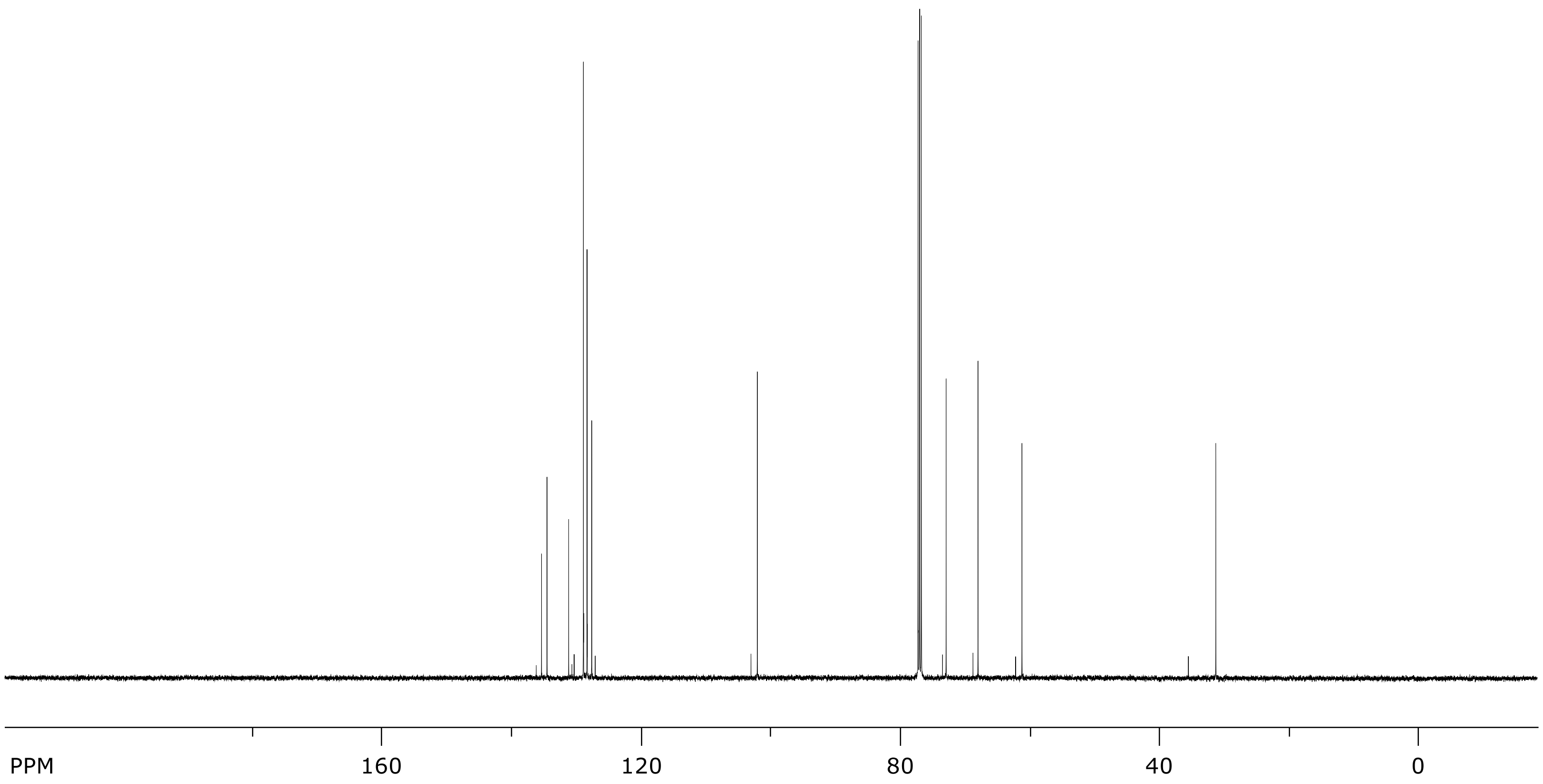

^{1}H NMR of (1*R*,2*R*,5*S*)-2-Allyl-7,7-dibenzyl-3,8-dioxabicyclo[3.2.1]octane (21)

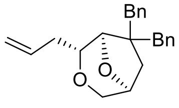

21

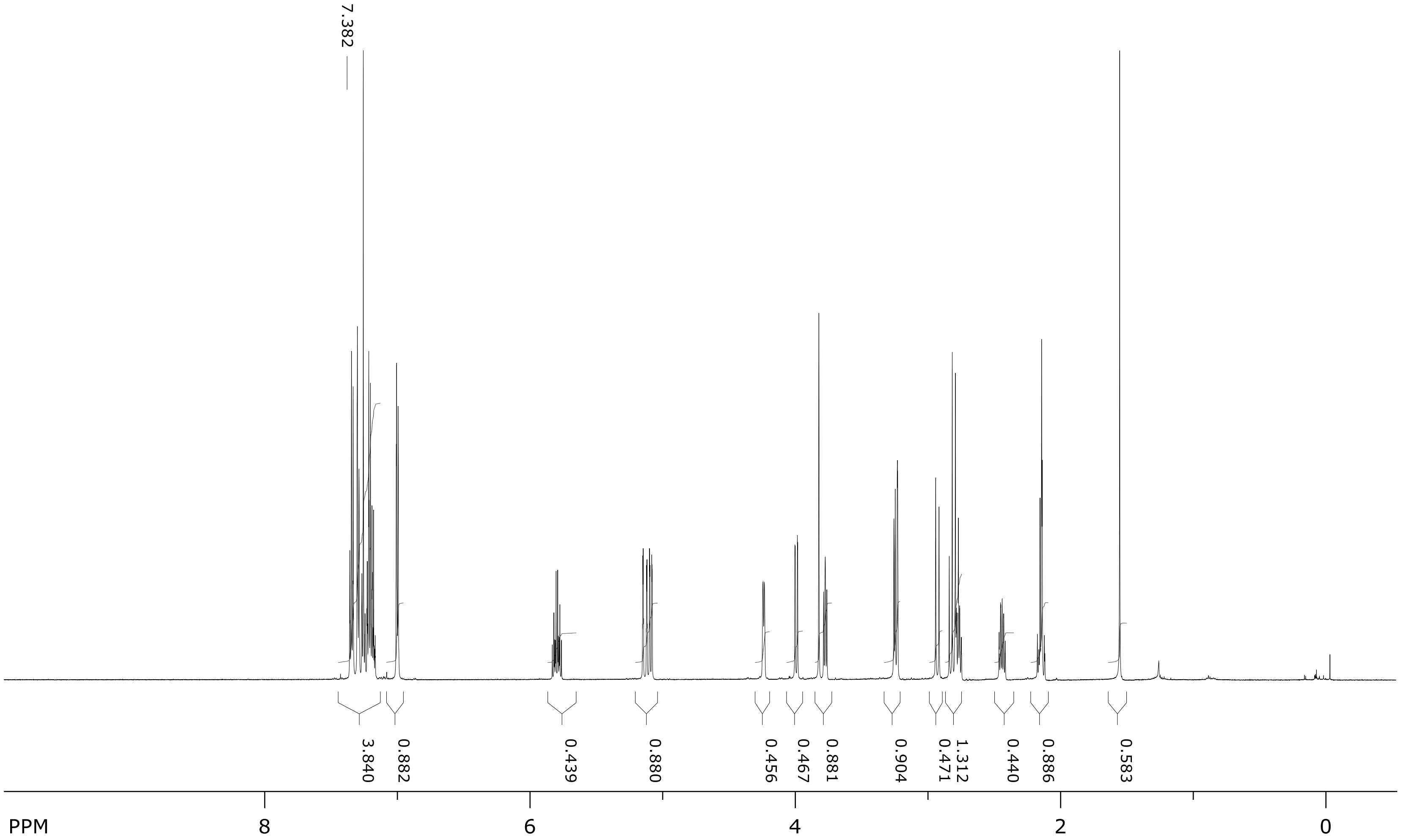

$^{13}C\{^1H\}$ NMR of (1*R*,2*R*,5*S*)-2-Allyl-7,7-dibenzyl-3,8-dioxabicyclo[3.2.1]octane (21)

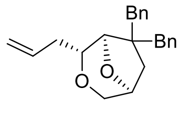

21

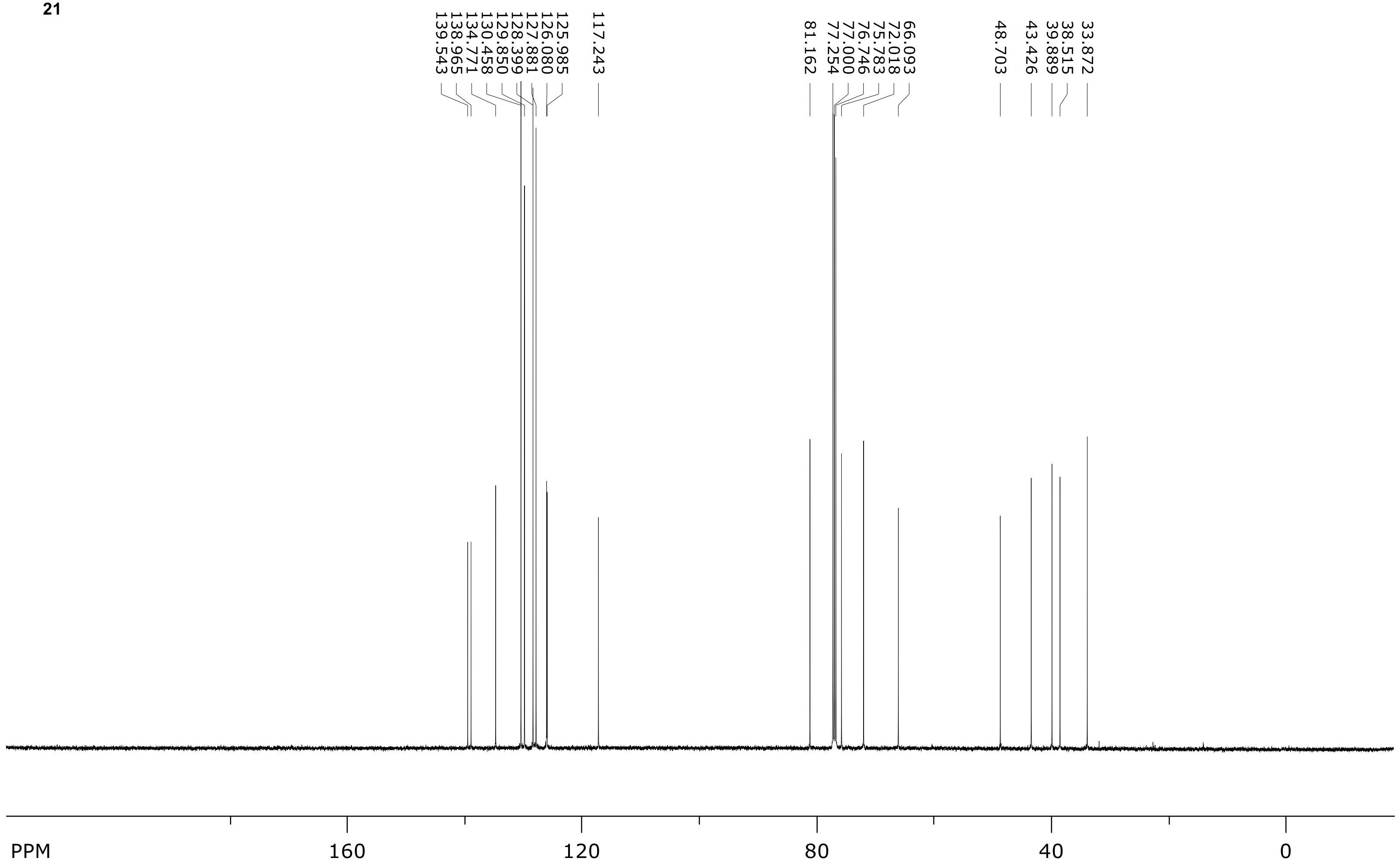

^{1}H NMR of (1*R*,2*R*,5*S*)-7,7-Dibenzyl-2-(4-methoxyphenyl)-3,8-dioxabicyclo[3.2.1]octane (22)

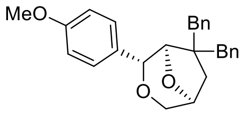

22

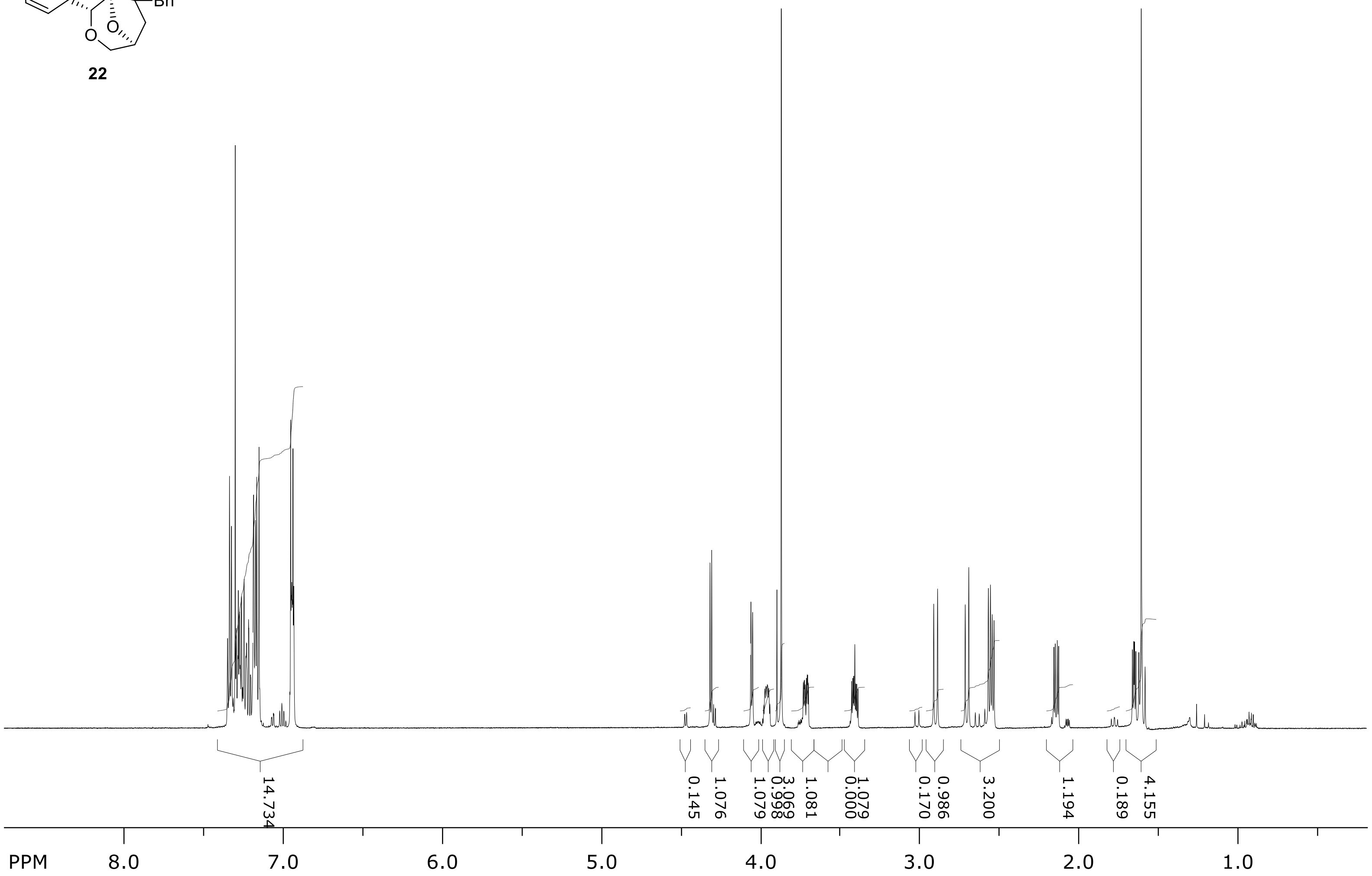

$^{13}C\{^{1}H\}$ NMR of (1*R*,2*R*,5*S*)-7,7-Dibenzyl-2-(4-methoxyphenyl)-3,8-dioxabicyclo[3.2.1]octane (22)

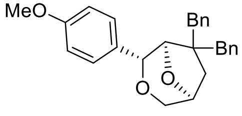

22

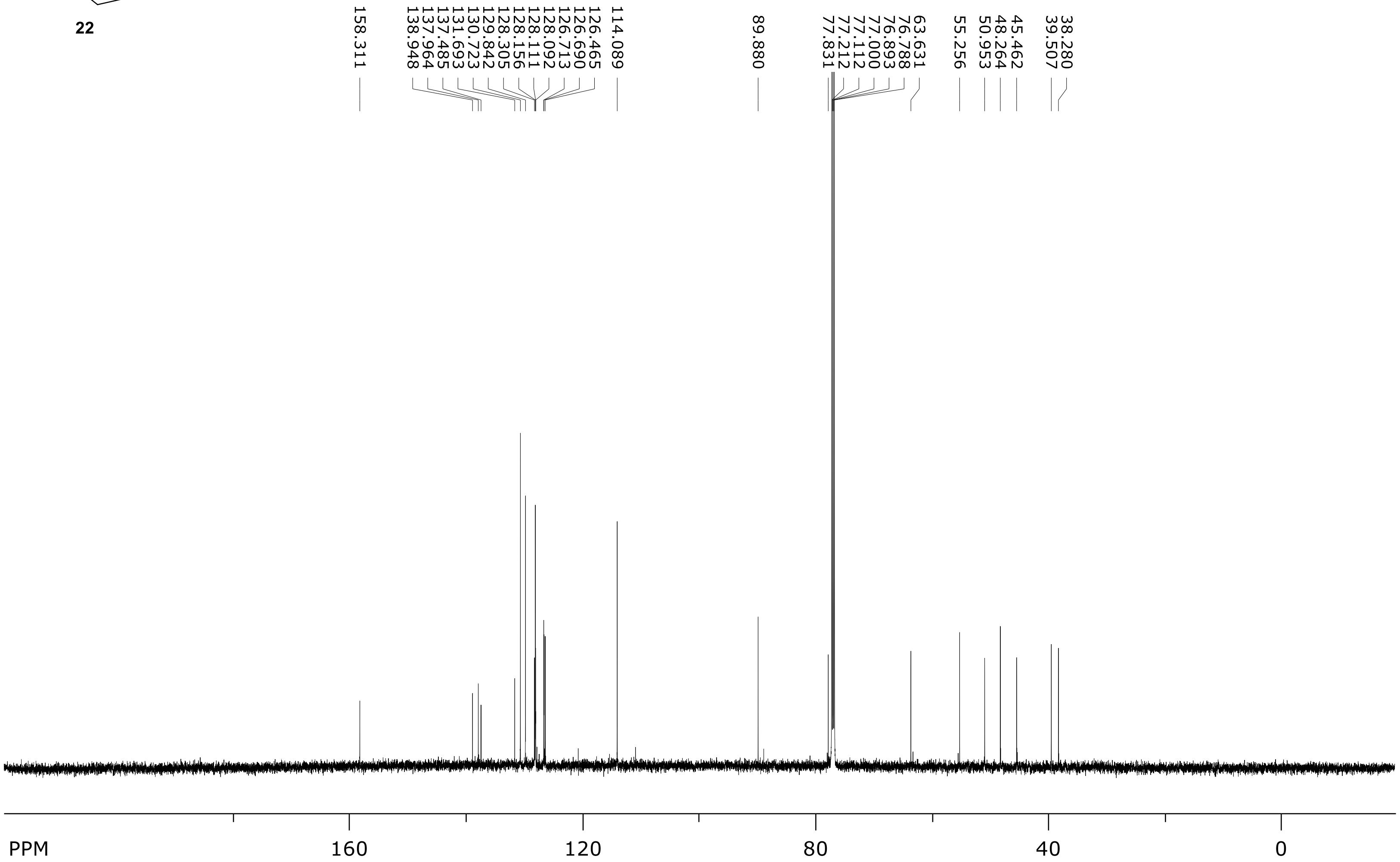

^{1}H NMR of (1*R*,2*R*,5*S*)-7,7-Dibenzyl-2-(4-phenoxyphenyl)-3,8-dioxabicyclo[3.2.1]octane (23)

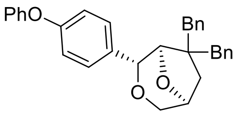

23

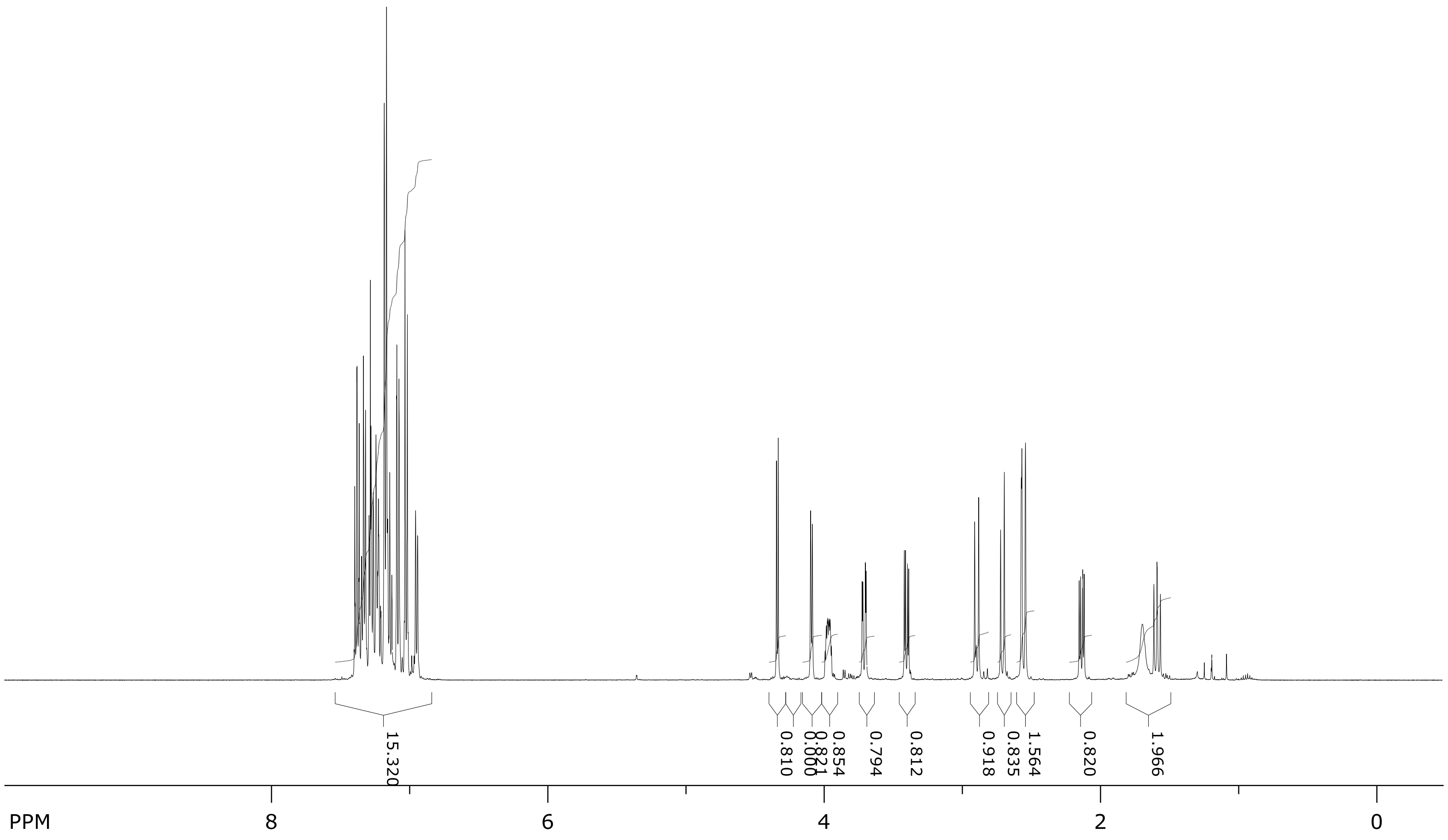

^{13}C{^{1}H} NMR of (1*R*,2*R*,5*S*)-7,7-Dibenzyl-2-(4-phenoxyphenyl)-3,8-dioxabicyclo[3.2.1]octane (23)

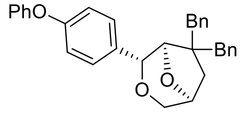

23

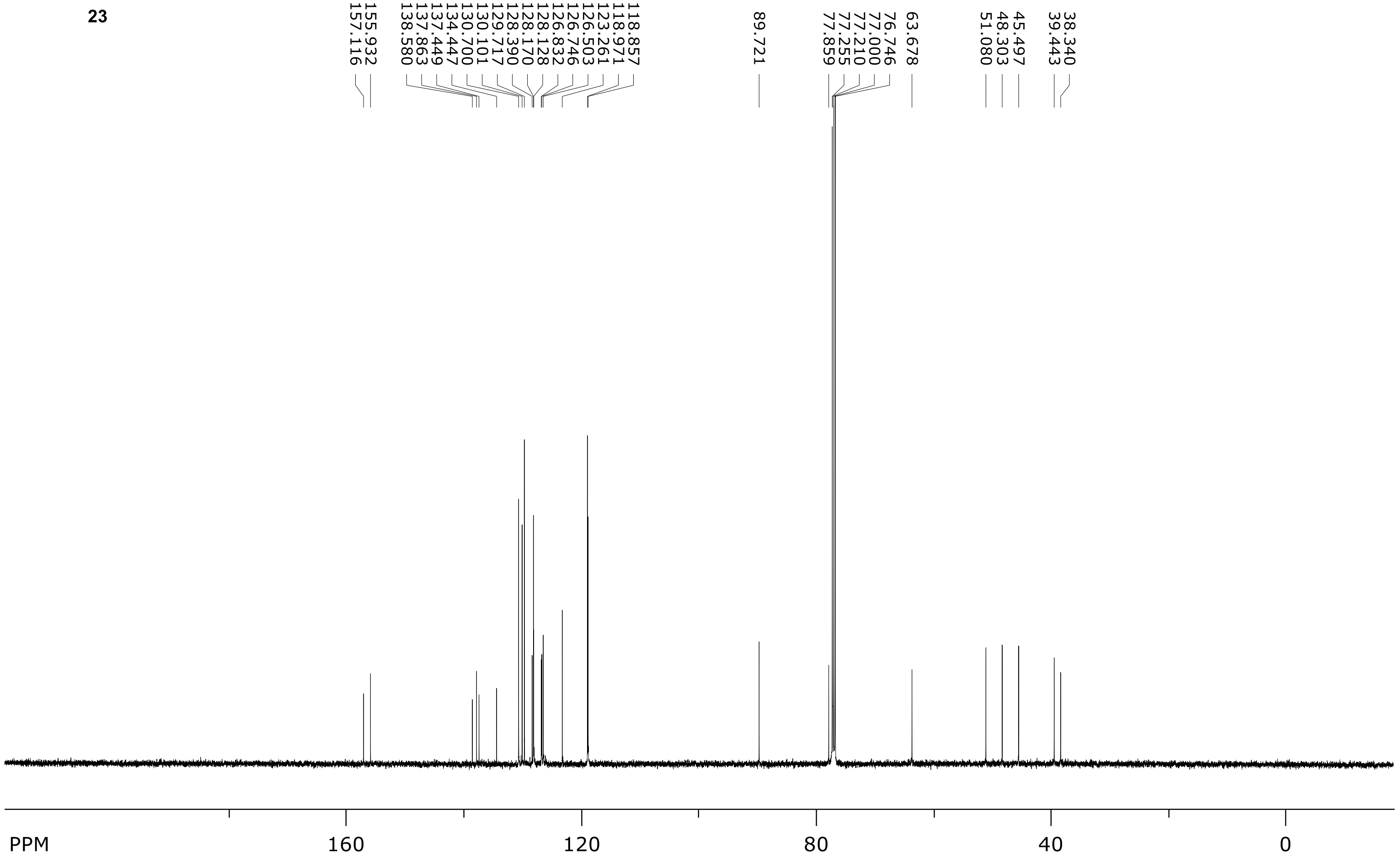

^{1}H NMR of (1*R*,5*S*)-7,7-Dibenzyl-3,8-dioxabicyclo[3.2.1]octan-2-one (24)

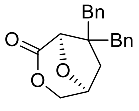

24

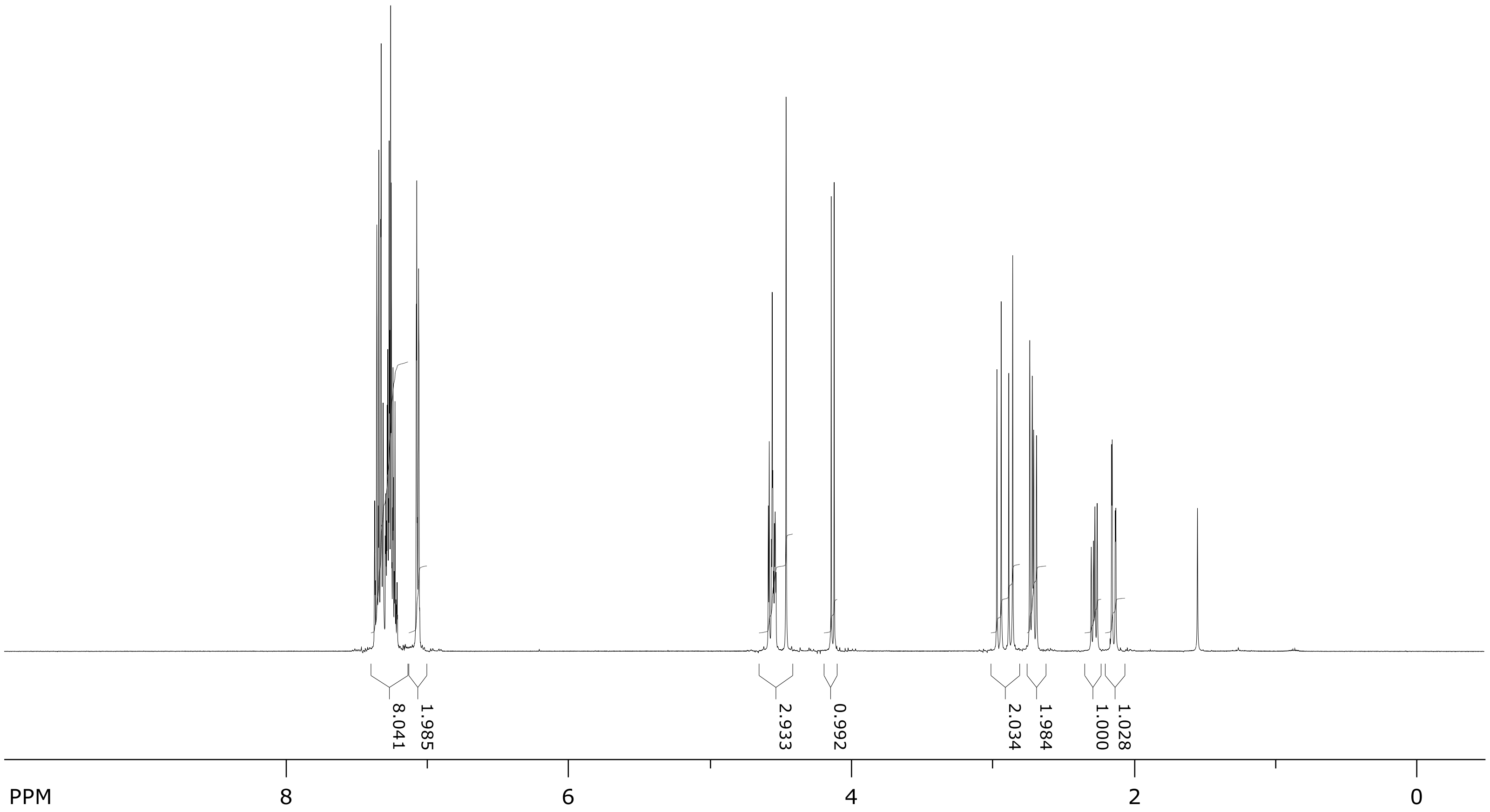

$^{13}C\{^{1}H\}$ NMR of (1*R*,5*S*)-7,7-Dibenzyl-3,8-dioxabicyclo[3.2.1]octan-2-one (24)

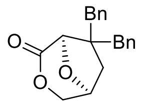

24

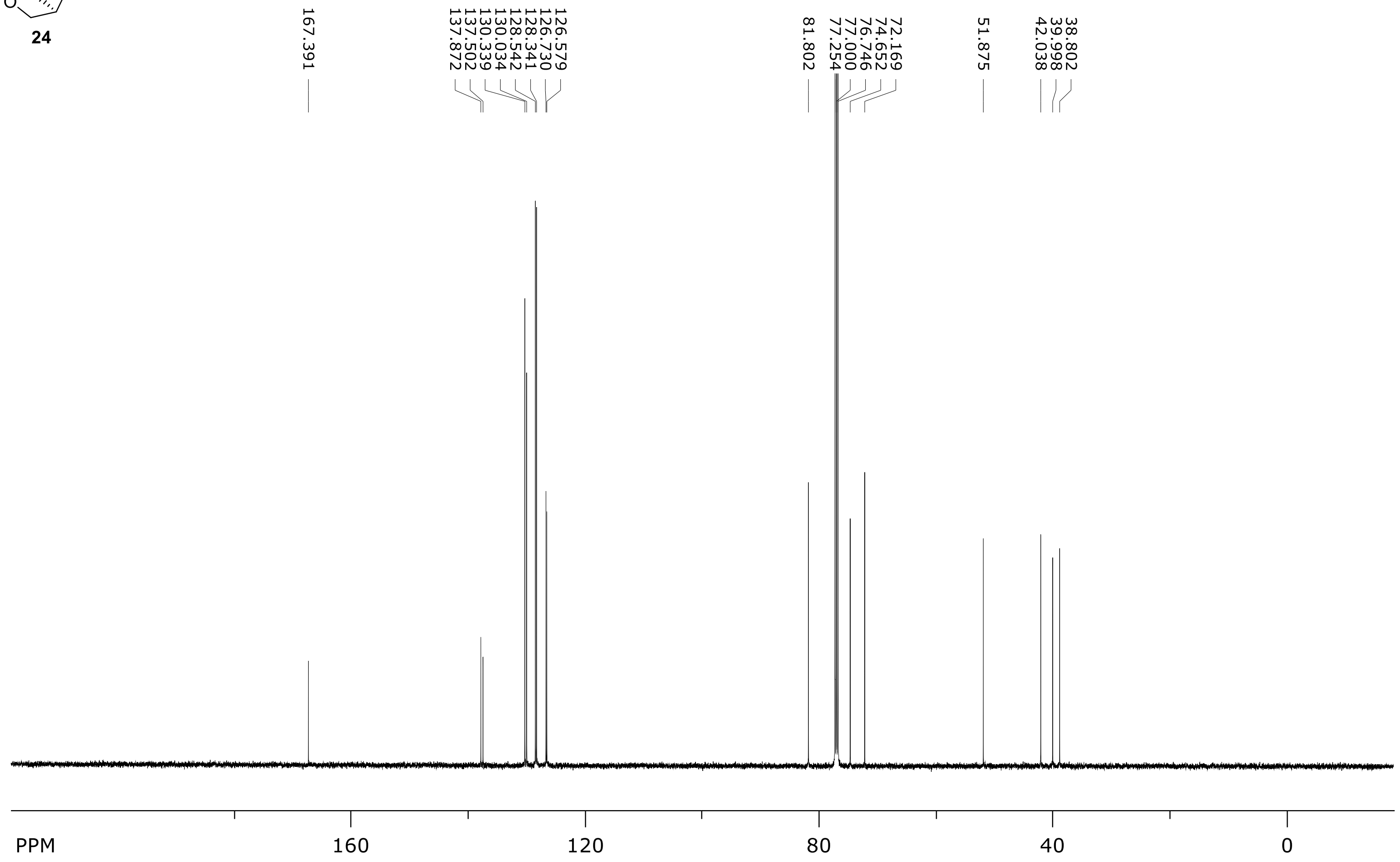

References

1. Klepp, J.; Sumby, C. J.; Greatrex, B. W. *Synlett* **2018**, *29* (11), 1441–1446.
2. Pedersen, J. M.; Pedersen, C. M. *Angew. Chem.***2021**, *60* (5), 2689–2693
3. Matsumoto, M.; Ishikawa, H.; Soya, Y.; Ozawa, T. *Heterocycles* **1994**, *38* (11), 2377.
4. CrysAlisPro 1.171.42.54a (Rigaku Oxford Diffraction, 2022).
5. (a) G. M. Sheldrick, A short history of SHELX. *Acta Cryst*., **2008**, A64, 112–122. (b) G. M. Sheldrick, SHELXT - Integrated space-group and crystal-structure determination. *Acta Cryst*., **2015**, A71, 3–8.
6. G. M. Sheldrick,. Crystal structure refinement with SHELXL. *Acta*. *Cryst*., **2015**, C71, 3–8.
7. L. J. Barbour, X-Seed 4: updates to a program for small-molecule supramolecular crystallography. *J. Appl. Cryst*., **2020**, 53, 1141–1146.
8. O. V. Dolomanov, L. J. Bourhis, R. J. Gildea, J. A. K. Howard and H. Puschmann, OLEX2: a complete structure solution, refinement and analysis program. *J. Appl. Cryst*., **2009**, 42, 339–341.
