# Supplementary material for: Skeletal rearrangement of 6,8-dioxabicyclo[3.2.1]octan-4-ols promoted by thionyl chloride or Appel conditions
Source: Beilstein J Org Chem. 2024 Apr 16;20:823–9. doi: 10.3762/bjoc.20.74 (PMC11035982; doi:10.3762/bjoc.20.74)
Supplement: File 2 — 1H and 13C NMR FIDs, HRMS spectra for all new compounds. [file Beilstein_J_Org_Chem-20-823-s002.zip › NMR files oxygen migration/10d/1H/pdata/1/cyroxOH_1_1.pdf]

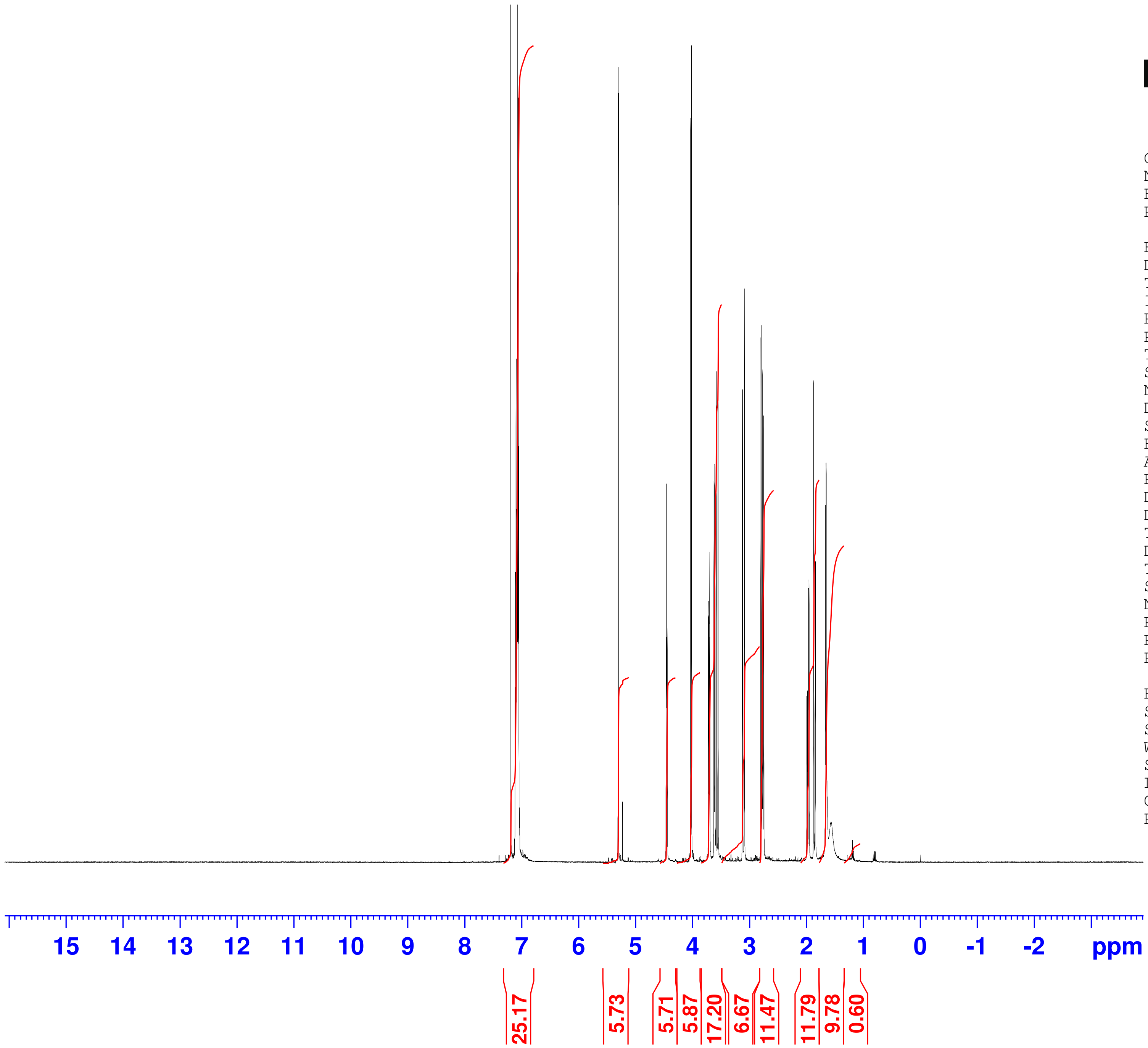

Current Data Parameters

NAME	cyroxOH	
EXPNO	1	
PROCNO	1	

F2 - Acquisition Parameters

Date_	20240103	
Time	17.00	h
INSTRUM	spect	
PROBHD	Z124627_0011 (	
PULPROG	zg30	
TD	65536	
SOLVENT	CDCl3	
NS	16	
DS	2	
SWH	10000.000	Hz
FIDRES	0.305176	Hz
AQ	3.2767999	sec
RG	191.36	
DW	50.000	usec
DE	13.04	usec
TE	297.7	K
D1	1.00000000	sec
TD0	1	
SFO1	500.1630885	MHz
NUC1	1H	
P0	5.00	usec
P1	15.00	usec
PLW1	9.50000000	W

F2 - Processing parameters

SI	65536	
SF	500.1600479	MHz
WDW	EM	
SSB	0	
LB	0.30	Hz
GB	0	
PC	1.00	
