# Supplementary material for: Skeletal rearrangement of 6,8-dioxabicyclo[3.2.1]octan-4-ols promoted by thionyl chloride or Appel conditions
Source: Beilstein J Org Chem. 2024 Apr 16;20:823–9. doi: 10.3762/bjoc.20.74 (PMC11035982; doi:10.3762/bjoc.20.74)
Supplement: File 2 — 1H and 13C NMR FIDs, HRMS spectra for all new compounds. [file Beilstein_J_Org_Chem-20-823-s002.zip › NMR files oxygen migration/10e/10e HRMS.pdf]

Single Mass Analysis
Tolerance = 5.0 PPM / DBE: min = -1.5, max = 120.0
Element prediction: Off
Number of isotope peaks used for i-FIT = 3

Monoisotopic Mass, Even Electron Ions
16 formula(e) evaluated with 1 results within limits (up to 20 closest results for each mass)
Elements Used:
C: 0-20 H: 0-22 O: 0-3 Na: 0-1

HBr5
UNE BGreatrex HBr5 18 (0.442) Cm (16:37)

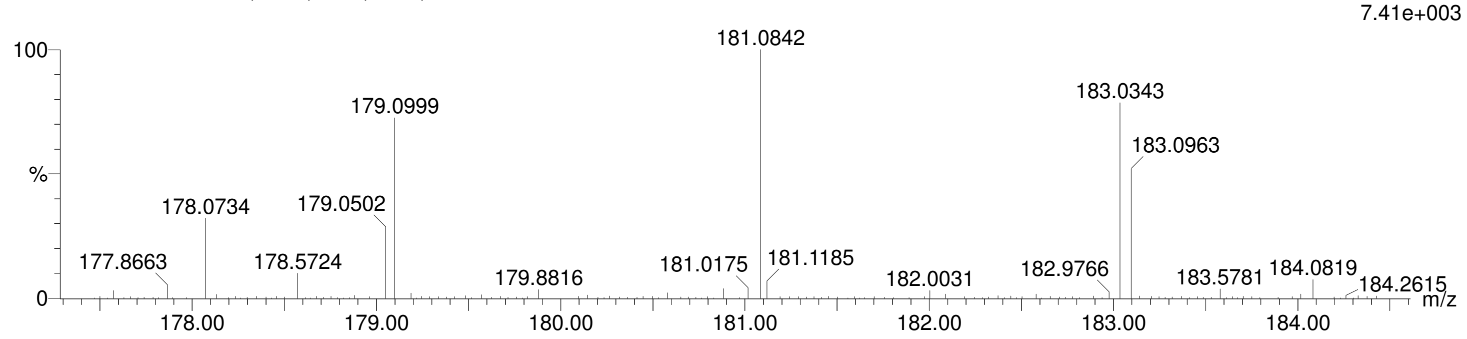

Minimum:				-1.5			
Maximum:		55.0	5.0	120.0			

Mass	Calc. Mass	mDa	PPM	DBE	i-FIT	i-FIT (Norm)	Formula
181.0842	181.0841	0.1	0.6	1.5	376.2	0.0	C8 H14 O3 Na
