# Supplementary material for: Skeletal rearrangement of 6,8-dioxabicyclo[3.2.1]octan-4-ols promoted by thionyl chloride or Appel conditions
Source: Beilstein J Org Chem. 2024 Apr 16;20:823–9. doi: 10.3762/bjoc.20.74 (PMC11035982; doi:10.3762/bjoc.20.74)
Supplement: File 2 — 1H and 13C NMR FIDs, HRMS spectra for all new compounds. [file Beilstein_J_Org_Chem-20-823-s002.zip › NMR files oxygen migration/10f/13C/pdata/1/4OMe-alcohol_2_1.pdf]

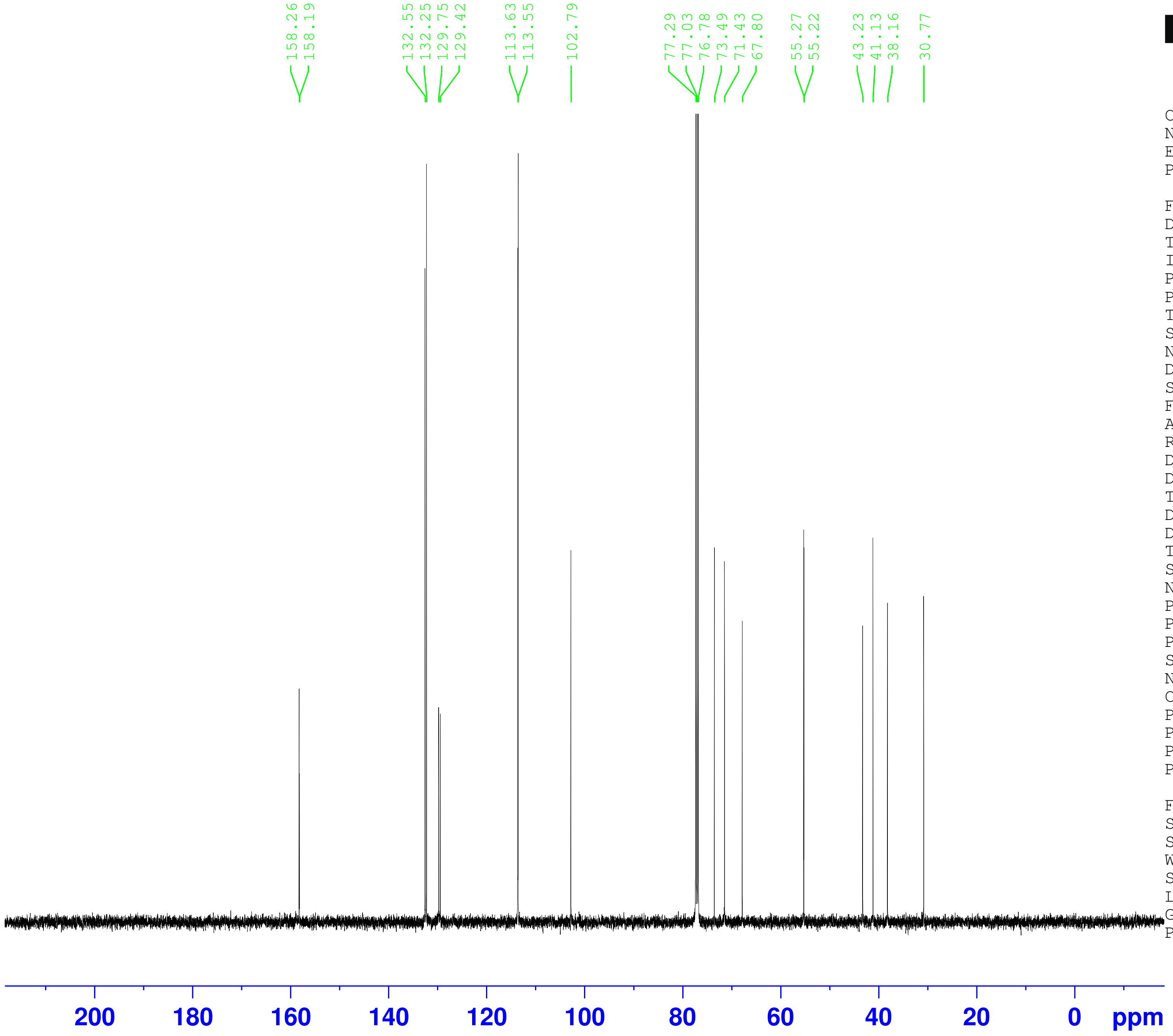

BRUKER

```
Current Data Parameters
NAME        4OMe-alcohol
EXPNO                  2
PROCNO                 1

F2 - Acquisition Parameters
Date_           20230908
Time               19.51 h
INSTRUM            spect
PROBHD     Z124627_0019 (
PULPROG          zgpg30
TD                65536
SOLVENT           CDCl3
NS                 1024
DS                    4
SWH           29761.904 Hz
FIDRES         0.908261 Hz
AQ            1.1010048 sec
RG               191.36
DW               16.800 usec
DE                 6.50 usec
TE                298.0 K
D1           2.00000000 sec
D11          0.03000000 sec
TD0                   1
SFO1        125.7779086 MHz
NUC1                13C
P0                 3.33 usec
P1                10.00 usec
PLW1        41.00000000 W
SFO2        500.1620006 MHz
NUC2                 1H
CPDPRG[2        waltz65
PCPD2             80.00 usec
PLW2        11.00000000 W
PLW12        0.37393999 W
PLW13        0.18809000 W

F2 - Processing parameters
SI                32768
SF          125.7653320 MHz
WDW                  EM
SSB                   0
LB                 1.00 Hz
GB                    0
PC                 1.40
```
