# Supplementary figures and images for: Skeletal rearrangement of 6,8-dioxabicyclo[3.2.1]octan-4-ols promoted by thionyl chloride or Appel conditions
Source: Beilstein J Org Chem. 2024 Apr 16;20:823–9. doi: 10.3762/bjoc.20.74 (PMC11035982; doi:10.3762/bjoc.20.74)

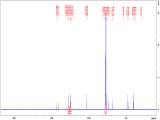

Supplement: File 2 — 1H and 13C NMR FIDs, HRMS spectra for all new compounds. [file Beilstein_J_Org_Chem-20-823-s002.zip › NMR files oxygen migration/10d/13C/pdata/1/thumb.png]

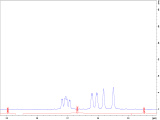

Supplement: File 2 — 1H and 13C NMR FIDs, HRMS spectra for all new compounds. [file Beilstein_J_Org_Chem-20-823-s002.zip › NMR files oxygen migration/10d/1H/pdata/1/thumb.png]

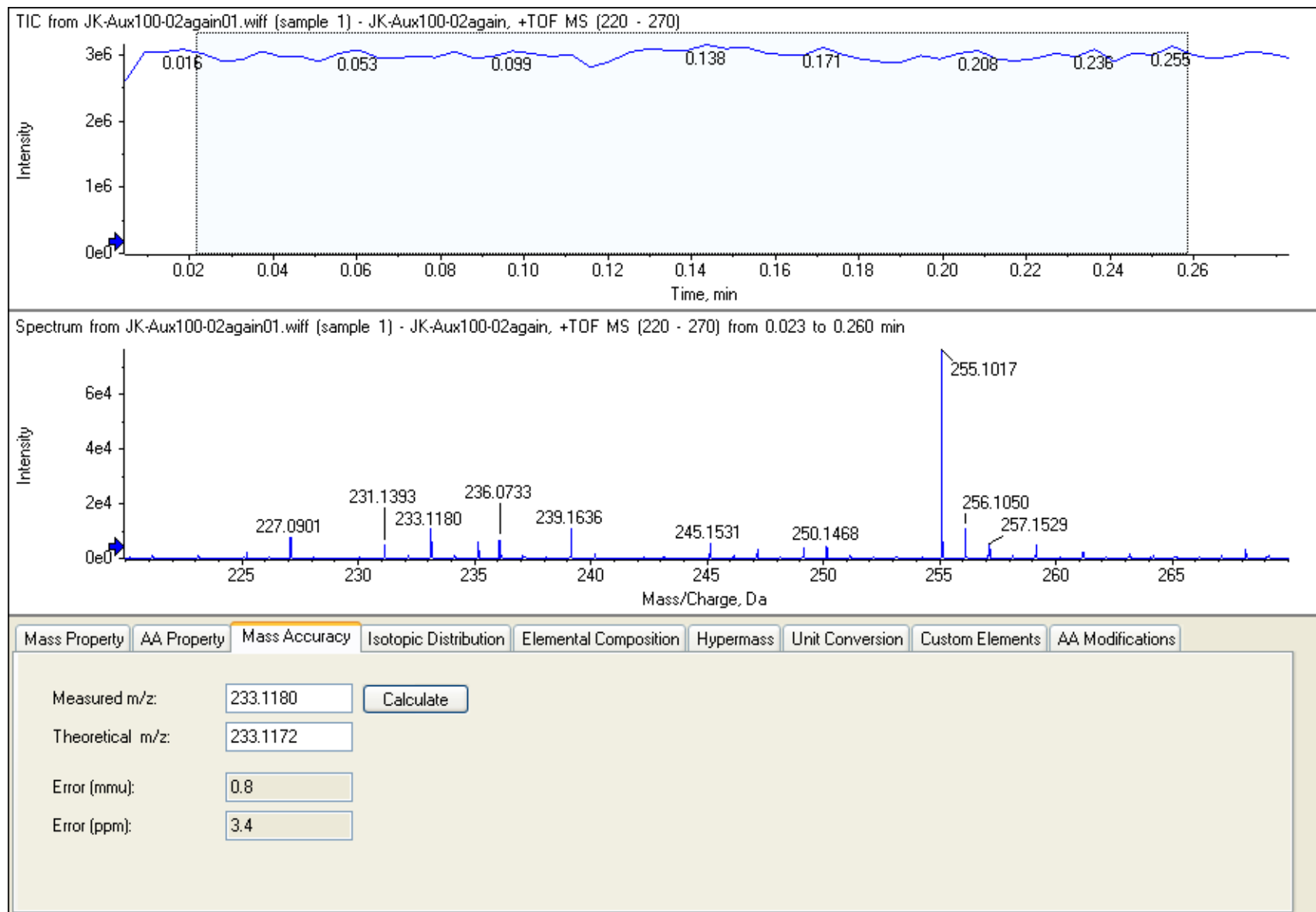

Supplement: File 2 — 1H and 13C NMR FIDs, HRMS spectra for all new compounds. [file Beilstein_J_Org_Chem-20-823-s002.zip › NMR files oxygen migration/10d/HRMS 10d.pdf]
