# Supplementary material for: Skeletal rearrangement of 6,8-dioxabicyclo[3.2.1]octan-4-ols promoted by thionyl chloride or Appel conditions
Source: Beilstein J Org Chem. 2024 Apr 16;20:823–9. doi: 10.3762/bjoc.20.74 (PMC11035982; doi:10.3762/bjoc.20.74)
Supplement: File 2 — 1H and 13C NMR FIDs, HRMS spectra for all new compounds. [file Beilstein_J_Org_Chem-20-823-s002.zip › NMR files oxygen migration/11a/13C/pdata/1/ABC_OM_1-1 (4-6)_2_1.pdf]

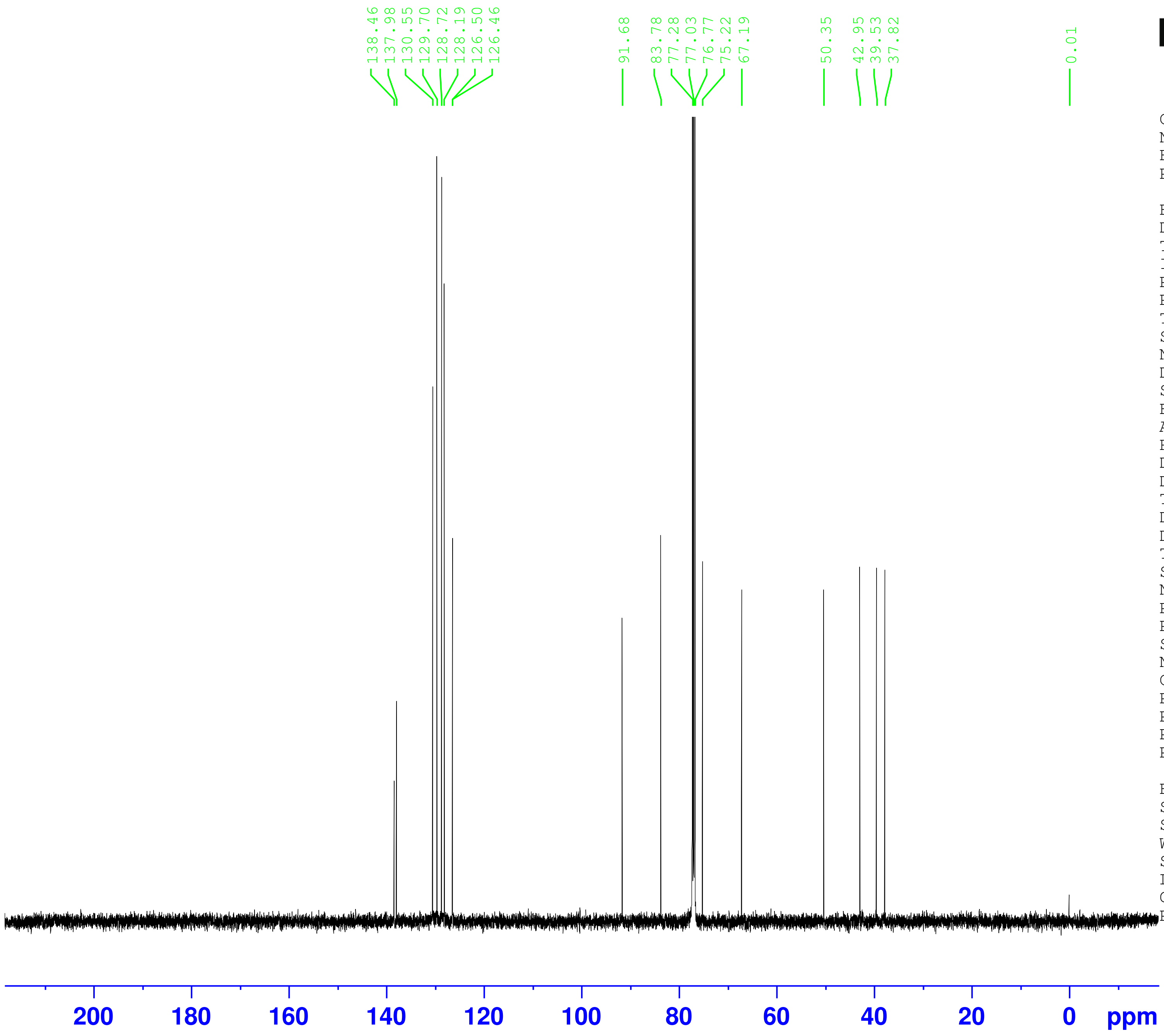

BRUKER

Current Data Parameters
NAME ABC_OM_1-1 (4-6)
EXPNO 2
PROCNO 1

F2 - Acquisition Parameters
Date_ 20190711
Time 6.30 h
INSTRUM spect
PROBHD Z119470_0196 (
PULPROG zgpg30
TD 65536
SOLVENT CDCl3
NS 2000
DS 4
SWH 29761.904 Hz
FIDRES 0.908261 Hz
AQ 1.1010048 sec
RG 191.36
DW 16.800 usec
DE 6.50 usec
TE 295.0 K
D1 2.00000000 sec
D11 0.03000000 sec
TD0 1
SFO1 125.7779086 MHz
NUC1 13C
P1 10.00 usec
PLW1 78.00000000 W
SFO2 500.1620006 MHz
NUC2 1H
CPDPRG[2 waltz16
PCPD2 80.00 usec
PLW2 22.00000000 W
PLW12 0.35764000 W
PLW13 0.17989001 W

F2 - Processing parameters
SI 32768
SF 125.7653320 MHz
WDW EM
SSB 0
LB 1.00 Hz
GB 0
PC 1.40
