# Supplementary material for: Skeletal rearrangement of 6,8-dioxabicyclo[3.2.1]octan-4-ols promoted by thionyl chloride or Appel conditions
Source: Beilstein J Org Chem. 2024 Apr 16;20:823–9. doi: 10.3762/bjoc.20.74 (PMC11035982; doi:10.3762/bjoc.20.74)
Supplement: File 2 — 1H and 13C NMR FIDs, HRMS spectra for all new compounds. [file Beilstein_J_Org_Chem-20-823-s002.zip › NMR files oxygen migration/11a/1H/pdata/1/ABC_OM_1-1 (4-6)_3_1.pdf]

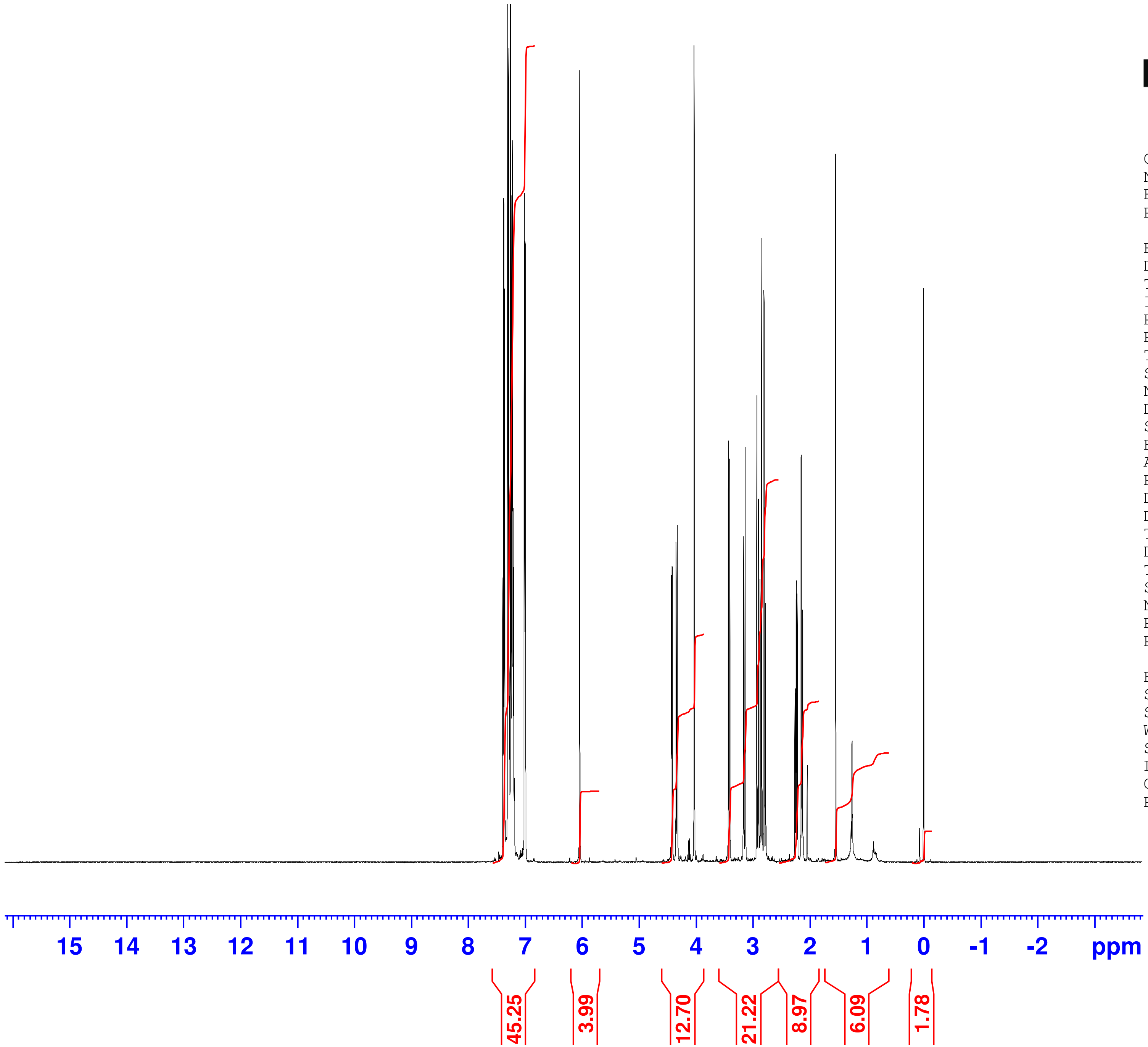


```
Current Data Parameters
NAME       ABC_OM_1-1 (4-6)
EXPNO                     3
PROCNO                    1

F2 - Acquisition Parameters
Date_              20190711
Time                   6.32 h
INSTRUM               spect
PROBHD     Z119470_0196 (
PULPROG                zg30
TD                    65536
SOLVENT               CDCl3
NS                       16
DS                        2
SWH               10000.000 Hz
FIDRES             0.305176 Hz
AQ                3.2767999 sec
RG                   123.82
DW                   50.000 usec
DE                     6.50 usec
TE                    295.0 K
D1               1.00000000 sec
TD0                       1
SFO1            500.1630885 MHz
NUC1                     1H
P1                    10.20 usec
PLW1            22.00000000 W

F2 - Processing parameters
SI                    65536
SF              500.1600131 MHz
WDW                      EM
SSB        0
LB                     0.30 Hz
GB         0
PC                     1.00
```
