# Supplementary material for: Skeletal rearrangement of 6,8-dioxabicyclo[3.2.1]octan-4-ols promoted by thionyl chloride or Appel conditions
Source: Beilstein J Org Chem. 2024 Apr 16;20:823–9. doi: 10.3762/bjoc.20.74 (PMC11035982; doi:10.3762/bjoc.20.74)
Supplement: File 2 — 1H and 13C NMR FIDs, HRMS spectra for all new compounds. [file Beilstein_J_Org_Chem-20-823-s002.zip › NMR files oxygen migration/11c/1H/pdata/1/email_cyrBnMe2RCl_1_1.pdf]

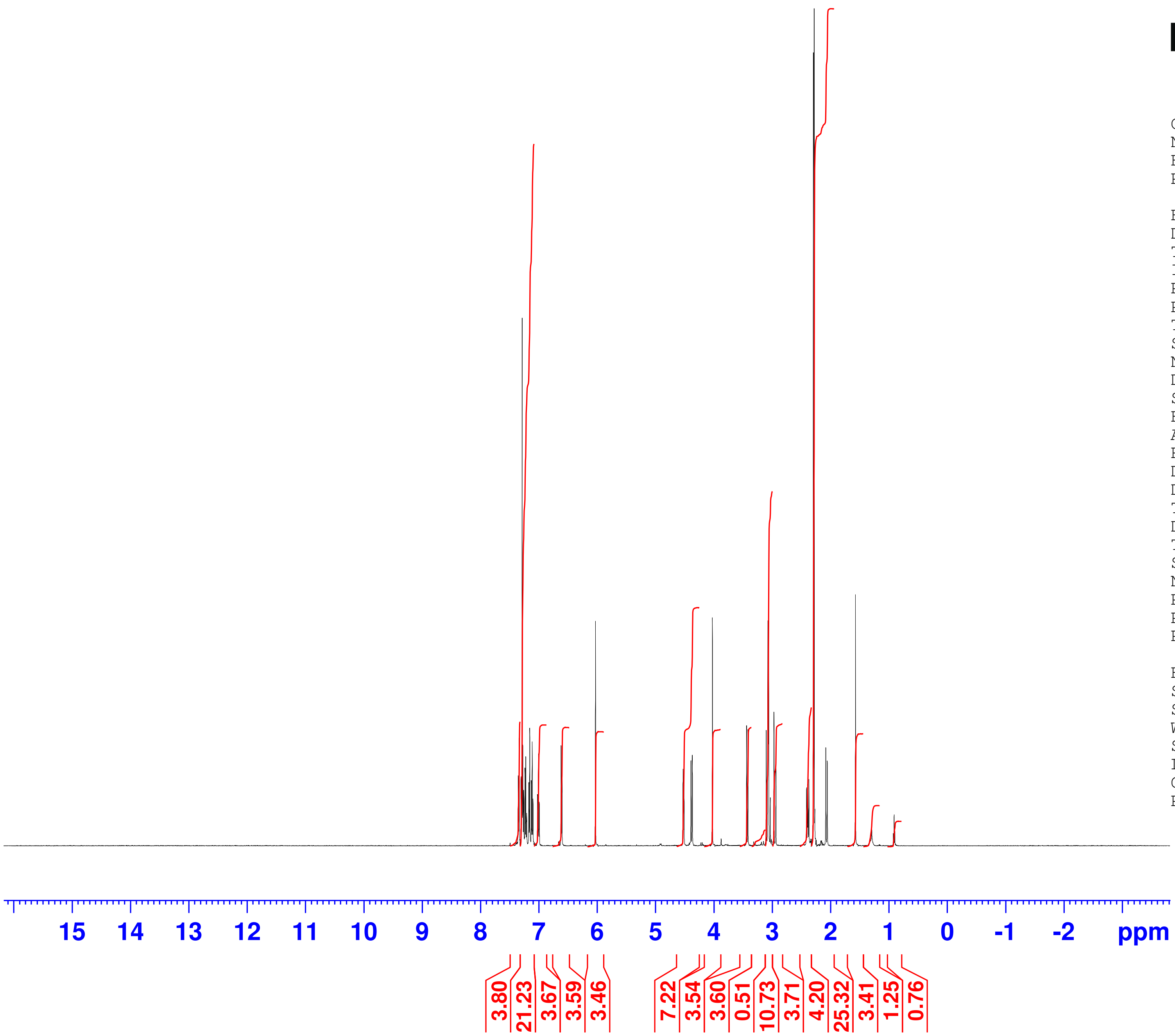

BRUKER

```
Current Data Parameters
NAME          cyrBnMe2RCl
EXPNO                   1
PROCNO                  1

F2 - Acquisition Parameters
Date_            20230930
Time                12.46 h
INSTRUM             spect
PROBHD   Z124627_0019 (
PULPROG              zg30
TD                  65536
SOLVENT             CDCl3
NS                     16
DS                      2
SWH             10000.000 Hz
FIDRES           0.305176 Hz
AQ              3.2767999 sec
RG                 191.36
DW                 50.000 usec
DE                  13.08 usec
TE                  298.0 K
D1             1.00000000 sec
TD0                     1
SFO1          500.1630885 MHz
NUC1                   1H
P0                   4.92 usec
P1                  14.75 usec
PLW1          11.00000000 W

F2 - Processing parameters
SI                  65536
SF            500.1600000 MHz
WDW                    EM
SSB                     0
LB                   0.30 Hz
GB                      0
PC                   1.00
```
