# Supplementary material for: Skeletal rearrangement of 6,8-dioxabicyclo[3.2.1]octan-4-ols promoted by thionyl chloride or Appel conditions
Source: Beilstein J Org Chem. 2024 Apr 16;20:823–9. doi: 10.3762/bjoc.20.74 (PMC11035982; doi:10.3762/bjoc.20.74)
Supplement: File 2 — 1H and 13C NMR FIDs, HRMS spectra for all new compounds. [file Beilstein_J_Org_Chem-20-823-s002.zip › NMR files oxygen migration/11c/ESIMS.pdf]

MS Spectrum

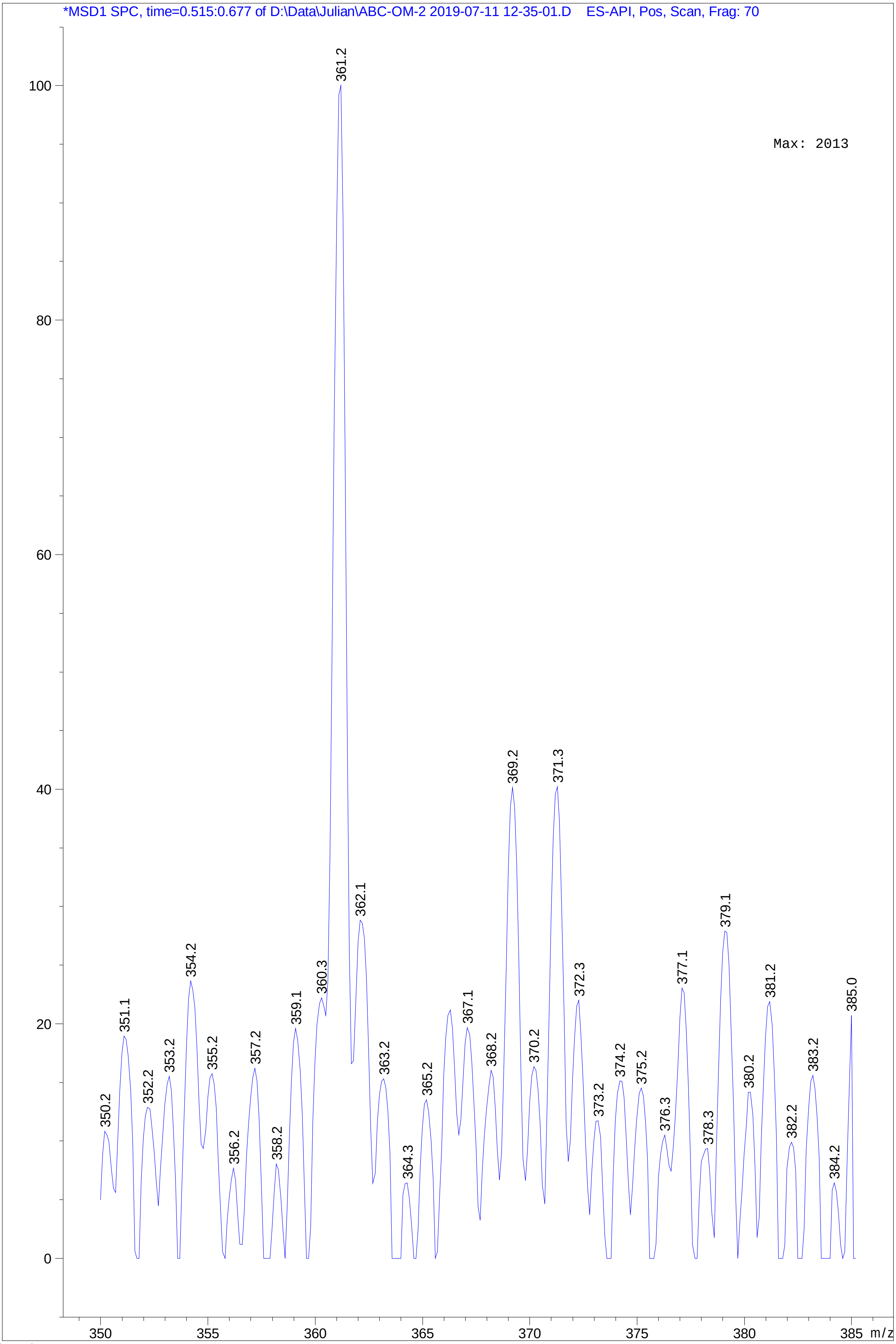
*MSD1 SPC, time=0.515:0.677 of D:\Data\Julian\ABC-OM-2 2019-07-11 12-35-01.D ES-API, Pos, Scan, Frag: 70
Max: 2013
361.2
369.2
371.3
362.1
379.1
354.2
360.3
377.1
372.3
381.2
385.0
351.1
367.1
359.1
370.2
357.2
368.2
353.2
355.2
363.2
374.2
375.2
383.2
352.2
365.2
373.2
350.2
380.2
376.3
378.3
382.2
356.2
358.2
364.3
384.2
100
80
60
40
20
0
350
355
360
365
370
375
380
385
m/z
