# Supplementary material for: Skeletal rearrangement of 6,8-dioxabicyclo[3.2.1]octan-4-ols promoted by thionyl chloride or Appel conditions
Source: Beilstein J Org Chem. 2024 Apr 16;20:823–9. doi: 10.3762/bjoc.20.74 (PMC11035982; doi:10.3762/bjoc.20.74)
Supplement: File 2 — 1H and 13C NMR FIDs, HRMS spectra for all new compounds. [file Beilstein_J_Org_Chem-20-823-s002.zip › NMR files oxygen migration/11c/IR.pdf]

PerkinElmer Spectrum Version 10.4.00
Saturday, 13 July 2019 7:35 PM

Analyst	Student
Date	Saturday, 13 July 2019 7:35 PM

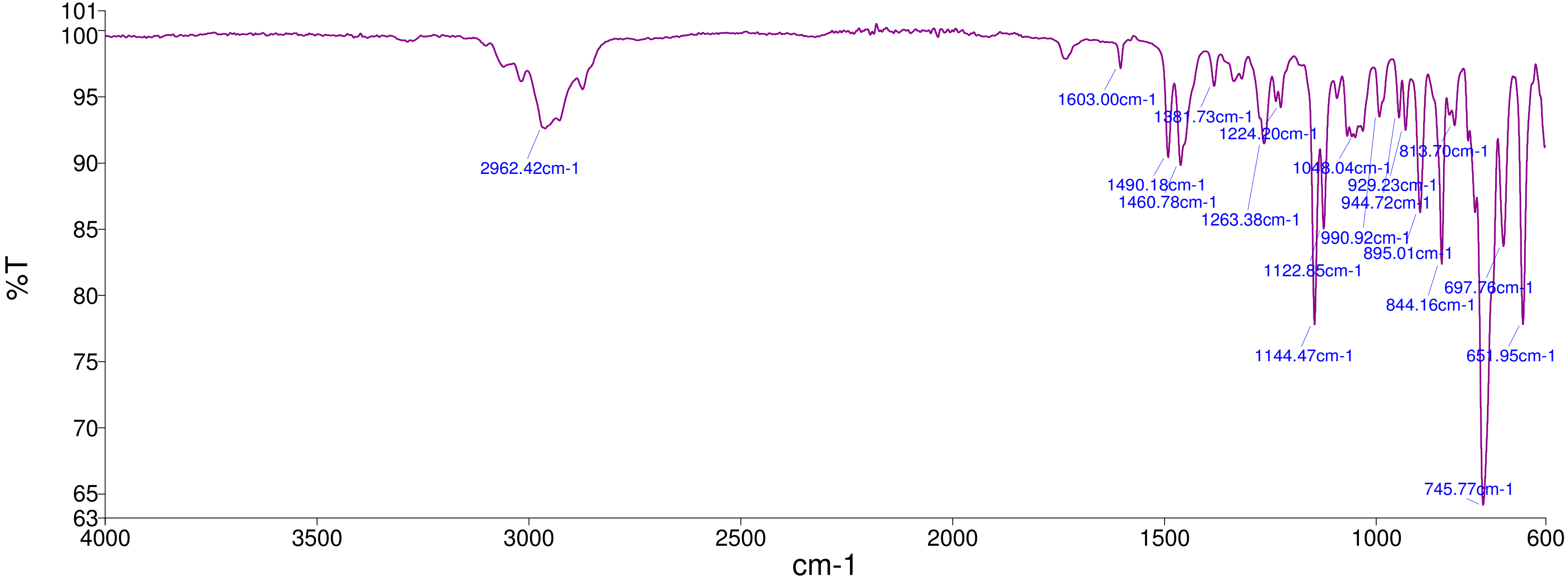

Sample Name	Description	Quality Checks
IR-ABC-OM-2	020	The Quality Checks do not report any warnings for the sample.
