# Supplementary material for: Skeletal rearrangement of 6,8-dioxabicyclo[3.2.1]octan-4-ols promoted by thionyl chloride or Appel conditions
Source: Beilstein J Org Chem. 2024 Apr 16;20:823–9. doi: 10.3762/bjoc.20.74 (PMC11035982; doi:10.3762/bjoc.20.74)
Supplement: File 2 — 1H and 13C NMR FIDs, HRMS spectra for all new compounds. [file Beilstein_J_Org_Chem-20-823-s002.zip › NMR files oxygen migration/11e/1H/pdata/1/email_cyrMe2RCl_1_1.pdf]

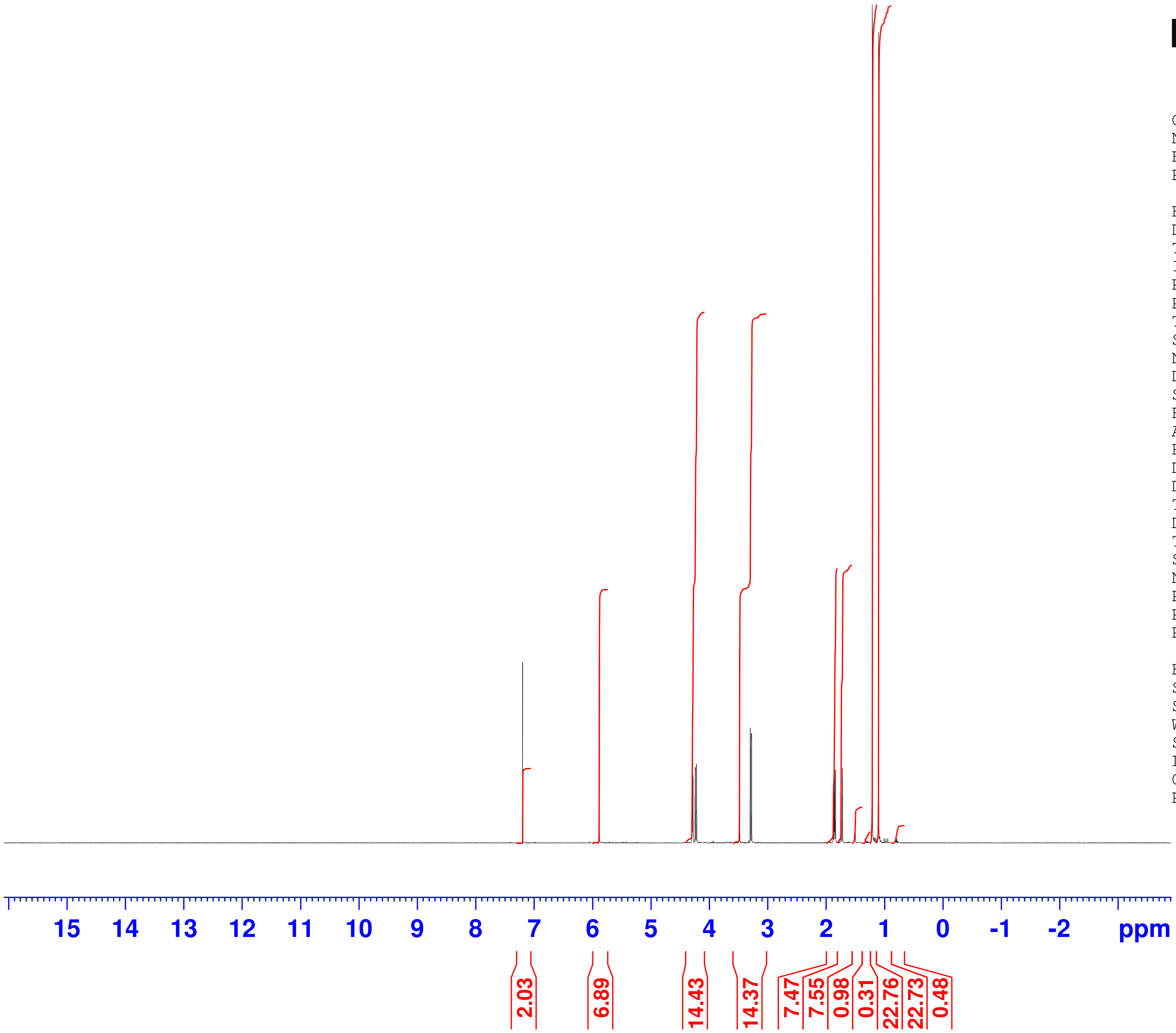


Current Data Parameters
NAME cyrMe2RCl
EXPNO 1
PROCNO 1

F2 - Acquisition Parameters
Date_ 20230909
Time 18.00 h
INSTRUM spect
PROBHD Z124627_0019 (
PULPROG zg30
TD 65536
SOLVENT CDCl3
NS 16
DS 2
SWH 10000.000 Hz
FIDRES 0.305176 Hz
AQ 3.2767999 sec
RG 191.36
DW 50.000 usec
DE 13.08 usec
TE 298.0 K
D1 1.00000000 sec
TD0 1
SFO1 500.1630885 MHz
NUC1 1H
P0 4.92 usec
P1 14.75 usec
PLW1 11.00000000 W

F2 - Processing parameters
SI 65536
SF 500.1600443 MHz
WDW EM
SSB 0
LB 0.30 Hz
GB 0
PC 1.00
