# Supplementary material for: Skeletal rearrangement of 6,8-dioxabicyclo[3.2.1]octan-4-ols promoted by thionyl chloride or Appel conditions
Source: Beilstein J Org Chem. 2024 Apr 16;20:823–9. doi: 10.3762/bjoc.20.74 (PMC11035982; doi:10.3762/bjoc.20.74)
Supplement: File 2 — 1H and 13C NMR FIDs, HRMS spectra for all new compounds. [file Beilstein_J_Org_Chem-20-823-s002.zip › NMR files oxygen migration/12c/12c HRMS.pdf]

Elemental Composition Report

Single Mass Analysis

Tolerance = 5.0 PPM / DBE: min = -1.5, max = 120.0
Element prediction: Off
Number of isotope peaks used for i-FIT = 3

Monoisotopic Mass, Even Electron Ions
6 formula(e) evaluated with 1 results within limits (all results (up to 1000) for each mass)
Elements Used:
C: 10-22 H: 5-26 O: 0-3 Na: 0-1

CyrBnMe2ROH
UNE BGreatrex CyrBnMe2ROH 259 (6.145) Cm (215:264)
1: TOF MS ES+
8.05e+003

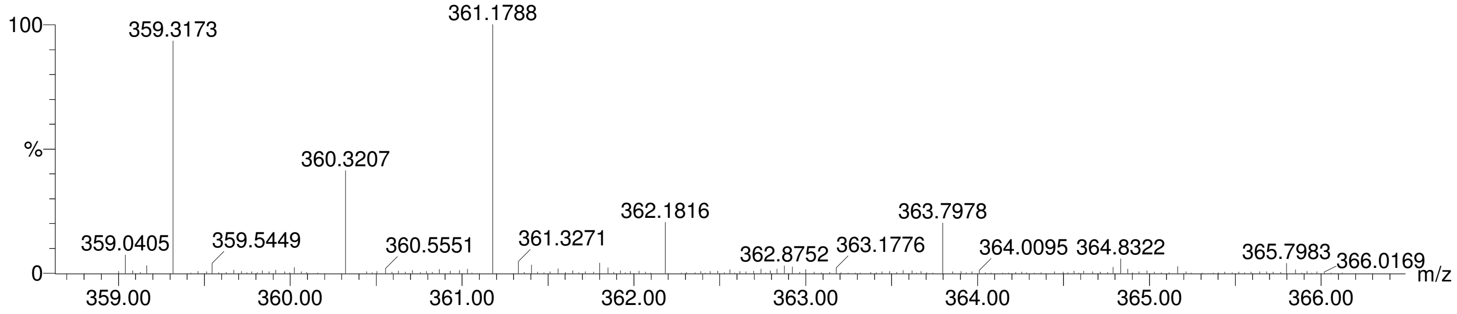

	Mass	Calc. Mass	mDa	PPM	DBE	i-FIT	i-FIT (Norm)	Formula
Minimum:					-1.5			
Maximum:			55.0	5.0	120.0			
	361.1788	361.1780	0.8	2.2	9.5	416.5	0.0	C22 H26 O3 Na
