# Supplementary material for: Skeletal rearrangement of 6,8-dioxabicyclo[3.2.1]octan-4-ols promoted by thionyl chloride or Appel conditions
Source: Beilstein J Org Chem. 2024 Apr 16;20:823–9. doi: 10.3762/bjoc.20.74 (PMC11035982; doi:10.3762/bjoc.20.74)
Supplement: File 2 — 1H and 13C NMR FIDs, HRMS spectra for all new compounds. [file Beilstein_J_Org_Chem-20-823-s002.zip › NMR files oxygen migration/12c/1H/pdata/1/email_cyrBnMe2ROH_fr_iPr2O_3_1.pdf]

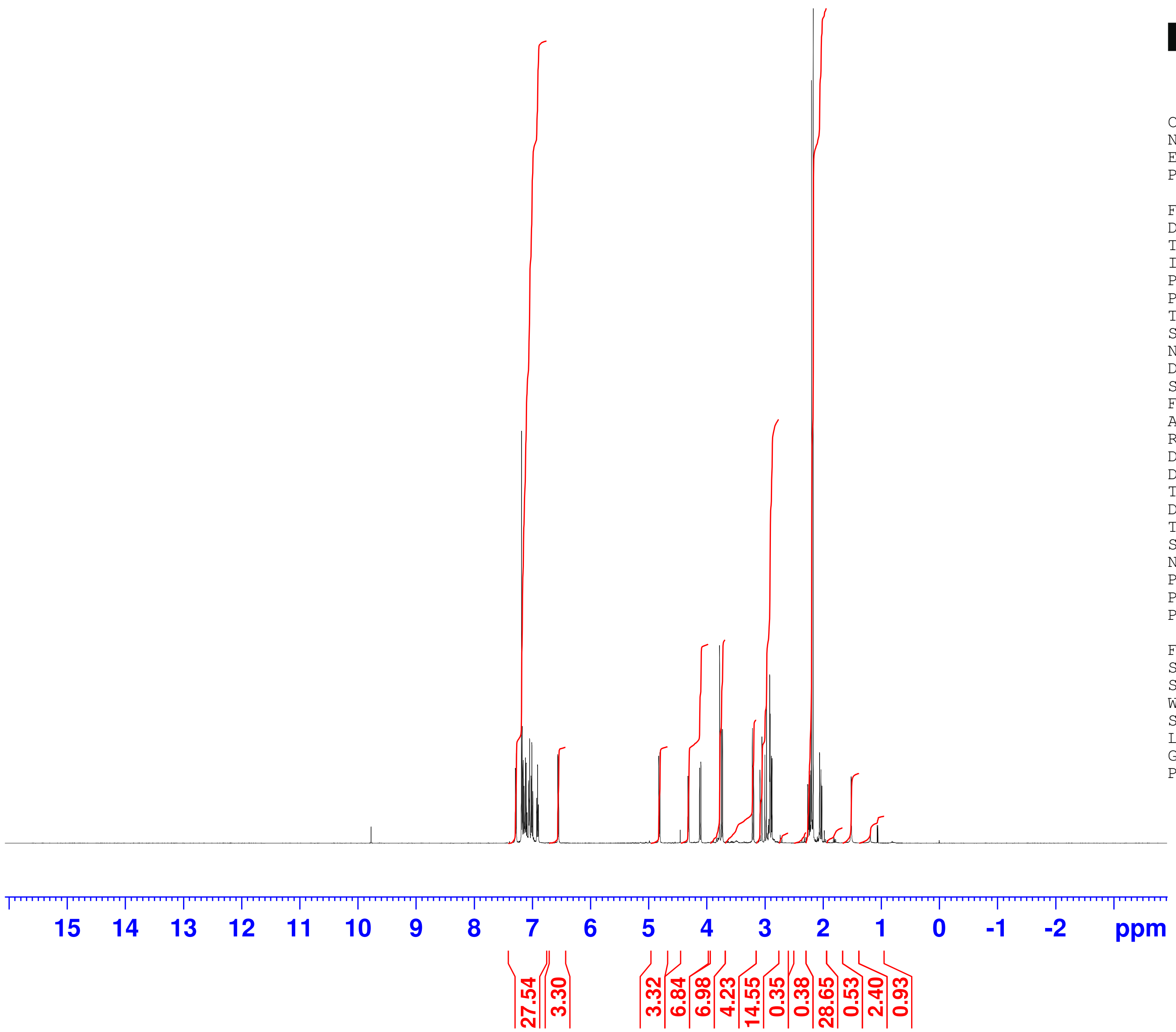

Current Data Parameters

NAME	cyrBnMe2ROH_fr_iPr2O
EXPNO	3
PROCNO	1

F2 - Acquisition Parameters

Date_	20231002
Time	15.00 h
INSTRUM	spect
PROBHD	Z124627_0019 (
PULPROG	zg30
TD	65536
SOLVENT	CDCl3
NS	16
DS	2
SWH	10000.000 Hz
FIDRES	0.305176 Hz
AQ	3.2767999 sec
RG	191.36
DW	50.000 usec
DE	13.08 usec
TE	298.0 K
D1	1.00000000 sec
TD0	1
SFO1	500.1630885 MHz
NUC1	1H
P0	4.92 usec
P1	14.75 usec
PLW1	11.00000000 W

F2 - Processing parameters

SI	65536
SF	500.1600500 MHz
WDW	EM
SSB	0
LB	0.30 Hz
GB	0
PC	1.00
