# Supplementary material for: Skeletal rearrangement of 6,8-dioxabicyclo[3.2.1]octan-4-ols promoted by thionyl chloride or Appel conditions
Source: Beilstein J Org Chem. 2024 Apr 16;20:823–9. doi: 10.3762/bjoc.20.74 (PMC11035982; doi:10.3762/bjoc.20.74)
Supplement: File 2 — 1H and 13C NMR FIDs, HRMS spectra for all new compounds. [file Beilstein_J_Org_Chem-20-823-s002.zip › NMR files oxygen migration/12d/1H/pdata/1/email_cyroxROH_1_1.pdf]

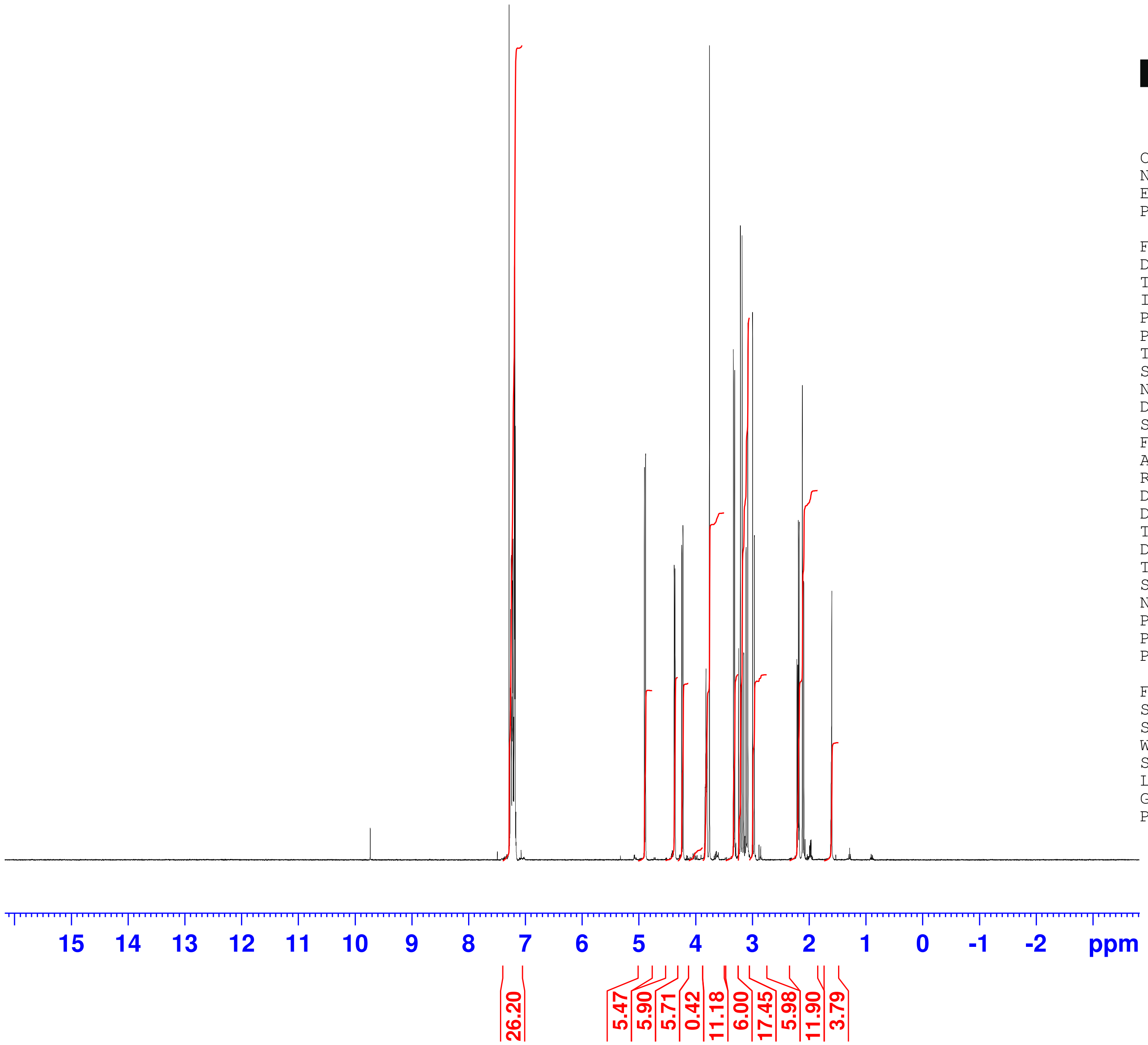


```
Current Data Parameters
NAME                cyroxROH
EXPNO                      1
PROCNO                     1

F2 - Acquisition Parameters
Date_               20230928
Time                   14.37 h
INSTRUM                spect
PROBHD     Z124627_0019 (
PULPROG                 zg30
TD                     65536
SOLVENT                CDCl3
NS                        16
DS                         2
SWH                10000.000 Hz
FIDRES              0.305176 Hz
AQ                 3.2767999 sec
RG                    191.36
DW                    50.000 usec
DE                     13.08 usec
TE                     298.0 K
D1                1.00000000 sec
TD0                        1
SFO1             500.1630885 MHz
NUC1                      1H
P0                      4.92 usec
P1                     14.75 usec
PLW1             11.00000000 W

F2 - Processing parameters
SI                     65536
SF               500.1600000 MHz
WDW                       EM
SSB                        0
LB                      0.30 Hz
GB                         0
PC                      1.00
```
