# Supplementary material for: Skeletal rearrangement of 6,8-dioxabicyclo[3.2.1]octan-4-ols promoted by thionyl chloride or Appel conditions
Source: Beilstein J Org Chem. 2024 Apr 16;20:823–9. doi: 10.3762/bjoc.20.74 (PMC11035982; doi:10.3762/bjoc.20.74)
Supplement: File 2 — 1H and 13C NMR FIDs, HRMS spectra for all new compounds. [file Beilstein_J_Org_Chem-20-823-s002.zip › NMR files oxygen migration/12f/12f HRMS.pdf]

Single Mass Analysis
Tolerance = 5.0 PPM / DBE: min = -1.5, max = 120.0
Element prediction: Off
Number of isotope peaks used for i-FIT = 3

Monoisotopic Mass, Even Electron Ions
10 formula(e) evaluated with 1 results within limits (all results (up to 1000) for each mass)
Elements Used:
C: 6-22 H: 5-26 O: 0-5 Na: 0-1
Cyr40MePh2ROH
UNE BGreatrex Cyr40MePh2ROH 49 (1.187) Cm (48:73) 1: TOF MS ES+
1.03e+004

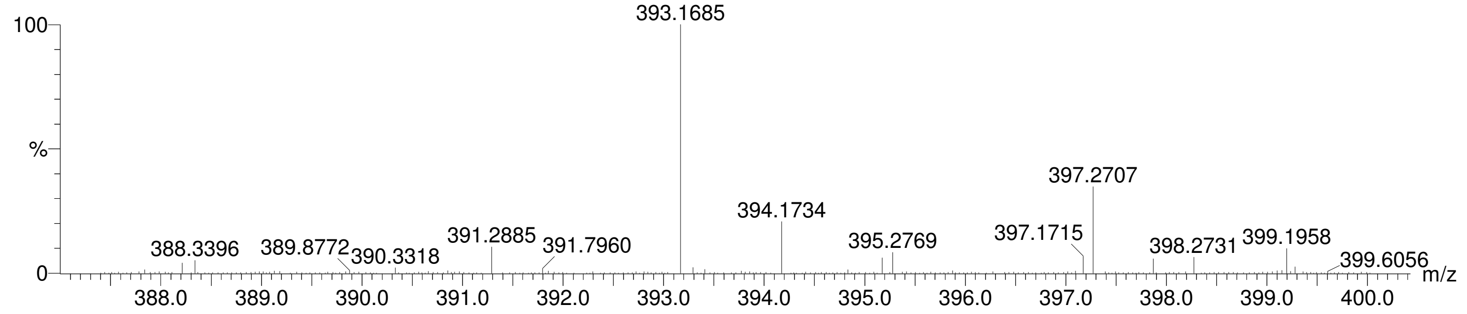

Minimum:				-1.5			
Maximum:		55.0	5.0	120.0			
Mass	Calc. Mass	mDa	PPM	DBE	i-FIT	i-FIT (Norm)	Formula
393.1685	393.1678	0.7	1.8	9.5	315.5	0.0	C22 H26 O5 Na
