# Supplementary material for: Skeletal rearrangement of 6,8-dioxabicyclo[3.2.1]octan-4-ols promoted by thionyl chloride or Appel conditions
Source: Beilstein J Org Chem. 2024 Apr 16;20:823–9. doi: 10.3762/bjoc.20.74 (PMC11035982; doi:10.3762/bjoc.20.74)
Supplement: File 2 — 1H and 13C NMR FIDs, HRMS spectra for all new compounds. [file Beilstein_J_Org_Chem-20-823-s002.zip › NMR files oxygen migration/13b/13b HRMS.pdf]

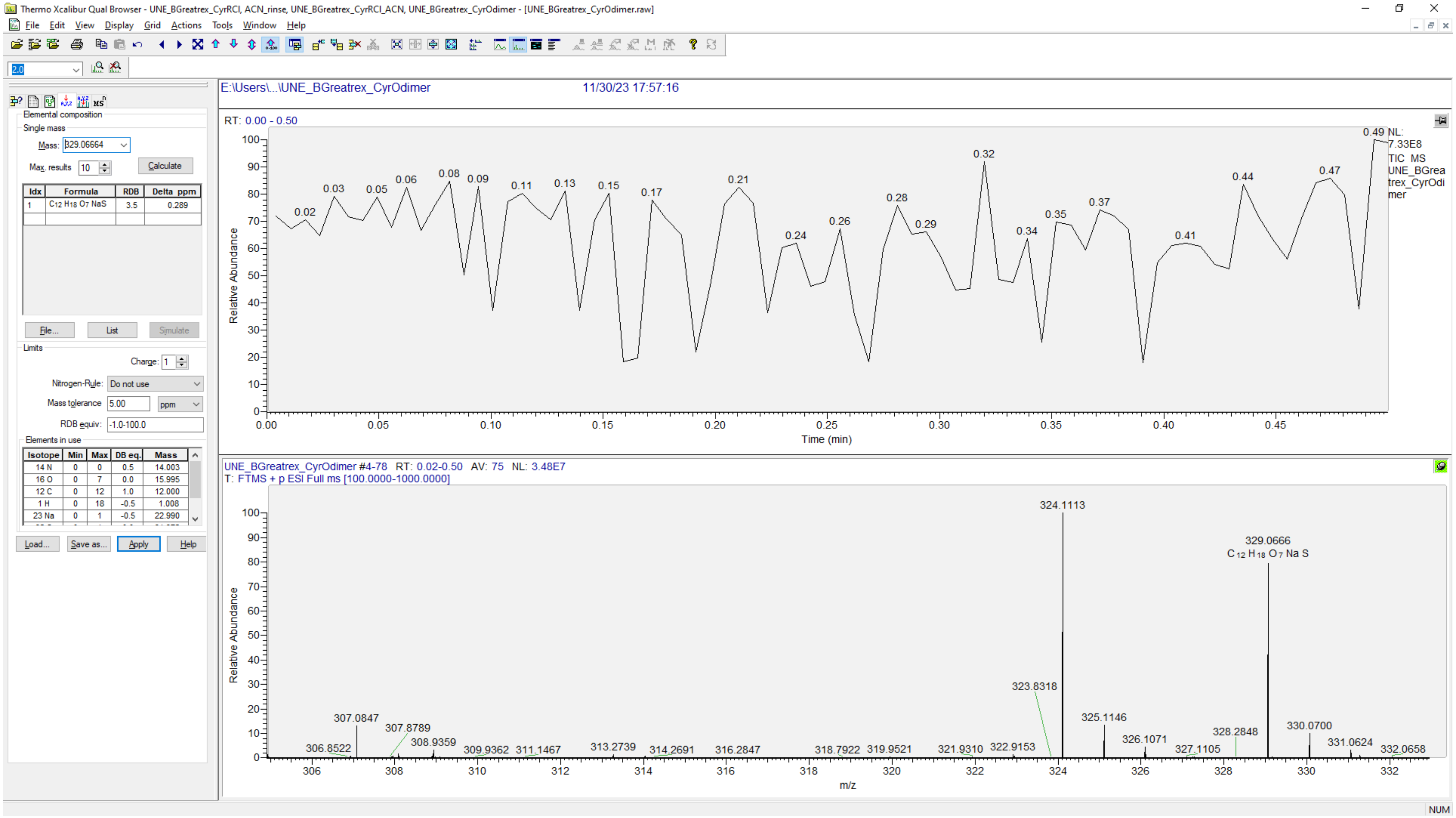

Elemental composition search on mass 329.07

m/z= 324.07-334.07

m/z	Theo. Mass	Delta (ppm)	RDB equiv.	Composition
329.0666	329.0665	0.29	3.5	$C_{12} H_{18} O_7$ Na S
