# Supplementary material for: Skeletal rearrangement of 6,8-dioxabicyclo[3.2.1]octan-4-ols promoted by thionyl chloride or Appel conditions
Source: Beilstein J Org Chem. 2024 Apr 16;20:823–9. doi: 10.3762/bjoc.20.74 (PMC11035982; doi:10.3762/bjoc.20.74)
Supplement: File 2 — 1H and 13C NMR FIDs, HRMS spectra for all new compounds. [file Beilstein_J_Org_Chem-20-823-s002.zip › NMR files oxygen migration/13b/13C/pdata/1/email_cyrenol_dimer_78mg_2_1.pdf]

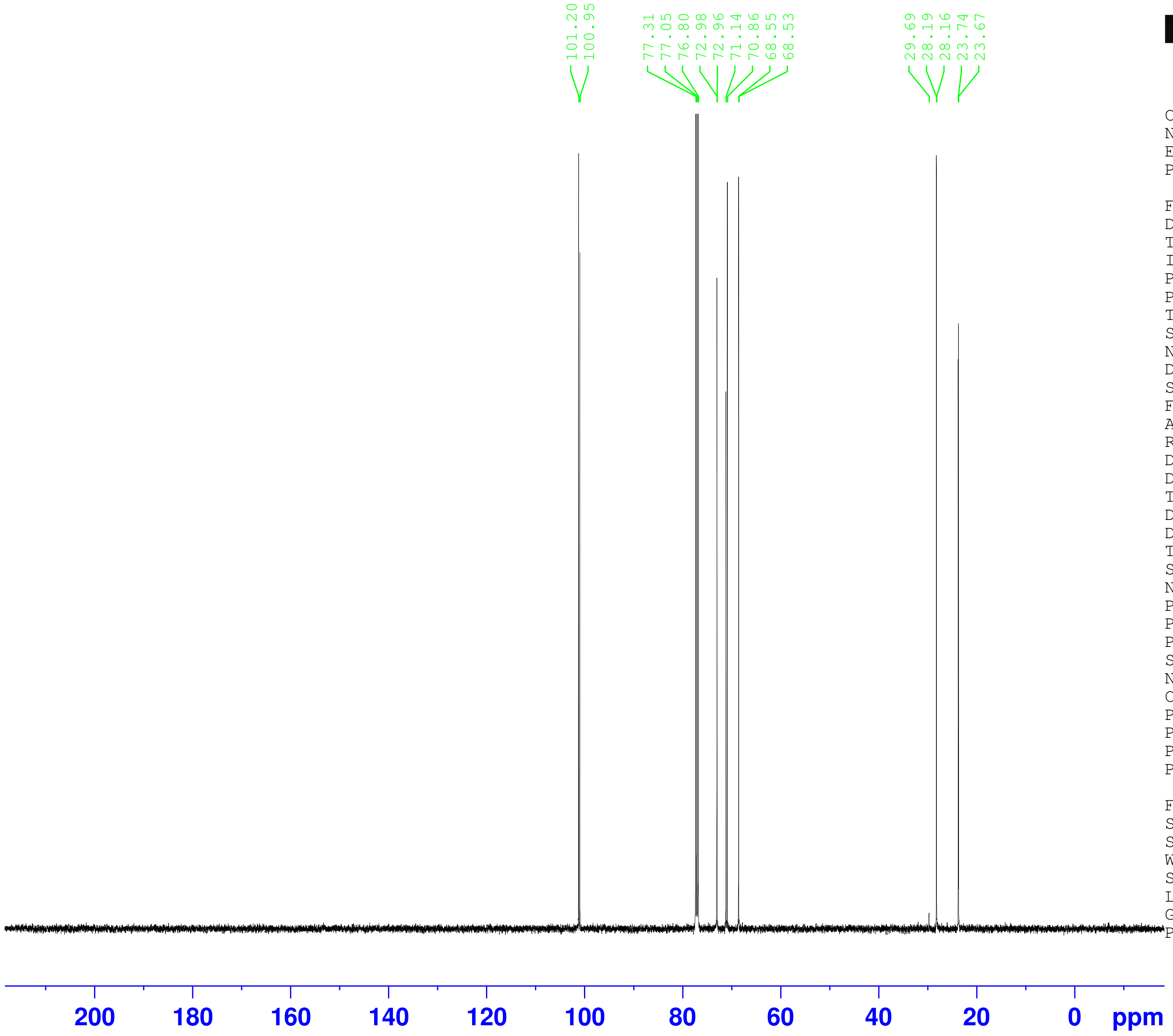


```
Current Data Parameters
NAME       cyrenol_dimer_78mg
EXPNO                       2
PROCNO                      1

F2 - Acquisition Parameters
Date_              20230923
Time                  15.53 h
INSTRUM               spect
PROBHD      Z124627_0019 (
PULPROG             zgpg30
TD                    65536
SOLVENT               CDCl3
NS                     1024
DS                        4
SWH               29761.904 Hz
FIDRES             0.908261 Hz
AQ                1.1010048 sec
RG                   191.36
DW                   16.800 usec
DE                     6.50 usec
TE                    298.0 K
D1               2.00000000 sec
D11              0.03000000 sec
TD0                       1
SFO1            125.7779086 MHz
NUC1                    13C
P0                     3.33 usec
P1                    10.00 usec
PLW1            41.00000000 W
SFO2            500.1620006 MHz
NUC2                     1H
CPDPRG[2            waltz65
PCPD2                 80.00 usec
PLW2            11.00000000 W
PLW12            0.37393999 W
PLW13            0.18809000 W

F2 - Processing parameters
SI                    32768
SF              125.7653320 MHz
WDW                      EM
SSB                       0
LB                     1.00 Hz
GB                        0
PC                     1.40
```
