# Supplementary material for: Skeletal rearrangement of 6,8-dioxabicyclo[3.2.1]octan-4-ols promoted by thionyl chloride or Appel conditions
Source: Beilstein J Org Chem. 2024 Apr 16;20:823–9. doi: 10.3762/bjoc.20.74 (PMC11035982; doi:10.3762/bjoc.20.74)
Supplement: File 2 — 1H and 13C NMR FIDs, HRMS spectra for all new compounds. [file Beilstein_J_Org_Chem-20-823-s002.zip › NMR files oxygen migration/13d/13C/pdata/1/email_cyroxOH_SOCl2_etoac_ext_highrf_6_1.pdf]

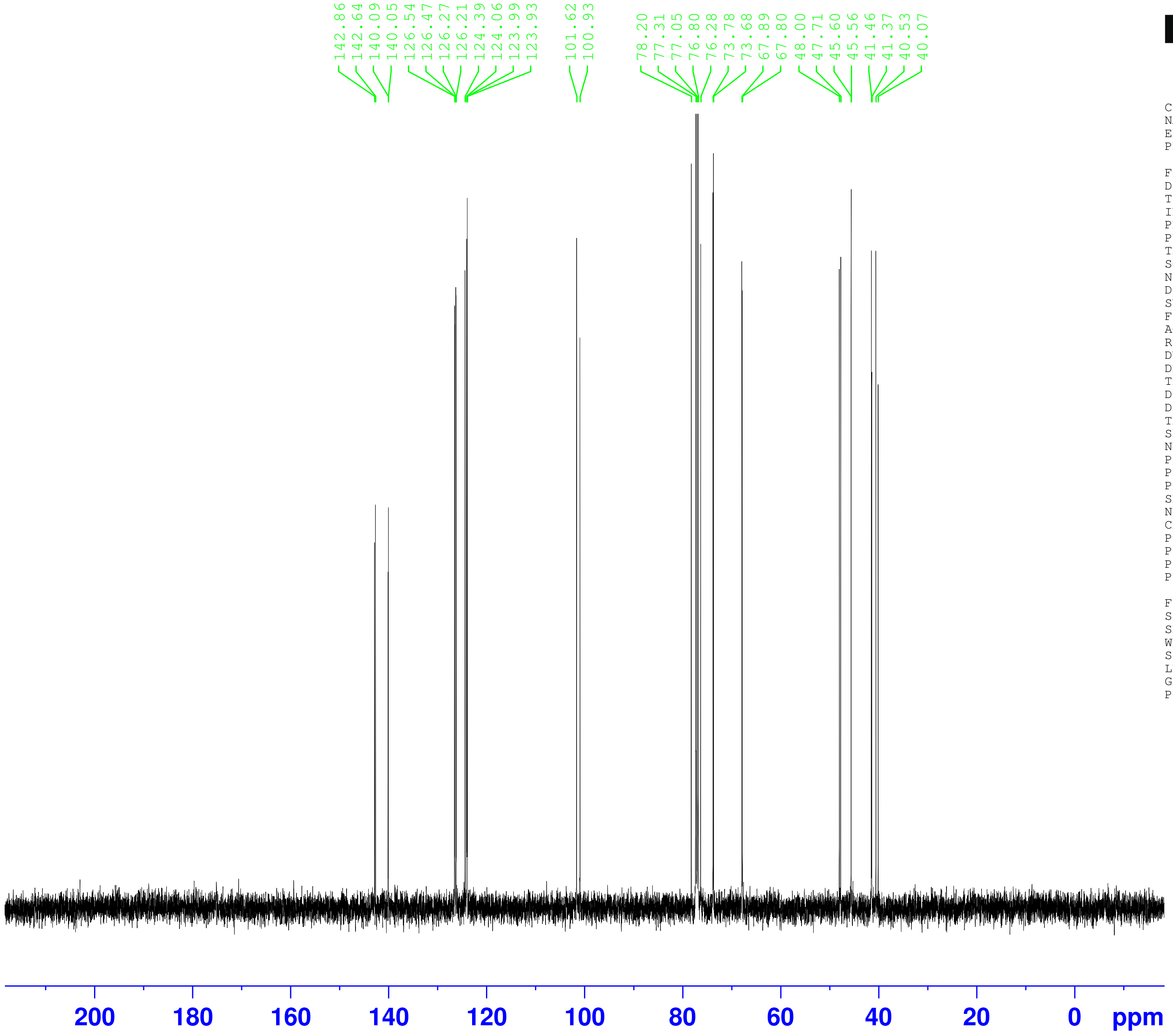

Current Data Parameters

NAME	cyroxOH_SOCl2_etoac_ext_highrf	
EXPNO	6	
PROCNO	1	

F2 - Acquisition Parameters

Date_	20230914	
Time	17.04	h
INSTRUM	spect	
PROBHD	Z124627_0019 (	
PULPROG	zgpg30	
TD	65536	
SOLVENT	CDCl3	
NS	256	
DS	4	
SWH	29761.904	Hz
FIDRES	0.908261	Hz
AQ	1.1010048	sec
RG	191.36	
DW	16.800	usec
DE	6.50	usec
TE	298.0	K
D1	2.00000000	sec
D11	0.03000000	sec
TD0	1	
SFO1	125.7779086	MHz
NUC1	13C	
P0	3.33	usec
P1	10.00	usec
PLW1	41.00000000	W
SFO2	500.1620006	MHz
NUC2	1H	
CPDPRG[2	waltz65	
PCPD2	80.00	usec
PLW2	11.00000000	W
PLW12	0.37393999	W
PLW13	0.18809000	W

F2 - Processing parameters

SI	32768	
SF	125.7653320	MHz
WDW	EM	
SSB	0	
LB	1.00	Hz
GB	0	
PC	1.40	
