# Supplementary material for: Skeletal rearrangement of 6,8-dioxabicyclo[3.2.1]octan-4-ols promoted by thionyl chloride or Appel conditions
Source: Beilstein J Org Chem. 2024 Apr 16;20:823–9. doi: 10.3762/bjoc.20.74 (PMC11035982; doi:10.3762/bjoc.20.74)
Supplement: File 2 — 1H and 13C NMR FIDs, HRMS spectra for all new compounds. [file Beilstein_J_Org_Chem-20-823-s002.zip › NMR files oxygen migration/13d/13d HRMS.pdf]

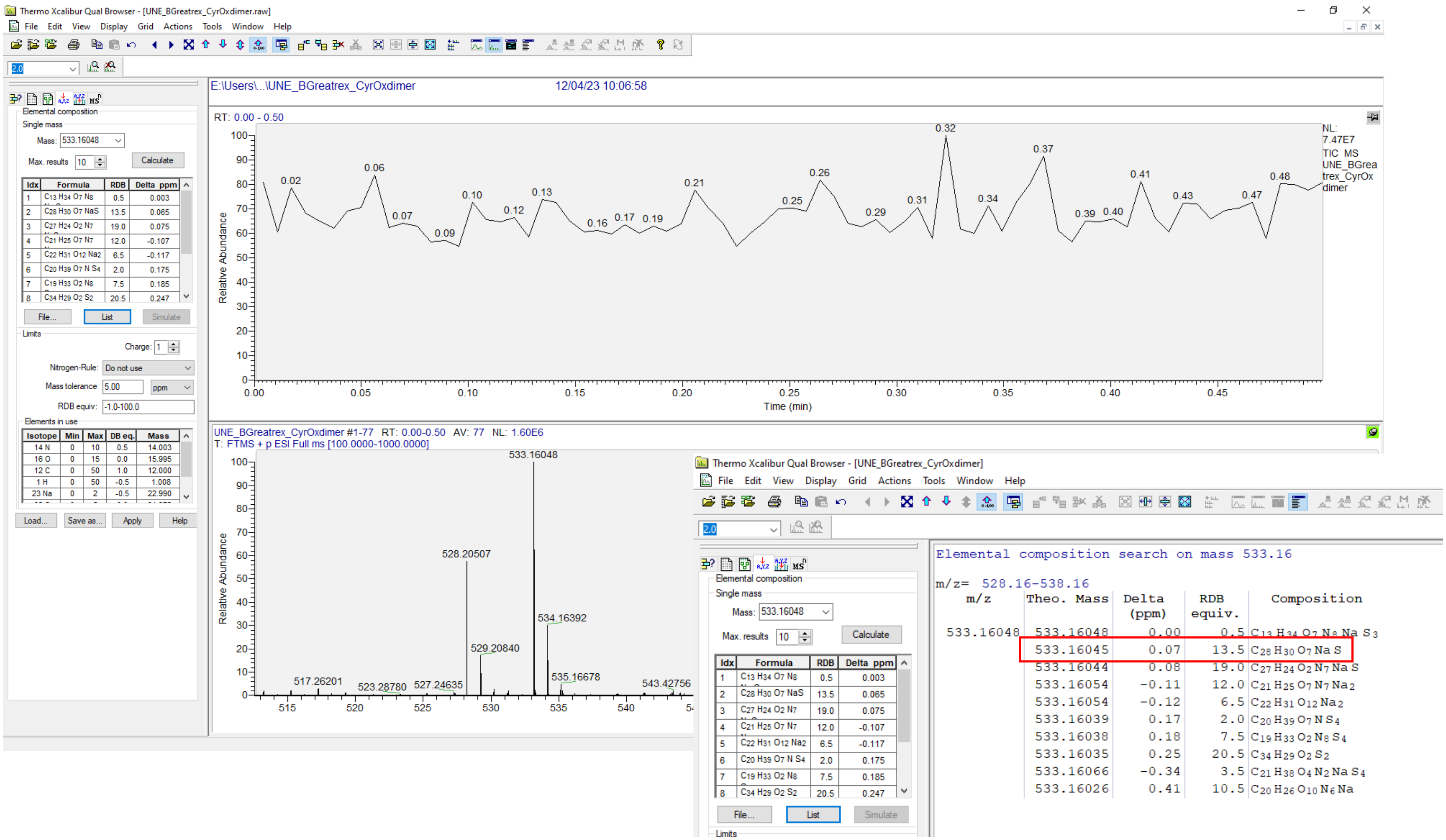
Thermo Xcalibur Qual Browser - [UNE_BGreatrex_CyrOxdimer.raw]
E:\Users\...\UNE_BGreatrex_CyrOxdimer
12/04/23 10:06:58
RT: 0.00 - 0.50
NL: 7.47E7 TIC MS UNE_BGreatrex_CyrOxdimer
Relative Abundance
Time (min)
UNE_BGreatrex_CyrOxdimer #1-77 RT: 0.00-0.50 AV: 77 NL: 1.60E6
T: FTMS + p ESI Full ms [100.0000-1000.0000]
533.16048
528.20507
534.16392
529.20840
535.16678
517.26201
523.28780
527.24635
543.42756
Elemental composition
Single mass
Mass: 533.16048
Max. results 10
Calculate
Charge: 1
Nitrogen-Rule: Do not use
Mass tolerance 5.00 ppm
RDB equiv: -1.0-100.0
Elements in use
Thermo Xcalibur Qual Browser - [UNE_BGreatrex_CyrOxdimer]
Elemental composition search on mass 533.16
m/z= 528.16-538.16
m/z | Theo. Mass | Delta (ppm) | RDB equiv. | Composition
533.16048 | 533.16048 | 0.00 | 0.5 | C13 H34 O7 N8 Na S3
533.16045 | 0.07 | 13.5 | C28 H30 O7 Na S
533.16044 | 0.08 | 19.0 | C27 H24 O2 N7 Na S
533.16054 | -0.11 | 12.0 | C21 H25 O7 N7 Na2
533.16054 | -0.12 | 6.5 | C22 H31 O12 Na2
533.16039 | 0.17 | 2.0 | C20 H39 O7 N S4
533.16038 | 0.18 | 7.5 | C19 H33 O2 N8 S4
533.16035 | 0.25 | 20.5 | C34 H29 O2 S2
533.16066 | -0.34 | 3.5 | C21 H38 O4 N2 Na S4
533.16026 | 0.41 | 10.5 | C20 H26 O10 N6 Na
