# Supplementary material for: Skeletal rearrangement of 6,8-dioxabicyclo[3.2.1]octan-4-ols promoted by thionyl chloride or Appel conditions
Source: Beilstein J Org Chem. 2024 Apr 16;20:823–9. doi: 10.3762/bjoc.20.74 (PMC11035982; doi:10.3762/bjoc.20.74)
Supplement: File 2 — 1H and 13C NMR FIDs, HRMS spectra for all new compounds. [file Beilstein_J_Org_Chem-20-823-s002.zip › NMR files oxygen migration/13e/13C/pdata/1/email_cyrMe2OH_SOCl2_etoac_extr_highRf_50mg_2_1.pdf]

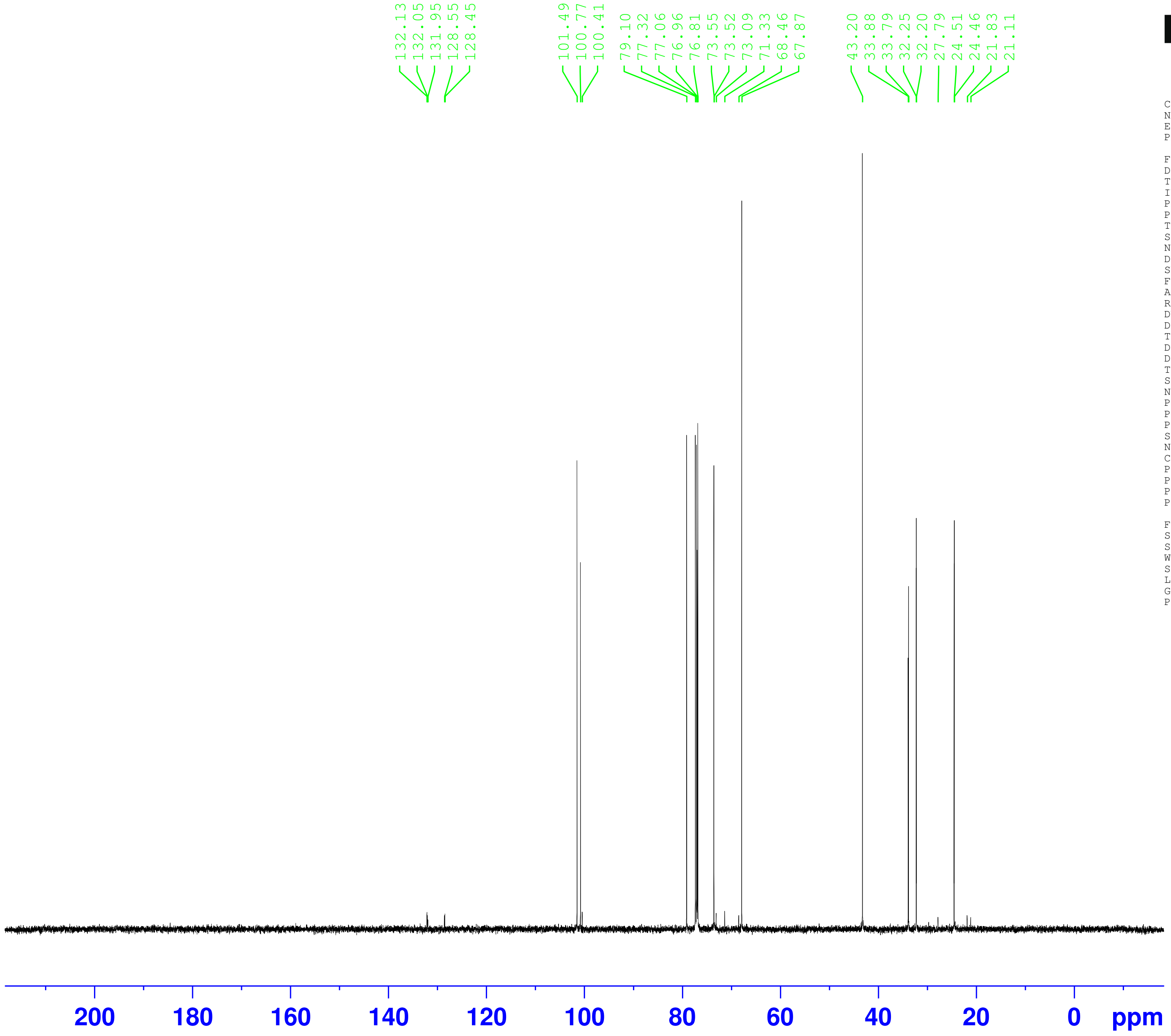


```
Current Data Parameters
NAME      cyrMe2OH_SOCl2_etoac_extr_highRf_50mg
EXPNO                  2
PROCNO                 1

F2 - Acquisition Parameters
Date_           20230914
Time               13.09 h
INSTRUM            spect
PROBHD   Z124627_0019 (
PULPROG           zgpg30
TD                 65536
SOLVENT            CDCl3
NS                   256
DS                     4
SWH            29761.904 Hz
FIDRES          0.908261 Hz
AQ             1.1010048 sec
RG                191.36
DW                16.800 usec
DE                  6.50 usec
TE                 298.0 K
D1            2.00000000 sec
D11           0.03000000 sec
TD0                    1
SFO1         125.7779086 MHz
NUC1                 13C
P0                  3.33 usec
P1                 10.00 usec
PLW1         41.00000000 W
SFO2         500.1620006 MHz
NUC2                  1H
CPDPRG[2         waltz65
PCPD2              80.00 usec
PLW2         11.00000000 W
PLW12         0.37393999 W
PLW13         0.18809000 W

F2 - Processing parameters
SI                 32768
SF           125.7653320 MHz
WDW                   EM
SSB                    0
LB                  1.00 Hz
GB                     0
PC                  1.40
```
