# Supplementary material for: Skeletal rearrangement of 6,8-dioxabicyclo[3.2.1]octan-4-ols promoted by thionyl chloride or Appel conditions
Source: Beilstein J Org Chem. 2024 Apr 16;20:823–9. doi: 10.3762/bjoc.20.74 (PMC11035982; doi:10.3762/bjoc.20.74)
Supplement: File 2 — 1H and 13C NMR FIDs, HRMS spectra for all new compounds. [file Beilstein_J_Org_Chem-20-823-s002.zip › NMR files oxygen migration/16/13C/pdata/1/email_64mg_KMnO4spot_column_appel_lgoallylOH_2_1.pdf]

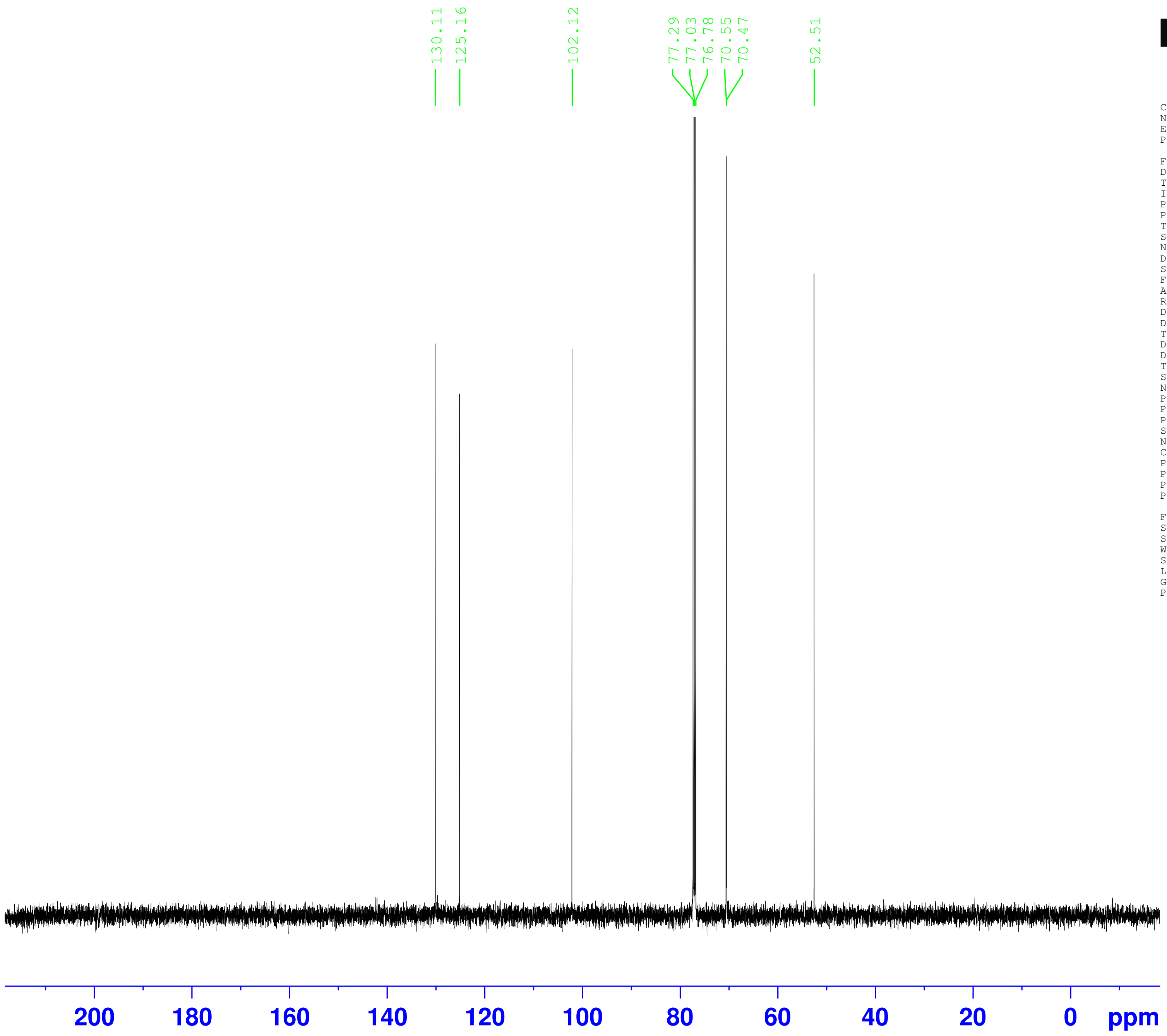

BRUKER

```
Current Data Parameters
NAME      64mg_KMnO4spot_column_appel_1goallylOH
EXPNO                       2
PROCNO                      1

F2 - Acquisition Parameters
Date_               20230903
Time                   17.36 h
INSTRUM                spect
PROBHD   Z124627_0019 (
PULPROG              zgpg30
TD                     65536
SOLVENT                CDCl3
NS                       256
DS                         4
SWH                29761.904 Hz
FIDRES              0.908261 Hz
AQ                 1.1010048 sec
RG                    191.36
DW                    16.800 usec
DE                      6.50 usec
TE                     298.0 K
D1                2.00000000 sec
D11               0.03000000 sec
TD0                        1
SFO1          125.7779086 MHz
NUC1                     13C
P0                      3.33 usec
P1                     10.00 usec
PLW1             41.00000000 W
SFO2          500.1620006 MHz
NUC2                      1H
CPDPRG[2             waltz65
PCPD2                  80.00 usec
PLW2             11.00000000 W
PLW12             0.37393999 W
PLW13             0.18809000 W

F2 - Processing parameters
SI                     32768
SF            125.7653320 MHz
WDW                       EM
SSB                        0
LB                      1.00 Hz
GB                         0
PC                      1.40
```
