# Supplementary material for: Skeletal rearrangement of 6,8-dioxabicyclo[3.2.1]octan-4-ols promoted by thionyl chloride or Appel conditions
Source: Beilstein J Org Chem. 2024 Apr 16;20:823–9. doi: 10.3762/bjoc.20.74 (PMC11035982; doi:10.3762/bjoc.20.74)
Supplement: File 2 — 1H and 13C NMR FIDs, HRMS spectra for all new compounds. [file Beilstein_J_Org_Chem-20-823-s002.zip › NMR files oxygen migration/17b/13C/pdata/1/email_LGO_allylOH_2SOCl25py_Fr3_6_1.pdf]

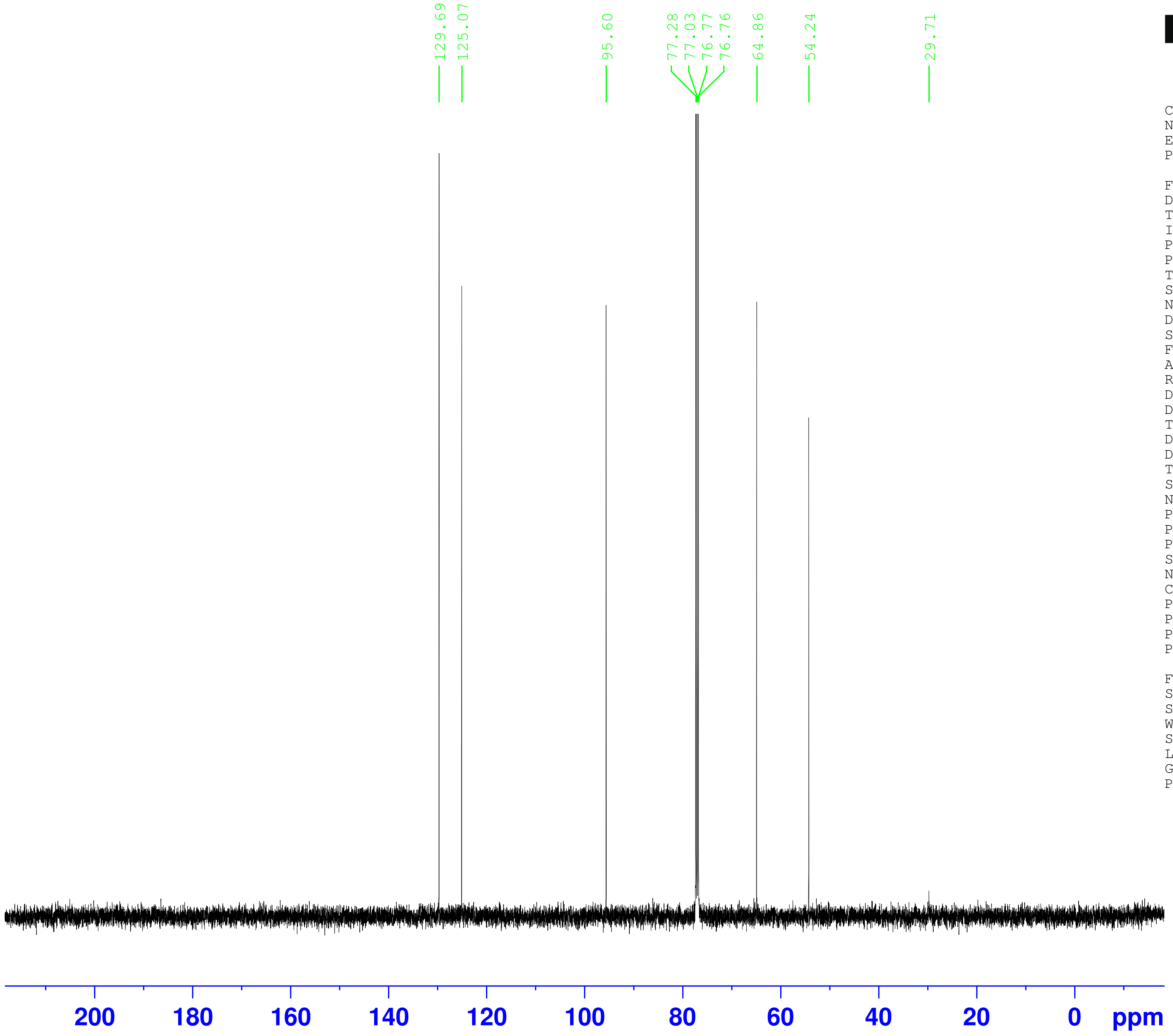


```
Current Data Parameters
NAME      LGO_allylOH_2SOCl25py_Fr3
EXPNO                     6
PROCNO                    1

F2 - Acquisition Parameters
Date_              20230917
Time                   2.13 h
INSTRUM               spect
PROBHD    Z124627_0019 (
PULPROG             zgpg30
TD                   65536
SOLVENT              CDCl3
NS                     512
DS                       4
SWH              29761.904 Hz
FIDRES            0.908261 Hz
AQ               1.1010048 sec
RG                  191.36
DW                  16.800 usec
DE                    6.50 usec
TE                   298.0 K
D1              2.00000000 sec
D11             0.03000000 sec
TD0                      1
SFO1           125.7779086 MHz
NUC1                   13C
P0                    3.33 usec
P1                   10.00 usec
PLW1           41.00000000 W
SFO2           500.1620006 MHz
NUC2                    1H
CPDPRG[2           waltz65
PCPD2                80.00 usec
PLW2           11.00000000 W
PLW12           0.37393999 W
PLW13           0.18809000 W

F2 - Processing parameters
SI                   32768
SF             125.7653320 MHz
WDW                     EM
SSB                      0
LB                    1.00 Hz
GB                       0
PC                    1.40
```
