# Supplementary material for: Skeletal rearrangement of 6,8-dioxabicyclo[3.2.1]octan-4-ols promoted by thionyl chloride or Appel conditions
Source: Beilstein J Org Chem. 2024 Apr 16;20:823–9. doi: 10.3762/bjoc.20.74 (PMC11035982; doi:10.3762/bjoc.20.74)
Supplement: File 2 — 1H and 13C NMR FIDs, HRMS spectra for all new compounds. [file Beilstein_J_Org_Chem-20-823-s002.zip › NMR files oxygen migration/21/21 HRMS.pdf]

Single Mass Analysis

Tolerance = 15.0 PPM / DBE: min = -1.5, max = 120.0
Element prediction: Off
Number of isotope peaks used for i-FIT = 3

Monoisotopic Mass, Even Electron Ions
7 formula(e) evaluated with 1 results within limits (all results (up to 1000) for each mass)
Elements Used:
C: 10-23 H: 5-27 O: 0-2 S: 0-1
CyrBn2Rallyl
UNE BGreatrex CyrBn2Rallyl 73 (1.754) Cm (71:161)   1: TOF MS ES+
1.10e+004

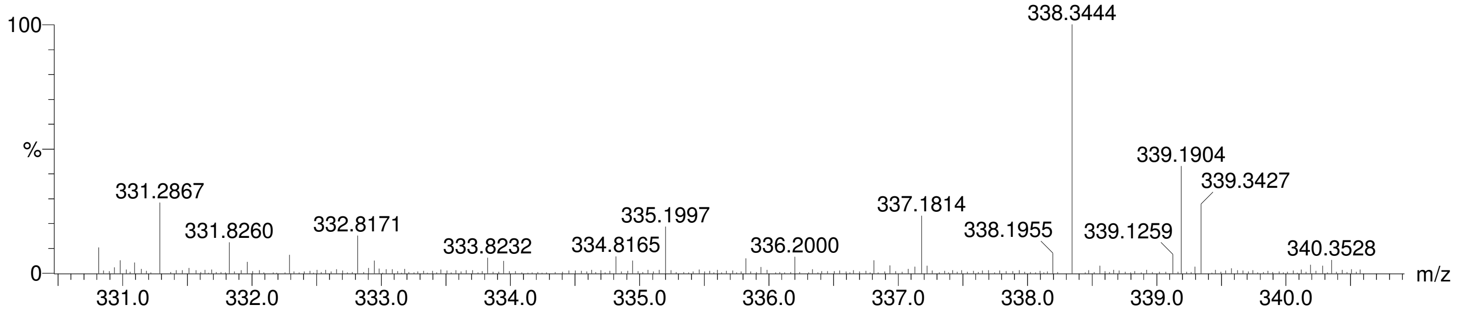

Mass	Calc. Mass	mDa	PPM	DBE	i-FIT	i-FIT (Norm)	Formula
Minimum:				-1.5			
Maximum:		55.0	15.0	120.0			
335.1997	335.2011	-1.4	-4.2	10.5	503.3	0.0	C23 H27 O2
