# Supplementary material for: Skeletal rearrangement of 6,8-dioxabicyclo[3.2.1]octan-4-ols promoted by thionyl chloride or Appel conditions
Source: Beilstein J Org Chem. 2024 Apr 16;20:823–9. doi: 10.3762/bjoc.20.74 (PMC11035982; doi:10.3762/bjoc.20.74)
Supplement: File 2 — 1H and 13C NMR FIDs, HRMS spectra for all new compounds. [file Beilstein_J_Org_Chem-20-823-s002.zip › NMR files oxygen migration/23/23 HRMS.pdf]

Single Mass Analysis

Tolerance = 5.0 PPM / DBE: min = -1.5, max = 120.0
Element prediction: Off
Number of isotope peaks used for i-FIT = 3

Monoisotopic Mass, Even Electron Ions
4 formula(e) evaluated with 1 results within limits (all results (up to 1000) for each mass)
Elements Used:
C: 10-32 H: 5-30 O: 0-3 Na: 0-1
CyrBn2RphOPh
UNE BGreatrex CyrBn2RphOPh 19 (0.479) Cm (19:100) 1: TOF MS ES+ 4.54e+004

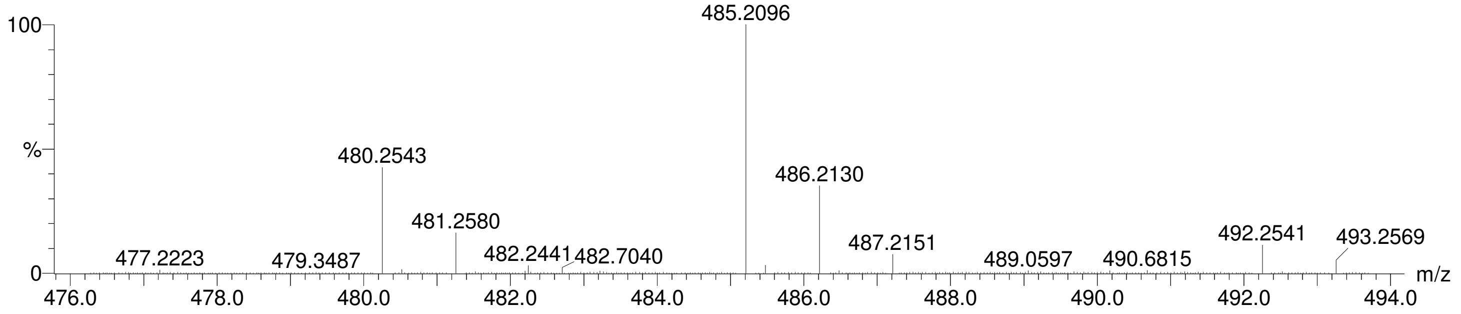

Minimum:				-1.5			
Maximum:		55.0	5.0	120.0			
Mass	Calc. Mass	mDa	PPM	DBE	i-FIT	i-FIT (Norm)	Formula
485.2096	485.2093	0.3	0.6	17.5	388.9	0.0	C32 H30 O3 Na
