# Supplementary material for: Skeletal rearrangement of 6,8-dioxabicyclo[3.2.1]octan-4-ols promoted by thionyl chloride or Appel conditions
Source: Beilstein J Org Chem. 2024 Apr 16;20:823–9. doi: 10.3762/bjoc.20.74 (PMC11035982; doi:10.3762/bjoc.20.74)
Supplement: File 2 — 1H and 13C NMR FIDs, HRMS spectra for all new compounds. [file Beilstein_J_Org_Chem-20-823-s002.zip › NMR files oxygen migration/9d/13C/pdata/1/JK-Aux100-01-2-C1-t30-34_2_1.pdf]

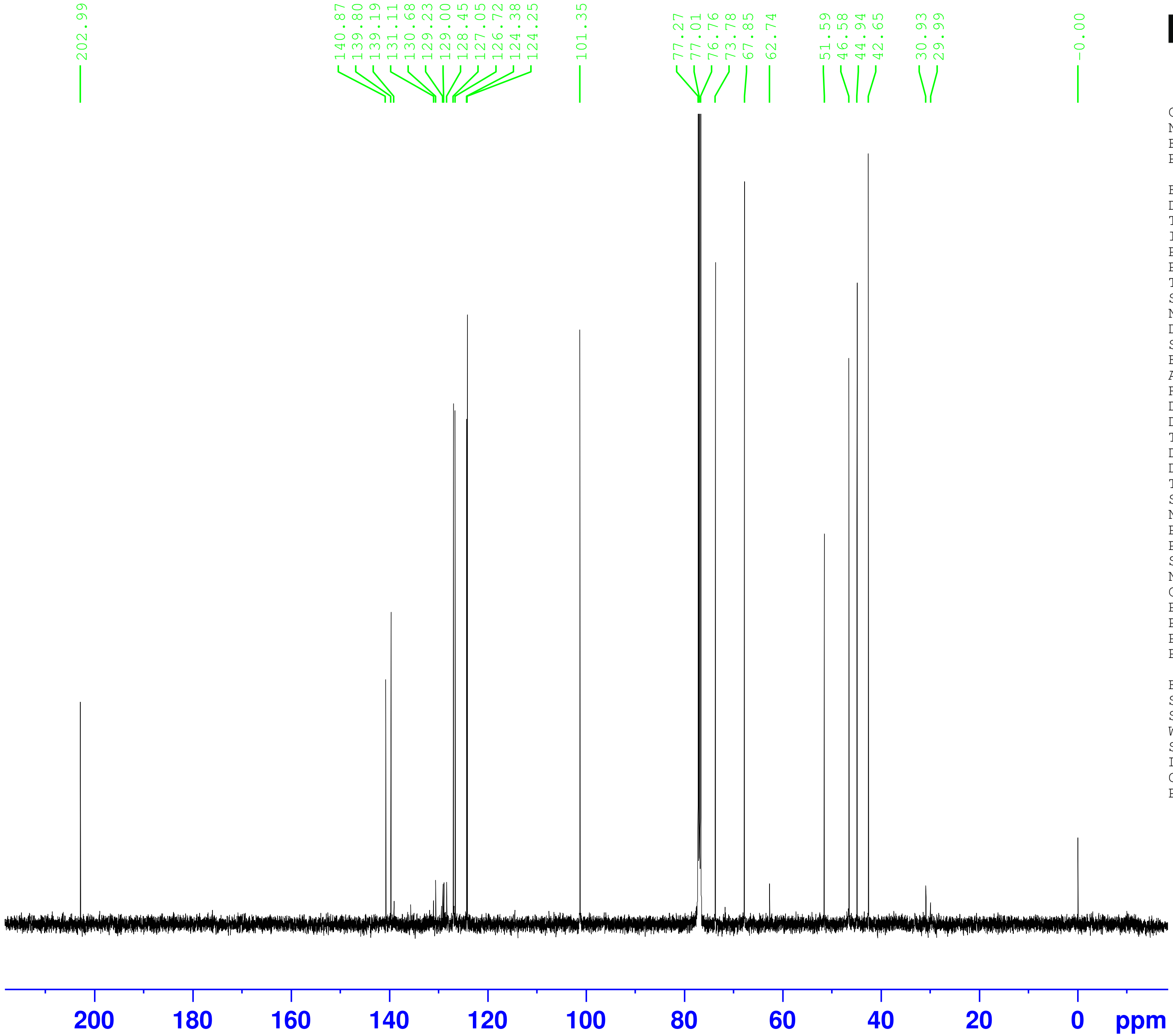

Current Data Parameters

NAME	JK-Aux100-01-2-C1-t30-34
EXPNO	2
PROCNO	1

F2 - Acquisition Parameters

Date_	20180223
Time	3.46 h
INSTRUM	spect
PROBHD	Z119470_0196 (
PULPROG	zgpg30
TD	65536
SOLVENT	CDCl3
NS	4000
DS	4
SWH	29761.904 Hz
FIDRES	0.908261 Hz
AQ	1.1010048 sec
RG	191.36
DW	16.800 usec
DE	6.50 usec
TE	295.0 K
D1	2.00000000 sec
D11	0.03000000 sec
TD0	1
SFO1	125.7779086 MHz
NUC1	13C
P1	10.00 usec
PLW1	78.00000000 W
SFO2	500.1620006 MHz
NUC2	1H
CPDPRG[2	waltz16
PCPD2	80.00 usec
PLW2	22.00000000 W
PLW12	0.35764000 W
PLW13	0.17989001 W

F2 - Processing parameters

SI	32768
SF	125.7653338 MHz
WDW	EM
SSB	0
LB	1.00 Hz
GB	0
PC	1.40
