# Supplementary material for: Skeletal rearrangement of 6,8-dioxabicyclo[3.2.1]octan-4-ols promoted by thionyl chloride or Appel conditions
Source: Beilstein J Org Chem. 2024 Apr 16;20:823–9. doi: 10.3762/bjoc.20.74 (PMC11035982; doi:10.3762/bjoc.20.74)
Supplement: File 2 — 1H and 13C NMR FIDs, HRMS spectra for all new compounds. [file Beilstein_J_Org_Chem-20-823-s002.zip › NMR files oxygen migration/9f/9f HRMS.pdf]

Single Mass Analysis
Tolerance = 5.0 PPM / DBE: min = -1.5, max = 120.0
Element prediction: Off
Number of isotope peaks used for i-FIT = 3

Monoisotopic Mass, Even Electron Ions
10 formula(e) evaluated with 1 results within limits (up to 20 closest results for each mass)
Elements Used:
C: 0-22 H: 0-26 O: 0-5 Na: 0-1
HBr7
UNE BGreatrex HBr7 40 (0.974) Cm (38:58)   1: TOF MS ES+ 2.43e+004

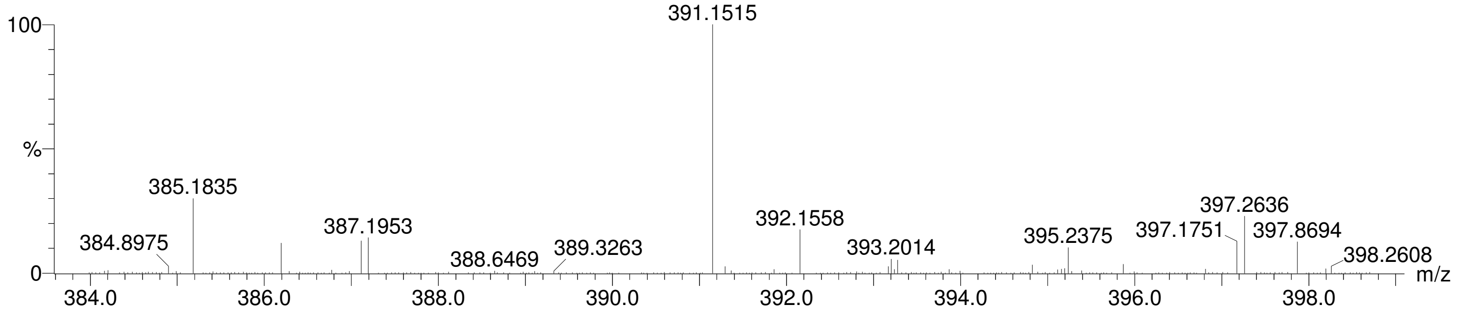

	Calc. Mass	mDa	PPM	DBE	i-FIT	i-FIT (Norm)	Formula
Minimum:				-1.5			
Maximum:		55.0	5.0	120.0			
**Mass**	**Calc. Mass**	**mDa**	**PPM**	**DBE**	**i-FIT**	**i-FIT (Norm)**	**Formula**
391.1515	391.1521	-0.6	-1.5	10.5	335.5	0.0	C22 H24 O5 Na
